# Dual gold and photoredox catalysis: visible light-mediated intermolecular atom transfer thiosulfonylation of alkenes†
†Electronic supplementary information (ESI) available. CCDC 1494115 for **3n** and 1494114 for **3s**. For ESI and crystallographic data in CIF or other electronic format see DOI: 10.1039/c6sc05093j



**DOI:** 10.1039/c6sc05093j

**Published:** 2017-01-04

**Authors:** Haoyu Li, Cuicui Shan, Chen-Ho Tung, Zhenghu Xu

**Affiliations:** a Key Lab for Colloid and Interface Chemistry of Education Ministry , School of Chemistry and Chemical Engineering , Shandong University , Jinan 250100 , People's Republic of China . Email: xuzh@sdu.edu.cn; b State Key Laboratory of Organometallic Chemistry , Shanghai Institute of Organic Chemistry , Chinese Academy of Sciences , Shanghai 200032 , PR China

## Abstract

Regioselective difunctionalization of alkenes has attracted significant attention from synthetic chemists and has the advantage of introducing diverse functional groups into vicinal carbons of common alkene moieties in a single operation. Herein, we report an unprecedented intermolecular atom transfer thiosulfonylation reaction of alkenes by combining gold catalysis and visible-light photoredox catalysis. A trifluoromethylthio group (SCF_3_) and other functionalized thio groups together with a sulfonyl group were regioselectively introduced into alkenes in the most atom economical manner. A detailed mechanism study indicated that a synergistic combination of gold catalysis and photoredox catalysis is crucial for this reaction.

## Abstract 2

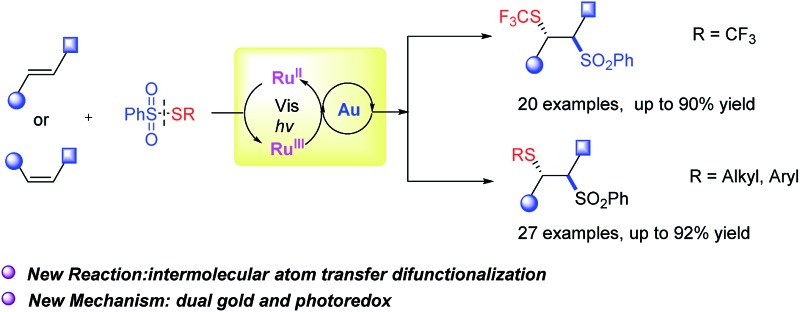
An unprecedented intermolecular atom transfer thiosulfonylation reaction of alkenes was achieved by combining Au catalysis and visible-light photoredox catalysis. A SCF_3_ group and other functionalized thio groups together with sulfonyl group were regioselectively introduced into alkenes.

## Introduction

Regioselective difunctionalization of alkenes has the advantage of introducing diverse functional groups into vicinal carbons of common alkene moieties in a single operation and has attracted extensive attention from synthetic chemists.^1^ Of the many catalytic processes that have been developed, a majority requires stoichiometric amounts of an external strong oxidant, such as PhI(OAc)_2_, Selectfluor (eqn (1)),^2^ and are typically initiated by a transition metal-catalyzed intramolecular addition. Intermolecular difunctionalization of alkenes is more challenging because of the regiochemical issue (eqn (2)). Recently, Stephenson developed an elegant visible light-mediated atom transfer radical addition reaction converting haloalkanes and α-halocarbonyl compounds into alkenes.^3^ These neutral redox reactions are very attractive because they are atom-economical and require no additional oxidants.1
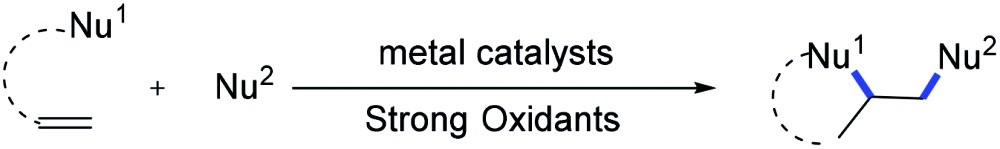

2
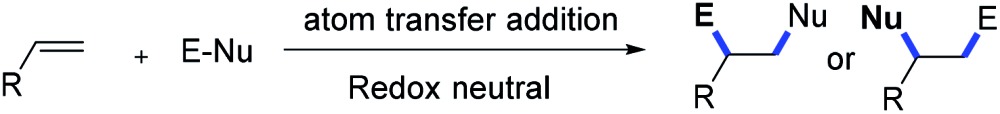



The trifluoromethylthio (SCF_3_) group is a key structural unit in many pharmaceutical and agrochemical products such as tiflorex, toltrazuril, and vaniliprole.^4^ It is well known that SCF_3_ groups in a molecule induce even higher lipophilicity than trifluoromethyl substituents, and consequently, the incorporation of SCF_3_ group into pharmaceuticals could greatly improve their ability to cross lipid membranes.^5^ Because of this, the introduction of a SCF_3_ group into small molecules has attracted significant attention in synthetic chemistry.^6,7^ Current methods for the construction of C–SCF_3_ bonds involve electrophilic trifluoromethylthio reagents^6^ or the nucleophilic AgSCF_3_ reagent.^7^ Sulfonyl groups are similar to carboxyl or phosphate groups in terms of molecular size and charge distribution, and the sulfonyl group has been introduced into bioactive molecules to improve their activity.^8^ A sulfonyl group has two receptors for hydrogen bonds and this can enhance the binding affinities of drug molecules with target proteins. Sulfones can be easily transformed into other functional groups, such as alkenes, *via* Julia olefination.^9^ We investigated that whether both SCF_3_ and sulfonyl groups could be simultaneously introduced into organic compounds in a single step. Moreover, this transformation has not been described to date. This report describes a dual gold and photoredox catalytic approach to the intermolecular atom transfer thiosulfonylation of alkenes.

**Scheme 1:**
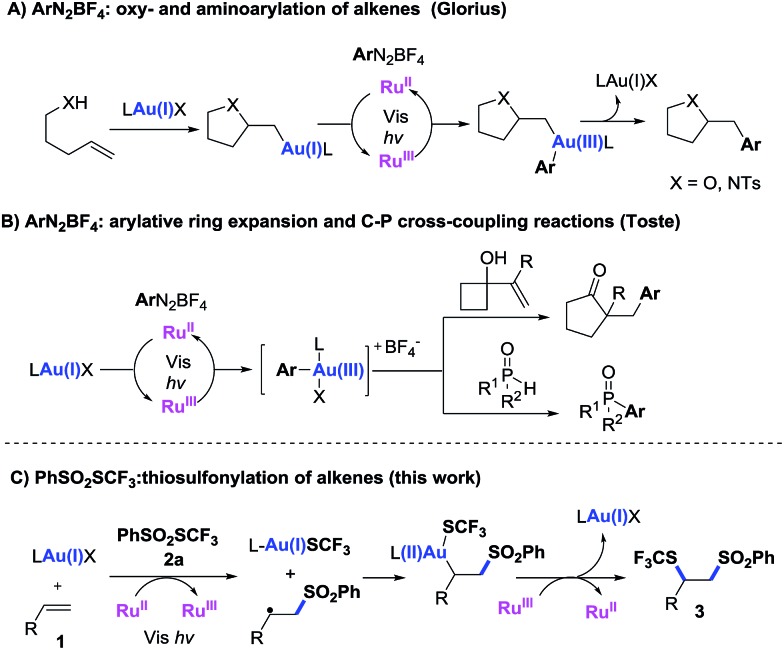
Dual gold and photoredox catalytic reactions.

A combination of visible light-mediated photoredox catalysis^10^ and transition-metal-catalysis is possible to bring two distinctive catalytic systems together and achieve unprecedented new reactions.^11^ Recently, the use of gold(i) complexes in photoredox catalysis has gained considerable attention.^12,13^ This photoredox catalytic cycle triggers the conversion of Au(i) into Au(iii) under mild conditions, a conversion which previously had only been achieved with stoichiometric quantities of strong oxidants.^14^ In 2013, Glorius reported a first dual gold and photoredox-catalyzed reaction, which achieved intramolecular oxy- and amino-arylation of alkenes with aryldiazonium salts (Scheme 1A).^12*b*^ Toste *et al.* took advantage of the visible light-mediated Au(i)/Au(iii) cycle to produce arylative ring expansion reactions and carbon–phosphorus cross-coupling reactions (Scheme 1B).^12d,e^ This visible light-mediated single electron oxidative reaction has been utilized to access gold(iii) complexes from gold(i) species.^12*k*^ Recently, Hashmi reported an aryldiazonium salts mediated Au(i) to Au(iii) transformation upon irradiation with blue LED in the absence of a photosensitizer.^12*q*^ In all these reactions,^12^ the same aryldiazonium salts were used. The development of new gold/photoredox catalysis mode is highly desirable. To achieve the proposed trifluoromethyl-thiosulfonylation reaction in the most atom-economical manner, a difunctionalization reagent, such as PhSO_2_SCF_3_ (**2a**), is required. This reagent can be easily prepared from PhSO_2_Na and AgSCF_3_ (for details, see the ESI†). We envisioned that the reaction of PhSO_2_SCF_3_ with a cationic gold catalyst in the presence of a photocatalyst would generate an LAuSCF_3_ species and a benzenesulfonyl radical, which can then add to the alkene forming a new alkyl radical.^3*a*^ This radical may oxidize LAuSCF_3_ into a Au(ii) intermediate, which is further oxidized to a Au(iii) derivative by the Ru^III^ catalyst. Subsequent reductive elimination forms the target difunctionalized product, regenerating the Au(i) catalyst (Scheme 1C). This reaction provided a new approach to introduce trifluoromethylthio (SCF_3_) group at the α position of styrenes.

## Results and discussion

To validate this concept, styrene (**1a**) and PhSO_2_SCF_3_ (**2a**) were selected as model substrates to test the feasibility of this hypothesis. After a detailed optimization of reaction conditions, the proposed alkene trifluoromethylthio-sulfonylation product (**3a**) was achieved in 94% yield under the standard conditions: a mixture of IPrAuCl (10 mol%), AgSbF_6_ (15 mol%), Ru(bpy)_3_Cl_2_ (2.5 mol%) in DCE (1 mL) was stirred under irradiation for 1–3 h with 100 W blue LED in a N_2_ atmosphere (Table 1, entry 1). The dual catalytic nature of this reaction was investigated by control experiments, which confirmed that the gold catalyst, the Ru(ii) photosensitizer, and visible light irradiation are all necessary for the reaction (Table S2,† entries 2–5). In the absence of silver salt, the reaction led to a dramatic decrease in the yields (NMR yields <5%), showing that the formation of a cationic gold species is highly important (Table S2,† entry 4). Other gold catalysts, such as AuCl, Ph_3_PAuCl, and Gagosz catalyst (Ph_3_PAuNTf_2_), are all less effective than IPrAuCl (entries 2–5). A silver free system using IPrAuSbF_6_ and Ru(bpy)_3_(PF6)_2_ led to a slightly lower yield (88%, entry 6); thus, silver is not necessary in this reaction. Reactions in other solvents, such as acetonitrile and methanol, led to only traces of products and no solvent addition products, indicating that a carbocation mechanism is not involved (entries 7 and 8). Other iridium photocatalysts including, Ir[dF(CF_3_)ppy]_2_(dtbbpy)PF_6_ and *fac*-Ir(ppy)_3_, were also tested and no product was observed in the reaction system (entries 10–11). The attempt to lower the catalysts loading also led to a lower reaction yield (entry 12).

**Table 1:** Optimization of the reaction conditions^*a*^

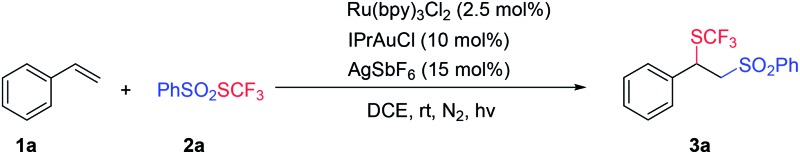		
Entry	Variation from the “standard” conditions	Yield^*b*^ (%)
1	None	94 (87)
2	AuCl instead of IPrAuCl	0
3	PPh_3_AuCl instead of IPrAuCl	56
4	IMesAuCl instead of IPrAuCl	72
5	PPh_3_AuNTf_2_ instead of IPrAuCl and AgSbF_6_	67
6^*c*^	IPrAuSbF_6_ instead of IPrAuCl and AgSbF_6_	88
7	CH_3_CN instead of DCE	5
8	CH_3_OH instead of DCE	4
9	Ru(phen)_3_Cl_2_ instead of Ru(bpy)_3_Cl_2_	67
10	Ir[dF(CF_3_)ppy]_2_(dtbbpy)PF_6_	0
11	*fac*-Ir(ppy)_3_ instead of Ru(bpy)_3_Cl_2_	0
12	IPrAuCl (5 mol%), AgSbF_6_ (7.5 mol%), Ru(bpy)_3_Cl_2_ (1.2 mol%)	64

^*a*^Reaction conditions: a mixture of **1a** (0.4 mmol), **2a** (0.2 mmol), IPrAuCl (10 mol%), AgSbF_6_ (15 mol%), Ru(bpy)_3_Cl_2_ (2.5 mol%), in DCE (1 mL) was stirred at rt under irradiation with a 100 W blue LED at N_2_ atmosphere.

^*b*^Determined by ^19^F NMR using (trifluoromethyl)benzene as the internal standard. The number in parentheses is the isolated yield. IPr = 1,3-bis(2,6-diisopropyl-phenyl)imidazol-2-ylidene, ppy = 2-phenylpyridine.

^*c*^Ru(bpy)_3_(PF_6_)_2_ instead of Ru(bpy)_3_Cl_2_.

With these optimized conditions established, the scope of the dual gold and visible light-mediated alkene difunctionalization reaction was explored. As summarized in Table 2, a wide range of styrene-type alkenes were applicable and moderate to good isolated yields of the product were achieved (**3a–3l**). Substrates bearing different electron-withdrawing or electron-donating groups at different positions in the aromatic ring were all compatible with this reaction. A series of functional groups, such as ester, cyano, trifluoromethyl, and halogen, were all well-tolerated under the reaction conditions. In particular, the alkene **1m**, containing an alkyne moiety, was also applicable to this reaction, giving the corresponding alkene difunctionalized product (**3m**) in moderate yield. Note that dienes, such as isoprene, were also amenable to this transformation, generating the 1,4-addition product (**3n**) as the major product in 55% yield, and its structure was confirmed by single crystal X-ray analysis.

**Table 2:** Substrate scope of alkene trifluoromethylthiosulfonylation reactions^*a*^

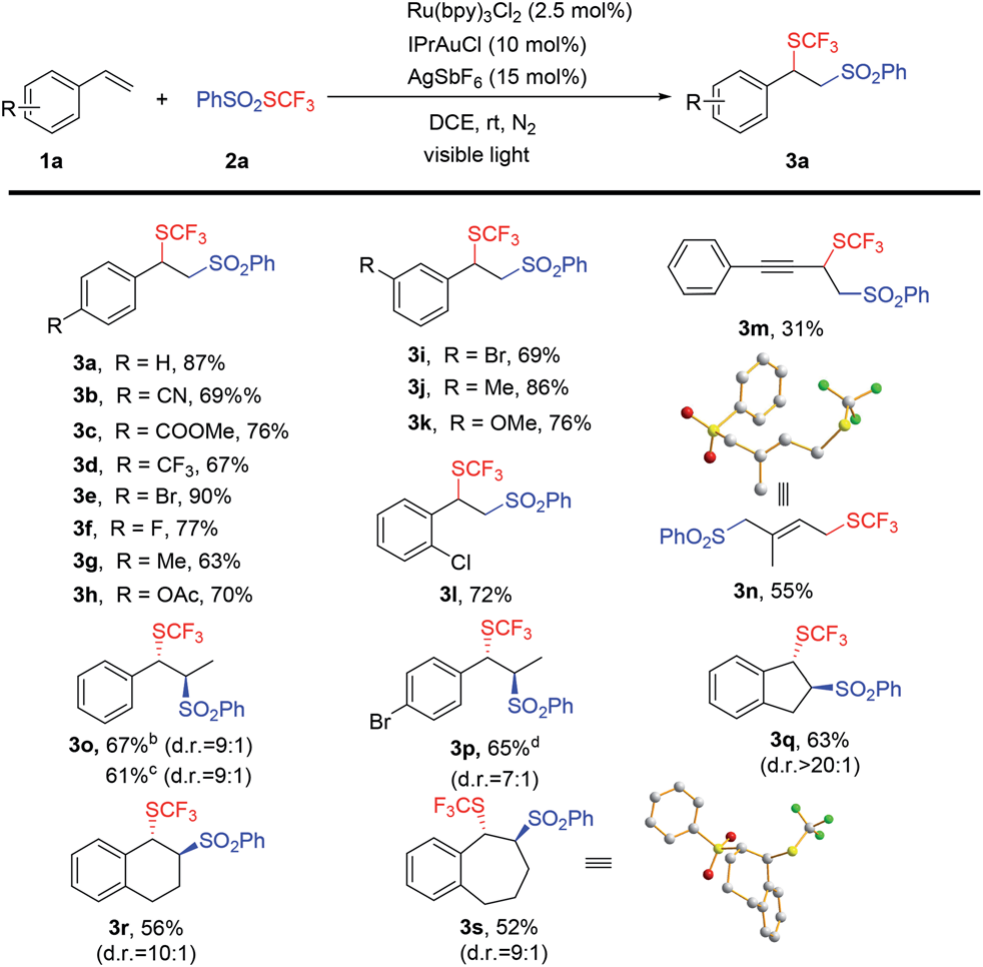

^*a*^Standard conditions were employed. Isolated yields were reported. Diastereoselectivities were determined by GC-mass.

^*b*^From (*E*)-alkene.

^*c*^From (*Z*)-alkene.

^*d*^From (*Z*)-alkene : (*E*)-alkene = 7 : 3.

Internal alkenes, including acyclic and cyclic alkenes, were also suitable substrates. Most of the reactions exhibited excellent diastereoselectivities. Interestingly, *trans*-alkenes and *cis*-alkenes afforded the same major products (**4o**) in similar yields with similar diastereoselectivities, which is a very important feature and also an advantage of this reaction. The relative configuration of the seven-membered product **3s** was unambiguously characterized by single X-ray crystallography. However, the reactions of aliphatic alkenes were unsuccessful under the same conditions.

Organosulfur compounds are ubiquitous in the pharmaceutical industry, materials science, and food chemistry.^15^ The construction of C–S bonds is important but challenging^16^ because a sulfur atom could coordinate with a metal catalyst, such as Au, leading to catalyst inactivation. When we applied the abovementioned dual catalysis system to the general thiosulfonylation reaction of alkenes using PhSO_2_SC_4_H_9_ (–1.64 V, *vs.* SCE) as the reagent, which might be more challenging because of its lower oxidative potential compared to that of PhSO_2_SCF_3_ (–1.11 V, *vs.* SCE, Fig. S5, ESI†), to our delight, the reactions were very successful (Table 3). Various alkenes, including styrenes, internal alkenes, and dienes, were all compatible with this thiosulfonylation reaction, giving the corresponding products in good yields (**4a–4r**). A large variety of alkyl and aromatic thio groups can be easily introduced into a styrene molecule, generating difunctional products generally in very good yields (**5a–5h**).

**Table 3:** Substrate scope of alkene thiosulfonylation reactions^*a*^

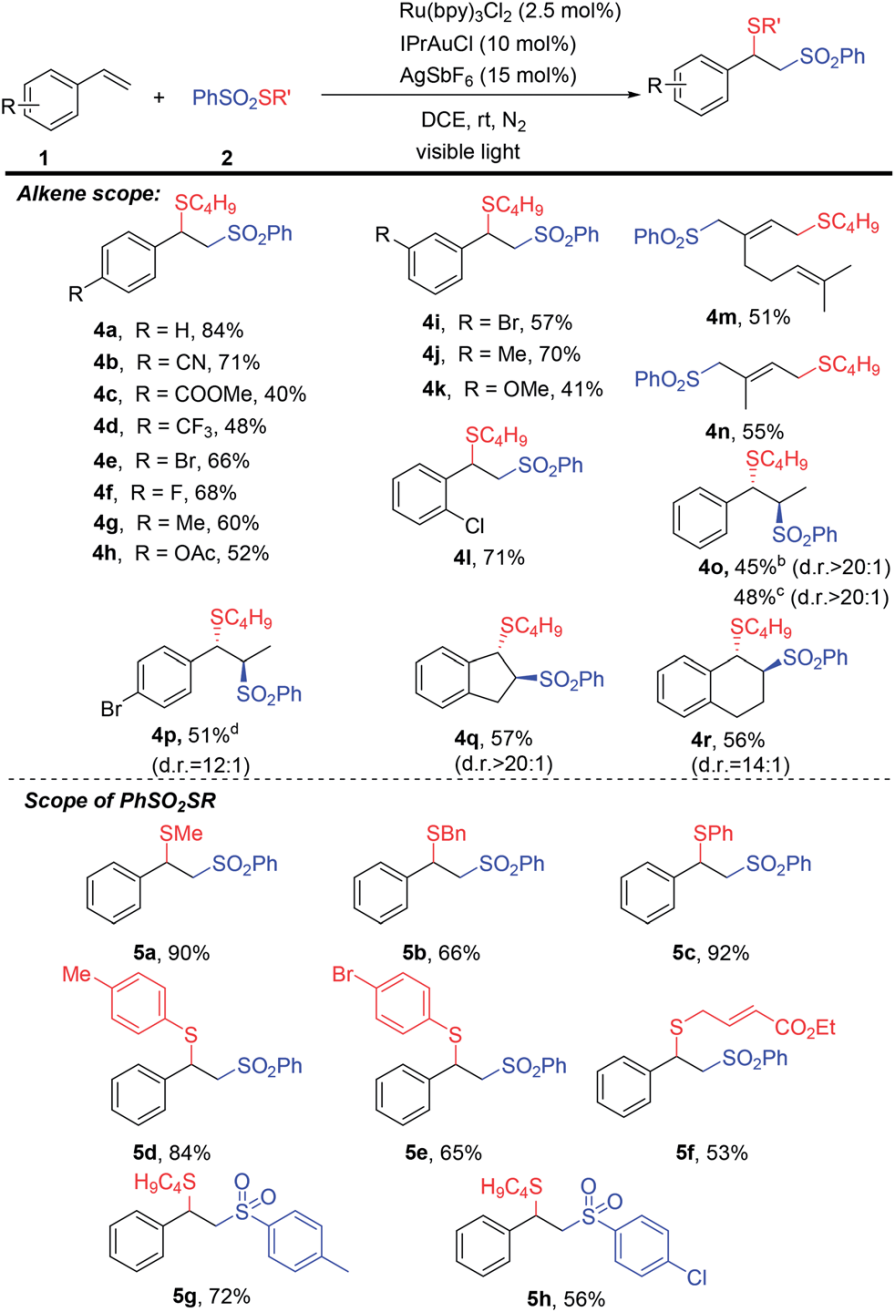

^*a*^Standard conditions were employed. Isolated yields were reported. Diastereoselectivities were determined by GC-mass.

^*b*^From (*E*)-alkene.

^*c*^From (*Z*)-alkene.

^*d*^From (*Z*)-alkene : (*E*)-alkene = 7 : 3.

The sulfonyl group (–SO_2_–) is a useful synthon for further transformations. For example, the reaction of compound **4a** with TMSCN or allylsilane in the presence of aluminium chloride provided the sulfur migration substitution products **6** and **7** in good yields (eqn (3) and (4)). This reaction might occur *via* a neighboring group participating in an S_N_1 type of reaction.3
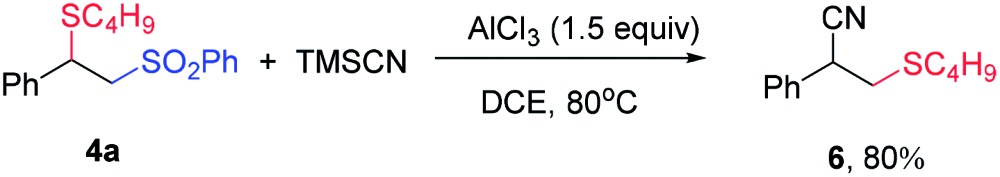

4
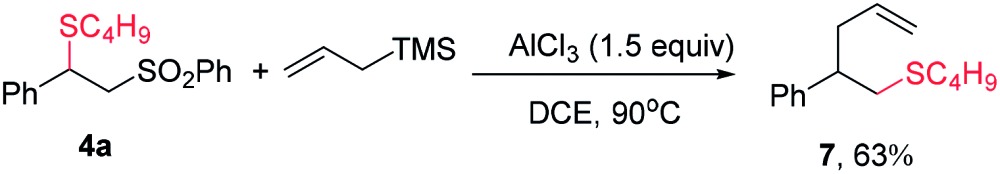



**Scheme 2:**
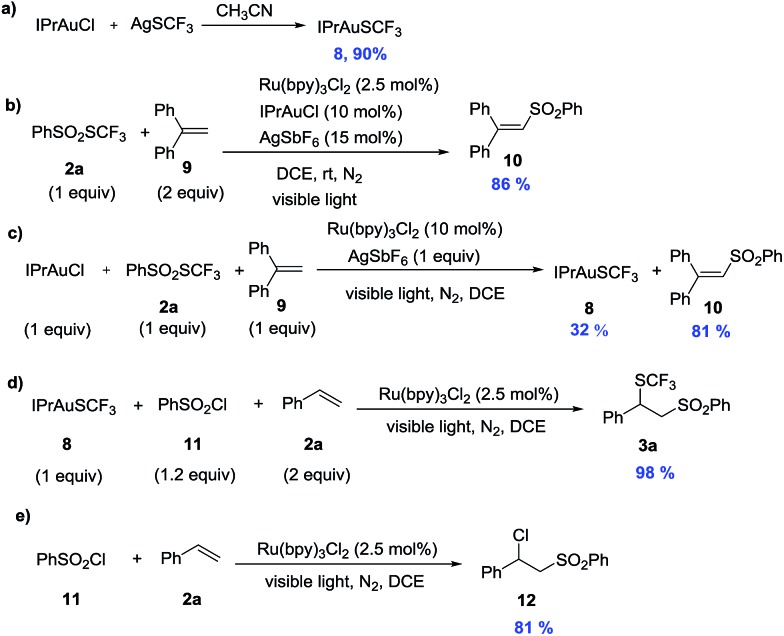
Control experiments.

Control experiments were conducted to explore the mechanism of this reaction. When the reaction of 1,1-diphenylethylene **9** was carried out under standard conditions, vinyl sulfone (**10**) was isolated in 86% isolated yield (Scheme 2b). These results clearly indicate the formation of a benzenesulfonyl radical in the reaction system. The ^19^F NMR spectrum of the crude mixture from the reaction exhibited an additional signal at –26 ppm, which was later determined to be from IPrAuSCF_3_ (**8**). This compound could be independently synthesized by the reaction of IPrAuCl and AgSCF_3_, which is sufficiently stable to be isolated by column chromatography (Scheme 2a). A stoichiometric reaction between IPrAuCl **2a** and the radical scavenger (**9**) afforded vinyl sulfone (**10**) in 81% isolated yield and **8** in 32% yield (Scheme 2c). This benzenesulfonyl radical could also be generated by PhSO_2_Cl (**11**) in the presence of visible light,^3*a*^ and the reaction between **8**, **11**, and **2a** afforded the target product (**3a**) in 98% yield (Scheme 2d). In contrast, without this gold intermediate, the direct atom transfer radical addition adduct **12** was obtained in 81% yield (Scheme 2e). This experiment demonstrated that IPrAuSCF_3_ is the possible reaction intermediate.^17^

Stern–Volmer fluorescence quenching experiments were performed to gain an insight into the photoredox catalytic cycle (for details, see the ESI (Fig. S1 and S2, ESI†)). The photoluminescence of Ru(bpy)_3_^2+^ was quenched by IPrAuSbF_6_ with a rate constant of 8.85 × 10^2^ L mol^–1^. In contrast, PhSO_2_SCF_3_ (–1.11 V *vs.* SCE) and styrene cannot serve as emission quenchers. The cyclic voltammogram of IPrAuSbF_6_ contains a reversible reduction peak at –0.11 V *vs.* SCE (Fig. S5, ESI†), indicating that this cationic gold catalyst is easily reduced by the excited state of the photocatalyst Ru(bpy)_3_^2+^ (*E*III/*II1/2 = –0.81 V *vs.* SCE).^10*b*^ To further characterize this electron transfer reaction, a flash-photolysis study was carried out (Fig. S3†). Upon laser excitation by 355 nm light, the ground state absorption at ∼450 nm was obviously bleached and a characteristic absorption band at ∼360 nm was detected. This is ascribed to the reductive state of bipyridine in Ru(bpy)_3_(SbF_6_)_2_ (Fig. S4-a, ESI†).^18^ When PhSO_2_SCF_3_ was introduced into a solution of Ru(bpy)_3_(SbF_6_)_2_, the transient absorption spectra did not show any difference (Fig. S4-b, ESI†). The lifetime of the excited state of Ru(bpy)_3_(SbF_6_)_2_ slightly decreased from 493 to 473 ns, as observed from the kinetics probed at 450 nm. However, when IPrAuSbF_6_ was added to a solution of Ru(bpy)_3_(SbF_6_)_2_, a new absorption peak appeared at ∼530 nm. This is characteristic of gold nanoparticles. At the same time, the lifetime of the excited state of Ru(bpy)_3_(SbF_6_)_2_ decreased from 493 to 394 ns (Fig. S4-c, ESI†). All these results suggested that the electron transfer between IPrAuSbF_6_ and the excited ^3^MLCT state of Ru(bpy)_3_(SbF_6_)_2_ occurred, generating the active IPrAu(0) species that might aggregate to form gold nanoparticles.^19^ The reductive IPrAu(0) catalyst formed *in situ* is highly reactive^20^ and might react with PhSO_2_SCF_3_, providing IPrAuSCF_3_ (**8**) and a benzenesulfonyl radical.

**Scheme 3:**
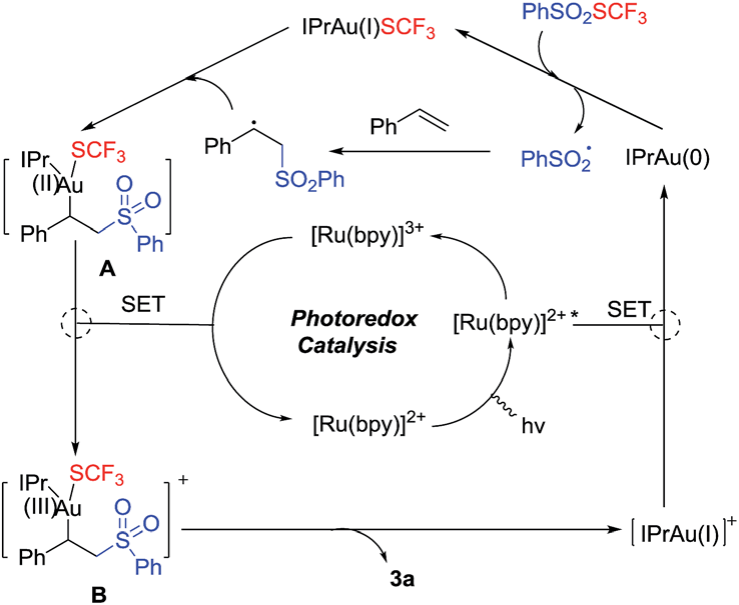
Proposed mechanism.

On the basis of the abovementioned results and previous reports,^12^ a tentative proposed mechanism is shown in Scheme 3. Irradiation of Ru(bpy)_3_^2+^ generates a long-lived photoexcited state Ru(bpy)_3_^2+*^, which undergoes a single-electron transfer reaction with cationic IPrAu(i) to initiate the catalytic cycle and provide Ru(bpy)_3_^3+^ and the highly active IPrAu(0), which can reduce PhSO_2_SCF_3_ to form IPrAu(i)SCF_3_ (**8**) and a benzenesulfonyl radical, which when added to styrene affords an alkyl radical. This radical is able to oxidize **8** to the Au(ii) intermediate **A**, which is further oxidized to the Au(iii) intermediate (**B**) by Ru(bpy)_3_^3+^, generating Ru(bpy)_3_^2+^ and thereby completing the photoredox catalytic cycle. Reductive elimination of the Au(iii) intermediate **B** delivers the product and regenerates the Au(i) catalyst. This alkyl radical directly reacting with another equivalent of PhSO_2_SCF_3_ could also afford the target product *via* a radical chain mechanism. In our reaction, when the internal alkenes were employed as reaction substrates, excellent diastereoselectivities were observed and both *trans*- and *cis*-alkenes yielded the same diastereomer, which is not common in a pure radical reaction. The IPrAu(i)SCF_3_ (**8**) approached the *in situ* formed alkyl radical from the less sterically hindered side, which might contribute to the excellent diastereoselectivity of this reaction. The proposed dual gold photoredox catalytic cycle may be the major pathway although the radical chain reaction couldn't be excluded. This gold catalytic cycle from Au(0) to Au(iii) is similar to that reported for nickel participating in photoredox catalytic cycles.

## Conclusions

In summary, we have reported an intermolecular atom transfer thiosulfonylation reaction of alkenes. Both trifluoromethylthio group and other functionalized thio groups can be introduced into alkenes with excellent regioselectivity and diastereoselectivity. These reactions are promoted by a synergistic combination of gold catalysis and visible light photoredox catalysis. Detailed control experiments and reaction mechanism studies indicate that the gold catalyst experiences four different valencies from Au(0) to Au(iii), which is unprecedented in previously reported Au-catalyzed transformations. This new dual gold and photocatalysis mode holds potential for applications to other important transformations.
