# Supplementary material for: Dual gold and photoredox catalysis: visible light-mediated intermolecular atom transfer thiosulfonylation of alkenes
Source: Chem Sci. 2017 Jan 4;8(4):2610–5. doi: 10.1039/c6sc05093j (PMC5431698; doi:10.1039/c6sc05093j)
Supplement: Supplementary file 1 [file SC-008-C6SC05093J-s001.pdf]

Electronic Supplementary Material (ESI) for Chemical Science.
This journal is © The Royal Society of Chemistry 2017

Supporting Information

Dual Gold and Photoredox Catalysis: Visible Light-Mediated Intermolecular Atom Transfer Thiosulfonylation of Alkenes

Haoyu Li, Cuicui Shan, Chen-Ho Tung and Zhenghu Xu

xuzh@sdu.edu.cn

General information………………………………………………….S2
Synthesis of the starting materials…………………………………S2
General procedure for Thiosulfonylation of Alkenes……………...S4
Optimization of light source……………………………………….S4
Mechanism Study……………………………..……………...S5
X-ray Crystallography Data …….………………………………...S9
Characterization Data………………………………..…………S10
NMR spectra for the products……………………………….…S22

General information

Unless otherwise noted, all the reagents were obtained commercially and used without further purification and reactions were monitored by TLC. Solvents used directly. All NMR spectra were recorded on Bruker-400 MHz spectrometer and Bruker-400 MHz spectrometer. HRMS were measured on the Q-TOF6510 instruments. The light source for the reaction is 100W 400nm blue LED and the total light intensity irradiated on the reaction vial was measured using a light intensity meter (model CEL-NP2000-10; Au light, China) and the value is ca. 120 mW/ cm^2.Thermo Scientific Lumina Fluorescence Instrument was used for Emission Quenching Experiments. In the transient absorption spectra measurements, excitation was provided by using an Nd:YAG laser and the detector was a xenon lamp on the Edingburge LP920 apparatus from Analytical Instruments.

Synthesis of the starting materials

1) But-3-en-1-yn-1-ylbenzene **1m** was prepared according to reported procedures[1].

   Typical procedure for the preparation of 4-Bromo-β-methylstyrene **1p.**

   Ethyltriphenylphosphonium bromide (3.7g, 10mmol) was dissolved in 50mL anhydrous THF under N_2 atmosphere and the suspension was cooled to -78 ℃. n-BuLi (2.5M in hexanes, 4.4mL, 11mmol) was added to the reaction mixture slowly and the mixture was stirred at 0 ℃ for an hour. After one hour, a solution of 4-bromobenzaldehyde (1.8g, 10mmol) in 10mL THF was slowly added to the flask at -78 ℃ and the mixture was stirred for two hours at room temperature. Brine (50mL) was added, and mixture was extracted with ethyl acetate (50mL, three times). The combined organic fractions were dried over $NaSO_4$. The reaction mixture was evaporated under reduced pressure and the residue was purified through column chromatography (petroleum ether: EtOAc = 50:1) afforded the desired product **1p** (1.4 g, 72% yield, (*Z*)-alkene: (*E*)-alkene = 7: 3) as a colourless oil. Known compound[2]. ^1H NMR (400 MHz, $CDCl_3$) δ 7.52-7.37 (m, 2H), 7.21-7.12 (m, 2H), 6.41 – 6.30 (m, 1H), 6.28-6.17 (m, 0.3H), 5.87-5.74 (m, 0.7H), 1.91-1.83 (m, 3H).

2) Typical procedure for the preparation of S-(trifluoromethyl) benzenesulfonothioate **2a**.

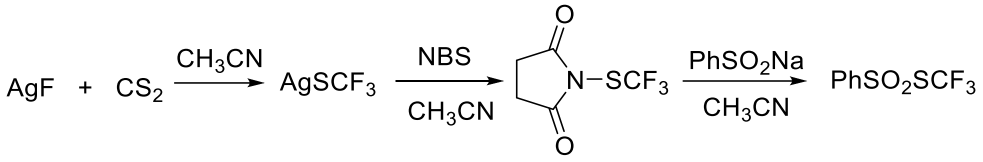

A mixture of AgF (10 g, mmol) and CS_2 20 mL in dry CH_3CN (60 mL) was refluxed at 80 °C for 16 h under N_2 atmosphere. The reaction mixture was filtered on celite and evaporated under reduced pressure. Pale yellow crude product $AgSCF_3$ was obtained. Then NBS (3.9 g, 22mmol) and CH_3CN (30 mL) were added to the flask with $AgSCF_3$.

The mixture was stirred for 2h and filtered on celite. After evaporating under reduced pressure, white solid was obtained. Then $PhSO_2Na$ (6.4 g, 40 mmol) and CH_3CN (30 mL) were added to the same flask and stirred at 10 °C for 1-3 h which was determined by TLC screening ($PhSO_2SCF_3$ was easy to decompose in the mixture). The reaction mixture was filtered on celite and evaporated under reduced pressure. The residue was purified through column chromatography (petroleum ether: EtOAc = 50:1) afforded the desired product **2a** (2.6 g, 54% yield) as a colourless oil. Known compound[3]. ^{1}H NMR (400 MHz, $CDCl_3$) δ 8.01 (d, *J* = 8.1 Hz, 2H), 7.74 (t, *J* = 7.5 Hz, 1H), 7.62 (t, *J* = 7.8 Hz, 2H).

Benzenesulfonothioates **2b-2h** were prepared according to reported procedure[4].

(1) Cheng, J.; Loh, T. *J. Am. Chem. Soc.* **2015**, *137*, 42.

(2) Zhang, L.; Dolbier, W. R.; Jr.; Sheeller, B.; Ingold, K. U. *J. Am. Chem. Soc.*, **2002**, *124*, 6362.

(3) Shao, X.; Xu, C.; Lu, L.; Shen, Q. *J. Org. Chem.*, **2015**, *80*, 3012.

(4) Wang, W.; Peng, X.; Wei, F.; Tung, C.; Xu, Z. *Angew. Chem. Int. Ed.* **2016**, *55*, 649.

General Procedure for the Thiosulfonylation of Alkenes

A mixture of alkene **1a** (0.4 mmol), thiosulfonylation reagent **2a** (0.2 mmol), IPrAuCl (10 mol%), $AgSbF_6$ (15 mol%), $Ru(bpy)_3Cl_2$ (2.5 mol%), DCE (1 mL) was stirred at room temperature under irradiation with 100 w blue LED at N_2 atmosphere for 1-3 h. The organic layer was filtered on celite and evaporated under reduced pressure. The crude reaction mixture was purified by silica gel flash chromatography to afford the desired product.

Optimization of light source

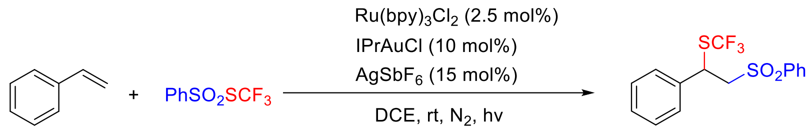

Entry	Light Source	Yield (%)
1	100W (100W*1,400nm)	94(87)
2	3W (3W*1,450nm)	0
3	21W (3W*7, 450nm)	68
4	50W (5W*10, 450nm)	90

Table S1. Reaction conditions: a mixture of **1a** (0.4 mmol), **2a** (0.2 mmol), IPrAuCl (10 mol%), $AgSbF_6$ (15 mol%), $Ru(bpy)_3Cl_2$ (2.5 mol%), in DCE (1 mL) was stirred at room temperature under irridiation of LED at N_2 atmosphere.

Mechanism Study

Control Experiments

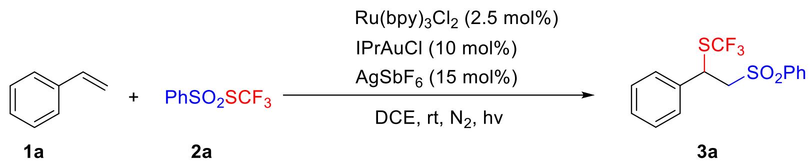

Entry	Variation from the "standard" conditions	Yield (%)
1	None	94 (87)
2	No IPrAuCl	<5
3	No $Ru(bpy)_3Cl_2$	0.
4	No $AgSbF_6$	<5
5	No light	0.

Table S2. Reaction conditions: a mixture of **1a** (0.4 mmol), **2a** (0.2 mmol), IPrAuCl (10 mol%), $AgSbF_6$ (15 mol%), $Ru(bpy)_3Cl_2$ (2.5 mol%), in DCE (1 mL) was stirred at rt under irradiation with 100 w 400nm LED at N_2 atmosphere.

Emission Quenching Experiments for $Ru(bpy)_3(SbF_6)_2$

Emission intensities were recorded using a HITACHI F-4500 Fluorescence Spectrometer. All $Ru(bpy)_3(SbF_6)_2$ solutions were excited at 450 nm and the emission intensity at 600 nm was observed. In the typical experiment, the $Ru(bpy)_3(SbF_6)_2$ solution was prepared by stirring the mixture of $Ru(bpy)_3Cl_2$•$6H_2O$ (0.01 mmol) and $AgSbF_6$ (0.02 mmol) in DCE (200 mL) for 5 minutes and filtering the precipitate, and 5×10^{-5} M $Ru(bpy)_3(SbF_6)_2$ solution was obtained. Then the $Ru(bpy)_3(SbF_6)_2$ solution was degassed with a stream of N_2 for 30 minutes. The emission spectrum of a 5×10^{-5} M solution of $Ru(bpy)_3(SbF_6)_2$ in DCE was collected. Then, appropriate amount of quencher was added to the measured solution and the emission spectrum of the sample was collected.

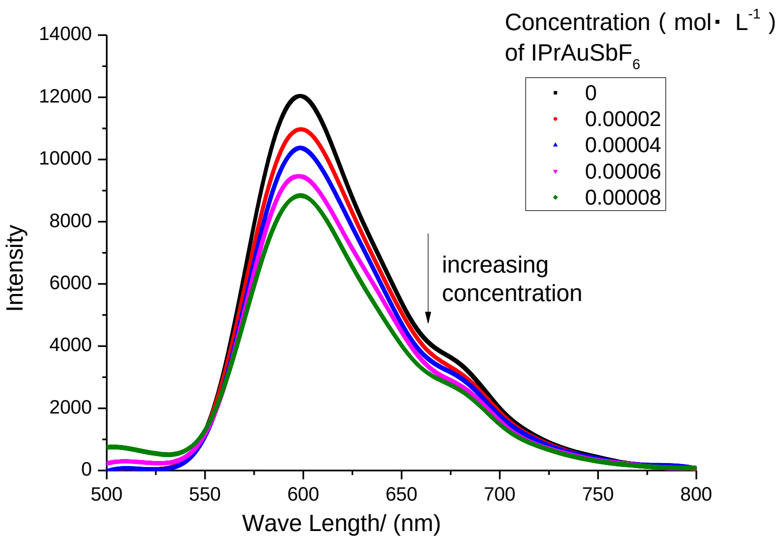

Figure S1. Ru(bpy)$_3$(SbF$_6$)$_2$ Emission Quenching by IPrAuSbF$_6$.

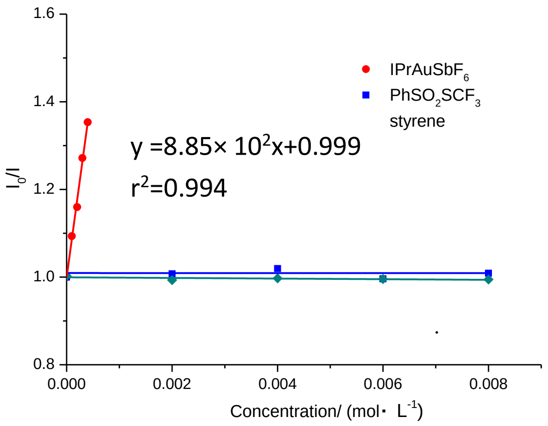

Figure S2. Ru(bpy)$_3$(SbF$_6$)$_2$ emission quenching with IPrAuSbF$_6$, PhSO$_2$SCF$_3$ and styrene; I_0 and I represent the intensities of the emission in the absence and presence of the quencher. Emission Quenching by IPrAuSbF$_6$, k_q = 8.85 × 10^2 mol^{-1}•L; no quenching were observed by PhSO$_2$SCF$_3$ and styrene.

Light/dark Experiments

The reaction was done on the condition: styrene **1a** (0.4mmol), $PhSO_2SCF_3$ **2a** (0.2mmol), IPrAuCl (10 mol％), $AgSbF_6$ (15 mol％), $Ru(bpy)_3Cl_2{\bullet}6H_2O$ (2.5 mol％), DCE 1ml at N_2 atmosphere. Yields of the **3** was determined by ^{19}F NMR and based on (trifluoromethyl)benzene as an internal standard.

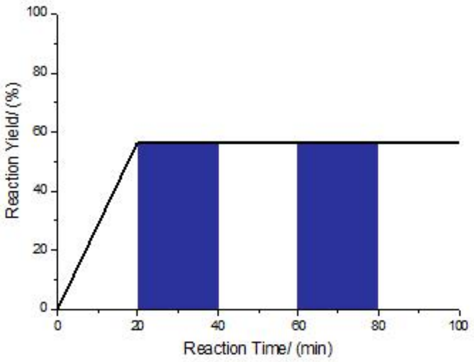

Figure S3. Light/dark experiments. The white area indicates the light irradiation, while the blue area indicates the dark treatment.

It was observed that the reaction completely ceased once the light source was removed. No more transformation was observed if irradiation was recommenced after a period of 20 minutes in the dark. This results may be caused by the altered reaction environment leading the aggregation of the active IPrAu(0) catalyst to inactive gold black.

Transient Absorption Experiments

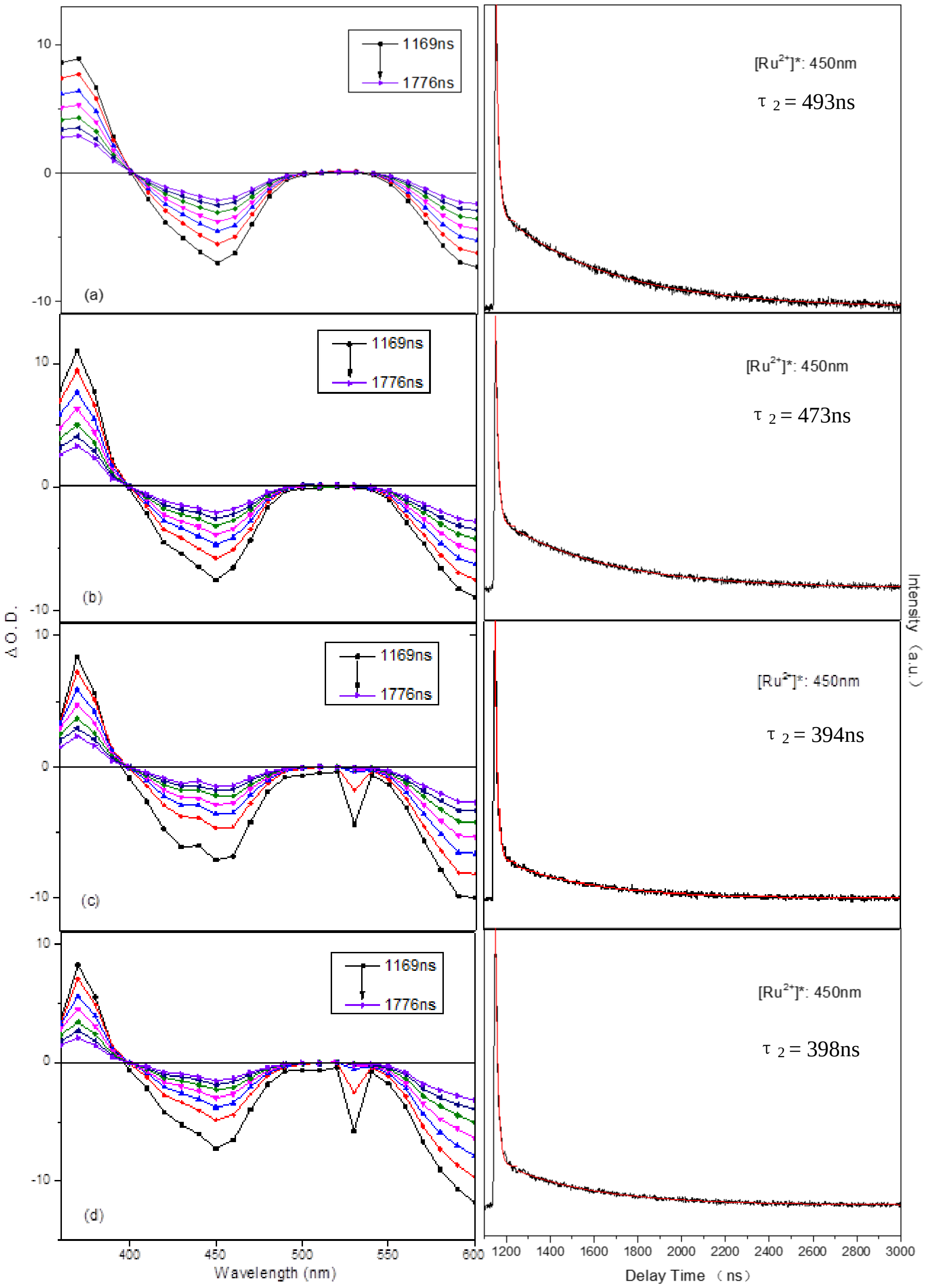

Figure S4. **Transient absorption spectra** (a) $Ru(bpy)_3Cl_2 \cdot 6H_2O = 8\times10^{-5}$ M, $AgSbF_6 = 1.6\times10^{-4}$ M, $\tau_1 = 13$ns, $\tau_2 = 493$ns ; (b) $Ru(bpy)_3Cl_2 \cdot 6H_2O = 8\times10^{-5}$ M, $AgSbF_6 = 1.6*10^{-4}$ M, $PhSO_2SCF_3 = 8\times10^{-3}$ M, $\tau_1 = 12$ns, $\tau_2 = 473$ns; (c) $Ru(bpy)_3Cl_2 \cdot 6H_2O = 8\times10^{-5}$ M, $AgSbF_6 = 1.6*10^{-4}$ M, $IPrAuSbF_6 = 1.5\times10^{-3}$, $\tau_1 = 12$ns, $\tau_2 = 394$ns; (d) $Ru(bpy)_3Cl_2 \cdot 6H_2O = 8\times10^{-5}$ M, $AgSbF_6 = 1.6*10^{-4}$ M, $IPrAuSbF_6 = 1.5\times10^{-3}$ M, $PhSO_2SCF_3 = 8\times10^{-3}$ M, $\tau_1 = 12$ns, $\tau_2 = 398$ns; in dce at room temrerature, excitation at 355nm.

CV Experiments

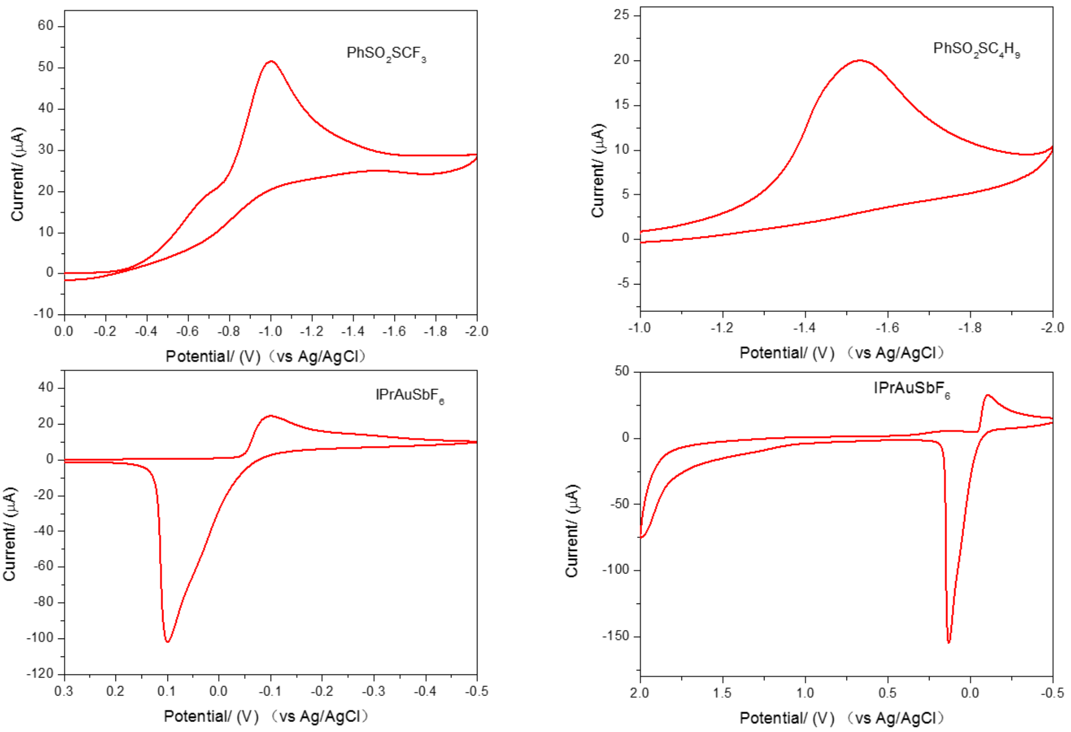

Figure S5. CV experiments condition: 10 mM sample in DCE, 0.1 M Bu_4NPF_6; scan rate, 100 mV s^{-1}. Potentials vs SCE results (Fc as a standard compound): $PhSO_2SCF_3$, Epa = −1.11 V; $PhSO_2SC_4H_9$, Epa = −1.64 V; $IPrAuSbF_6$, Epox =0.08 V, Epred -0.11 V.

X-ray Crystallography Data

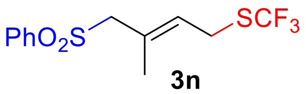 **3n**	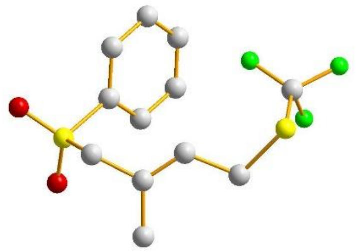
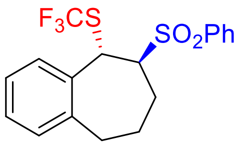 **3s**	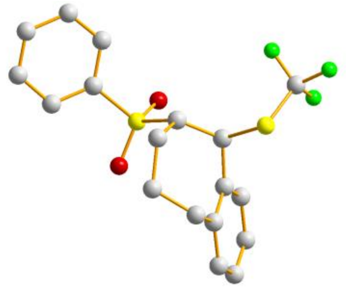

Characterization Data

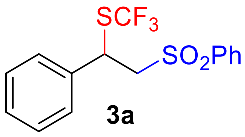

3a

Yield: 87%. ^{1}H NMR (400 MHz, $CDCl_3$) δ 7.61 (d, J = 7.3 Hz, 2H), 7.54-7.50 (m, 1H), 7.39-7.34 (m, 2H), 7.22-7.13 (m, 5H), 4.87 (dd, J = 10.2, 4.4 Hz, 1H), 3.99-3.90 (m, 1H), 3.85-3.79 (m, 1H). ^{13}C NMR (100 MHz, $CDCl_3$) δ 139.07, 135.94, 133.73, 129.62 (q, J = 307.1 Hz, SCF_3), 129.14, 129.06, 128.90, 127.88, 127.65, 61.24, 43.60. ^{19}F NMR (282 MHz, $CDCl_3$) δ -40.05. HRMS (ESI, m/z) calcd for $C_{15}H_{13}F_3O_2S_2$ $[M+NH_4]^+$ 364.0647, found 364.0630.

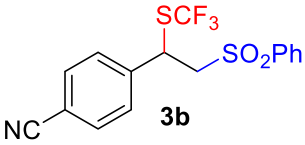

3b

Yield: 69%. ^{1}H NMR (400 MHz, $CDCl_3$) δ 7.66 (d, J = 7.3 Hz, 2H), 7.62-7.57 (m, 1H), 7.55-7.51 (m, 2H), 7.49-7.41 (m, 2H), 7.39-7.31 (m, 2H), 4.90 (dd, J = 10.0, 4.5 Hz, 1H), 3.93-3.82 (m, 1H), 3.81-3.73 (m, 1H). ^{13}C NMR (100 MHz, $CDCl_3$) δ 141.78, 138.82, 134.20, 132.73, 129.42, 129.31 (q, J = 307.1 Hz, SCF_3), 128.62, 127.85, 117.96, 112.84, 60.51, 42.92. ^{19}F NMR (282 MHz, $CDCl_3$) δ -39.26. HRMS (ESI, m/z) calcd for $C_{16}H_{12}F_3NO_2S_2$ $[M+H]^+$ 372.0334, found 372.0330.

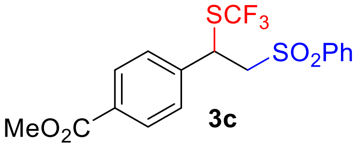

3c

Yield: 76%. ^{1}H NMR (400 MHz, $CDCl_3$) δ 7.87 (d, J = 8.4 Hz, 2H), 7.65-7.62 (m, 2H), 7.56-7.52 (m, 1H), 7.40-7.36 (m, 2H), 7.29-7.22 (m, 2H), 4.89 (dd, J = 10.0, 4.5 Hz, 1H), 3.94-3.86 (m, 4H), 3.83-3.76 (m, 1H). ^{13}C NMR (100 MHz, $CDCl_3$) δ 166.17, 141.17, 138.86, 133.97, 130.59, 130.26, 129.45 (q, J = 307.0 Hz, SCF_3), 129.25, 127.88, 127.78, 60.82, 52.32, 43.13. ^{19}F NMR (282 MHz, $CDCl_3$) δ -39.94. HRMS (ESI, m/z) calcd for $C_{17}H_{15}F_3O_4S_2$ $[M+H]^+$ 405.0437, found 405.0418.

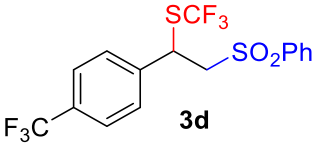

3d

Yield: 67%. ^{1}H NMR (400 MHz, $CDCl_3$) δ 7.63-7.49 (m, 3H), 7.48-7.41 (m, 2H), 7.41-7.34 (m, 2H), 7.32-7.27 (m, 2H), 4.92 (dd, J = 10.4, 4.4 Hz, 1H), 3.98-3.87 (m, 1H), 3.87-3.77 (m, 1H). ^{13}C NMR (100 MHz, $CDCl_3$) δ 140.11, 138.86, 133.95, 131.01 (q, J = 32.6 Hz), 129.40 (q, J = 307.0 Hz, SCF_3), 129.26, 128.22, 127.77, 125.98 (q, J = 3.8 Hz), 123.56 (q, J = 270.8 Hz, CF_3), 60.76, 42.99. ^{19}F NMR (282 MHz, $CDCl_3$) δ -39.89, -62.93. HRMS (ESI, m/z) calcd for $C_{16}H_{12}F_6O_2S_2$ $[M+Na]^+$ 437.0075, found 437.0075.

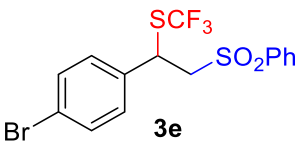

3e

Yield: 90%. ^{1}H NMR (400 MHz, $CDCl_3$) δ 7.65-7.53 (m, 3H), 7.47-7.37 (m, 2H), 7.34-7.28 (m, 2H), 7.09-7.00 (m, 2H), 4.83 (dd, J = 10.3, 4.4 Hz, 1H), 3.94-3.84 (m, 1H), 3.83-3.74 (m, 1H). ^{13}C NMR (100 MHz, $CDCl_3$) δ 138.99, 135.11, 133.79, 132.17, 129.45 (q, J = 307.1 Hz, SCF_3), 129.36, 129.25, 127.82, 123.04, 61.01, 42.98. ^{19}F NMR (282 MHz, $CDCl_3$) δ -39.89. HRMS (ESI, m/z) calcd for $C_{15}H_{12}BrF_3O_2S_2$ $[M+Na]^+$ 446.9306, found 446.9302.

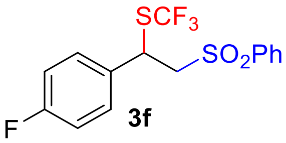

3f

Yield: 77%. ^{1}H NMR (400 MHz, $CDCl_3$) δ 7.62 (d, J = 7.3 Hz, 2H), 7.59-7.55 (m, 1H), 7.43-7.39 (m, 2H), 7.17-7.13 (m, 2H), 6.91-6.86 (m, 2H), 4.88 (dd, J = 10.3, 4.4 Hz, 1H), 3.92-3.86 (m, 1H), 3.82-3.77 (m, 1H). ^{13}C NMR (100 MHz, $CDCl_3$) δ 163.92, 161.44, 139.13, 133.82, 131.88, 129.58, 129.50, 129.49 (q, J = 306.9 Hz, SCF_3), 129.20, 128.06, 127.85, 116.17, 115.95, 61.25, 42.87. ^{19}F NMR (282 MHz, $CDCl_3$) δ -39.97, -112.02. HRMS (ESI, m/z) calcd for $C_{15}H_{12}F_4O_2S_2$ $[M+Na]^+$ 387.0107, found 387.0096.

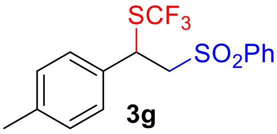

3g

Yield: 63%. ^{1}H NMR (400 MHz, $CDCl_3$) δ 7.61 (d, J = 7.5 Hz, 2H), 7.58-7.48 (m, 1H), 7.43-7.31 (m, 2H), 7.09-6.96 (m, 4H), 4.83 (dd, J = 10.3, 4.3 Hz, 1H), 3.97-3.76 (m, 2H), 2.28 (s, 3H). ^{13}C NMR (100 MHz, $CDCl_3$) δ 139.16, 138.86, 133.52, 132.79, 129.69, 129.65 (q, J = 306.9 Hz, SCF_3) 129.06, 127.91, 127.55, 61.40, 43.37, 21.11. ^{19}F NMR (282 MHz, $CDCl_3$) δ -40.06. HRMS (ESI, m/z) calcd for $C_{16}H_{15}F_3O_2S_2$ $[M+Na]^+$ 383.0358, found 383.0360.

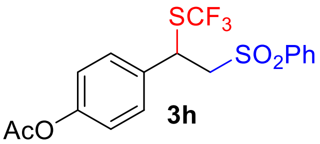

3h

Yield: 70%. ^{1}H NMR (400 MHz, $CDCl_3$) δ 7.67-7.48 (m, 3H), 7.44-7.34 (m, 2H), 7.15 (d, J = 8.5 Hz, 2H), 6.92 (d, J = 8.5 Hz, 2H), 4.88 (dd, J = 10.0, 4.5 Hz, 1H), 3.97-3.85 (m, 1H), 3.85-3.76 (m, 1H), 2.28 (s, 3H). ^{13}C NMR (100 MHz, $CDCl_3$) δ 168.89, 150.88, 138.93, 133.88, 133.36, 129.53 (q, J = 306.9 Hz, SCF_3), 129.28, 128.78, 127.80, 122.22, 61.29, 43.04, 21.12. ^{19}F NMR (282 MHz, $CDCl_3$) δ -39.97. HRMS (ESI, m/z) calcd for $C_{17}H_{15}F_3O_4S_2$ $[M+Na]^+$ 427.0256, found 427.0257.

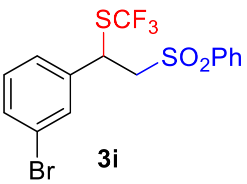

3i

Yield: 69%. ^{1}H NMR (400 MHz, $CDCl_3$) δ 7.62 (d, *J* = 7.8 Hz, 2H), 7.58-7.53 (m, 1H), 7.42-7.38 (m, 2H), 7.34-7.31 (m, 1H), 7.23 (s, 1H), 7.15-7.06 (m, 2H), 4.81 (dd, *J* = 10.2, 4.4 Hz, 1H), 3.92-3.85 (m, 1H), 3.80-3.76 (m, 1H). ^{13}C NMR (100 MHz, $CDCl_3$) δ 138.80, 138.22, 132.06, 130.66, 130.56, 129.44 (q, *J* = 307.0 Hz, SCF_3), 129.18, 128.03, 127.80, 126.47, 122.96, 60.84, 42.96.^{19}F NMR (282 MHz, $CDCl_3$) δ -39.94. HRMS (ESI, m/z) calcd for $C_{15}H_{12}BrF_3O_2S_2$ $[M+Na]^+$ 446.9306, found 446.9301.

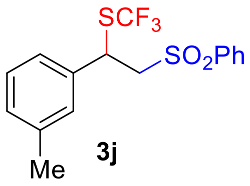

3j

Yield: 86%. ^{1}H NMR (400 MHz, $CDCl_3$) δ 7.59 (d, *J* = 8.0 Hz, 2H), 7.54-7.47 (m, 1H), 7.40-7.32 (m, 2H), 7.10-7.05 (m, 1H), 7.03-6.95 (m, 2H), 6.88 (s, 1H), 4.83 (dd, *J* = 10.3, 4.2 Hz, 1H), 4.06-3.89 (m, 1H), 3.87-3.77 (m, 1H), 2.20 (s, 3H). ^{13}C NMR (100 MHz, $CDCl_3$) δ 139.12, 138.83, 135.59, 133.61, 129.69, 129.65 (q, *J* = 306.9 Hz, SCF_3), 129.05, 128.97, 128.15, 127.85, 124.84, 61.27, 43.60, 21.22. ^{19}F NMR (282 MHz, $CDCl_3$) δ -40.08. HRMS (ESI, m/z) calcd for $C_{16}H_{15}F_3O_2S_2$ $[M+Na]^+$ 383.0358, found 383.0353.

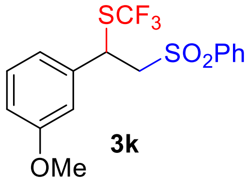

3k

Yield: 76%. ^{1}H NMR (400 MHz, $CDCl_3$) δ 7.62 (d, *J* = 7.3 Hz, 2H), 7.54-7.50 (m, 1H), 7.39-7.35 (m, 2H), 7.13-7.09 (m, 1H), 6.76-6.71 (m, 2H), 6.63-6.61 (m, 1H), 4.82 (dd, *J* = 10.2, 4.4 Hz, 1H), 3.98-3.90 (m, 1H), 3.83-3.76 (m, 1H), 3.71 (s, 3H). ^{13}C NMR (100 MHz, $CDCl_3$) δ 159.86, 139.08, 137.27, 133.66, 130.16, 129.61 (q, *J* = 306.8 Hz, SCF_3), 129.06, 127.88, 119.92, 114.30, 113.26, 61.24, 55.22, 43.54. ^{19}F NMR (282 MHz, $CDCl_3$) δ -40.01. HRMS (ESI, m/z) calcd for $C_{16}H_{15}F_3O_3S_2$ $[M+Na]^+$ 399.0307, found 399.0299.

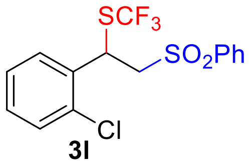

3l

Yield: 72%. ^{1}H NMR (400 MHz, $CDCl_3$) δ 7.69 (d, *J* = 7.5 Hz, 2H), 7.61-7.51 (m, 1H), 7.48-7.38 (m, 2H), 7.27-7.23 (m, 2H), 7.21-7.12 (m, 2H), 5.28-5.08 (m, 1H), 4.28-4.08 (m, 1H), 3.88-3.78 (m, 1H). ^{13}C NMR (100 MHz, $CDCl_3$) δ 138.57, 133.84, 133.66, 133.25, 130.42, 130.06, 129.65 (q, *J* = 306.9 Hz, SCF_3), 129.17, 128.25, 127.91, 127.33, 62.68, 60.02. ^{19}F NMR (282 MHz, $CDCl_3$) δ -40.72. HRMS (ESI, m/z) calcd for $C_{15}H_{12}ClF_3O_2S_2$ $[M+Na]^+$ 402.9812, found 402.9800.

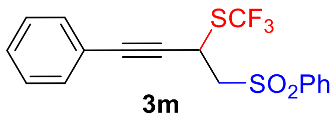

3m

Yield: 31%. ^{1}H NMR (400 MHz, $CDCl_3$) δ 7.98 (d, *J* = 7.2 Hz, 2H), 7.64-7.55 (m, 1H), 7.54-7.48 m, 2H), 7.34-7.26 (m, 3H), 7.23-7.17 (m, 2H), 4.68 (dd, *J* = 9.9, 3.8 Hz, 1H), 3.87-3.78 (m, 1H), 3.74-3.65 (m, 1H). ^{13}C NMR (100 MHz, $CDCl_3$) δ 138.94, 134.21, 132.78 (q, *J* = 306.9 Hz, SCF_3), 131.77, 129.36, 129.13, 128.51, 128.19, 121.28, 87.89, 82.54, 61.18, 30.50, 29.69. ^{19}F NMR (282 MHz, $CDCl_3$) δ -39.43. HRMS (ESI, m/z) calcd for $C_{17}H_{13}F_3O_2S_2$ $[M+Na]^+$ 393.0201, found 393.0200.

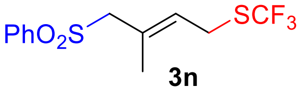

3n

Yield: 55%. ^{1}H NMR (400 MHz, $CDCl_3$) δ 7.85 (d, *J* = 7.3 Hz, 2H), 7.72-7.62 (m, 1H), 7.61-7.53 (m, 2H), 5.20 (t, *J* = 7.8 Hz, 1H), 3.76 (s, 2H), 3.46 (d, *J* = 7.9 Hz, 2H), 1.85 (s, 3H). ^{13}C NMR (100 MHz, $CDCl_3$) δ 138.16, 133.88, 130.60 (q, *J* = 306.8 Hz, SCF_3), 129.53, 129.19, 128.44, 128.28, 65.62, 27.36, 16.83. ^{19}F NMR (282 MHz, $CDCl_3$) δ -41.43. HRMS (ESI, m/z) calcd for $C_{12}H_{13}F_3O_2S_2$ $[M+H]^+$ 311.0382, found 311.0394.

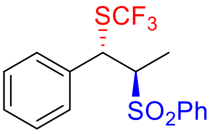

Yield: 67% / 61% (from (*E*)-alkene / from (*Z*)-alkene), d.r.=9:1 / d.r.=9:1. ^{1}H NMR (400 MHz, $CDCl_3$) δ 7.86 (d, *J* = 8.0 Hz, 2H), 7.70-7.62 (m, 1H), 7.58-7.49 (m, 2H), 7.35-7.27 (m, 5H), 5.04 (d, *J* = 3.8 Hz, 1H), 3.48 (m, 1H), 1.43 (d, *J* = 7.1 Hz, 3H). ^{13}C NMR (100 MHz, $CDCl_3$) δ 138.75, 137.65, 134.01, 130.00 (q, *J* = 306.2 Hz, SCF_3), 129.24, 128.99, 128.86, 128.38, 127.78, 65.31, 48.13, 10.28. ^{19}F NMR (282 MHz, $CDCl_3$) δ -40.03. HRMS (ESI, m/z) calcd for $C_{16}H_{15}F_3O_2S_2$ $[M+Na]^+$ 383.0358, found 383.0357.

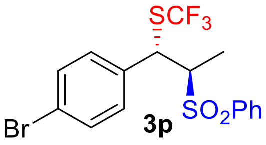

3p

Yield: 65%, d.r.=7:1. ^{1}H NMR (400 MHz, $CDCl_3$) δ 7.79 (d, *J* = 7.8 Hz, 2H), 7.68-7.59 (m, 1H), 7.57-7.48 (m, 2H), 7.43-7.36 (d, *J* = 8.5 Hz, 2H), 7.18 (d, *J* = 8.4 Hz, 2H), 4.94 (d, *J* = 4.6 Hz, 1H), 3.51 – 3.39 (m, 1H), 1.44 (d, *J* = 7.1 Hz, 3H). ^{13}C NMR (100 MHz, $CDCl_3$) δ 137.70, 133.99, 131.96, 130.03, 129.60, 129.85 (q, *J* = 305.9 Hz, SCF_3), 129.28, 128.81, 122.52, 64.94, 48.01, 10.79. ^{19}F NMR (282 MHz, $CDCl_3$) δ -40.01. HRMS (ESI, m/z) calcd for $C_{16}H_{14}BrF_3O_2S_2$ $[M+Na]^+$ 460.9463, found 460.9468.

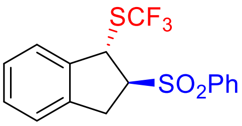

3q

Yield: 63%, d.r.>20:1. ^{1}H NMR (400 MHz, $CDCl_3$) δ 7.89 (d, *J* = 7.4 Hz, 2H), 7.70-7.59 (m, 1H), 7.57-7.48 (m, 2H), 7.38-7.14 (m, 4H), 5.33-5.21 (m, 1H), 4.24-4.14 (m, 1H), 3.64-3.45 (m, 2H). ^{13}C NMR (100 MHz, $CDCl_3$) δ 140.20, 137.15, 137.11, 134.21, 129.65 (q, *J* = 307.2 Hz, SCF_3), 129.55, 129.35, 128.87, 128.00, 125.51, 124.70, 70.34, 49.24, 31.86. ^{19}F NMR (282 MHz, $CDCl_3$) δ -40.14. HRMS (ESI, m/z) calcd for

$C_{16}H_{13}F_3O_2S_2$ [M+Na]$^+$ 381.0201, found 381.0206.

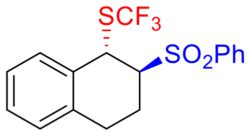

3r

Yield: 56%, d.r.=10:1. ^{1}H NMR (400 MHz, $CDCl_3$) δ 7.88 (d, *J* = 7.7 Hz, 2H), 7.73-7.63 (m, 1H), 7.64-7.53 (m, 2H), 7.38-7.32 (m, 1H), 7.24-7.17 (m, 2H), 7.13-7.04 (m, 1H), 5.08-4.95 (m, 1H), 3.90-3.79 (m, 1H), 3.27-3.14 (m, 1H), 2.90-2.79 (m, 1H), 2.61-2.42 (m, 2H). ^{13}C NMR (100 MHz, $CDCl_3$) δ 137.93, 136.65, 134.14, 130.74, 129.76 (q, *J* = 307.1 Hz, SCF_3), 129.41, 129.28, 129.15, 128.59, 128.57, 126.82, 64.58, 42.07, 24.37, 18.07. ^{19}F NMR (282 MHz, $CDCl_3$) δ -41.05. HRMS (ESI, m/z) calcd for $C_{17}H_{15}F_3O_2S_2$ [M+Na]$^+$ 395.0358, found 395.0367.

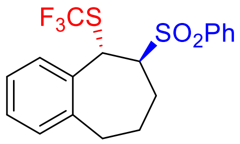

3s

Yield: 52%, d.r.=9:1. ^{1}H NMR (400 MHz, $CDCl_3$) δ 7.80 (d, *J* = 7.5 Hz, 2H), 7.72-7.64 (m, 1H), 7.58-7.51 (m, 2H), 7.26-7.17 (m, 3H), 7.12-7.05 (m, 1H), 5.13 (d, *J* = 4.3 Hz, 1H), 3.60-3.52 (m, 1H), 3.07-2.94 (m, 1H), 2.80-2.70 (m, 1H), 2.43-2.27 (m, 1H), 2.15-2.05 (m, 1H), 1.92-1.75 (m, 2H). ^{13}C NMR (100 MHz, $CDCl_3$) δ 140.90, 137.95, 134.87, 134.02, 130.75, 130.34, 130.12 (q, *J* = 306.1 Hz, SCF_3), 129.36, 129.30, 128.86, 126.85, 66.28, 48.36, 33.94, 25.02, 22.75. ^{19}F NMR (282 MHz, $CDCl_3$) δ -40.58. HRMS (ESI, m/z) calcd for $C_{18}H_{17}F_3O_2S_2$ [M+Na]$^+$ 409.0514, found 409.0514.

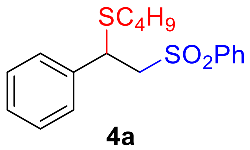

4a

Yield: 84%. ^{1}H NMR (400 MHz, $CDCl_3$) δ 7.63 (d, *J* = 7.6 Hz, 2H), 7.51-7.47 (m, 1H), 7.37-7.33 (m, 2H), 7.14-7.12 (m, 5H), 4.32 (dd, *J* = 9.4, 4.6 Hz, 1H), 3.80-3.68 (m, 1H), 3.68-3.63 (m, 1H), 2.37-2.24 (m, 2H), 1.49-1.38 (m, 2H), 1.31-1.23 (m, 2H), 0.83 (t, *J* = 7.3 Hz, 3H). ^{13}C NMR (100 MHz, $CDCl_3$) δ 139.56, 138.95, 133.37, 128.95, 128.59, 127.91, 127.81, 127.79, 61.58, 43.30, 31.23, 31.04, 21.87, 13.58. HRMS (ESI, m/z) calcd for $C_{18}H_{22}O_2S_2$ [M+H]$^+$ 335.1134, found 335.1136.

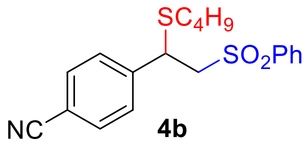

4b

Yield: 71%. ^{1}H NMR (400 MHz, $CDCl_3$) δ 7.64 (d, J = 7.3 Hz, 2H), 7.58-7.54 (m, 1H), 7.48-7.46 (m, 2H), 7.42-7.38 (m, 2H), 7.30-7.28 (m, 2H), 4.35 (dd, *J* = 9.7, 4.5Hz, 1H), 3.74-3.62 (m, 2H), 2.36-2.24 (m, 2H), 1.46-1.41 (m, 2H), 1.29-1.24 (m, 2H), 0.83 (t, *J* = 7.3 Hz, 3H). ^{13}C NMR (100 MHz, $CDCl_3$) δ 144.72, 139.32, 133.78, 132.34, 129.19, 128.71, 127.84, 118.37, 111.63, 60.98, 43.00, 31.36, 30.90, 21.80, 13.53. HRMS (ESI,

m/z) calcd for $C_{19}H_{21}NO_2S_2$ $[M+H]^+$ 360.1086, found 360.1081.

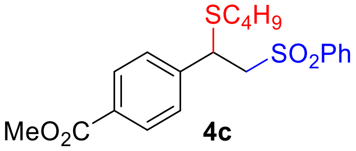

4c

Yield: 40%. ^{1}H NMR (400 MHz, $CDCl_3$) δ 7.83 (d, J = 8.2 Hz, 2H), 7.63 (d, J = 7.6 Hz, 2H), 7.52-7.48 (m, 1H), 7.37-7.33 (m, 2H), 7.23-7.21 (m, 2H), 4.35 (dd, *J* = 9.6, 4.4 Hz, 1H), 3.90 (s, 3H), 3.78-3.63 (m, 2H), 2.35-2.23 (m, 2H), 1.45-1.41 (m, 2H), 1.29-1.25 (m, 2H), 0.81 (t, *J* = 7.4 Hz, 3H). ^{13}C NMR (100 MHz, $CDCl_3$) δ 166.51, 144.27, 139.35, 133.58, 129.87, 129.55, 129.04, 127.90, 127.89, 61.20, 52.18, 43.01, 31.25, 30.95, 21.83, 13.55. HRMS (ESI, m/z) calcd for $C_{20}H_{24}O_4S_2$ $[M+Na]^+$ 415.1008, found 415.0999.

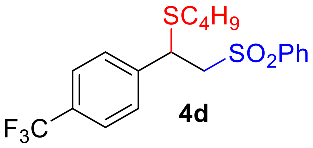

4d

Yield: 48%. ^{1}H NMR (300 MHz, $CDCl_3$) δ 7.58 (d, *J* = 7.2 Hz, 2H), 7.54-7.45 (m, 1H), 7.44-7.31 (m, 4H), 7.29-7.21 (m, 2H), 4.37 (dd, *J* = 9.7, 4.6 Hz, 1H), 3.85-3.62 (m, 2H), 2.41-2.24 (m, 2H), 1.51-1.41 (m, 2H), 1.35-1.25 (m, 2H), 0.84 (t, *J* = 7.3 Hz, 3H). ^{13}C NMR (75 MHz, $CDCl_3$) δ 143.01, 139.34, 133.54, 129.02, 128.26, 127.76, 125.52, 125.47, 122.01, 61.14, 42.97, 31.30, 30.95, 21.82, 13.53. ^{19}F NMR (282 MHz, $CDCl_3$) δ -62.73. HRMS (ESI, m/z) calcd for $C_{19}H_{21}F_3O_2S_2$ $[M+H]^+$ 403.1008, found 403.1003.

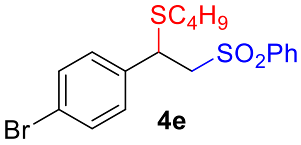

4e

Yield: 66%. ^{1}H NMR (400 MHz, $CDCl_3$) δ 7.60 (d, *J* = 7.3 Hz, 2H), 7.56-7.52 (m, 1H), 7.39-7.35 (m, 2H), 7.26-7.24 (m, 2H), 7.02-7.00 (m, 2H), 4.28 (dd, *J* = 9.7, 4.6 Hz, 1H), 3.73-3.61 (m, 2H), 2.36-2.23 (m, 2H), 1.49-1.40 (m, 2H), 1.34-1.22 (m, 2H), 0.83 (t, *J* = 7.3 Hz, 3H). ^{13}C NMR (100 MHz, $CDCl_3$) δ 139.44, 138.01, 133.42, 131.66, 129.56, 129.05, 127.83, 121.68, 61.38, 42.76, 31.22, 30.99, 21.85, 13.58. HRMS (ESI, m/z) calcd for $C_{18}H_{21}BrO_2S_2$ $[M+H]^+$ 413.0239, found 413.0236.

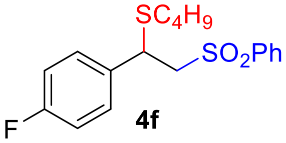

4f

Yield: 68%. ^{1}H NMR (400 MHz, $CDCl_3$) δ 7.62 (d, *J* = 7.4 Hz, 2H), 7.55-7.51 (m, 1H), 7.40-7.35 (m, 2H), 7.14-7.09 (m, 2H), 6.86-6.81 (m, 2H), 4.32 (dd, *J* = 9.7, 4.5 Hz, 1H), 3.75-3.61 (m, 2H), 2.38-2.23 (m, 2H), 1.50-1.39 (m, 2H), 1.33-1.23 (m, 2H), 0.83 (t, *J* = 7.3 Hz, 3H). ^{13}C NMR (100 MHz, $CDCl_3$) δ 163.28, 160.83, 139.51, 134.71, 133.48, 129.54, 129.46, 129.01, 127.85, 115.60, 115.38, 61.55, 42.58, 31.21, 31.00, 21.87, 13.59. ^{19}F NMR (282 MHz, $CDCl_3$) δ -114.10. HRMS (ESI, m/z) calcd for $C_{18}H_{21}FO_2S_2$ $[M+H]^+$ 353.1040, found 353.1044.

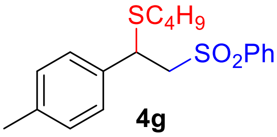

4g

Yield: 60%. ^{1}H NMR (400 MHz, $CDCl_3$) δ 7.67-7.58 (m, 2H), 7.56-7.46 (m, 1H), 7.40-7.29 (m, 2H), 7.03 (d, *J* = 8.1 Hz, 2H), 6.95 (d, *J* = 8.0 Hz, 2H), 4.30 (dd, *J* = 9.5, 4.6 Hz, 1H), 3.80-3.60 (m, 2H), 2.37-2.30 (m, 2H), 2.26 (s, 3H), 1.50-1.40 (m, 2H), 1.32-1.26 (m, 2H), 0.84 (t, *J* = 7.3 Hz, 3H).^{13}C NMR (100 MHz, $CDCl_3$) δ 139.65, 137.48, 135.85, 133.18, 129.24, 128.88, 127.94, 127.69, 61.72, 42.99, 31.19, 31.08, 21.89, 21.07, 13.60. HRMS (ESI, m/z) calcd for $C_{19}H_{24}O_2S_2$ $[M+Na]^+$ 371.1110, found 371.1115.

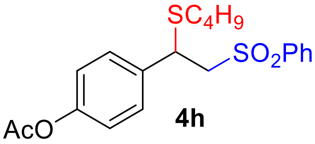

4h

Yield: 52%. ^{1}H NMR (400 MHz, $CDCl_3$) δ 7.61 (d, *J* = 7.3 Hz, 2H), 7.53-7.49 (m, 1H), 7.39-7.35 (m, 2H), 7.14-7.11 (m, 2H), 6.88-6.85 (m, 2H), 4.33 (dd, *J* = 9.3, 4.8 Hz, 1H), 3.76-3.62 (m, 2H), 2.34-2.28 (m, 2H), 2.27 (s, 3H), 1.47-1.42 (m, 2H), 1.33-1.25 (m, 2H), 0.83 (t, *J* = 7.3 Hz, 3H). ^{13}C NMR (100 MHz, $CDCl_3$) δ 169.08, 150.05, 139.41, 136.46, 133.53, 129.08, 128.80, 127.83, 121.69, 61.66, 42.78, 31.27, 31.00, 21.87, 21.16, 13.59. HRMS (ESI, m/z) calcd for $C_{20}H_{24}O_4S_2$ $[M+NH_4]^+$ 410.1454, found 410.1458.

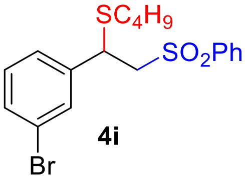

4i

Yield: 57%. ^{1}H NMR (300 MHz, $CDCl_3$) δ 7.67-7.59 (m, 2H), 7.57-7.49 (m, 1H), 7.45-7.33 (m, 2H), 7.29-7.18 (m, 2H), 7.15-6.99 (m, 2H), 4.26 (dd, *J* = 9.5, 4.7 Hz, 1H), 3.79-3.57 (m, 2H), 2.41-2.24 (m, 2H), 1.53-1.39 (m, 2H), 1.39-1.23 (m, 2H), 0.84 (t, *J* = 7.3 Hz, 3H). ^{13}C NMR (75 MHz, $CDCl_3$) δ 141.26, 139.27, 133.63, 130.92, 130.80, 130.07, 128.96, 127.81, 126.60, 122.61, 61.19, 42.90, 31.31, 30.96, 21.83, 13.55. HRMS (ESI, m/z) calcd for $C_{18}H_{21}BrO_2S_2$ $[M+H]^+$ 413.0239, found 413.0248.

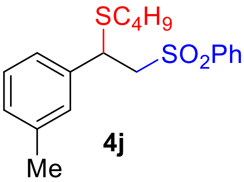

4j

Yield: 70%. ^{1}H NMR (300 MHz, $CDCl_3$) δ 7.66-7.55 (m, 2H), 7.51-7.44 (m, 1H), 7.38-7.28 (m, 2H), 7.03 (d, *J* = 7.5 Hz, 1H), 6.97-6.89 (m, 2H), 6.86 (s, 1H), 4.27 (dd, *J* = 9.4, 4.7 Hz, 1H), 3.84-3.71 (m, 1H), 3.69-3.58 (m, 1H), 2.35-2.28 (m, 2H), 2.18 (s, 3H), 1.52-1.39 (m, 2H), 1.33-1.24 (m, 2H), 0.84 (t, *J* = 7.3 Hz, 3H). ^{13}C NMR (75 MHz, $CDCl_3$) δ 139.56, 138.68, 138.18, 133.27, 128.77, 128.57, 128.44, 128.28, 127.88, 125.01, 61.51, 43.29, 31.26, 31.06, 21.87, 21.26, 13.58. HRMS (ESI, m/z) calcd for

$C_{19}H_{24}O_2S_2$ [M+H]$^+$ 349.1290, found 349.1296.

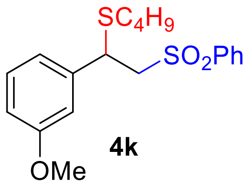

4k

Yield: 41%. ^{1}H NMR (400 MHz, $CDCl_3$) δ 7.64 (d, J = 7.4 Hz, 2H), 7.52-7.48 (m, 1H), 7.38-7.34 (m, 2H), 7.09-7.05 (m, 1H), 6.74 (d, J = 7.6 Hz, 1H), 6.67 (d, J = 8.3 Hz, 1H), 6.62 (s, 1H), 4.28 (dd, J = 9.4, 4.7 Hz, 1H), 3.78-3.71 (m, 1H), 3.70 (s, 3H), 3.69-3.61 (m, 1H), 2.42-2.23 (m, 2H), 1.48-1.40 (m, 2H), 1.36-1.26 (m, 2H), 0.84 (t, J = 7.3 Hz, 3H). ^{13}C NMR (100 MHz, $CDCl_3$) δ 159.66, 140.50, 139.62, 133.30, 129.57, 128.86, 127.92, 120.32, 113.35, 113.22, 61.60, 55.14, 43.38, 31.29, 31.05, 21.86, 13.56. HRMS (ESI, m/z) calcd for $C_{19}H_{24}O_3S_2$ [M+H]$^+$ 365.1240, found 365.1242.

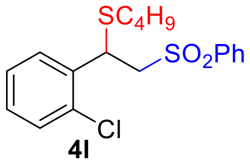

4l

Yield: 71%. ^{1}H NMR (400 MHz, $CDCl_3$) δ 7.73 (d, J = 7.9 Hz, 2H), 7.55-7.48 (m, 1H), 7.43-7.35 (m, 2H), 7.24-7.20 (m, 2H), 7.10-7.05 (m, 2H), 4.88-4.76 (m, 1H), 3.94-3.81 (m, 1H), 3.69-3.64 (m, 1H), 2.47-2.32 (m, 2H), 1.53-1.41 (m, 2H), 1.33-1.26 (m, 2H), 0.84 (t, J = 7.3 Hz, 3H). ^{13}C NMR (100 MHz, $CDCl_3$) δ 139.01, 136.63, 133.57, 133.49, 129.82, 129.05, 129.01, 128.80, 128.01, 127.19, 60.62, 52.55, 31.54, 31.20, 21.87, 13.57. HRMS (ESI, m/z) calcd for $C_{18}H_{21}ClO_2S_2$ [M+H]$^+$ 369.0744, found 369.0744.

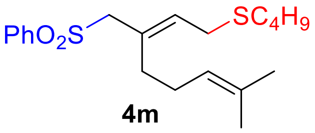

4m

Yield: 51%. ^{1}H NMR (400 MHz, $CDCl_3$) δ 7.85 (d, J = 7.3 Hz, 2H), 7.69-7.59 (m, 1H), 7.58-7.50 (m, 2H), 5.23 (t, J = 7.8 Hz, 1H), 5.04 (t, J = 7.1 Hz, 1H), 3.77 (s, 2H), 3.06 (d, J = 7.8 Hz, 2H), 2.41-2.31 (m, 2H), 2.28-2.13 (m, 2H), 2.12-2.01 (m, 2H), 1.66 (s, 3H), 1.58 (s, 3H), 1.55-1.44 (m, 2H), 1.43-1.31 (m, 2H), 0.90 (t, J = 7.2 Hz, 3H). ^{13}C NMR (100 MHz, $CDCl_3$) δ 138.59, 133.60, 132.79, 132.70, 130.12, 129.07, 128.50, 122.97, 63.14, 31.62, 31.35, 29.75, 29.10, 26.65, 25.65, 22.00, 17.73, 13.69. HRMS (ESI, m/z) calcd for $C_{20}H_{30}O_2S_2$ [M+H]$^+$ 367.1760, found 367.1754.

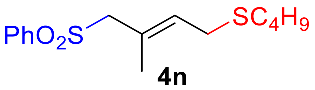

4n

Yield: 55%. ^{1}H NMR (400 MHz, $CDCl_3$) δ 7.86 (d, J = 7.2 Hz, 2H), 7.66-7.61 (m, 1H), 7.56-7.52 (m, 2H), 5.20 (t, J = 7.7 Hz, 1H), 3.76 (s, 2H), 3.04 (d, J = 7.5 Hz, 2H), 2.39 -2.33 (m, 2H), 1.81 (s, 3H), 1.51-1.44 (m, 2H), 1.38-1.34 (m, 2H), 0.89 (t, J = 7.3 Hz, 3H). ^{13}C NMR (100 MHz, $CDCl_3$) δ 138.46, 133.68, 132.19, 129.09, 128.43, 126.01, 65.91, 31.58, 31.07, 29.13, 22.04, 16.85, 13.71. HRMS (ESI, m/z) calcd for $C_{15}H_{22}O_2S_2$ [M+H]$^+$ 299.1134, found 299.1132.

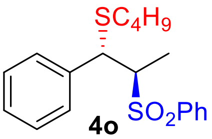

4o

Yield: 45% / 48% (from (*E*)-alkene / from (*Z*)-alkene), d.r.>20:1 / d.r.>20:1. ^{1}H NMR (400 MHz, $CDCl_3$) δ 7.81 (d, *J* = 7.3 Hz, 2H), 7.63-7.55 (m, 1H), 7.51-7.43 (m, 2H), 7.36-7.29 (m, 2H), 7.28 -7.17 (m, 3H), 4.55 (d, *J* = 4.6 Hz, 1H), 3.52-3.41 (m, 1H), 2.26 (m, 2H), 1.53-1.38 (m, 5H), 1.31-123 (m, 2H), 0.82 (t, *J* = 7.3 Hz, 3H). ^{13}C NMR (100 MHz, $CDCl_3$) δ 140.15, 138.58, 133.40, 129.78, 128.87, 128.58, 128.38, 127.59, 66.06, 48.84, 31.42, 31.00, 21.89, 13.59, 10.50. HRMS (ESI, m/z) calcd for $C_{19}H_{24}O_2S_2$ $[M+Na]^+$ 371.1110, found 371.1110.

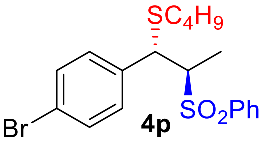

4p

Yield: 51%, d.r.=12:1. ^{1}H NMR (400 MHz, $CDCl_3$) δ 7.72 (d, *J* = 8.0 Hz, 2H), 7.62-7.55 (m, 1H), 7.47-7.41 (m, 2H), 7.32 (d, *J* = 8.4 Hz, 2H), 7.16 (d, *J* = 8.4 Hz, 2H), 4.43 (d, *J* = 5.7 Hz, 1H), 3.50-3.39 (m, 1H), 2.32-2.17 (m, 2H), 1.48 (d, *J* = 7.1 Hz, 3H), 1.45-1.38 (m, 2H), 1.30-1.22 (m, 2H), 0.82 (t, *J* = 7.3 Hz, 3H). ^{13}C NMR (100 MHz, $CDCl_3$) δ 139.18, 138.62, 133.34, 131.61, 130.14, 128.95, 128.65, 121.50, 65.71, 48.65, 31.36, 30.95, 21.87, 13.58, 10.97. HRMS (ESI, m/z) calcd for $C_{19}H_{23}BrO_2S_2$ $[M+H]^+$ 427.0396, found 427.0392.

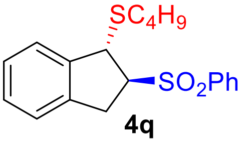

4q

Yield: 57%, d.r.>20:1. ^{1}H NMR (400 MHz, $CDCl_3$) δ 7.89 (d, *J* = 7.8 Hz, 2H), 7.63-7.58 (m, 1H), 7.53-7.49 (m, 2H), 7.29-7.26 (m, 1H), 7.18-7.08 (m, 3H), 4.78 (d, *J* = 2.9 Hz, 1H), 4.02 -3.96 (m, 1H), 3.46-3.44 (m, 2H), 2.52-2.34 (m, 2H), 1.55-1.43 (m, 2H), 1.37-1.27 (m, 2H), 0.88 (t, *J* = 7.4 Hz, 3H). ^{13}C NMR (100 MHz, $CDCl_3$) δ 140.51, 139.36, 137.59, 133.88, 129.18, 128.85, 128.34, 127.50, 125.14, 124.31, 71.18, 49.80, 32.49, 31.36, 31.16, 22.01, 13.65. HRMS (ESI, m/z) calcd for $C_{19}H_{22}O_2S_2$ $[M+H]^+$ 347.1134, found 347.1135.

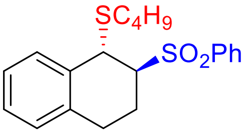

4r

Yield: 56%, d.r.=14:1. ^{1}H NMR (400 MHz, $CDCl_3$) δ 7.87 (d, *J* = 7.6 Hz, 2H), 7.68-7.60 (m, 1H), 7.57-7.46 m, 2H), 7.28-7.22 (m, 1H), 7.18-7.07 (m, 2H), 7.02 (d, *J* = 6.7 Hz, 1H), 4.52-4.41 (m, 1H), 3.68-3.62 (m, 1H), 3.05-2.94 (m, 1H), 2.90-2.76 (m, 1H), 2.55-2.42 (m, 1H), 2.37-2.16 (m, 3H), 1.48-1.36 (m, 2H), 1.31-1.23 (m, 2H), 0.85 (t, *J* = 7.2 Hz, 3H). ^{13}C NMR (100 MHz, $CDCl_3$) δ 138.09, 136.35, 133.80, 133.36, 130.04,

129.20, 128.76, 128.54, 127.35, 126.28, 64.97, 41.13, 31.89, 31.20, 25.19, 21.96, 19.71, 13.62. HRMS (ESI, m/z) calcd for $C_{20}H_{24}O_2S_2$ $[M+Na]^+$ 383.1110, found 383.1122.

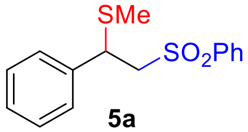

5a

Yield: 90%. ^{1}H NMR (400 MHz, $CDCl_3$) δ 7.64 (d, J = 7.3 Hz, 2H), 7.52-7.48 (m, 1H), 7.38-7.33 (m, 2H), 7.18-7.11 (m, 5H), 4.24 (dd, J = 9.3, 4.8 Hz, 1H), 3.81-3.65 (m, 2H), 1.91 (s, 3H). ^{13}C NMR (100 MHz, $CDCl_3$) δ 139.48, 138.43, 133.46, 129.00, 128.63, 127.92, 127.88, 127.82, 61.17, 44.98, 14.87. HRMS (ESI, m/z) calcd for $C_{15}H_{16}O_2S_2$ $[M+H]^+$ 293.0664, found 293.0677.

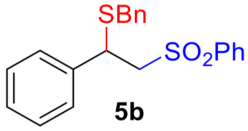

5b

Yield: 66%. ^{1}H NMR (400 MHz, $CDCl_3$) δ 7.59 (d, J = 8.0 Hz, 2H), 7.53-7.48 (m, 1H), 7.38-7.26 (m, 5H), 7.23-7.15 (m, 5H), 7.16-7.06 (m, 2H), 4.20 (dd, J = 9.2, 4.8 Hz, 1H), 3.80-3.41 (m, 4H). ^{13}C NMR (100 MHz, $CDCl_3$) δ 139.43, 138.64, 137.13, 133.40, 128.97, 128.95, 128.67, 128.62, 128.00, 127.93, 127.92, 127.31, 61.41, 43.06, 36.05. HRMS (ESI, m/z) calcd for $C_{21}H_{20}O_2S_2$ $[M+H]^+$ 369.0977, found 369.0977.

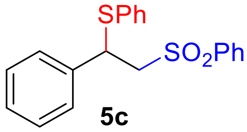

5c

Yield: 92%. ^{1}H NMR (300 MHz, $CDCl_3$) δ 7.54 (d, J = 7.3 Hz, 2H), 7.50-7.44 (m, 1H), 7.33-7.25 (m, 6H), 7.16-6.98 (m, 6H), 4.65 (dd, J = 10.4, 3.8 Hz, 1H), 3.92-3.77 (m, 1H), 3.72-3.60 (m, 1H). ^{13}C NMR (75 MHz, $CDCl_3$) δ 139.28, 137.27, 133.34, 133.19, 132.74, 129.25, 128.91, 128.56, 128.36, 128.03, 127.91, 127.88, 60.54, 47.28. HRMS (ESI, m/z) calcd for $C_{20}H_{18}O_2S_2$ $[M+H]^+$ 355.0821, found 355.0823.

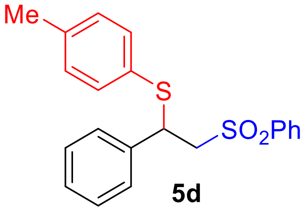

5d

Yield: 84%. ^{1}H NMR (300 MHz, $CDCl_3$) δ 7.52 (d, J = 7.1 Hz, 2H), 7.47-7.42 (m, 1H), 7.32-7.25 (m, 3H), 7.20-7.16 (m, 2H), 7.12-7.05 (m, 6H), 4.57 (dd, J = 10.5, 3.8 Hz, 1H), 3.89-3.77 (m, 1H), 3.69-3.59 (m, 1H), 2.33 (s, 3H). ^{13}C NMR (75 MHz, $CDCl_3$) δ 139.32, 138.78, 137.38, 133.82, 133.28, 130.04, 128.95, 128.86, 128.51, 127.95, 127.92, 127.88, 60.51, 47.68, 21.21. HRMS (ESI, m/z) calcd for $C_{21}H_{20}O_2S_2$ $[M+H]^+$ 369.0977, found 369.0979.

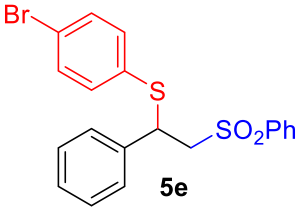

5e

Yield: 65%. ^{1}H NMR (300 MHz, $CDCl_3$) δ 7.57 (d, *J* = 7.3 Hz, 2H), 7.51-7.47 (m, 1H), 7.39-7.30 (m, 4H), 7.15-7.04 (m, 7H), 4.63 (dd, *J* = 10.1, 4.1 Hz, 1H), 3.89-3.77 (m, 1H), 3.68-3.58 (m, 1H). ^{13}C NMR (75 MHz, $CDCl_3$) δ 139.69, 137.67, 135.22, 133.94, 132.79, 129.45, 129.10, 128.64, 128.64, 128.37, 128.35, 123.28, 60.99, 47.85. HRMS (ESI, m/z) calcd for $C_{20}H_{17}BrO_2S_2$ $[M+Na]^+$ 454.9746, found 454.9743.

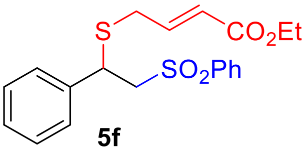

5f

Yield: 53%. ^{1}H NMR (400 MHz, $CDCl_3$) δ 7.65 (d, *J* = 7.4 Hz, 2H), 7.56-7.48 (m, 1H), 7.42-7.33 (m, 2H), 7.20-7.12 (m, 5H), 6.78-6.66 (m, 1H), 5.74 (d, *J* = 15.5 Hz, 1H), 4.31-4.16 (m, 3H), 3.78-3.57 (m, 2H), 3.11-2.89 (m, 2H), 1.31 (t, *J* = 7.1 Hz, 3H). ^{13}C NMR (100 MHz, $CDCl_3$) δ 165.79, 142.61, 139.33, 138.21, 133.56, 129.06, 128.81, 128.18, 128.03, 127.95, 123.62, 61.34, 60.57, 42.85, 32.33, 14.28. HRMS (ESI, m/z) calcd for $C_{20}H_{22}O_4S_2$ $[M+H]^+$ 391.1032, found 391.1035.

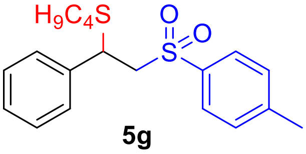

5g

Yield: 72%. ^{1}H NMR (500 MHz, $CDCl_3$) δ 7.52 (d, *J* = 8.3 Hz, 2H), 7.20-7.05 (m, 7H), 4.30 (dd, *J* = 9.4, 4.6 Hz, 1H), 3.79-3.68 (m, 1H), 3.66-3.57 (m, 1H), 2.37 (s, 3H), 2.34-2.20 (m, 2H), 1.49-1.38 (m, 2H), 1.36-1.23 (m, 2H), 0.83 (t, *J* = 7.3 Hz, 3H). ^{13}C NMR (126 MHz, $CDCl_3$) δ 144.37, 139.11, 136.54, 129.57, 128.56, 127.97, 127.82, 127.62, 61.65, 43.29, 31.22, 31.05, 21.89, 21.58, 13.60. HRMS (ESI, m/z) calcd for $C_{19}H_{24}O_2S_2$ $[M+Na]^+$ 371.1110, found 371.1121.

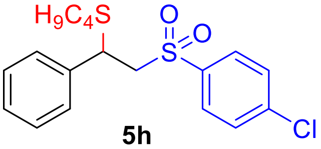

5h

Yield: 56%. ^{1}H NMR (500 MHz, $CDCl_3$) δ 7.56-7.46 (m, 2H), 7.32-7.27 (m, 2H), 7.21 -7.13 (m, 3H), 7.12-7.08 (m, 2H), 4.30 (dd, *J* = 9.7, 4.6 Hz, 1H), 3.80-3.70 (m, 1H), 3.70-3.61 (m, 1H), 2.36-2.25 (m, 2H), 1.50-1.37 (m, 2H), 1.37-1.20 (m, 3H), 0.84 (d, *J* = 7.3 Hz, 3H). ^{13}C NMR (126 MHz, $CDCl_3$) δ 140.09, 138.64, 137.95, 130.95, 129.40, 129.16, 128.67, 127.84, 61.59, 43.37, 31.24, 31.04, 21.88, 13.59. HRMS (ESI, m/z) calcd for $C_{18}H_{21}ClO_2S_2$ $[M+Na]^+$ 391.0564, found 391.0562.

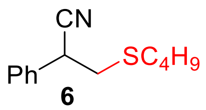

6

Yield: 80%. ^{1}H NMR (300 MHz, $CDCl_3$) δ 7.36-7.25 (m, 5H), 3.91 (dd, *J* = 8.2, 6.4 Hz, 1H), 3.01-2.82 (m, 2H), 2.54-2.40 (m, 2H), 1.51-1.44 (m, 2H), 1.37-1.25 (m, 2H), 0.83 (t, *J* = 7.3 Hz, 3H). ^{13}C NMR (75 MHz, $CDCl_3$) δ 134.71, 129.17, 128.56, 127.48, 119.97, 39.19, 37.64, 32.75, 31.55, 21.83, 13.59. HRMS (ESI, m/z) calcd for $C_{13}H_{17}NS$ $[M+H]^+$ 220.1154, found 220.1155.

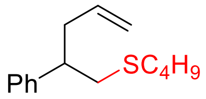

7

Yield: 63%. ^{1}H NMR (400 MHz, $CDCl_3$) δ 7.33-7.25 (m, 2H), 7.21-7.09 (m, 3H), 5.63 (m, 1H), 5.03-4.87 (m, 2H), 2.88-2.68 (m, 3H), 2.62-2.52 (m, 1H), 2.45-2.32 (m, 3H), 1.53-1.44 (m, 2H), 1.38-1.28 (m, 2H), 0.86 (t, *J* = 7.3 Hz, 3H). ^{13}C NMR (100 MHz, $CDCl_3$) δ 143.76, 136.30, 128.38, 127.68, 126.57, 116.52, 46.00, 39.70, 38.54, 32.48, 31.76, 21.98, 13.68. HRMS (ESI, m/z) calcd for $C_{15}H_{22}S$ $[M+H]^+$ 235.1515, found 235.1514.

IPrAuSCF$_3$ (**8**)

^{1}H NMR (400 MHz, CD_3CN) δ 7.62-7.48 (m, 4H), 7.45-7.32 (m, 4H), 2.56 (m, 4H), 1.29 (d, *J* = 6.9 Hz, 12H), 1.23 (d, *J* = 6.9 Hz, 12H). ^{13}C NMR (100 MHz, CD_3CN) δ 165.45, 145.94, 133.99, 130.63, 124.12, 117.32, 28.63, 23.54, 23.12(SCF_3 was not observed). ^{19}F NMR (282 MHz, $CDCl_3$) δ -22.49. HRMS (ESI, m/z) calcd for $C_{28}H_{36}Au$ F_3N_2S $[M+H]^+$ 687.2290, found 687.2293.

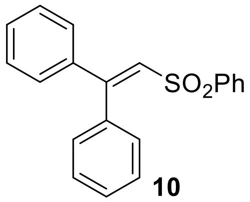

10

Known compound[5]. ^{1}H NMR (400 MHz, $CDCl_3$) δ 7.59 (d, *J* = 7.5 Hz, 2H), 7.53-7.45 (m, 1H), 7.40-7.25 (m, 8H), 7.24-7.19 (m, 2H), 7.12-7.06 (m, 2H), 7.04 (s, 1H).

(5) Russell, G. A.; Ngoviwatchai, P.; Tashtoush, H. 1.; Pla-Dalmau, A.; Khanna, R. K. *J. Am. Chem. Soc.* **1988**, *110*, 3530.

NMR spectra for the products

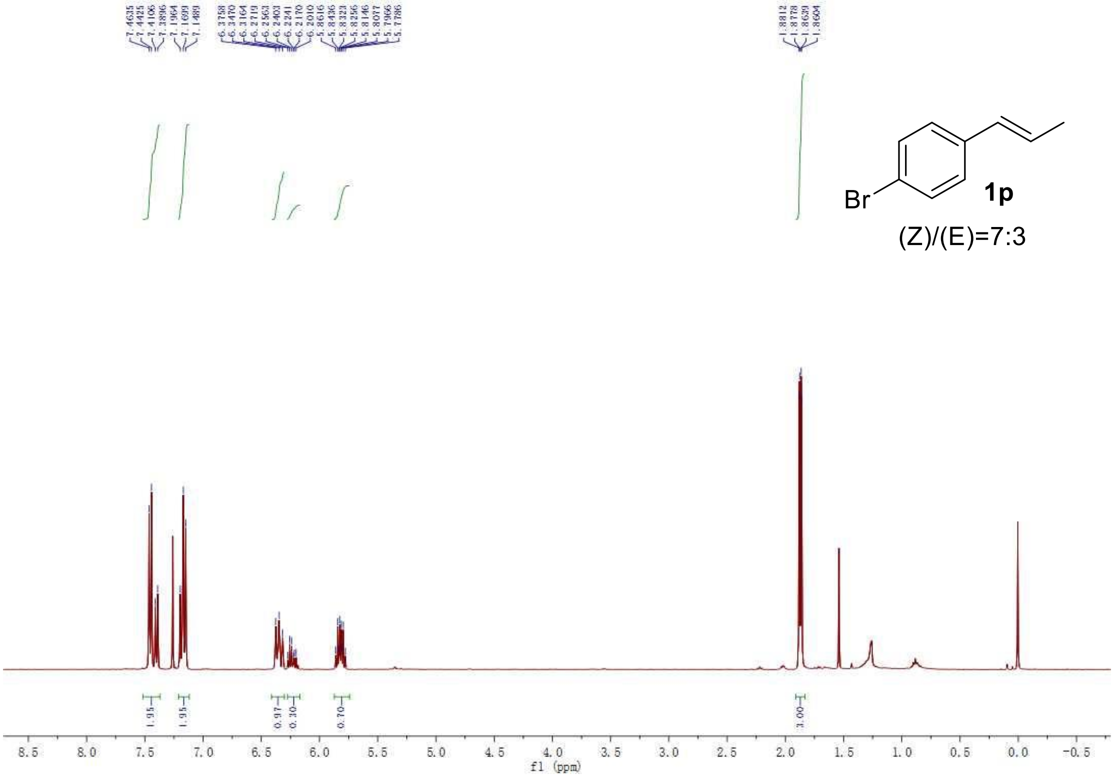

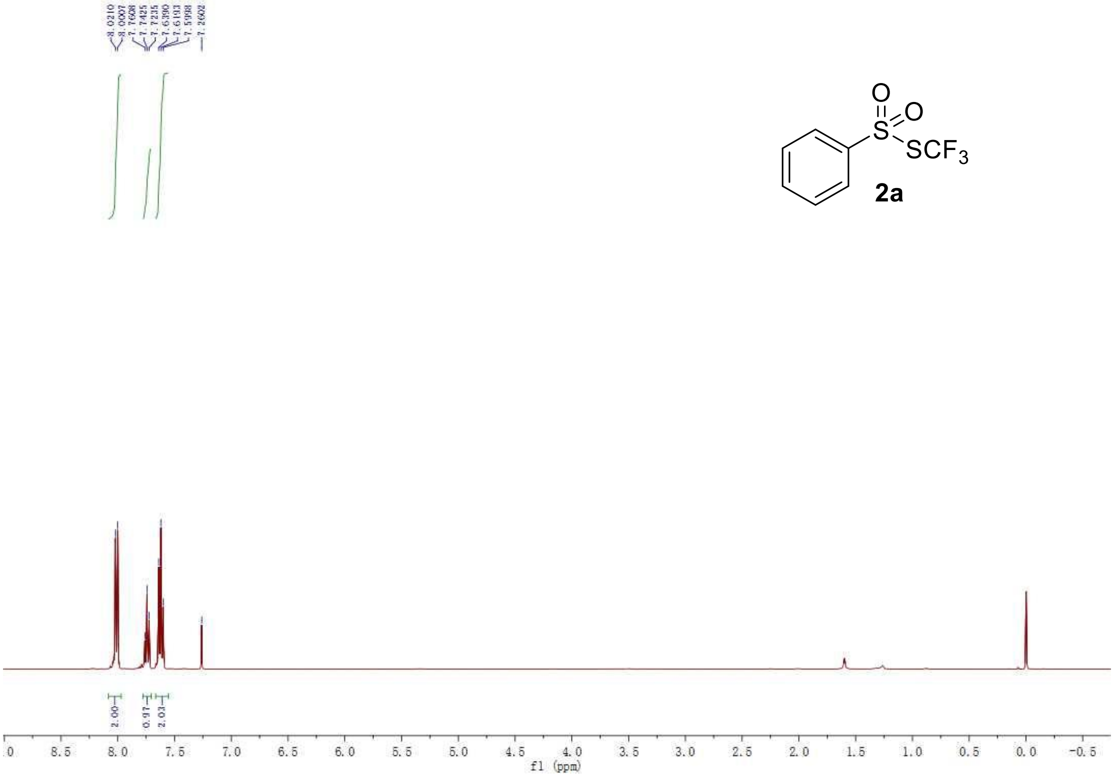

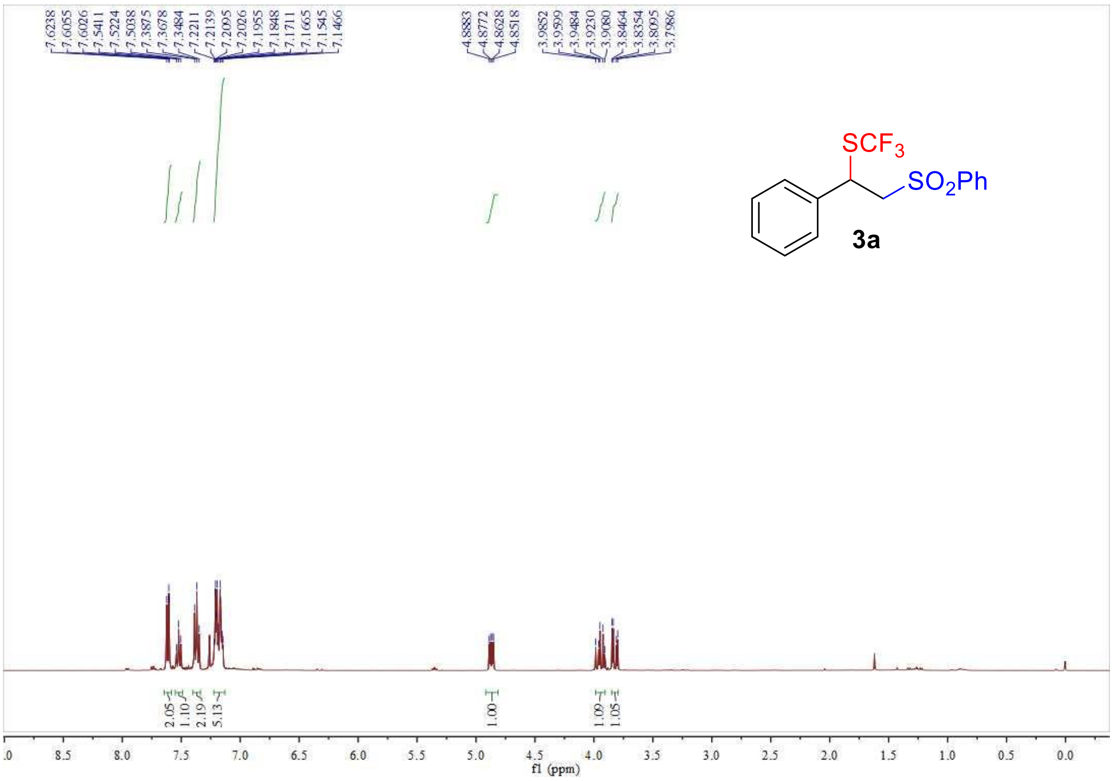
7.6238 7.6055 7.6026 7.5411 7.5224 7.5038 7.3875 7.3678 7.3484 7.2211 7.2139 7.2095 7.2026 7.1955 7.1848 7.1711 7.1665 7.1545 7.1466
4.8883 4.8772 4.8628 4.8518
3.9852 3.9599 3.9484 3.9230 3.9080 3.8464 3.8354 3.8095 3.7986
SCF3
SO2Ph
3a
2.05 1.10 2.19 5.13
1.00
1.09 1.05
f1 (ppm)

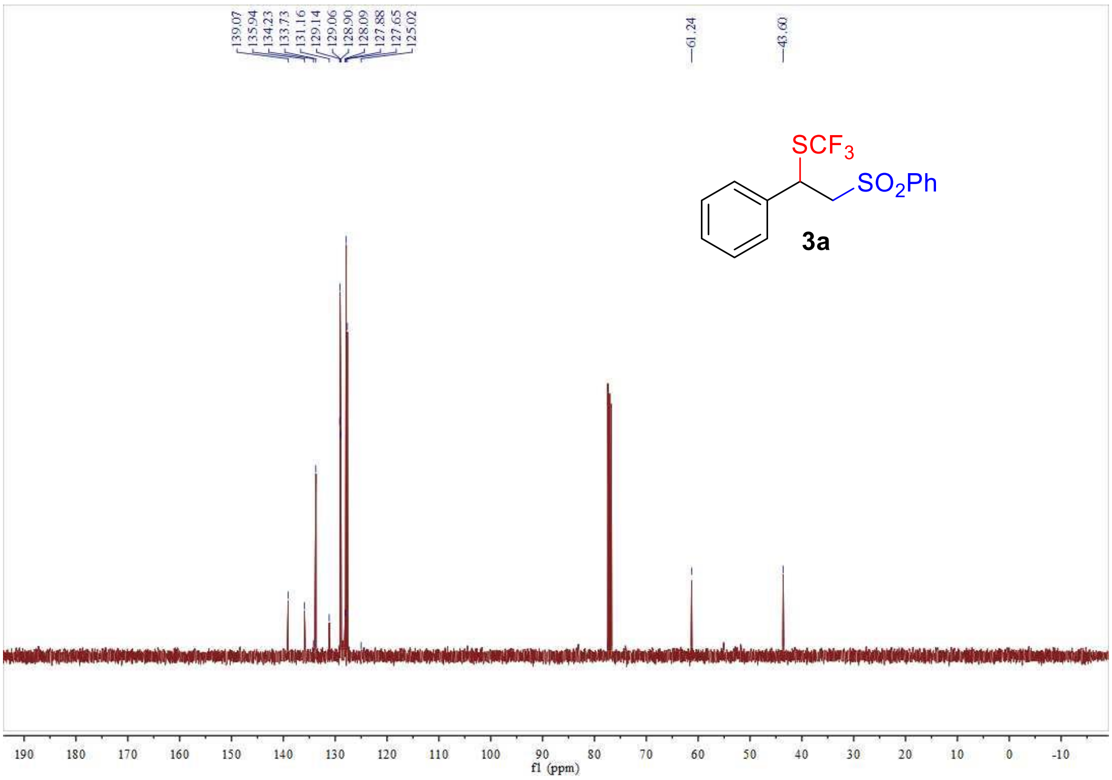
139.07 135.94 134.23 133.73 131.16 129.14 129.06 128.90 128.09 127.88 127.65 125.02
61.24
43.60
SCF3
SO2Ph
3a
f1 (ppm)

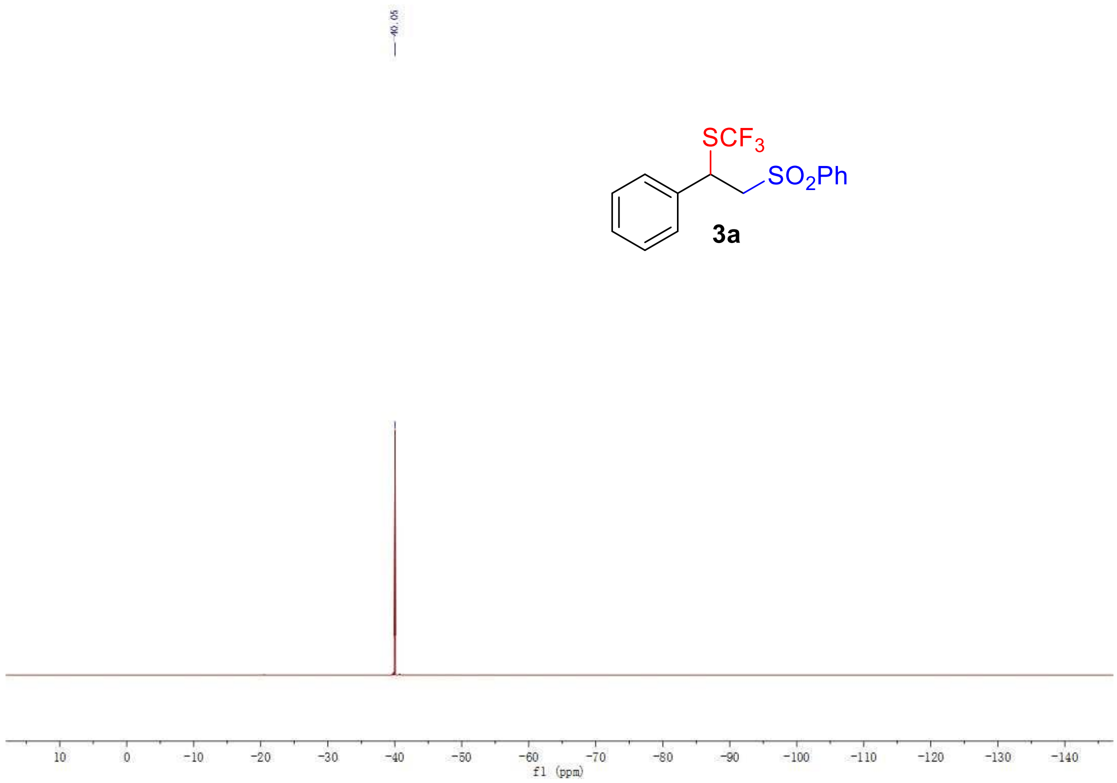
-40.05
SCF3
SO2Ph
3a
10 0 -10 -20 -30 -40 -50 -60 -70 -80 -90 -100 -110 -120 -130 -140
f1 (ppm)

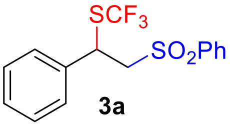

3a

Chemical Formula: $C_{15}H_{13}F_3O_2S_2$
Exact Mass: 346.0309
Molecular Weight: 346.3822
m/z: 346.0309 (100.0%), 347.0343 (16.2%), 348.0267 (9.0%), 347.0303 (1.6%), 349.0301 (1.5%), 348.0376 (1.2%)
Elemental Analysis: C, 52.01; H, 3.78; F, 16.45; O, 9.24; S, 18.51

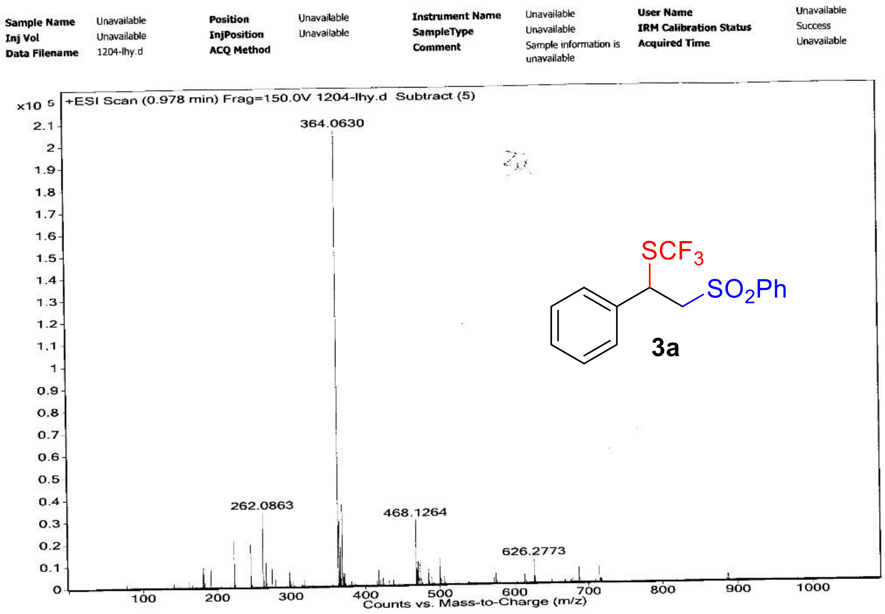

HRMS (ESI, m/z) calcd for $C_{15}H_{13}F_3O_2S_2$ $[M+NH_4]^+$ 364.0647, found 364.0630.

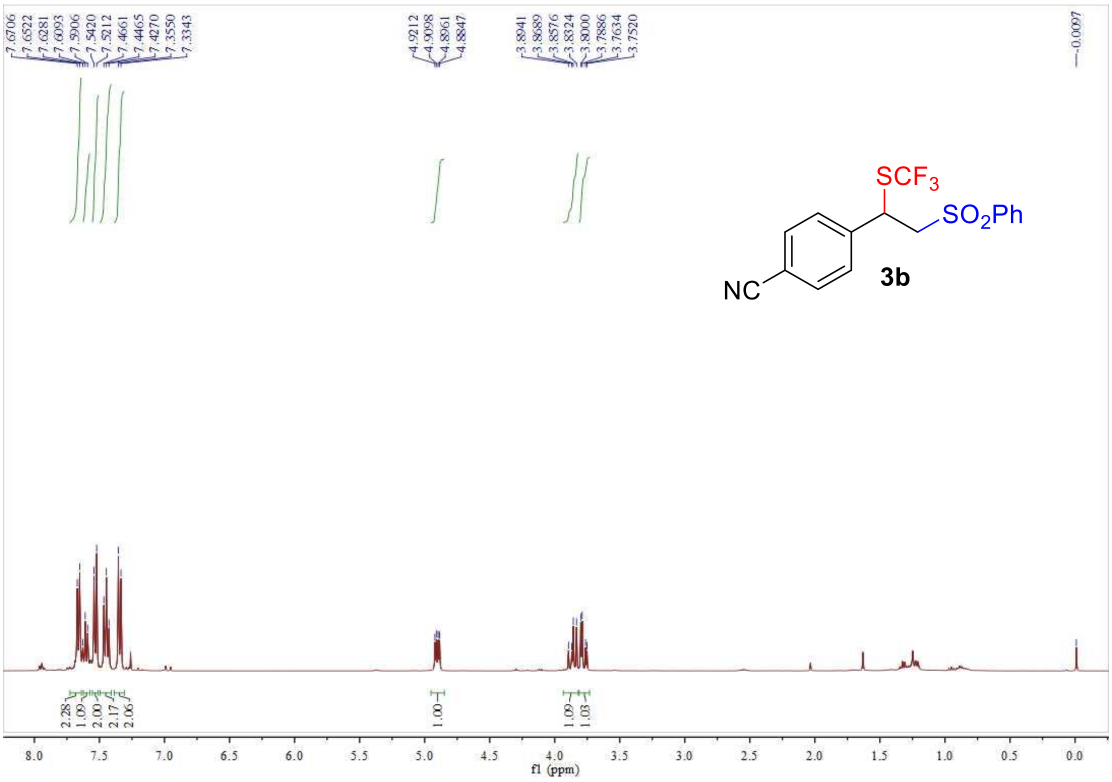
7.6706
7.6522
7.6281
7.6093
7.5906
7.5420
7.5212
7.4661
7.4465
7.4270
7.3550
7.3343
4.9212
4.9098
4.8961
4.8847
3.8941
3.8689
3.8576
3.8324
3.8000
3.7886
3.7634
3.7520
-0.0097
SCF3
SO2Ph
NC
3b
2.28
1.09
2.00
2.17
2.06
1.00
1.09
1.03
f1 (ppm)

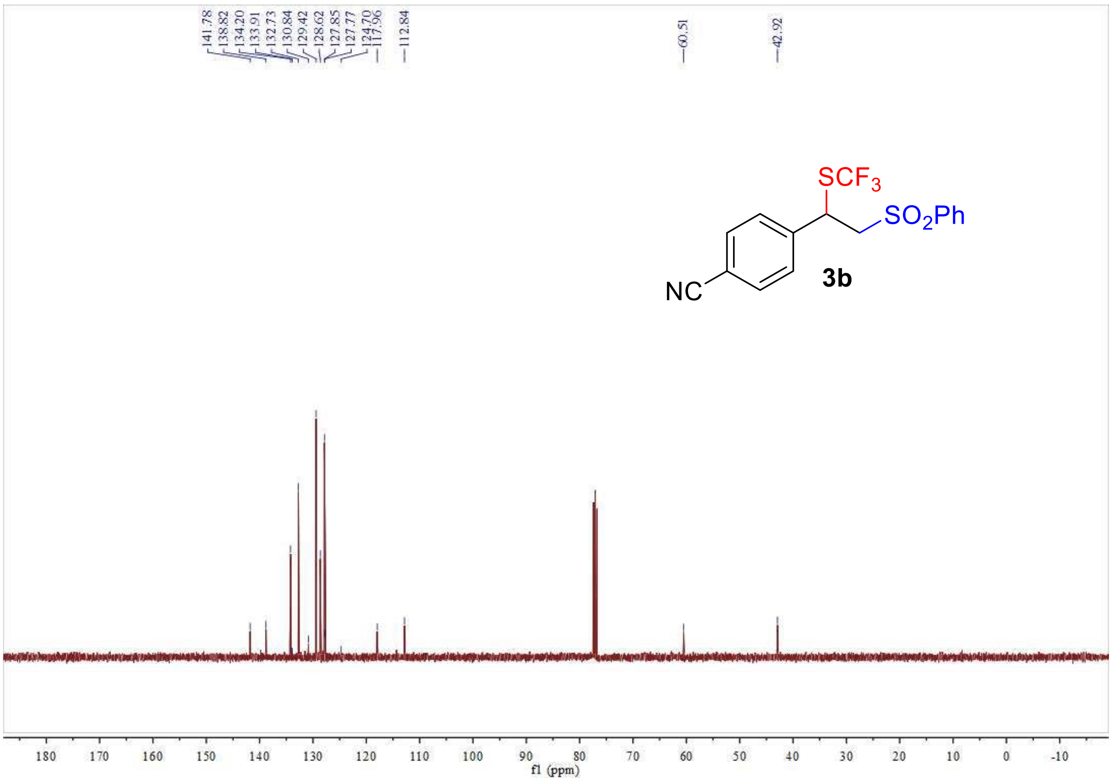
141.78
138.82
134.20
133.91
132.73
130.84
129.42
128.62
127.85
127.77
124.70
117.96
112.84
60.51
42.92
SCF3
SO2Ph
NC
3b
f1 (ppm)

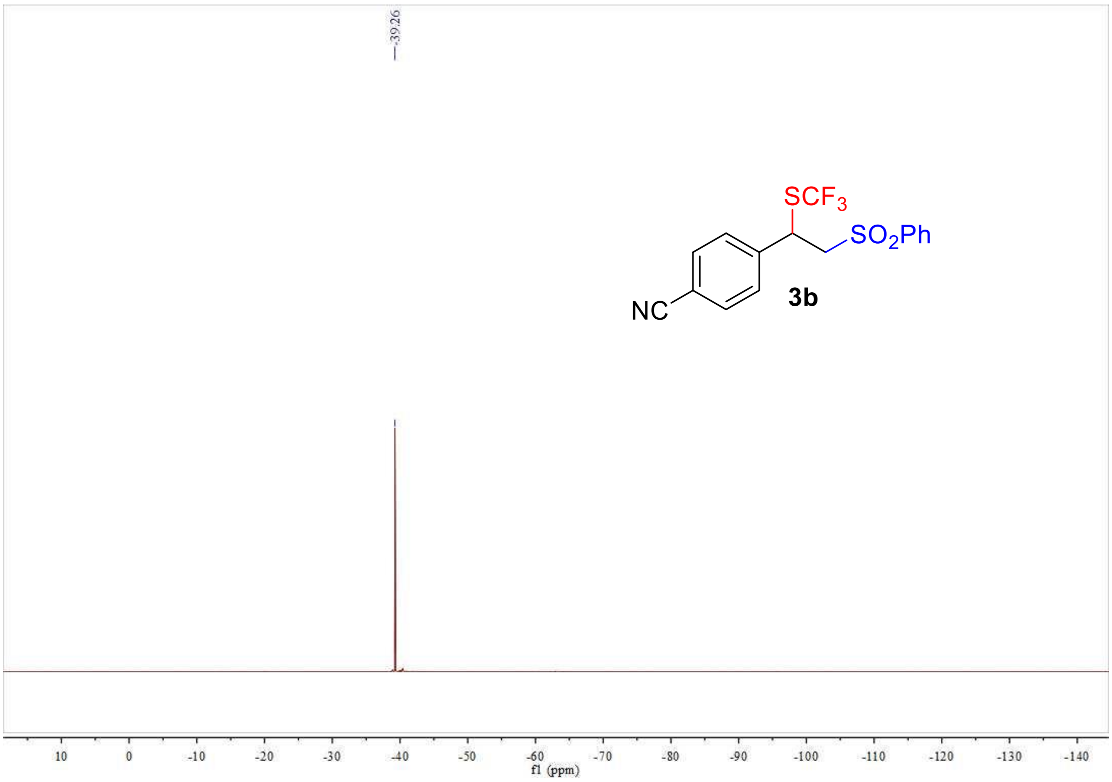
-39.26
SCF3
SO2Ph
NC
3b
10
0
-10
-20
-30
-40
-50
-60
-70
-80
-90
-100
-110
-120
-130
-140
f1 (ppm)

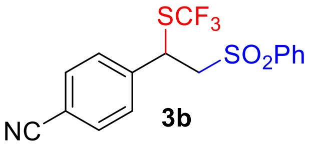

3b

Chemical Formula: $C_{16}H_{12}F_3NO_2S_2$
Exact Mass: 371.0262
Molecular Weight: 371.3922
m/z: 371.0262 (100.0%), 372.0295 (17.3%), 373.0220 (9.0%), 372.0255 (1.6%), 374.0253 (1.6%), 373.0329 (1.4%)
Elemental Analysis: C, 51.74; H, 3.26; F, 15.35; N, 3.77; O, 8.62; S, 17.26

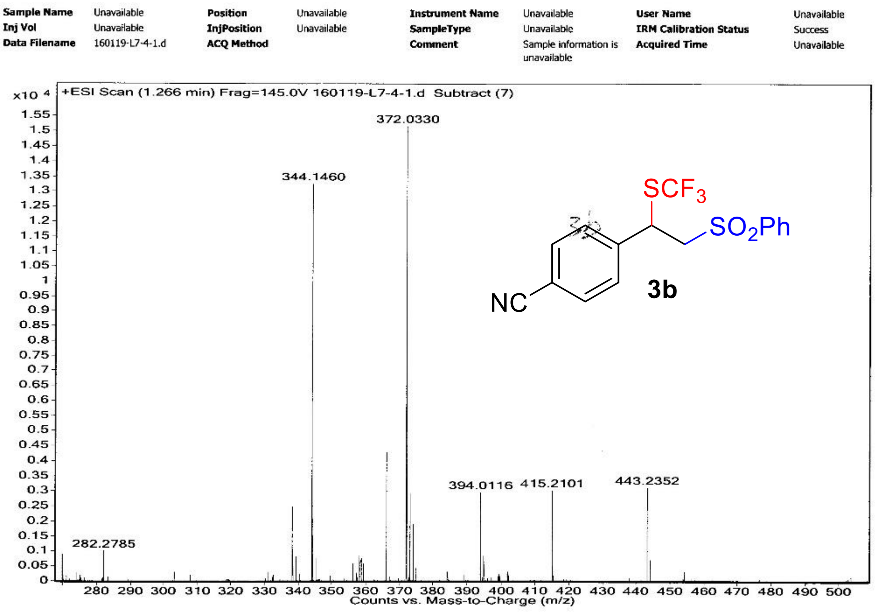

HRMS (ESI, m/z) calcd for $C_{16}H_{12}F_3NO_2S_2$ $[M+H]^+$ 372.0334, found 372.0330.

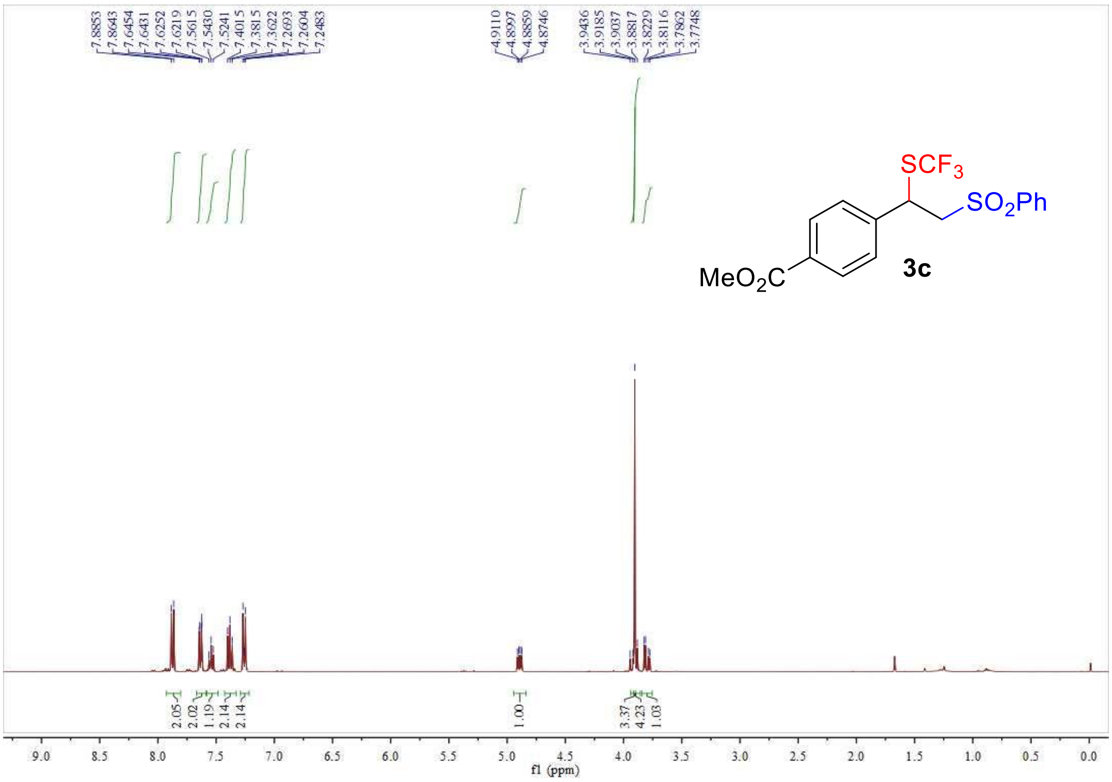
7.8853
7.8643
7.6454
7.6431
7.6252
7.6219
7.5615
7.5430
7.5241
7.4015
7.3815
7.3622
7.2693
7.2604
7.2483
4.9110
4.8997
4.8859
4.8746
3.9436
3.9185
3.9037
3.8817
3.8229
3.8116
3.7862
3.7748
SCF3
SO2Ph
MeO2C
3c
2.05
2.02
1.19
2.14
2.14
1.00
3.37
4.23
1.03
f1 (ppm)

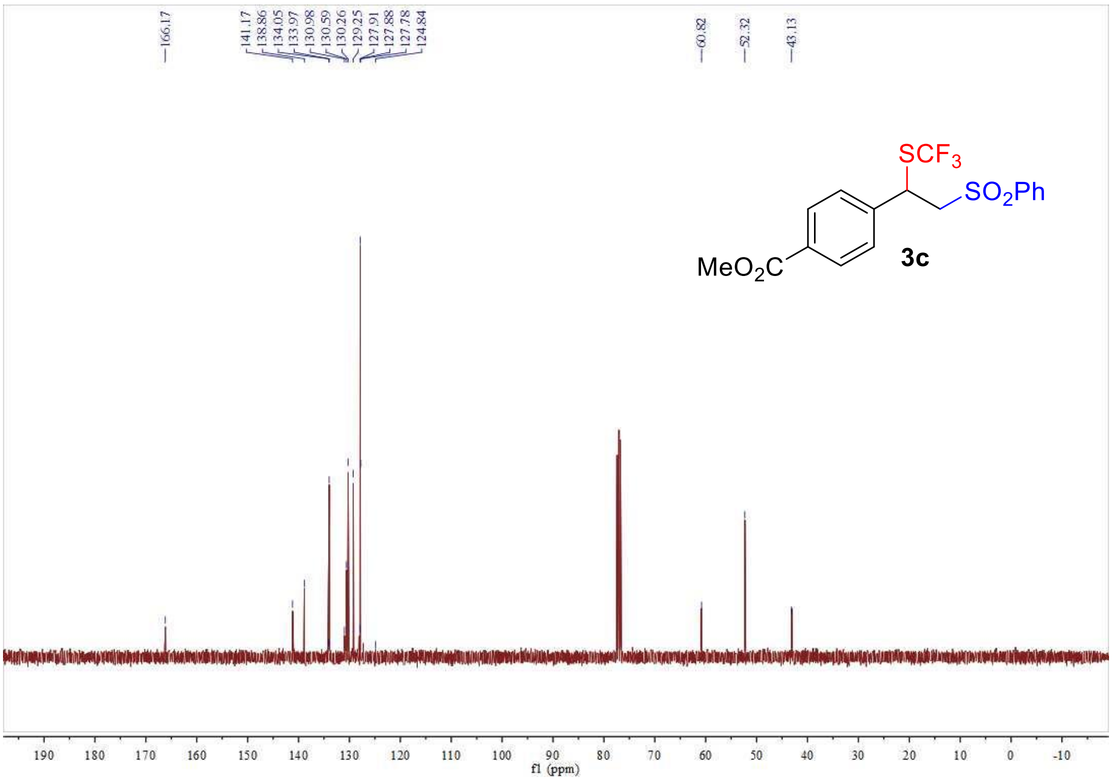
166.17
141.17
138.86
134.05
133.97
130.98
130.59
130.26
129.25
127.91
127.88
127.78
124.84
60.82
52.32
43.13
SCF3
SO2Ph
MeO2C
3c
f1 (ppm)

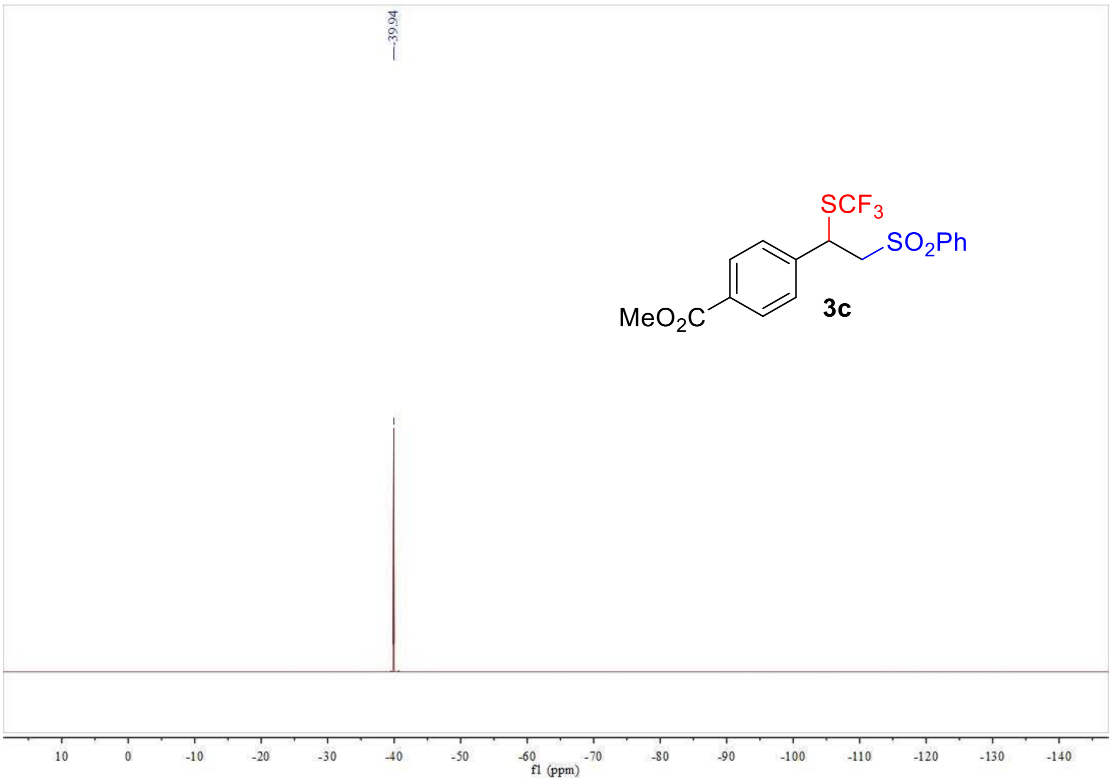

-39.94
SCF3
SO2Ph
MeO2C
3c
10
0
-10
-20
-30
-40
-50
-60
-70
-80
-90
-100
-110
-120
-130
-140
f1 (ppm)

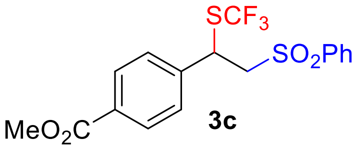

3c

Chemical Formula: $C_{17}H_{15}F_3O_4S_2$
Exact Mass: 404.0364
Molecular Weight: 404.4182
m/z: 404.0364 (100.0%), 405.0397 (18.4%), 406.0322 (9.0%), 407.0355 (1.7%), 405.0358 (1.6%), 406.0431 (1.6%)
Elemental Analysis: C, 50.49; H, 3.74; F, 14.09; O, 15.82; S, 15.85

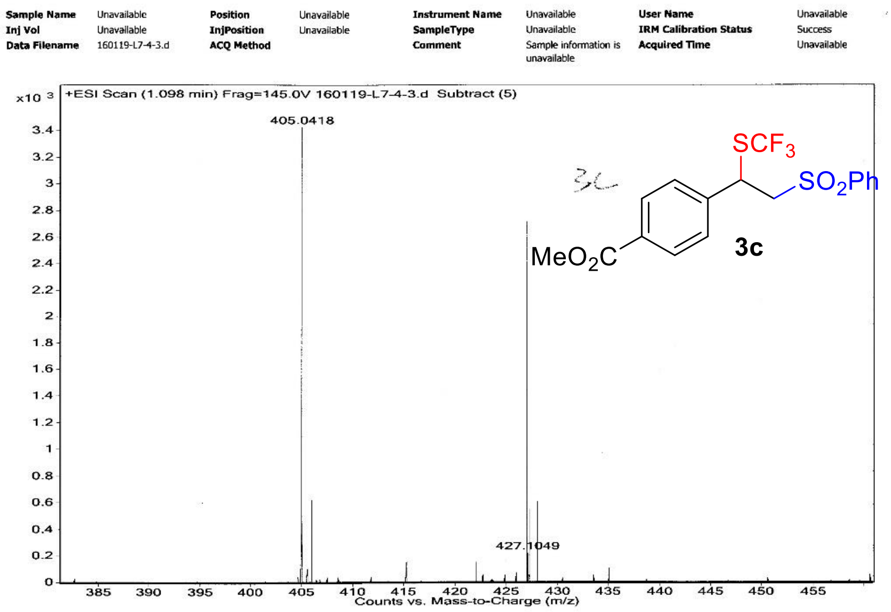

HRMS (ESI, m/z) calcd for $C_{17}H_{15}F_3O_4S_2$ $[M+H]^+$ 405.0437, found 405.0418.

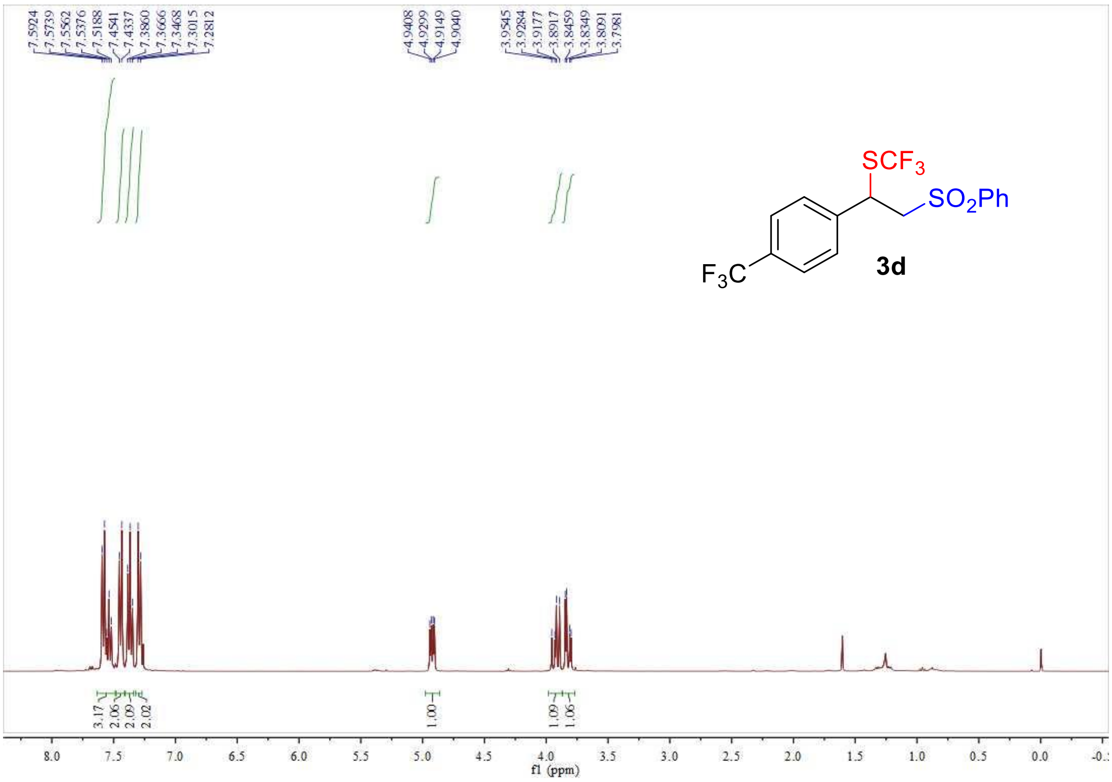
7.5924
7.5739
7.5562
7.5376
7.5188
7.4541
7.4337
7.3860
7.3666
7.3468
7.3015
7.2812
4.9408
4.9299
4.9149
4.9040
3.9545
3.9284
3.9177
3.8917
3.8459
3.8349
3.8091
3.7981
SCF3
SO2Ph
F3C
3d
3.17
2.06
2.09
2.02
1.00
1.09
1.06
f1 (ppm)

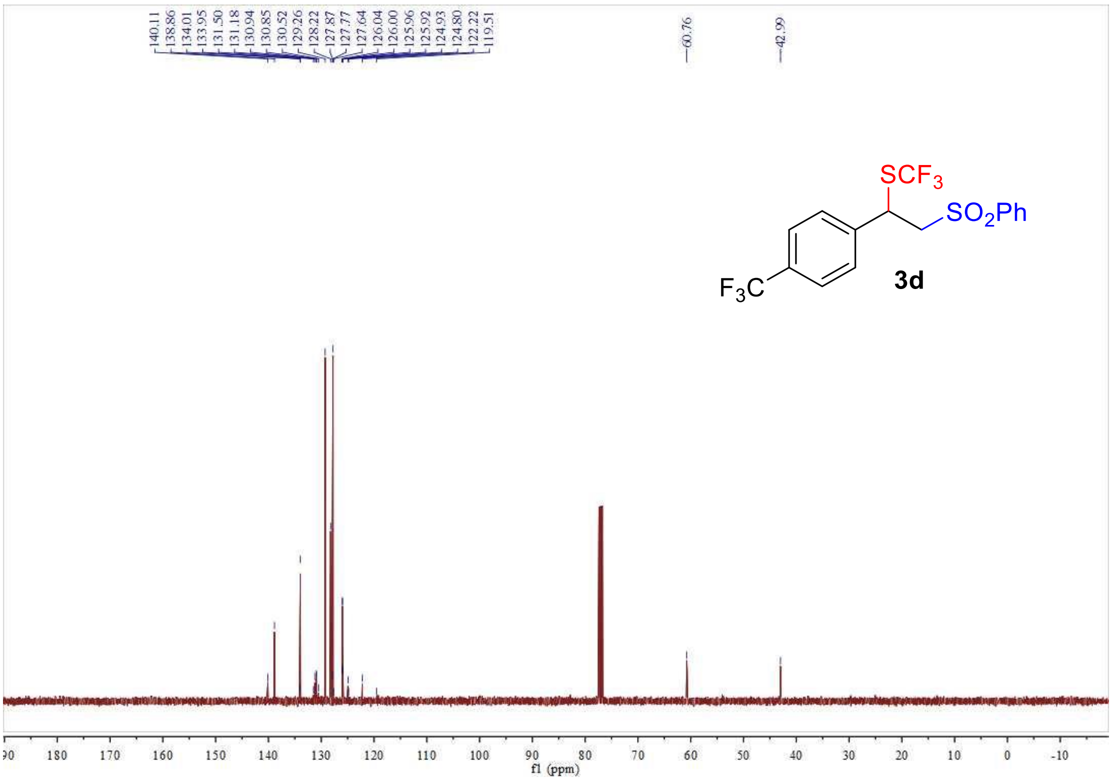
140.11
138.86
134.01
133.95
131.50
131.18
130.94
130.85
130.52
129.26
128.22
127.87
127.77
127.64
126.04
126.00
125.96
125.92
124.93
124.80
122.22
119.51
60.76
42.99
SCF3
SO2Ph
F3C
3d
f1 (ppm)

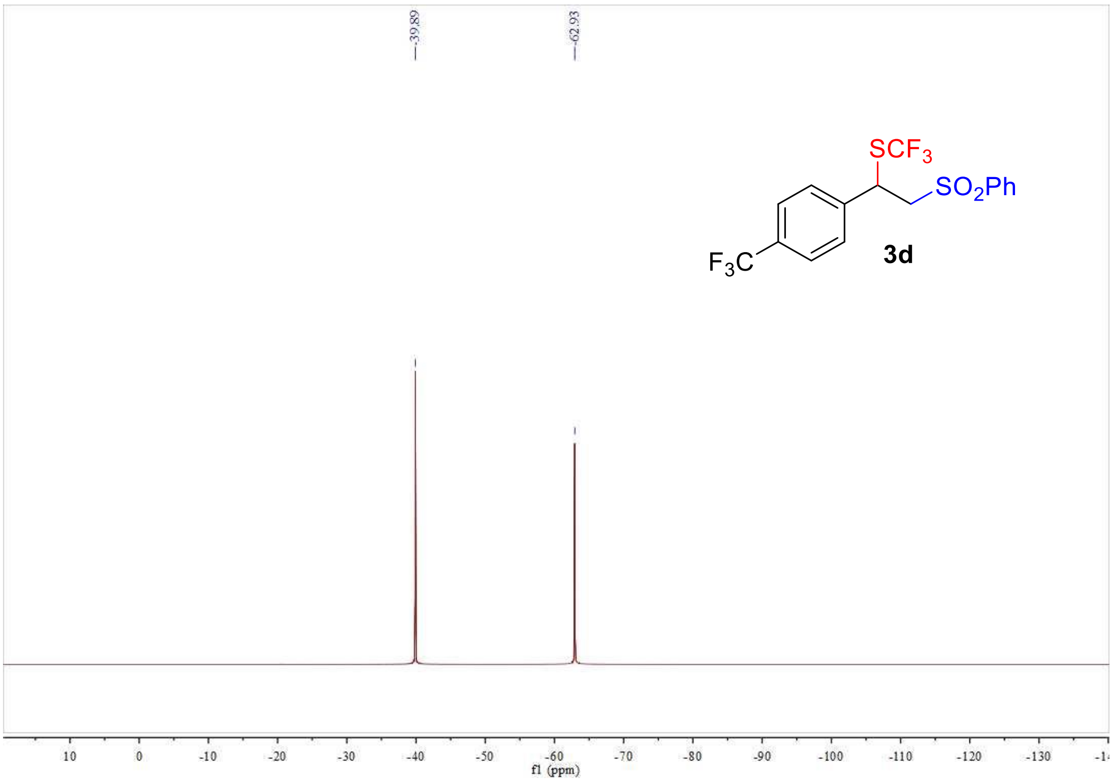

-39.89
-62.93
SCF3
SO2Ph
F3C
3d
10
0
-10
-20
-30
-40
-50
-60
-70
-80
-90
-100
-110
-120
-130
f1 (ppm)

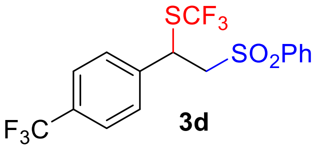

3d

Chemical Formula: $C_{16}H_{12}F_6O_2S_2$

Exact Mass: 414.0183

Molecular Weight: 414.3804

m/z: 414.0183 (100.0%), 415.0216 (17.3%), 416.0141 (9.0%), 415.0177 (1.6%), 417.0174 (1.6%), 416.0250 (1.4%)

Elemental Analysis: C, 46.38; H, 2.92; F, 27.51; O, 7.72; S, 15.47

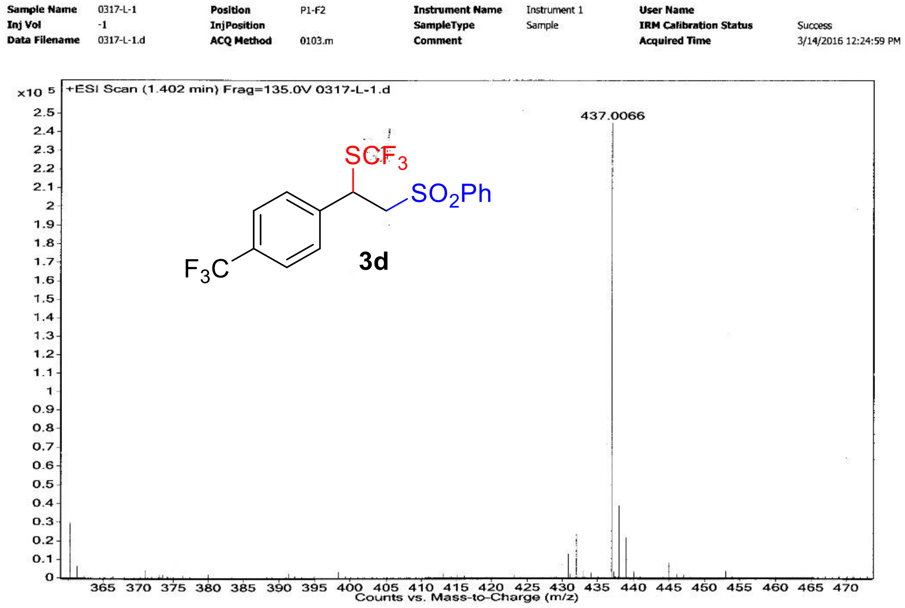

HRMS (ESI, m/z) calcd for $C_{16}H_{12}F_6O_2S_2$ $[M+Na]^+$ 437.0075, found 437.0075.

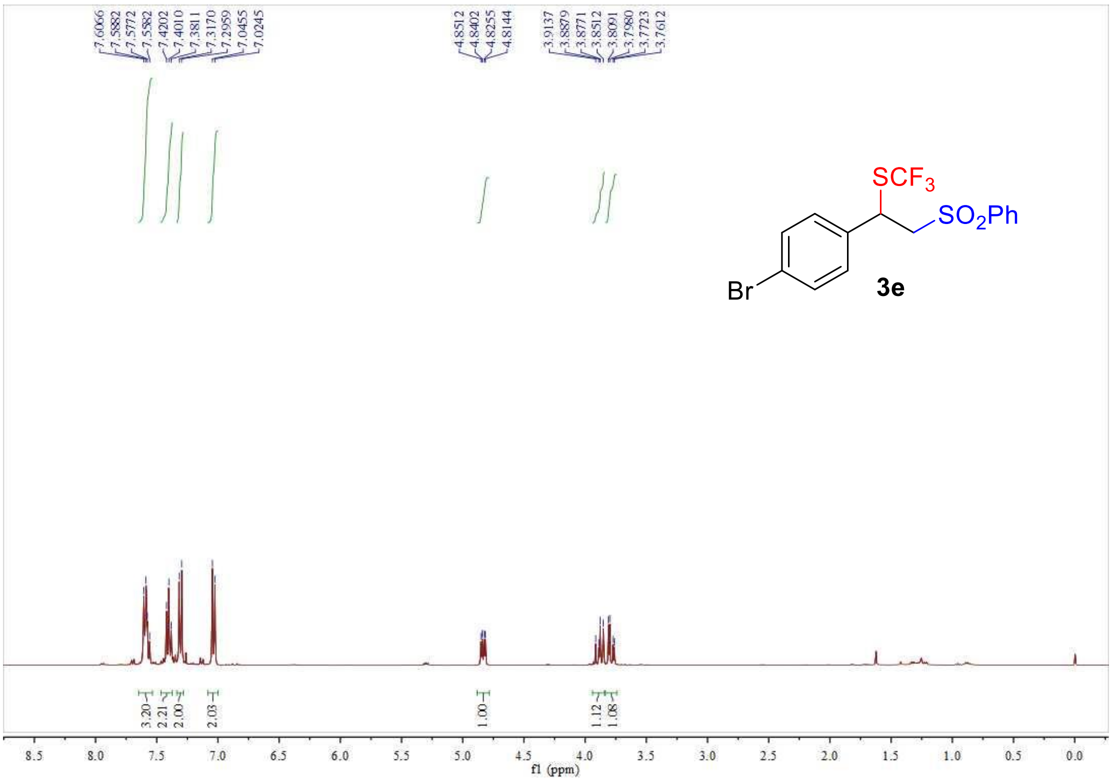
7.6066 7.5882 7.5772 7.5582 7.4202 7.4010 7.3811 7.3170 7.2959 7.0455 7.0245
4.8512 4.8402 4.8255 4.8144
3.9137 3.8879 3.8771 3.8512 3.8091 3.7980 3.7723 3.7612
SCF3
SO2Ph
Br
3e
3.20 2.21 2.00 2.03 1.00 1.12 1.08
8.5 8.0 7.5 7.0 6.5 6.0 5.5 5.0 4.5 4.0 3.5 3.0 2.5 2.0 1.5 1.0 0.5 0.0
f1 (ppm)

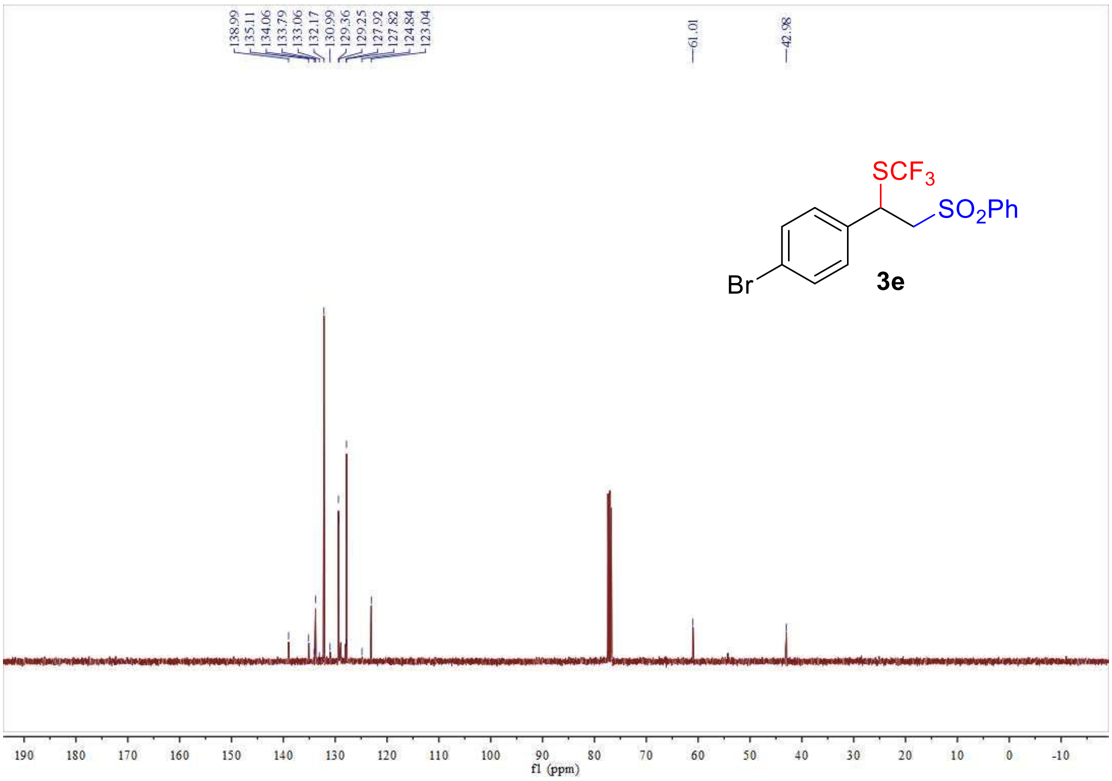
138.99 135.11 134.06 133.79 133.06 132.17 130.99 129.36 129.25 127.92 127.82 124.84 123.04
61.01
42.98
SCF3
SO2Ph
Br
3e
190 180 170 160 150 140 130 120 110 100 90 80 70 60 50 40 30 20 10 0 -10
f1 (ppm)

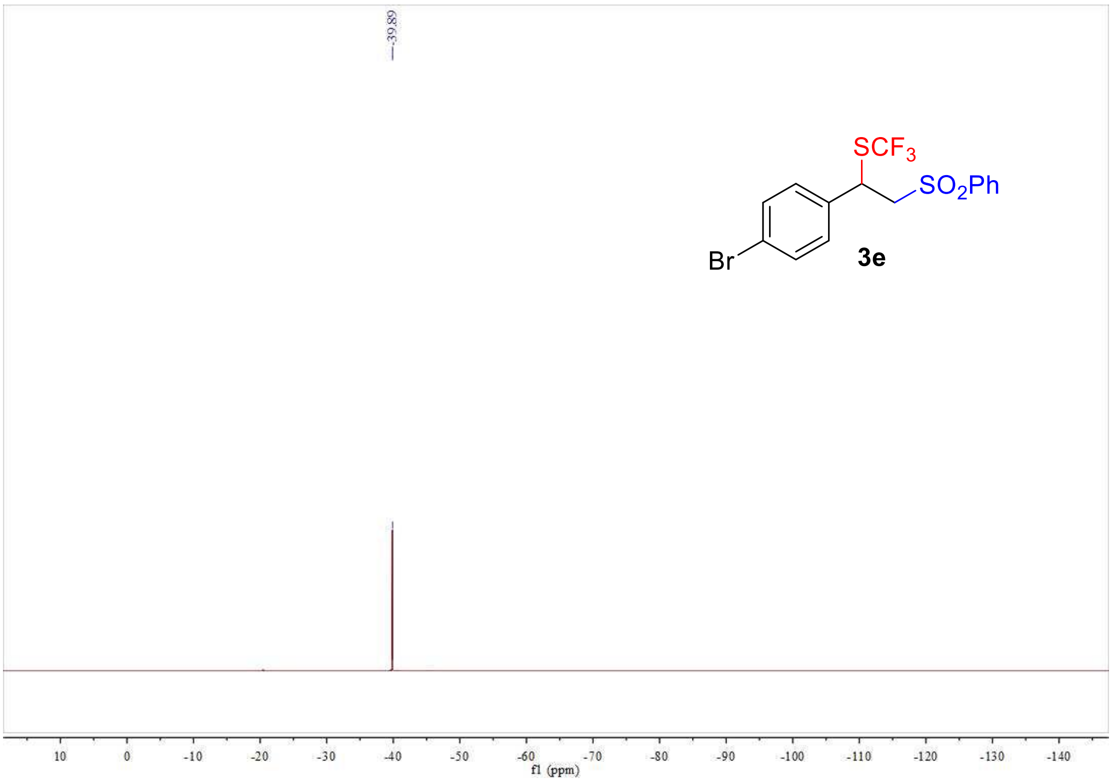

—39.89
SCF3
SO2Ph
Br
3e
10
0
-10
-20
-30
-40
-50
-60
-70
-80
-90
-100
-110
-120
-130
-140
f1 (ppm)

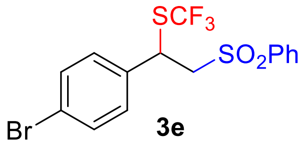

3e

Chemical Formula: $C_{15}H_{12}BrF_3O_2S_2$
Exact Mass: 423.9414
Molecular Weight: 425.2782
m/z: 423.9414 (100.0%), 425.9394 (97.3%), 424.9448 (16.2%), 426.9427 (15.8%), 425.9372 (9.0%), 427.9352 (8.8%), 424.9408 (1.6%), 426.9388 (1.6%), 426.9406 (1.5%), 428.9385 (1.4%), 425.9481 (1.2%), 427.9461 (1.2%)
Elemental Analysis: C, 42.36; H, 2.84; Br, 18.79; F, 13.40; O, 7.52; S, 15.08

**Sample Name**	2016-0309-L7-13-4	**Position**	P1-D9	**Instrument Name**	Instrument 1	**User Name**	
**Inj Vol**	-1	**InjPosition**		**SampleType**	Sample	**IRM Calibration Status**	Success
**Data Filename**	2016-0309-L7-13-4.d	**ACQ Method**	0103.m	**Comment**		**Acquired Time**	3/6/2016 2:48:37 PM

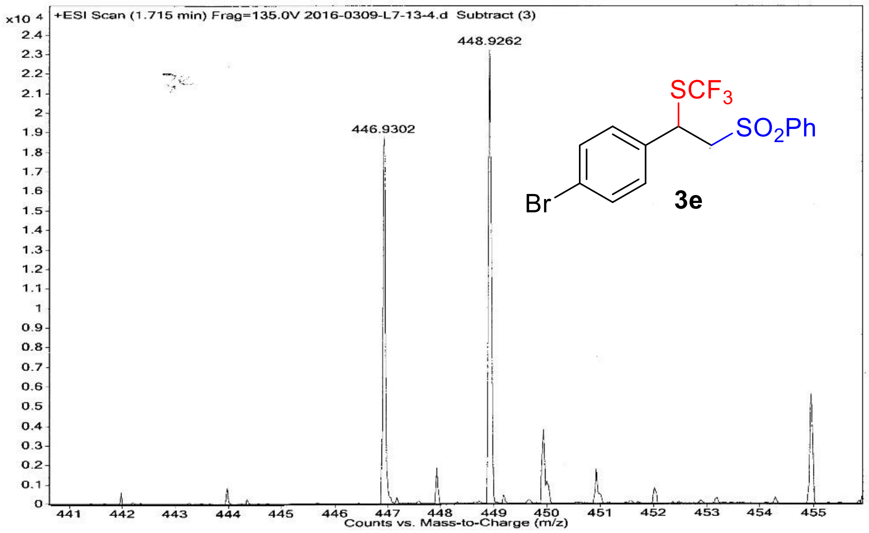

HRMS (ESI, m/z) calcd for $C_{15}H_{12}BrF_3O_2S_2$ $[M+Na]^+$ 446.9306, found 446.9302.

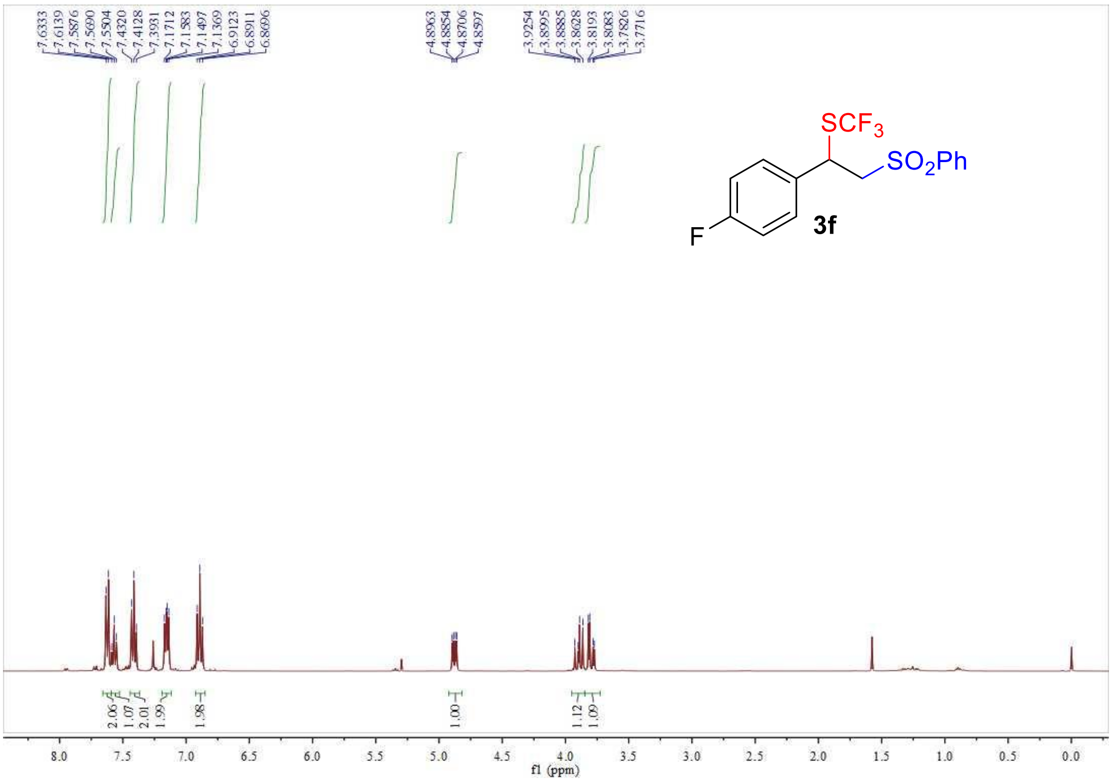
7.6333
7.6139
7.5876
7.5690
7.5504
7.4320
7.4128
7.3931
7.1712
7.1583
7.1497
7.1369
6.9123
6.8911
6.8696
4.8963
4.8854
4.8706
4.8597
3.9254
3.8995
3.8885
3.8628
3.8193
3.8083
3.7826
3.7716
SCF3
SO2Ph
F
3f
2.06
1.07
2.01
1.99
1.98
1.00
1.12
1.09
f1 (ppm)

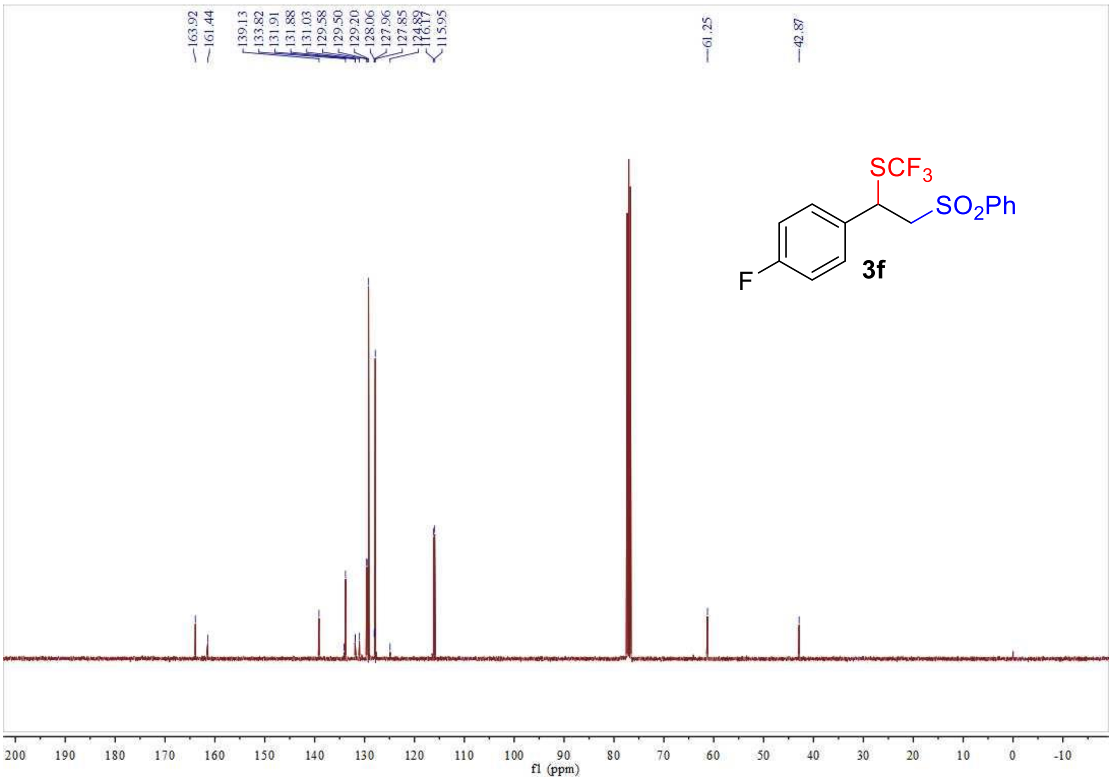
163.92
161.44
139.13
133.82
131.91
131.88
131.03
129.58
129.50
129.20
128.06
127.96
127.85
124.89
116.17
115.95
61.25
42.87
SCF3
SO2Ph
F
3f
f1 (ppm)

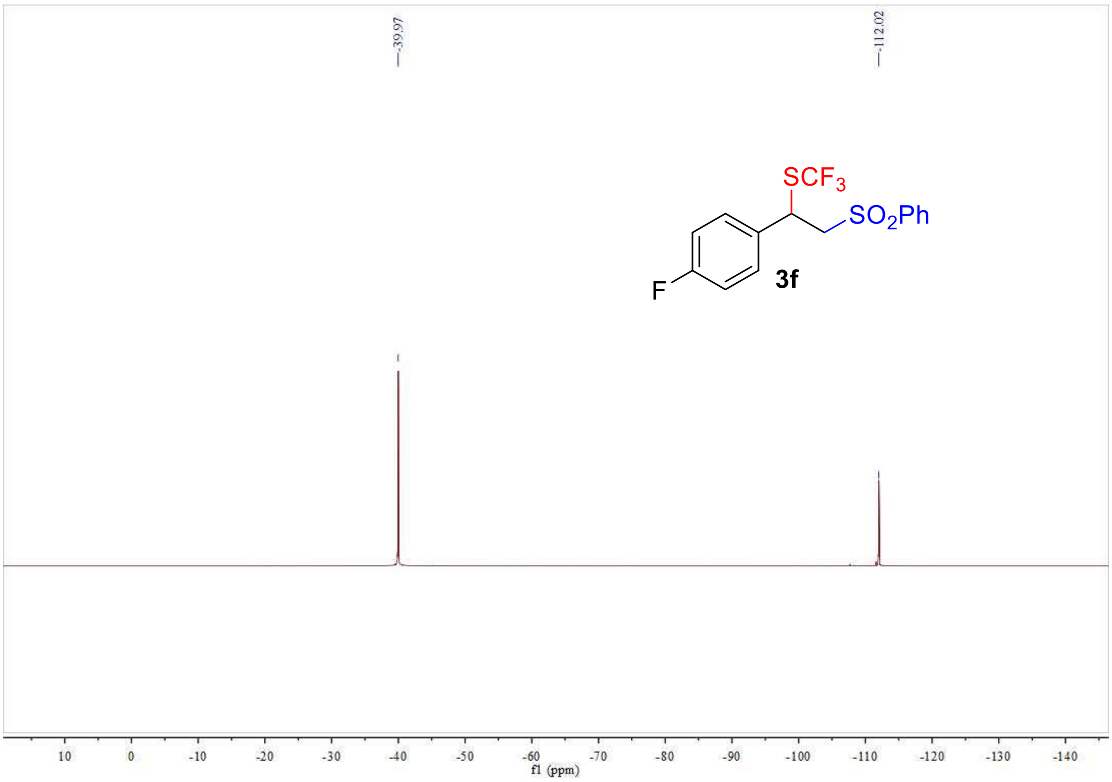
-39.97
-112.02
SCF3
SO2Ph
F
3f
10
0
-10
-20
-30
-40
-50
-60
-70
-80
-90
-100
-110
-120
-130
-140
f1 (ppm)

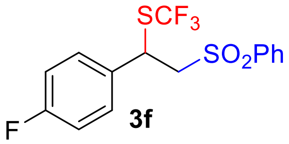

3f

Chemical Formula: $C_{15}H_{12}F_4O_2S_2$
Exact Mass: 364.0215
Molecular Weight: 364.3726
m/z: 364.0215 (100.0%), 365.0248 (16.2%), 366.0173 (9.0%), 365.0209 (1.6%), 367.0206 (1.5%), 366.0282 (1.2%)
Elemental Analysis: C, 49.45; H, 3.32; F, 20.86; O, 8.78; S, 17.60

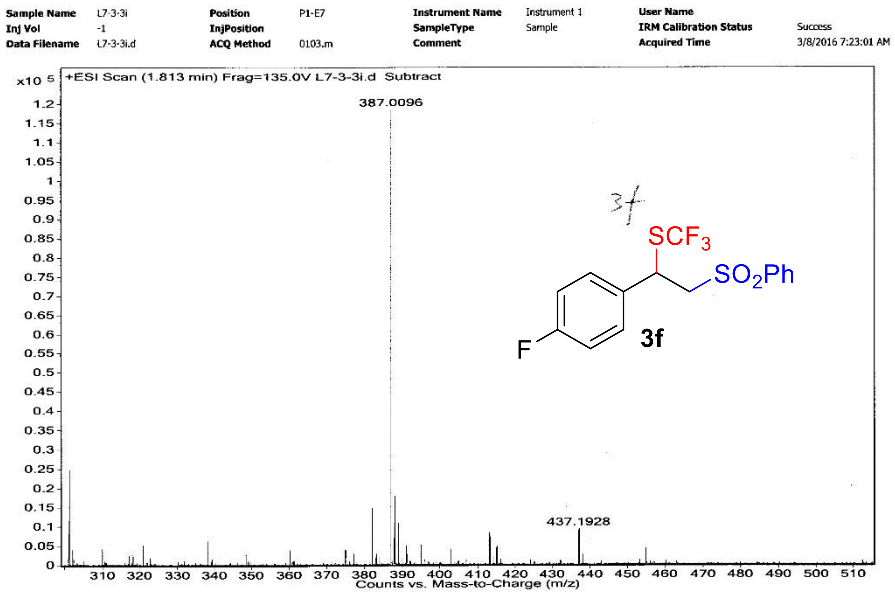

HRMS (ESI, m/z) calcd for $C_{15}H_{12}F_4O_2S_2$ $[M+Na]^+$ 387.0107, found 387.0096.

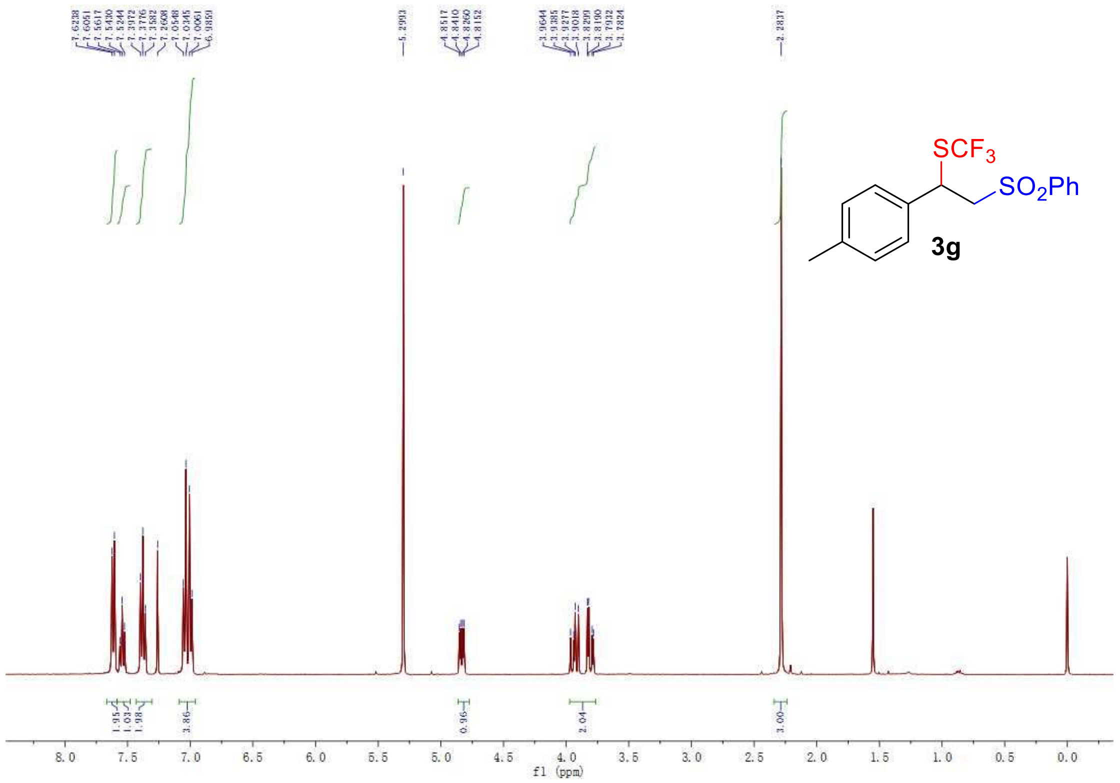
7.6238
7.6051
7.5617
7.5430
7.5244
7.3972
7.3776
7.3582
7.2608
7.0546
7.0345
7.0061
6.9859
5.2993
4.8517
4.8410
4.8260
4.8152
3.9644
3.9385
3.9277
3.9018
3.8299
3.8190
3.7932
3.7824
2.2837
SCF3
SO2Ph
3g
1.95
1.03
1.98
3.86
0.96
2.04
3.00
f1 (ppm)

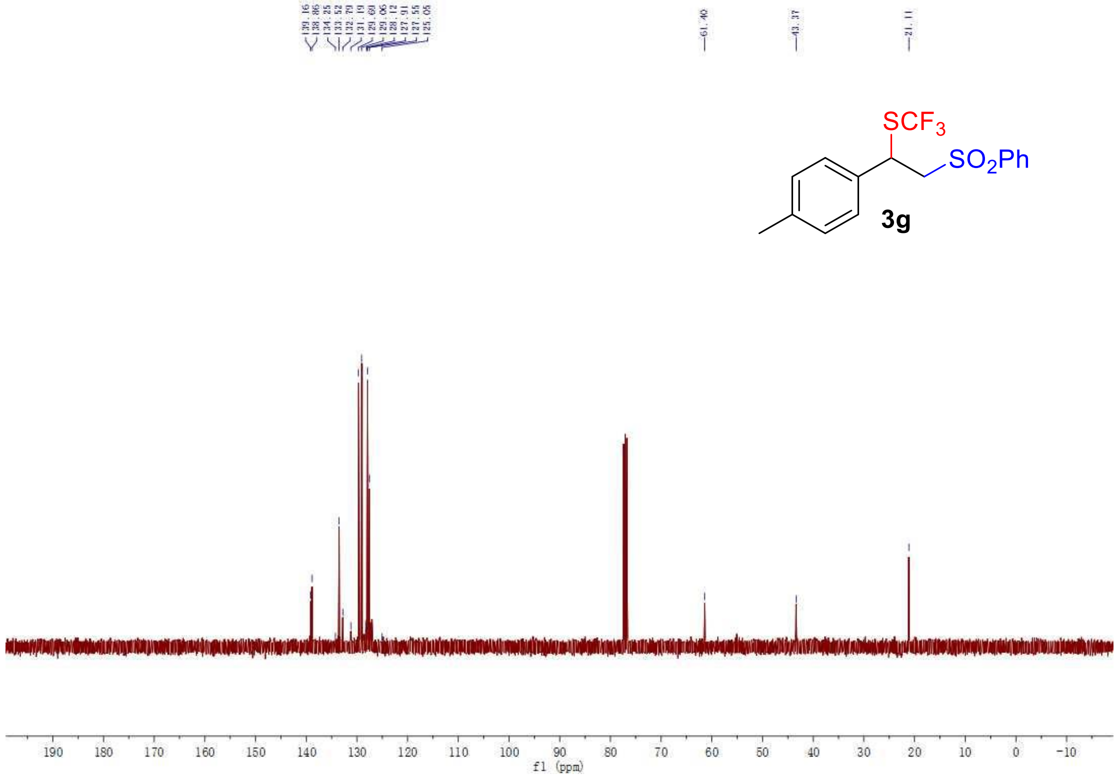
139.16
138.86
134.25
133.52
132.79
131.19
129.60
129.06
128.12
127.91
127.55
125.05
61.40
43.37
21.11
SCF3
SO2Ph
3g
f1 (ppm)

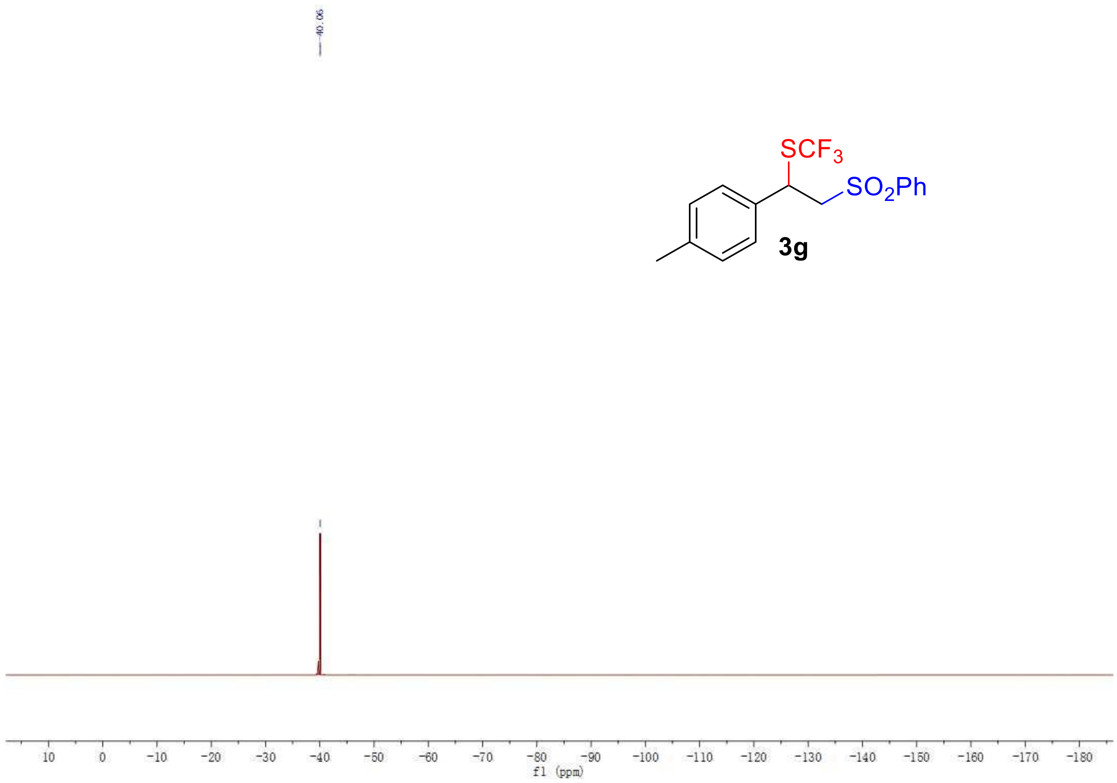
-40.06
10
0
-10
-20
-30
-40
-50
-60
-70
-80
-90
-100
-110
-120
-130
-140
-150
-160
-170
-180
f1 (ppm)
SCF3
SO2Ph

3g

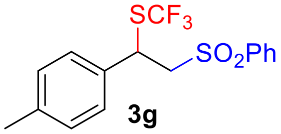

3g

Chemical Formula: $C_{16}H_{15}F_3O_2S_2$

Exact Mass: 360.0466

Molecular Weight: 360.4092

m/z: 360.0466 (100.0%), 361.0499 (17.3%), 362.0424 (9.0%), 361.0459 (1.6%), 363.0457 (1.6%), 362.0533 (1.4%)

Elemental Analysis: C, 53.32; H, 4.20; F, 15.81; O, 8.88; S, 17.79

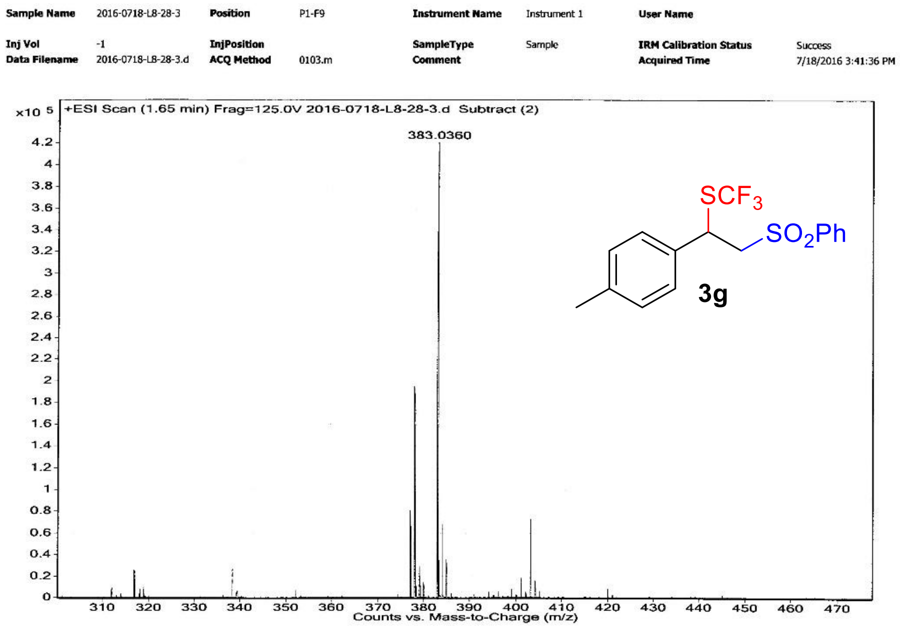

HRMS (ESI, m/z) calcd for $C_{16}H_{15}F_3O_2S_2$ $[M+Na]^+$ 383.0358, found 383.0360.

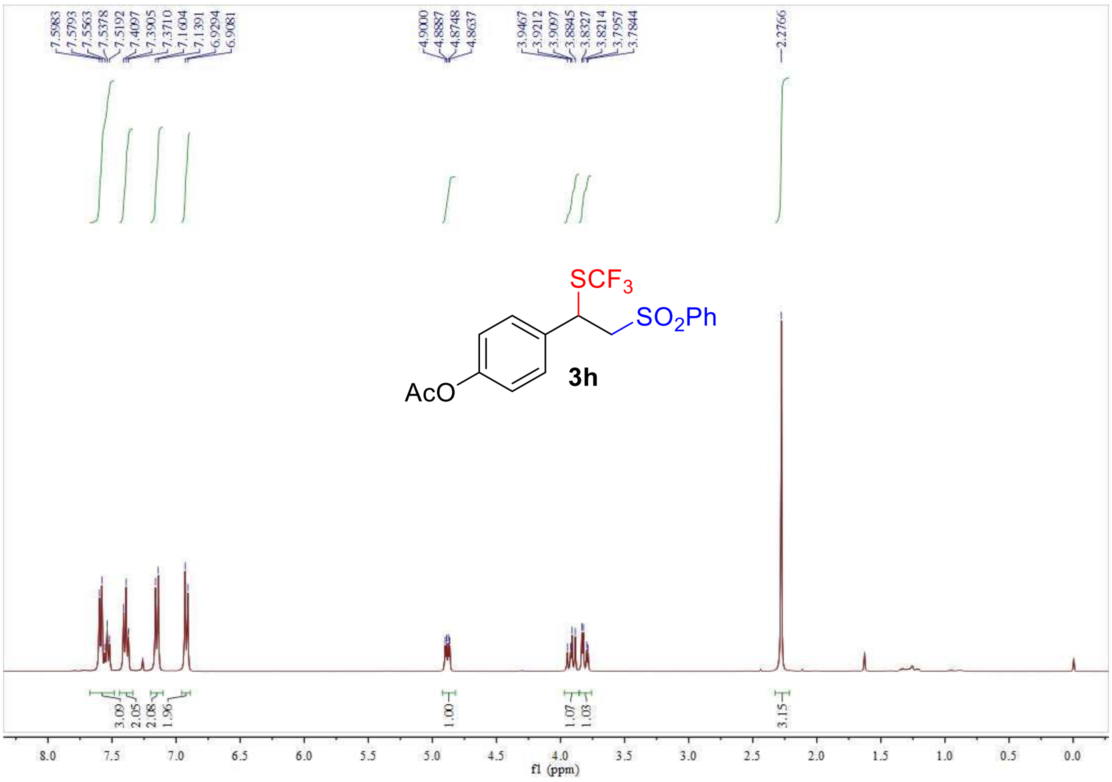
7.5983
7.5793
7.5563
7.5378
7.5192
7.4097
7.3905
7.3710
7.1604
7.1391
6.9294
6.9081
4.9000
4.8887
4.8748
4.8637
3.9467
3.9212
3.9097
3.8845
3.8327
3.8214
3.7957
3.7844
2.2766
SCF3
SO2Ph
AcO
3h
3.09
2.05
2.08
1.96
1.00
1.07
1.03
3.15
f1 (ppm)

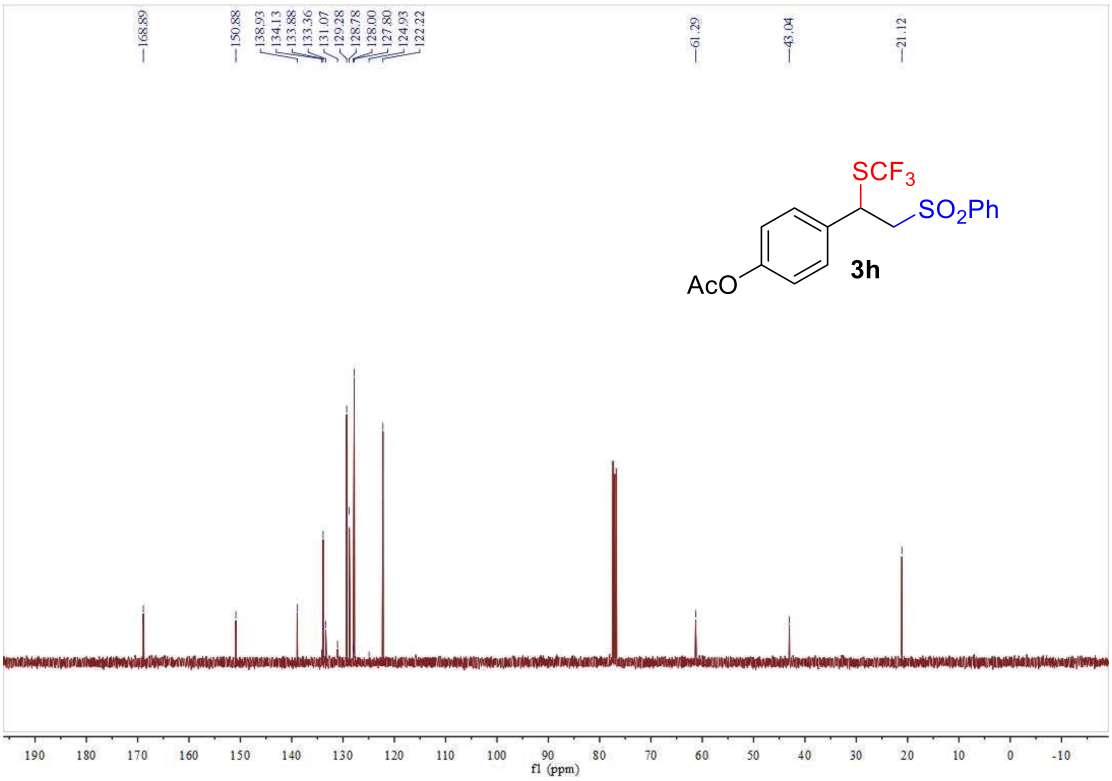
168.89
150.88
138.93
134.13
133.88
133.36
131.07
129.28
128.78
128.00
127.80
124.93
122.22
61.29
43.04
21.12
SCF3
SO2Ph
AcO
3h
f1 (ppm)

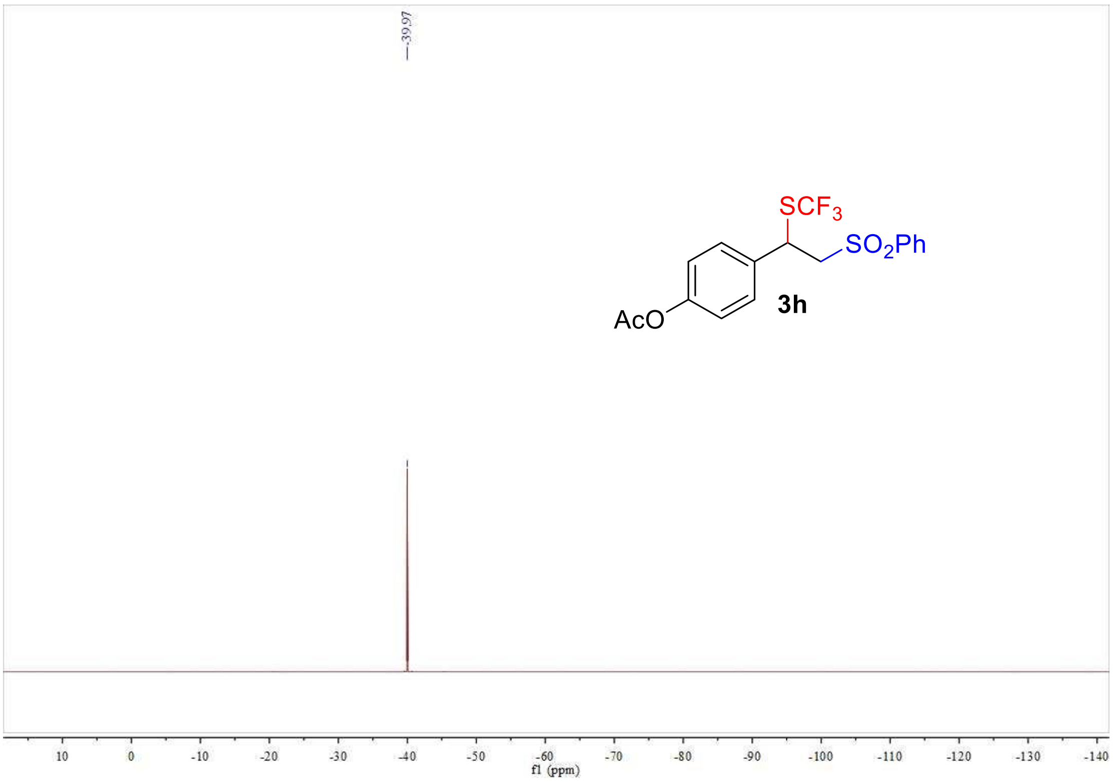

—39.97
SCF3
SO2Ph
AcO
3h
10
0
-10
-20
-30
-40
-50
-60
-70
-80
-90
-100
-110
-120
-130
-140
f1 (ppm)

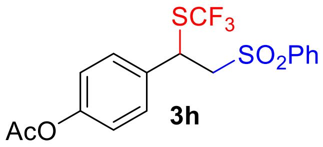

3h

Chemical Formula: $C_{17}H_{15}F_3O_4S_2$
Exact Mass: 404.0364
Molecular Weight: 404.4182
m/z: 404.0364 (100.0%), 405.0397 (18.4%), 406.0322 (9.0%), 407.0355 (1.7%), 405.0358 (1.6%), 406.0431 (1.6%)
Elemental Analysis: C, 50.49; H, 3.74; F, 14.09; O, 15.82; S, 15.85

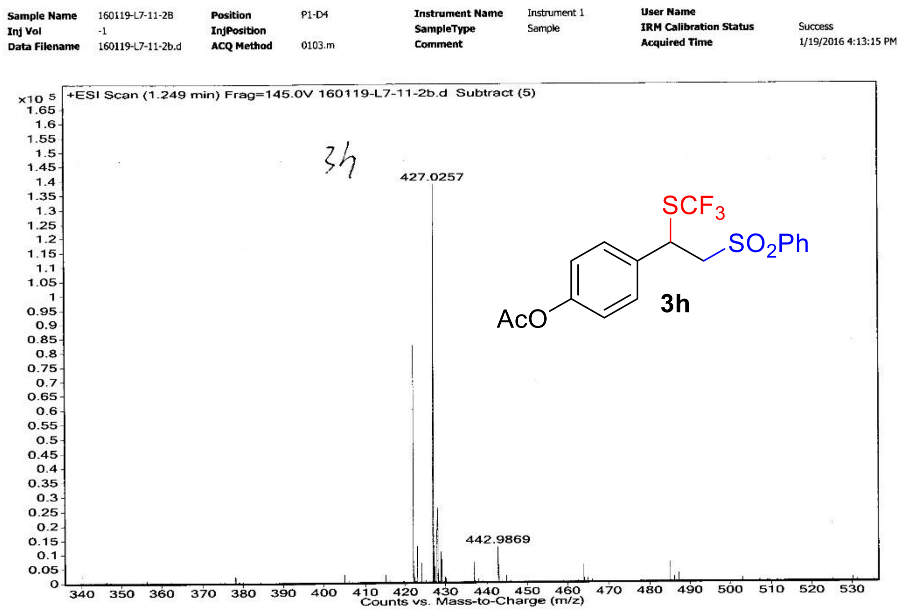

HRMS (ESI, m/z) calcd for $C_{17}H_{15}F_3O_4S_2$ $[M+Na]^+$ 427.0256, found 427.0257.

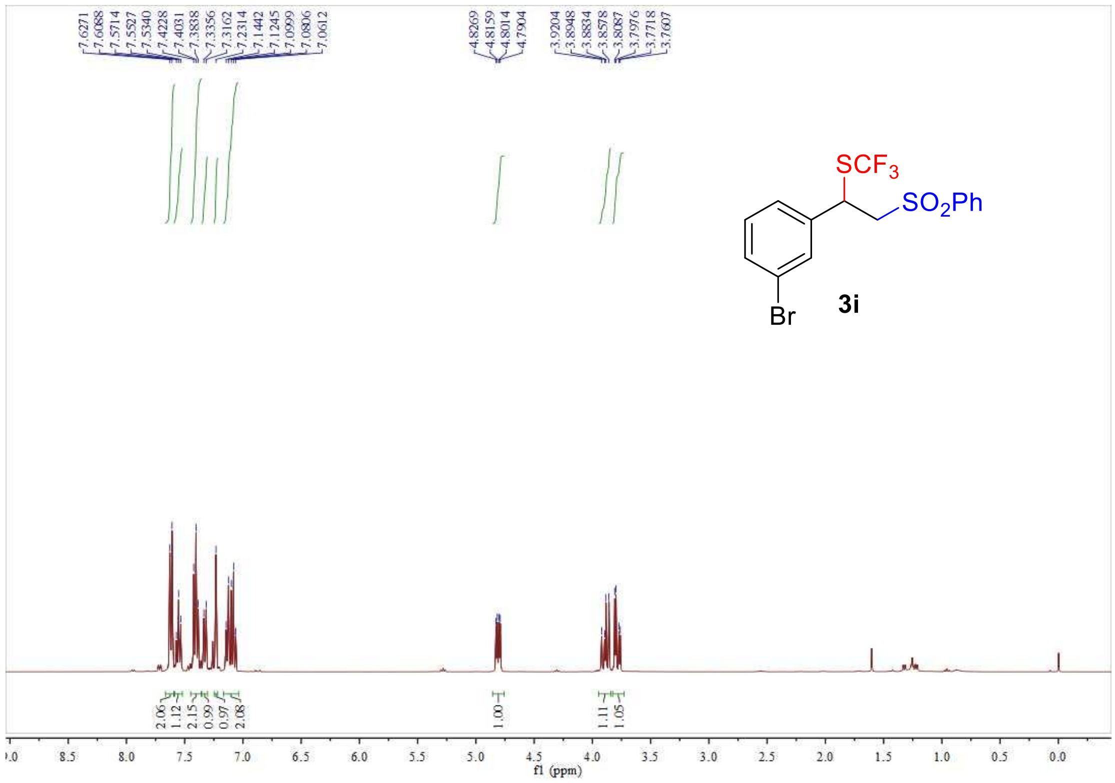

7.6271 7.6088 7.5714 7.5527 7.5340 7.4228 7.4031 7.3838 7.3356 7.3162 7.2314 7.1442 7.1245 7.0999 7.0806 7.0612
4.8269 4.8159 4.8014 4.7904
3.9204 3.8948 3.8834 3.8578 3.8087 3.7976 3.7718 3.7607
SCF3
SO2Ph
Br
3i
2.06 1.12 2.15 0.99 0.97 2.08
1.00
1.11 1.05
f1 (ppm)

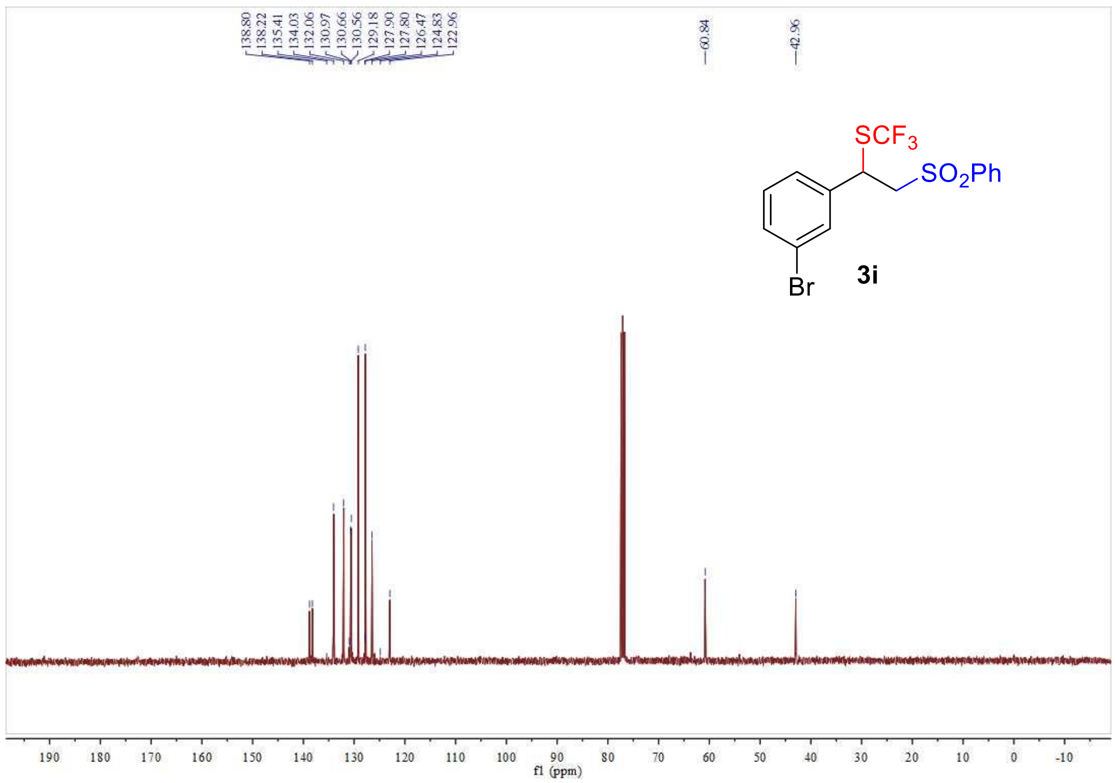

138.80 138.22 135.41 134.03 132.06 130.97 130.66 130.56 129.18 127.90 127.80 126.47 124.83 122.96
60.84
42.96
SCF3
SO2Ph
Br
3i
f1 (ppm)

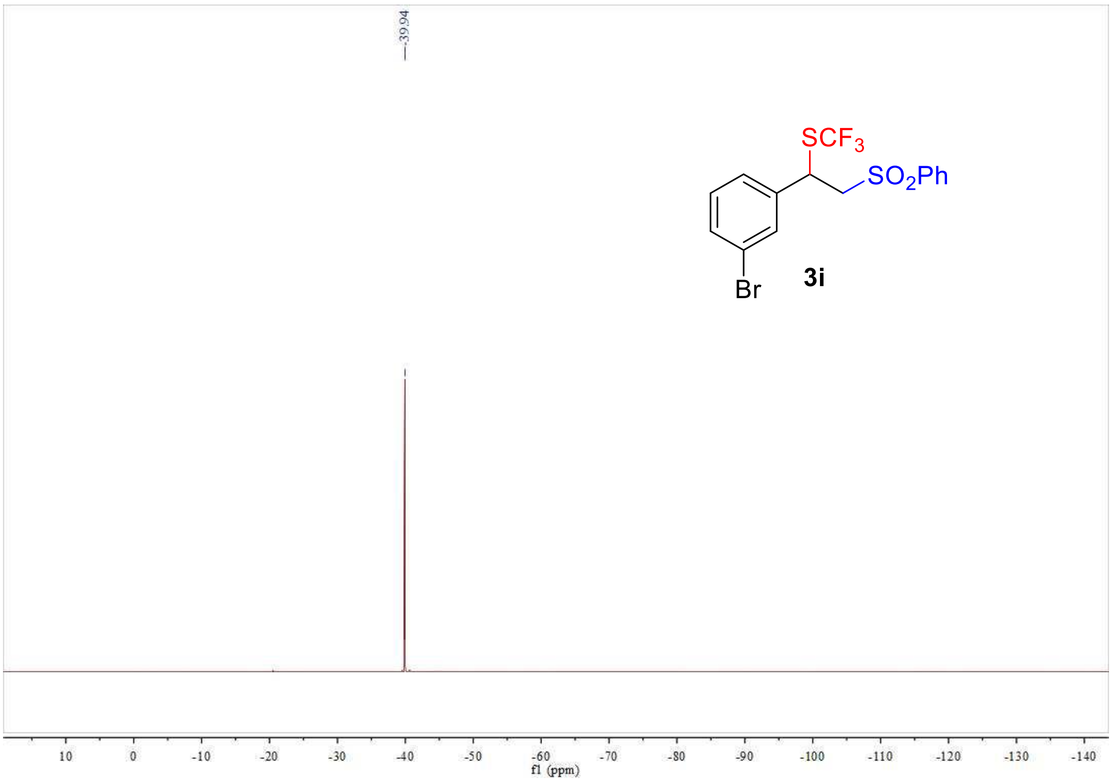

-39.94
SCF3
SO2Ph
Br
3i
10
0
-10
-20
-30
-40
-50
-60
-70
-80
-90
-100
-110
-120
-130
-140
f1 (ppm)

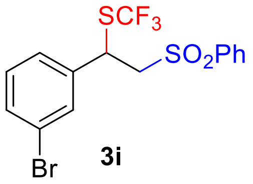

3i

Chemical Formula: $C_{15}H_{12}BrF_3O_2S_2$
Exact Mass: 423.9414
Molecular Weight: 425.2782
m/z: 423.9414 (100.0%), 425.9394 (97.3%), 424.9448 (16.2%), 426.9427 (15.8%), 425.9372 (9.0%), 427.9352 (8.8%), 424.9408 (1.6%), 426.9388 (1.6%), 426.9406 (1.5%), 428.9385 (1.4%), 425.9481 (1.2%), 427.9461 (1.2%)
Elemental Analysis: C, 42.36; H, 2.84; Br, 18.79; F, 13.40; O, 7.52; S, 15.08

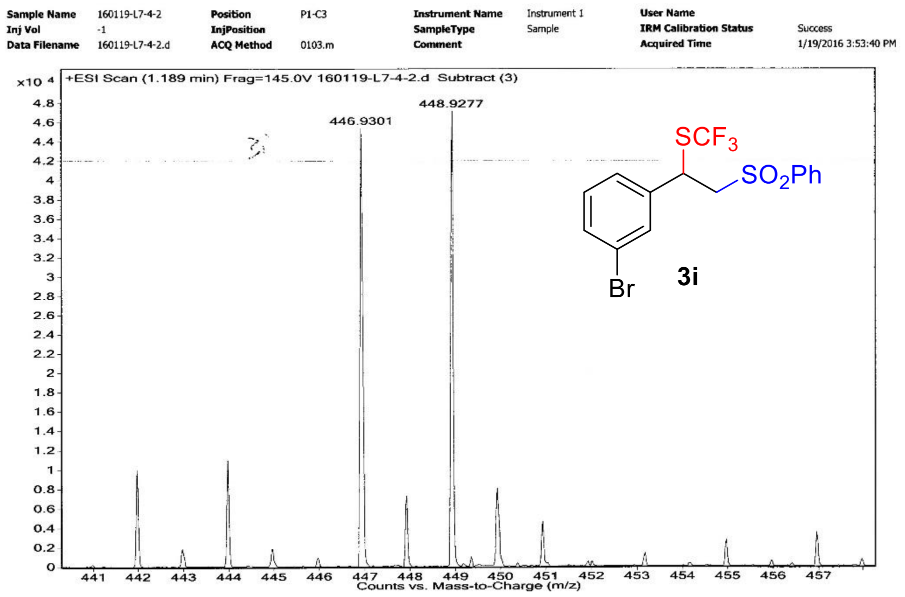

HRMS (ESI, m/z) calcd for $C_{15}H_{12}BrF_3O_2S_2$ $[M+Na]^+$ 446.9306, found 446.9301.

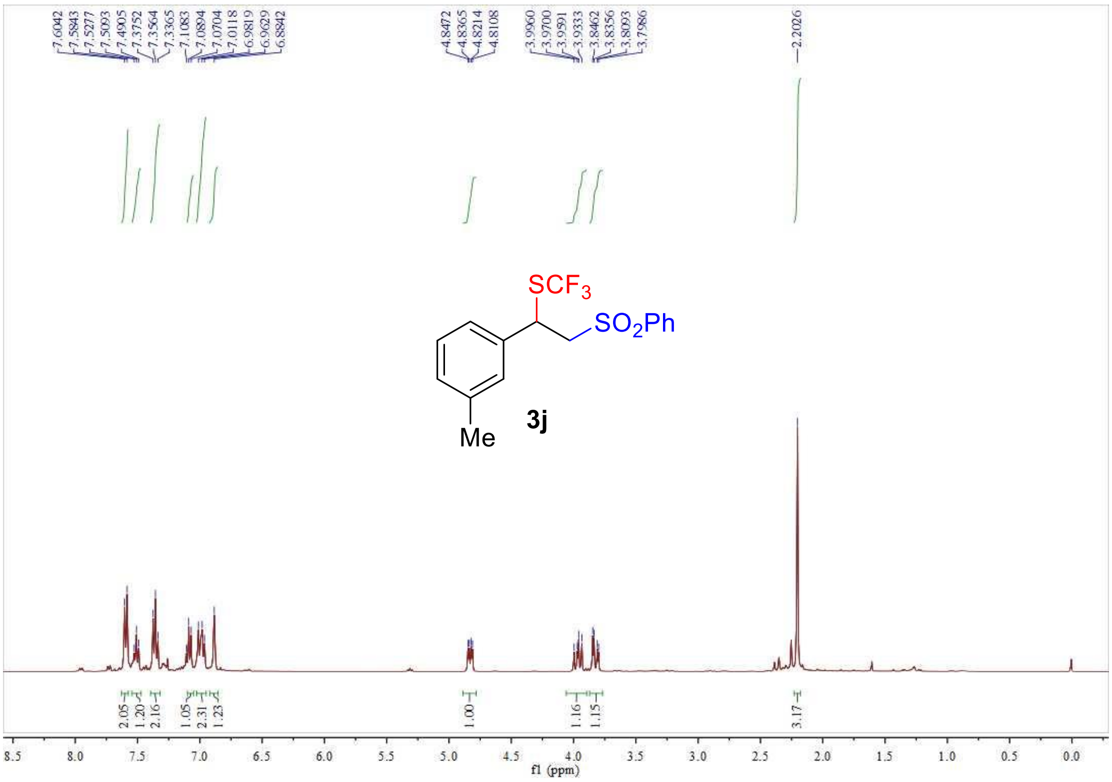
7.6042
7.5843
7.5277
7.5093
7.4905
7.3752
7.3564
7.3365
7.1083
7.0894
7.0704
7.0118
6.9819
6.9629
6.8842
4.8472
4.8365
4.8214
4.8108
3.9960
3.9700
3.9591
3.9333
3.8462
3.8356
3.8093
3.7986
2.2026
SCF3
SO2Ph
Me
3j
2.05
1.20
2.16
1.05
2.31
1.23
1.00
1.16
1.15
3.17
f1 (ppm)

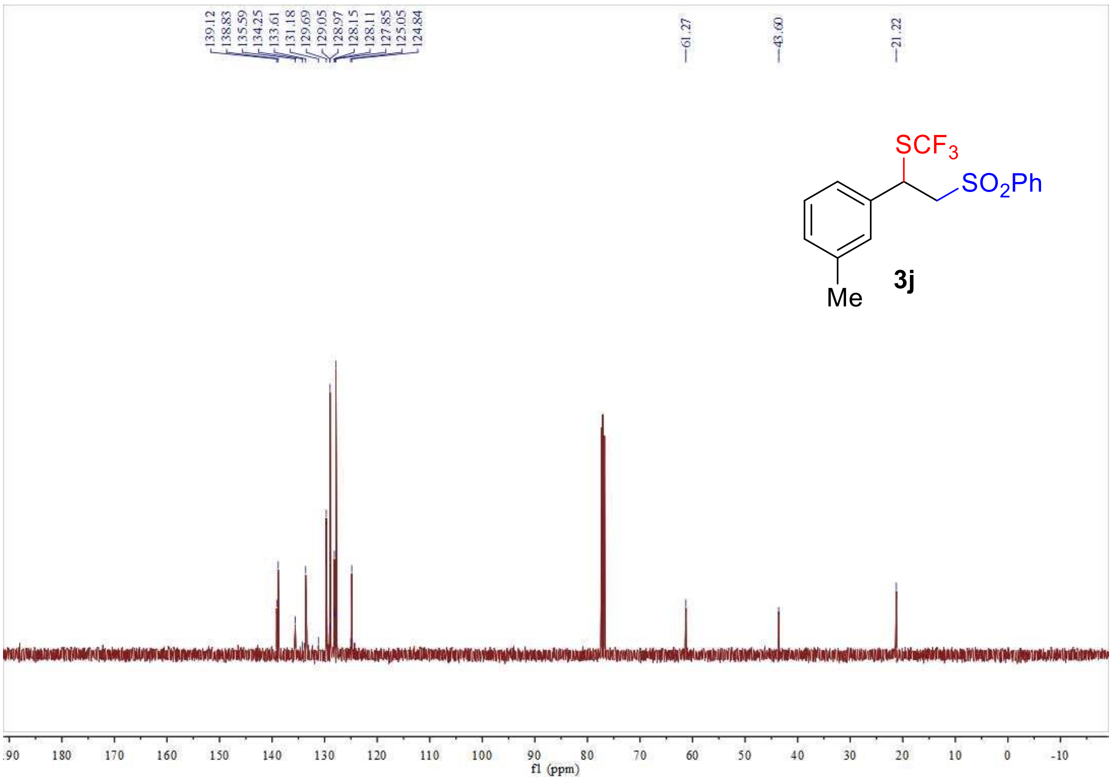
139.12
138.83
135.59
134.25
133.61
131.18
129.69
129.05
128.97
128.15
128.11
127.85
125.05
124.84
61.27
43.60
21.22
SCF3
SO2Ph
Me
3j
f1 (ppm)

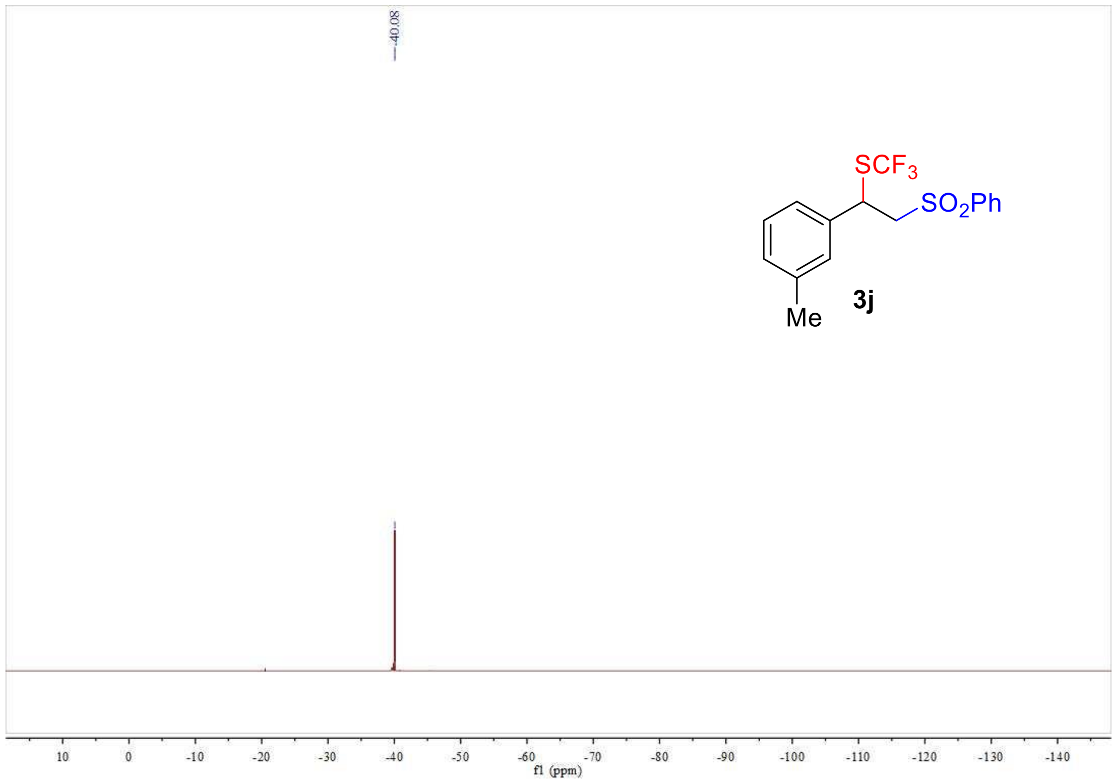

—40.08
SCF3
SO2Ph
Me
3j
10
0
-10
-20
-30
-40
-50
-60
-70
-80
-90
-100
-110
-120
-130
-140
f1 (ppm)

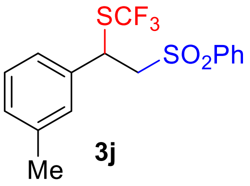

3j

Chemical Formula: $C_{16}H_{15}F_3O_2S_2$
Exact Mass: 360.0466
Molecular Weight: 360.4092
m/z: 360.0466 (100.0%), 361.0499 (17.3%), 362.0424 (9.0%), 361.0459 (1.6%), 363.0457 (1.6%), 362.0533 (1.4%)
Elemental Analysis: C, 53.32; H, 4.20; F, 15.81; O, 8.88; S, 17.79

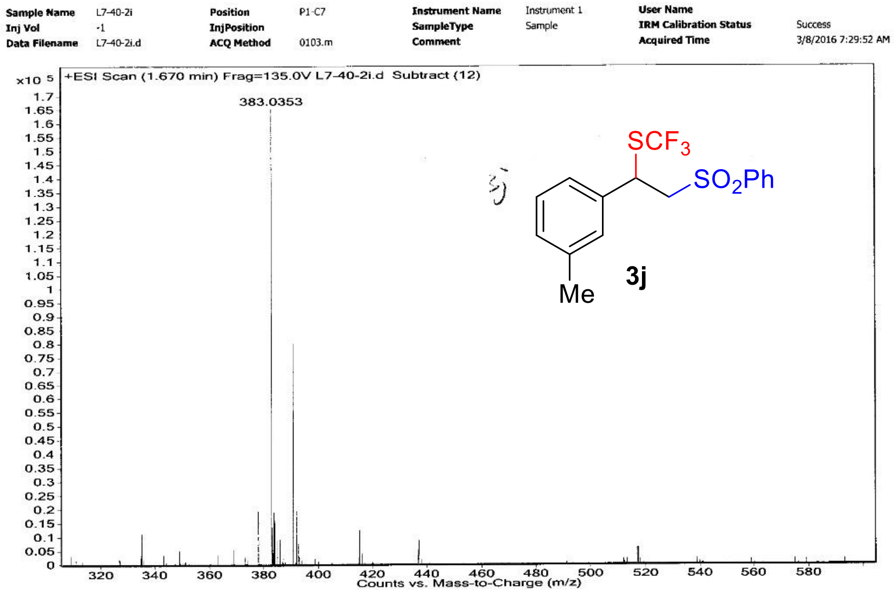

HRMS (ESI, m/z) calcd for $C_{16}H_{15}F_3O_2S_2$ $[M+Na]^+$ 383.0358, found 383.0353.

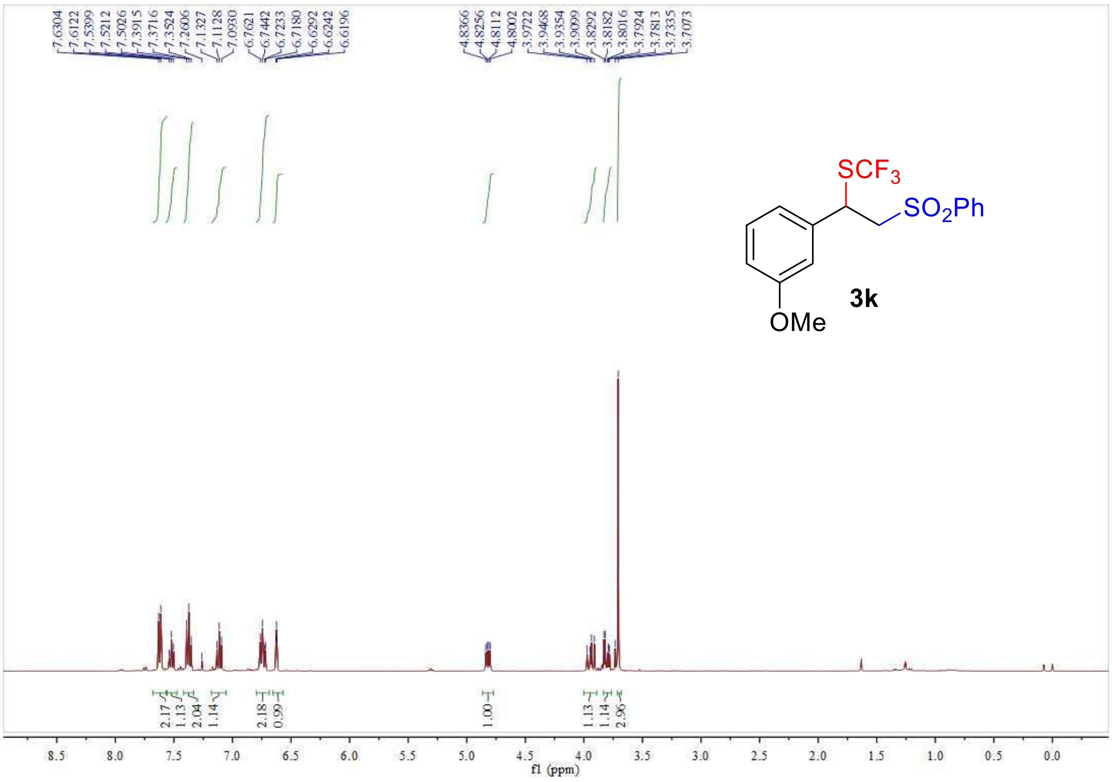
SCF3
SO2Ph
OMe
3k
f1 (ppm)

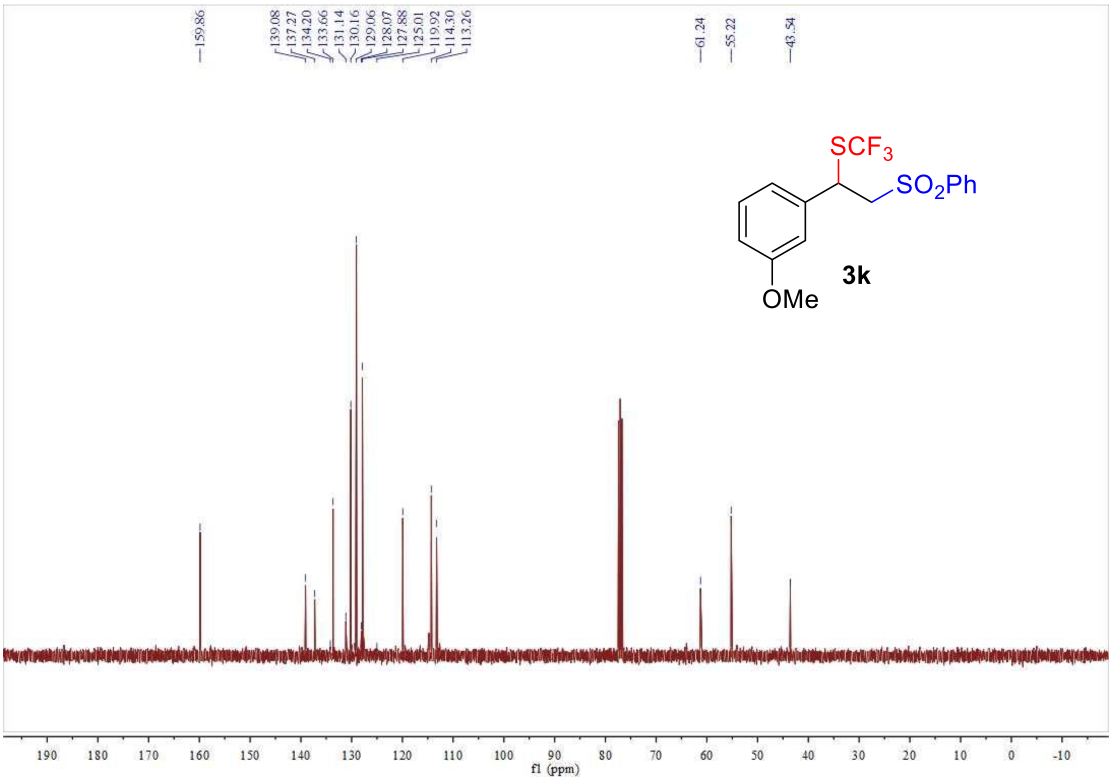
SCF3
SO2Ph
OMe
3k
f1 (ppm)

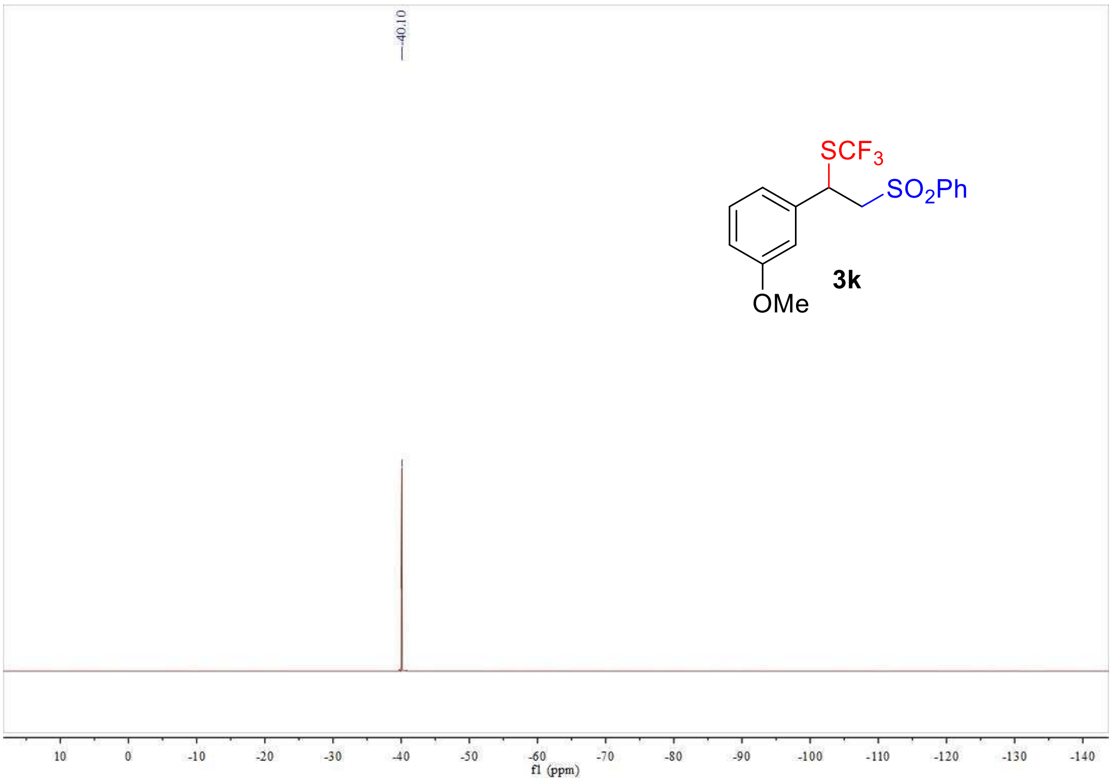
-40.10
SCF3
SO2Ph
OMe
3k
10 0 -10 -20 -30 -40 -50 -60 -70 -80 -90 -100 -110 -120 -130 -140
f1 (ppm)

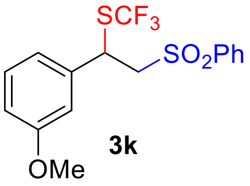

3k

Chemical Formula: $C_{16}H_{15}F_3O_3S_2$

Exact Mass: 376.0415

Molecular Weight: 376.4082

m/z: 376.0415 (100.0%), 377.0448 (17.3%), 378.0373 (9.0%), 377.0409 (1.6%), 379.0406 (1.6%), 378.0482 (1.4%)

Elemental Analysis: C, 51.06; H, 4.02; F, 15.14; O, 12.75; S, 17.03

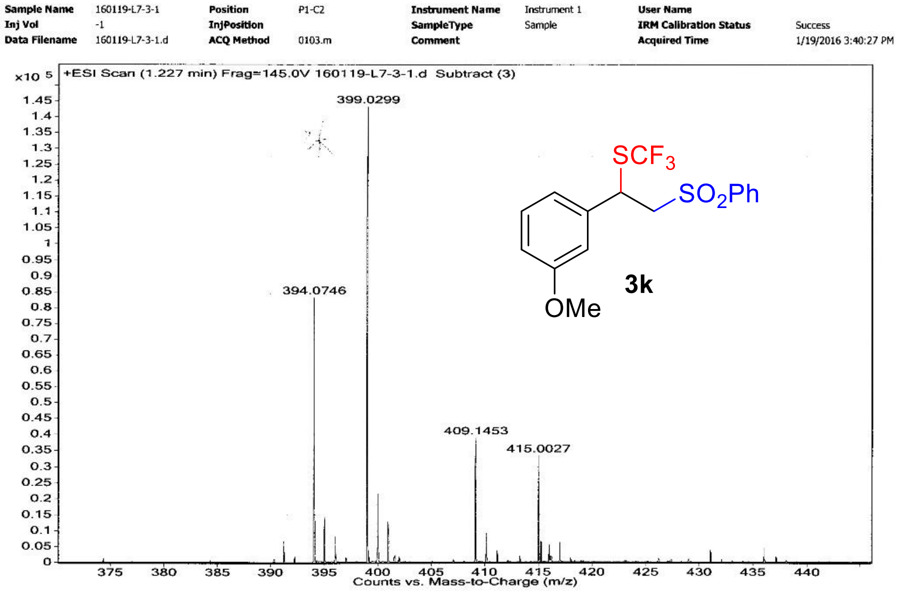

HRMS (ESI, m/z) calcd for $C_{16}H_{15}F_3O_3S_2$ $[M+Na]^+$ 399.0307, found 399.0299.

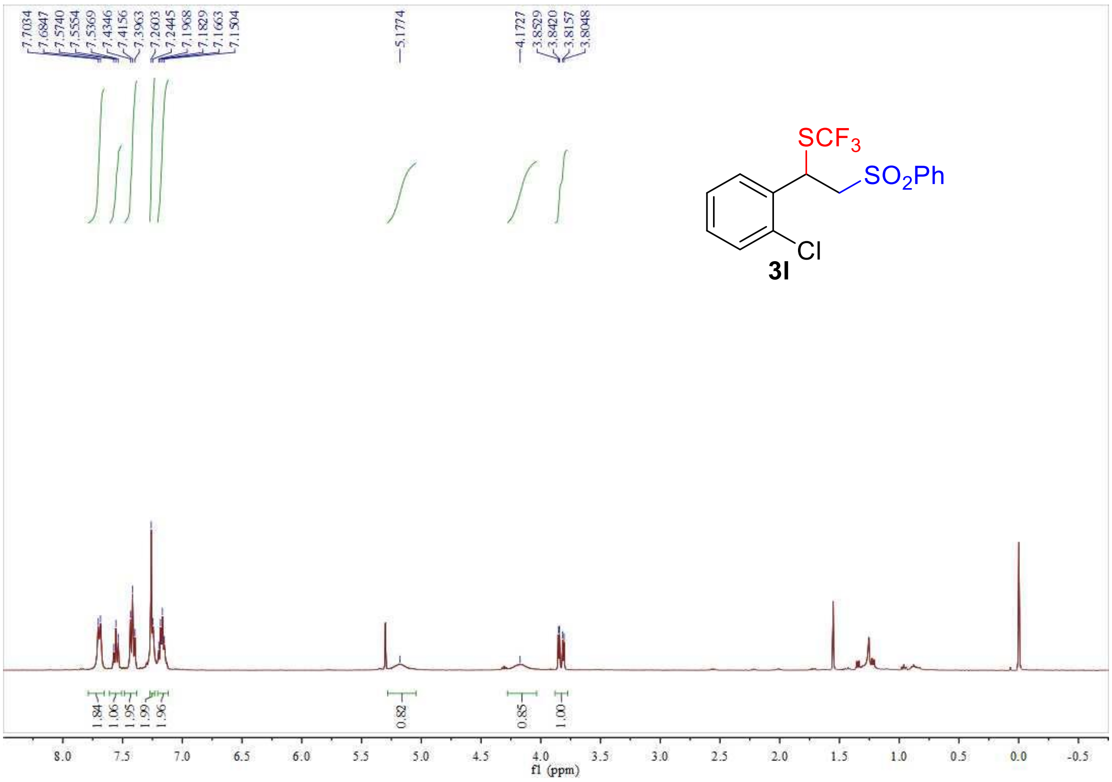
7.7034
7.6847
7.5740
7.5554
7.5369
7.4346
7.4156
7.3963
7.2603
7.2445
7.1968
7.1829
7.1663
7.1504
5.1774
4.1727
3.8529
3.8420
3.8157
3.8048
SCF3
SO2Ph
Cl
3l
1.84
1.06
1.95
1.99
1.96
0.82
0.85
1.00
8.0 7.5 7.0 6.5 6.0 5.5 5.0 4.5 4.0 3.5 3.0 2.5 2.0 1.5 1.0 0.5 0.0 -0.5
f1 (ppm)

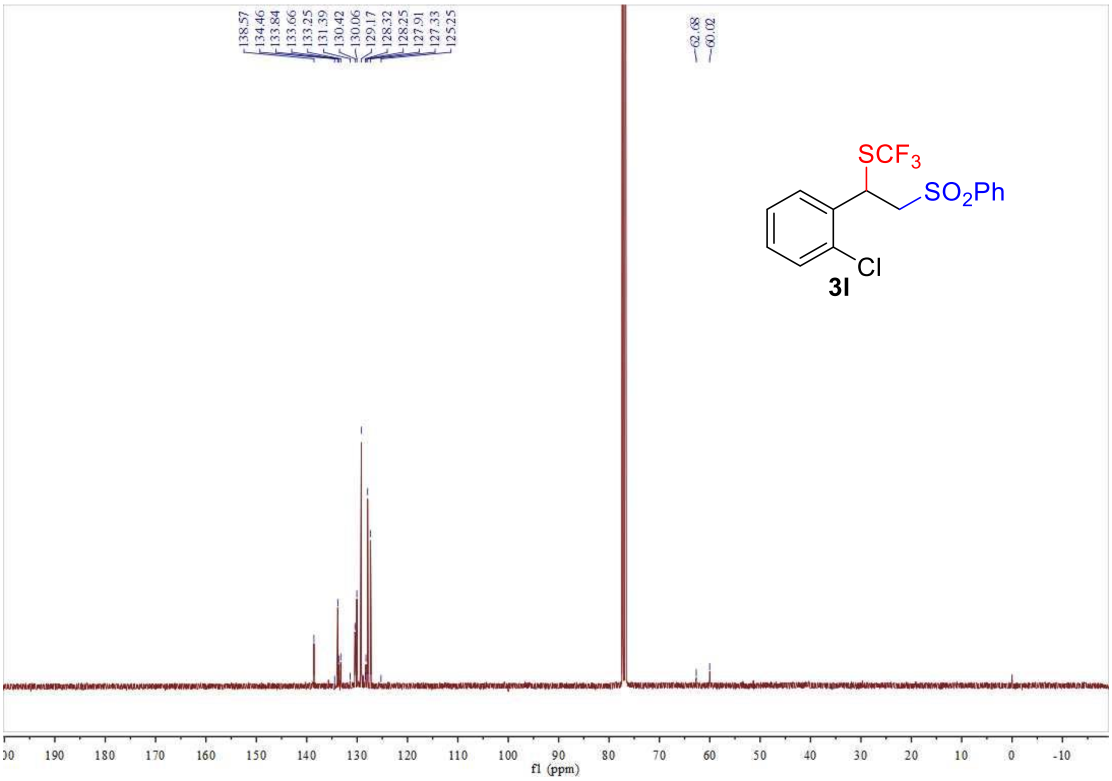
138.57
134.46
133.84
133.66
133.25
131.39
130.42
130.06
129.17
128.32
128.25
127.91
127.33
125.25
62.68
60.02
SCF3
SO2Ph
Cl
3l
190 180 170 160 150 140 130 120 110 100 90 80 70 60 50 40 30 20 10 0 -10
f1 (ppm)

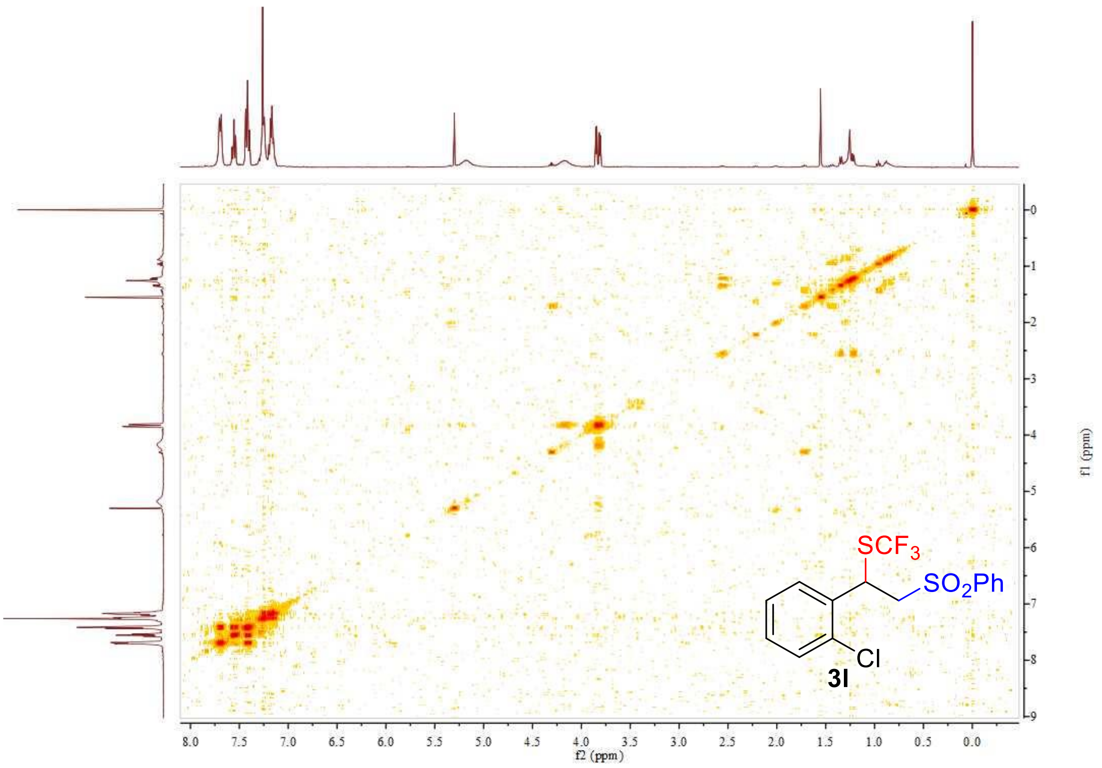
SCF3
SO2Ph
Cl
3l
f2 (ppm)
f1 (ppm)

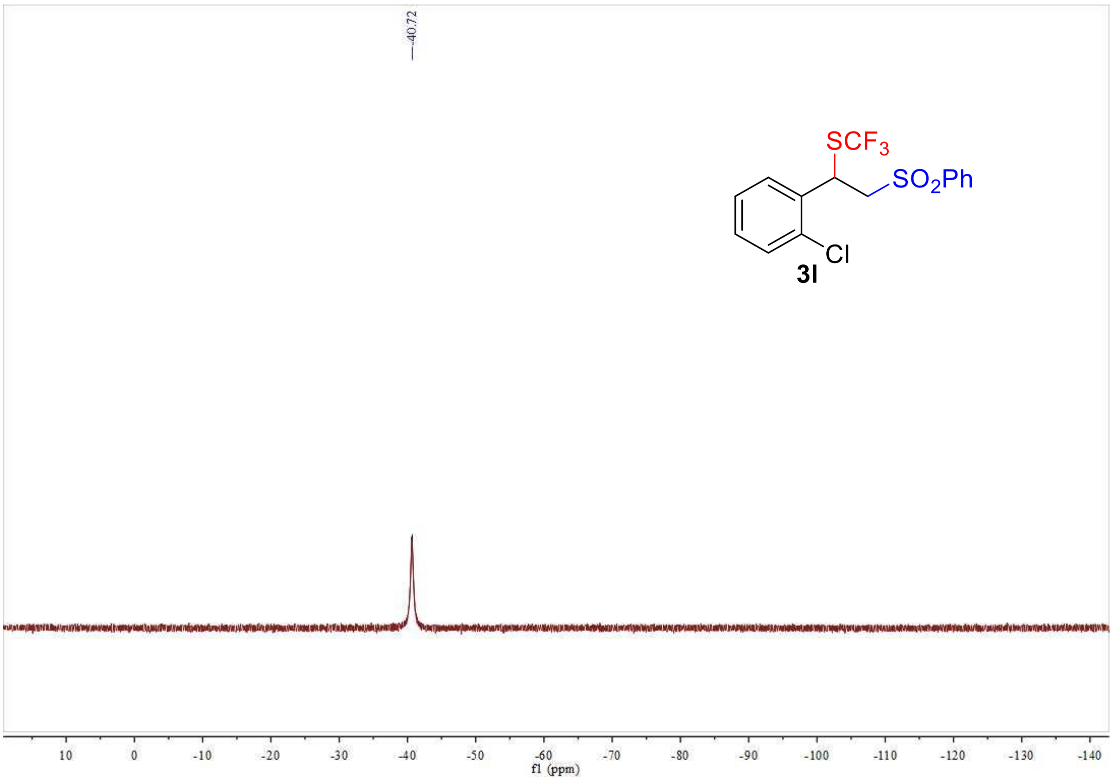
—40.72
SCF3
SO2Ph
Cl
3l
f1 (ppm)

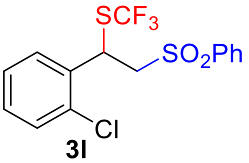

3l

Chemical Formula: $C_{15}H_{12}ClF_3O_2S_2$
Exact Mass: 379.9919
Molecular Weight: 380.8242
m/z: 379.9919 (100.0%), 381.9890 (32.0%), 380.9953 (16.2%), 381.9877 (9.0%), 382.9923 (5.2%), 383.9848 (2.9%), 380.9913 (1.6%), 382.9911 (1.5%), 381.9986 (1.2%)
Elemental Analysis: C, 47.31; H, 3.18; Cl, 9.31; F, 14.97; O, 8.40; S, 16.84

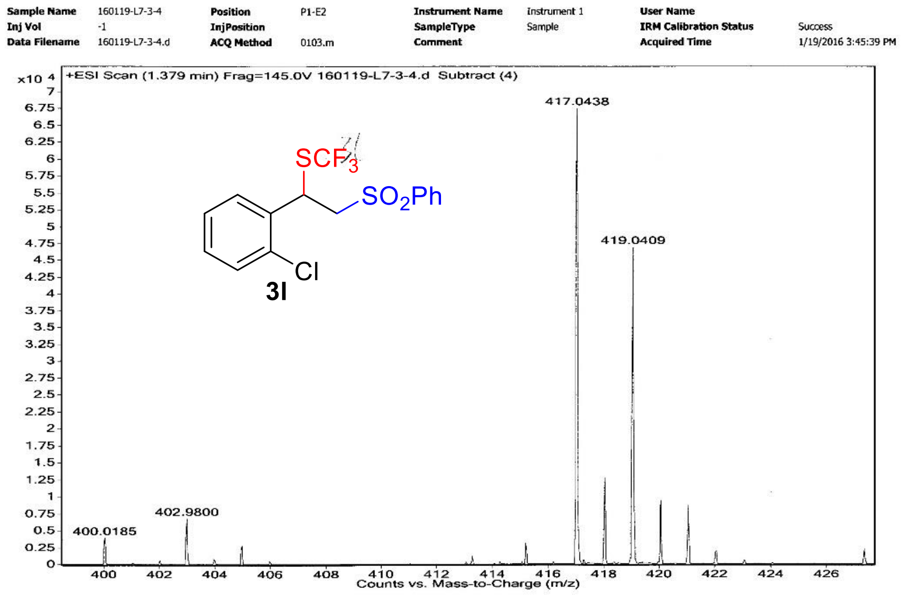

HRMS (ESI, m/z) calcd for $C_{15}H_{12}ClF_3O_2S_2$ $[M+Na]^+$ 402.9812, found 402.9800.

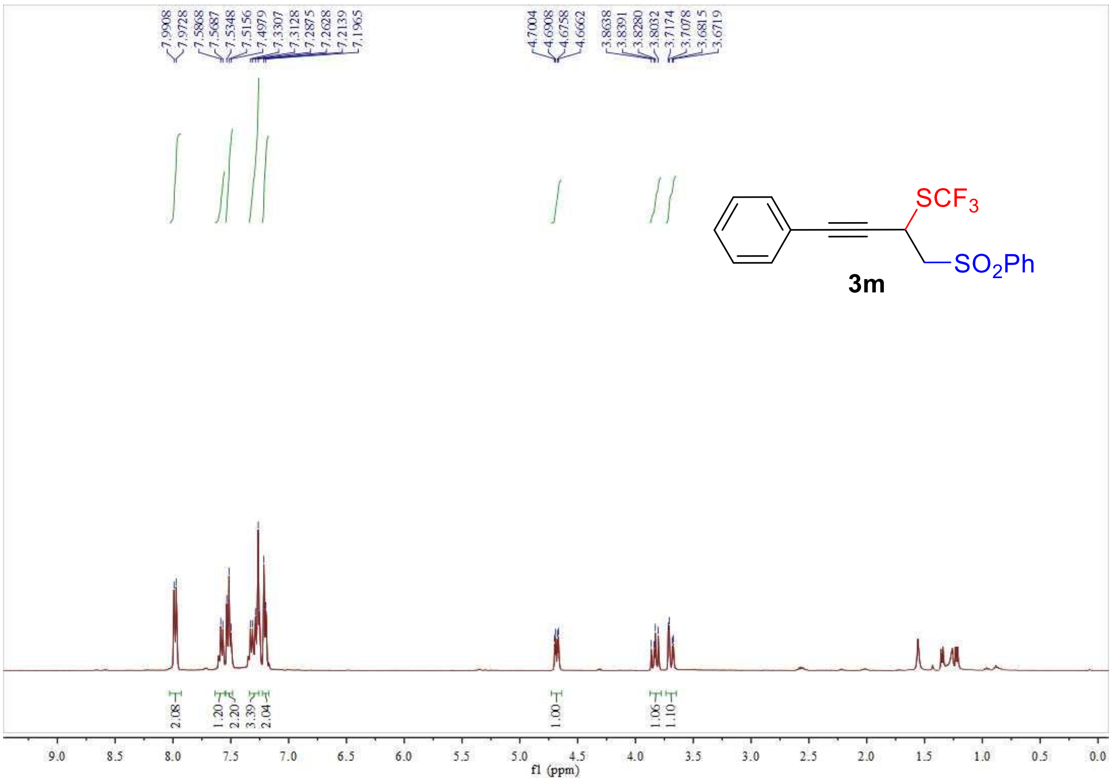
7.9908
7.9728
7.5868
7.5687
7.5348
7.5156
7.4979
7.3307
7.3128
7.2875
7.2628
7.2139
7.1965
4.7004
4.6908
4.6758
4.6662
3.8638
3.8391
3.8280
3.8032
3.7174
3.7078
3.6815
3.6719
SCF3
SO2Ph
3m
2.08
1.20
2.20
3.39
2.04
1.00
1.06
1.10
f1 (ppm)

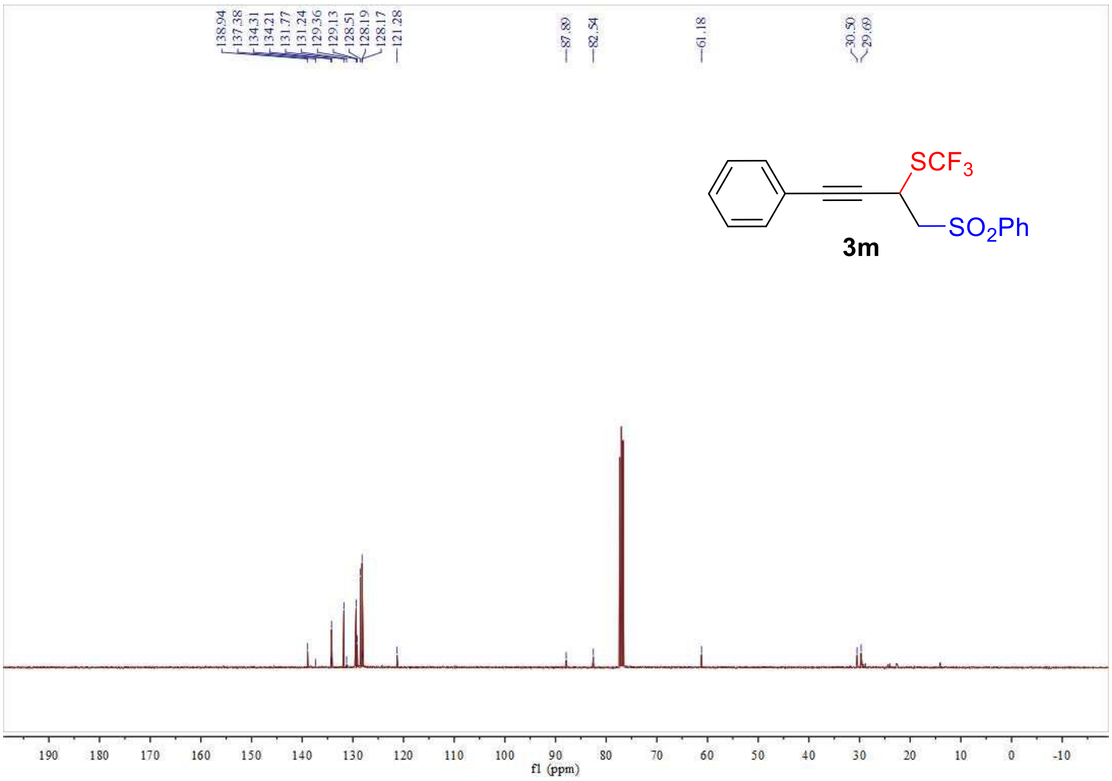
138.94
137.38
134.31
134.21
131.77
131.24
129.36
129.13
128.51
128.19
128.17
121.28
87.89
82.54
61.18
30.50
29.69
SCF3
SO2Ph
3m
f1 (ppm)

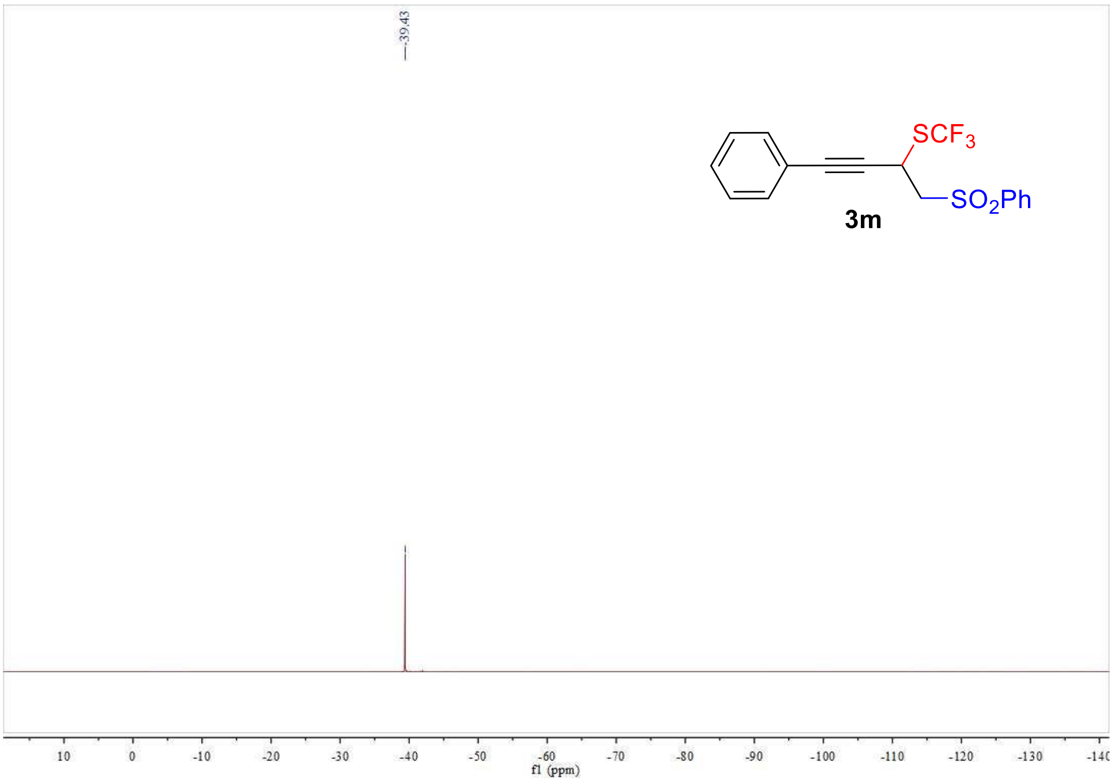
-39.43
SCF3
SO2Ph
3m
10 0 -10 -20 -30 -40 -50 -60 -70 -80 -90 -100 -110 -120 -130 -140
f1 (ppm)

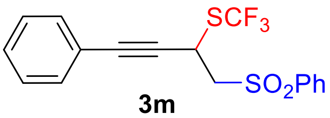

3m

Chemical Formula: $C_{17}H_{13}F_3O_2S_2$
Exact Mass: 370.0309
Molecular Weight: 370.4042
m/z: 370.0309 (100.0%), 371.0343 (18.4%), 372.0267 (9.0%), 373.0301 (1.7%), 371.0303 (1.6%), 372.0376 (1.6%)
Elemental Analysis: C, 55.13; H, 3.54; F, 15.39; O, 8.64; S, 17.31

**Sample Name**	2016-0309-L7-50-1	**Position**	P1-C9	**Instrument Name**	Instrument 1	**User Name**	
**Inj Vol**	-1	**InjPosition**		**SampleType**	Sample	**IRM Calibration Status**	Success
**Data Filename**	2016-0309-L7-50-1.d	**ACQ Method**	0103.m	**Comment**		**Acquired Time**	3/6/2016 2:55:14 PM

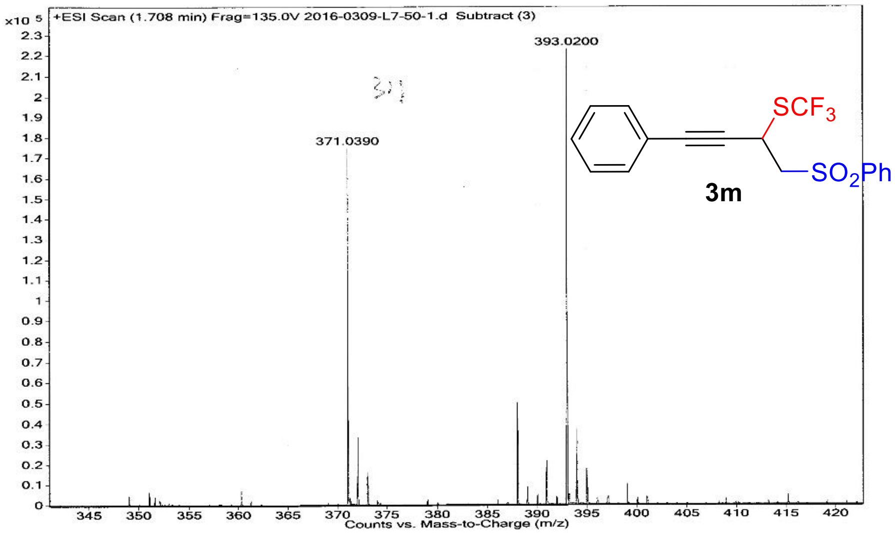

HRMS (ESI, m/z) calcd for $C_{17}H_{13}F_3O_2S_2$ $[M+Na]^+$ 393.0201, found 393.0200.

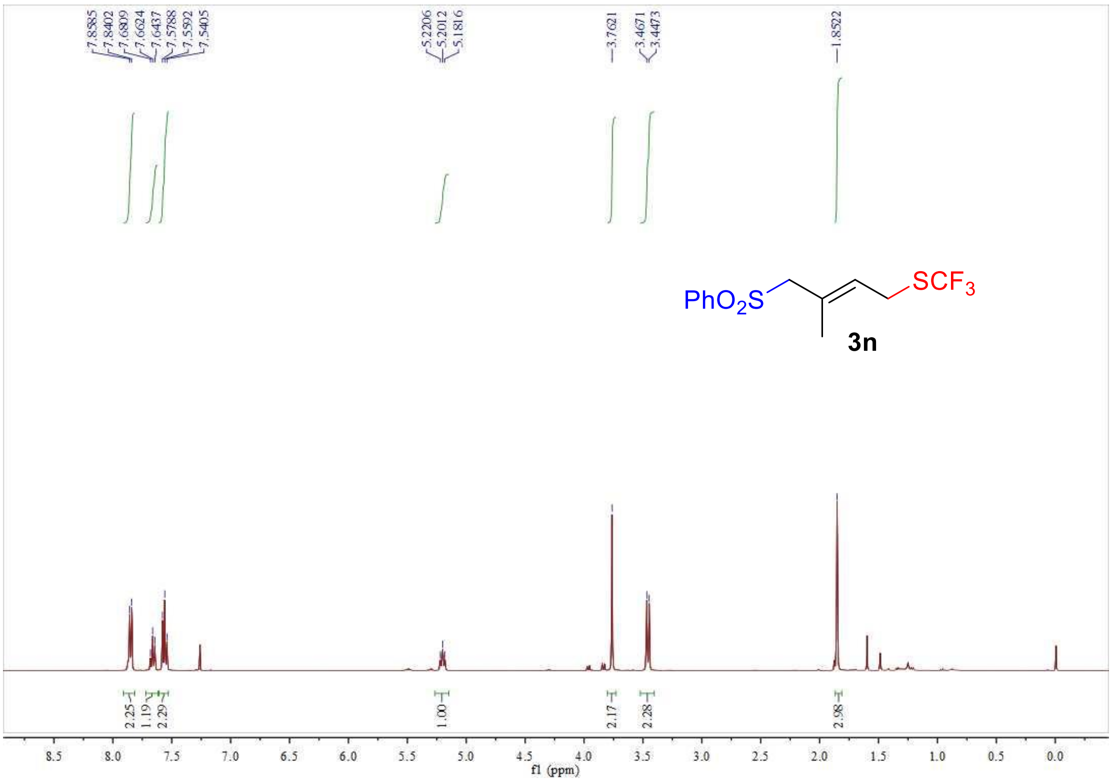
7.8585
7.8402
7.6809
7.6624
7.6437
7.5788
7.5592
7.5405
5.2206
5.2012
5.1816
3.7621
3.4671
3.4473
1.8522
PhO2S
SCF3
3n
2.25
1.19
2.29
1.00
2.17
2.28
2.98
fl (ppm)

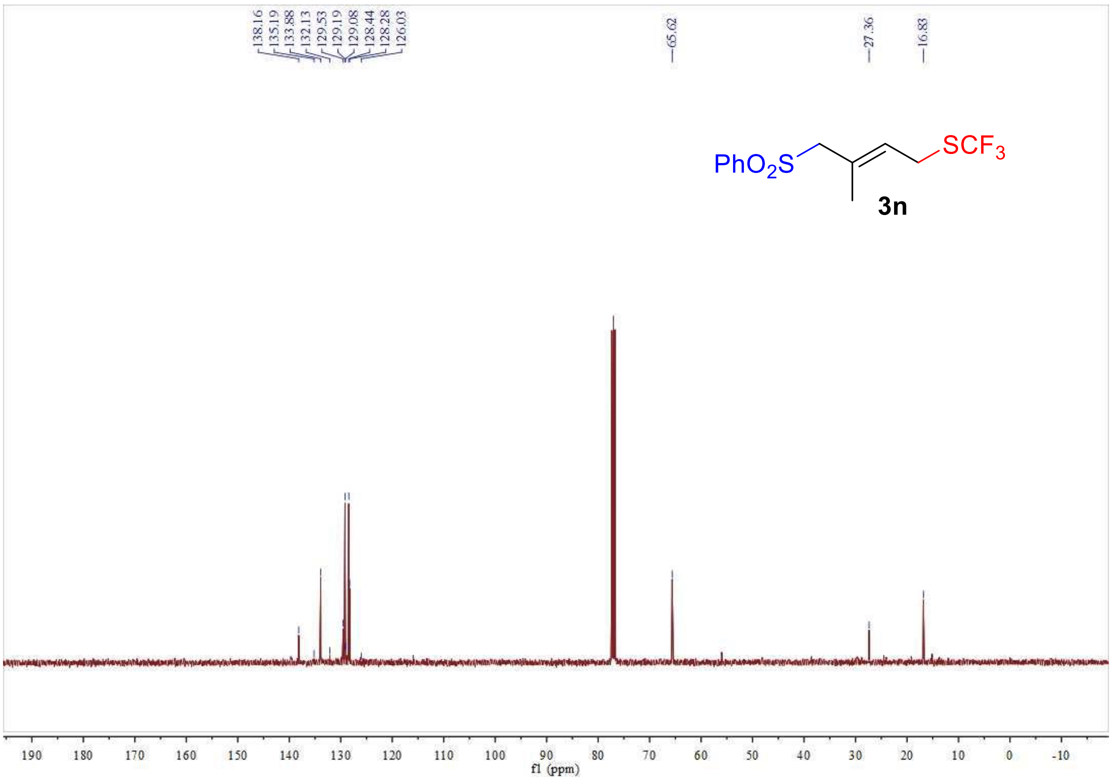
138.16
135.19
133.88
132.13
129.53
129.19
129.08
128.44
128.28
126.03
65.62
27.36
16.83
PhO2S
SCF3
3n
fl (ppm)

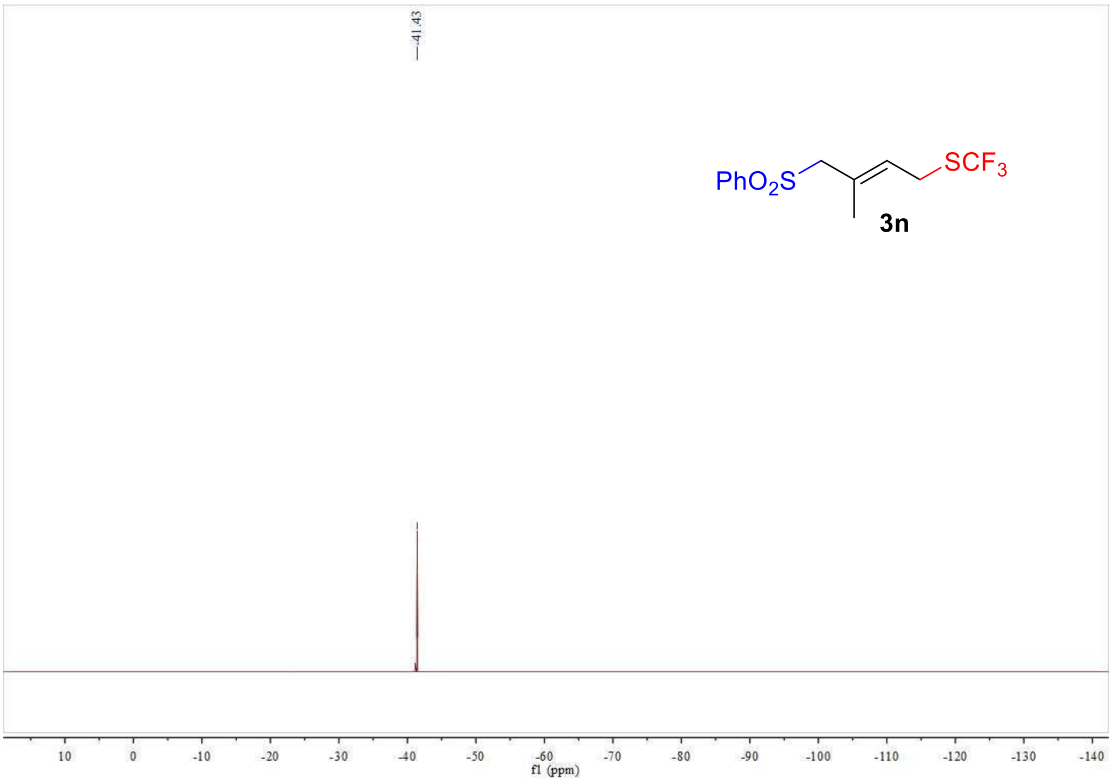

—41.43
PhO2S
SCF3
3n
10
0
-10
-20
-30
-40
-50
-60
-70
-80
-90
-100
-110
-120
-130
-140
f1 (ppm)

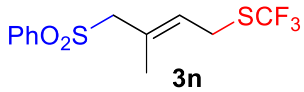

3n

Chemical Formula: $C_{12}H_{13}F_3O_2S_2$
Exact Mass: 310.0309
Molecular Weight: 310.3492
m/z: 310.0309 (100.0%), 311.0343 (13.0%), 312.0267 (4.5%), 312.0267 (4.5%)
Elemental Analysis: C, 46.44; H, 4.22; F, 18.36; O, 10.31; S, 20.66

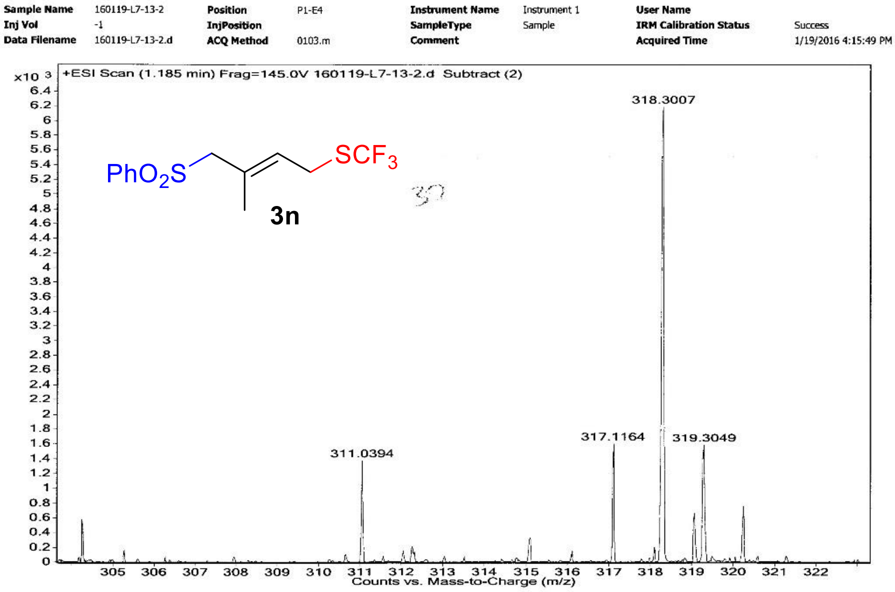

HRMS (ESI, m/z) calcd for $C_{12}H_{13}F_3O_2S_2$ $[M+H]^+$ 311.0382, found 311.0394.

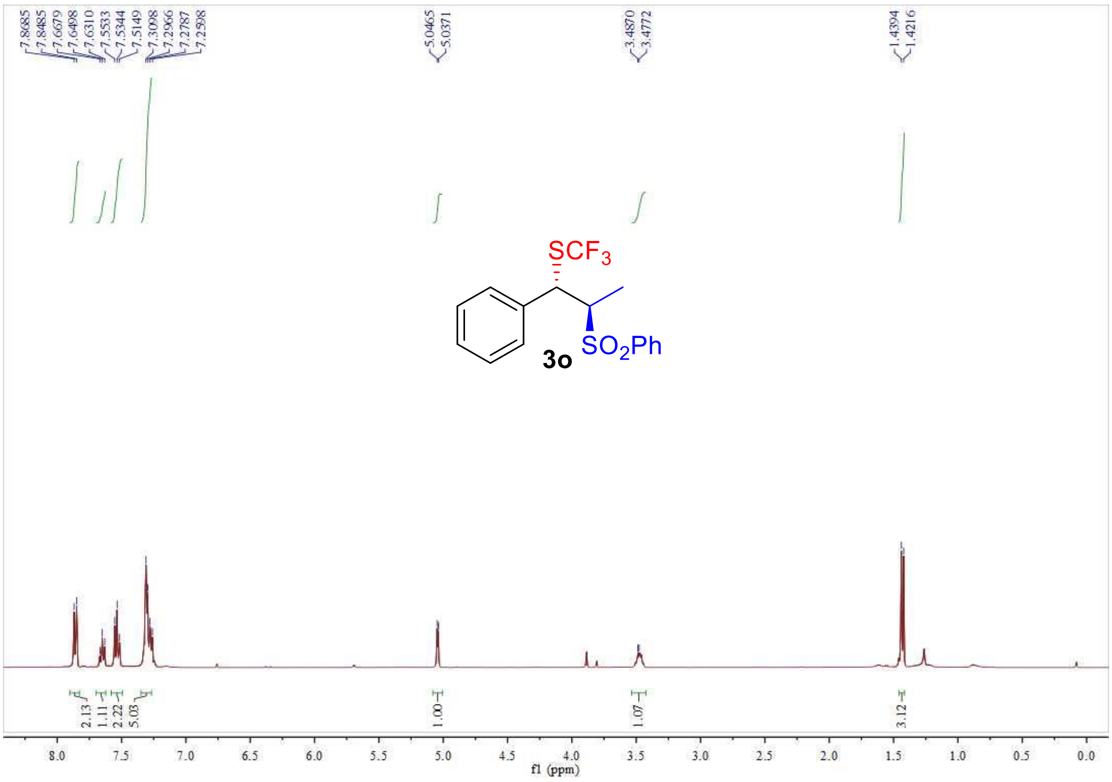
7.8685
7.8485
7.6679
7.6498
7.6310
7.5533
7.5344
7.5149
7.3098
7.2966
7.2787
7.2598
5.0465
5.0371
3.4870
3.4772
1.4394
1.4216
SCF3
SO2Ph
3o
2.13
1.11
2.22
5.03
1.00
1.07
3.12
f1 (ppm)

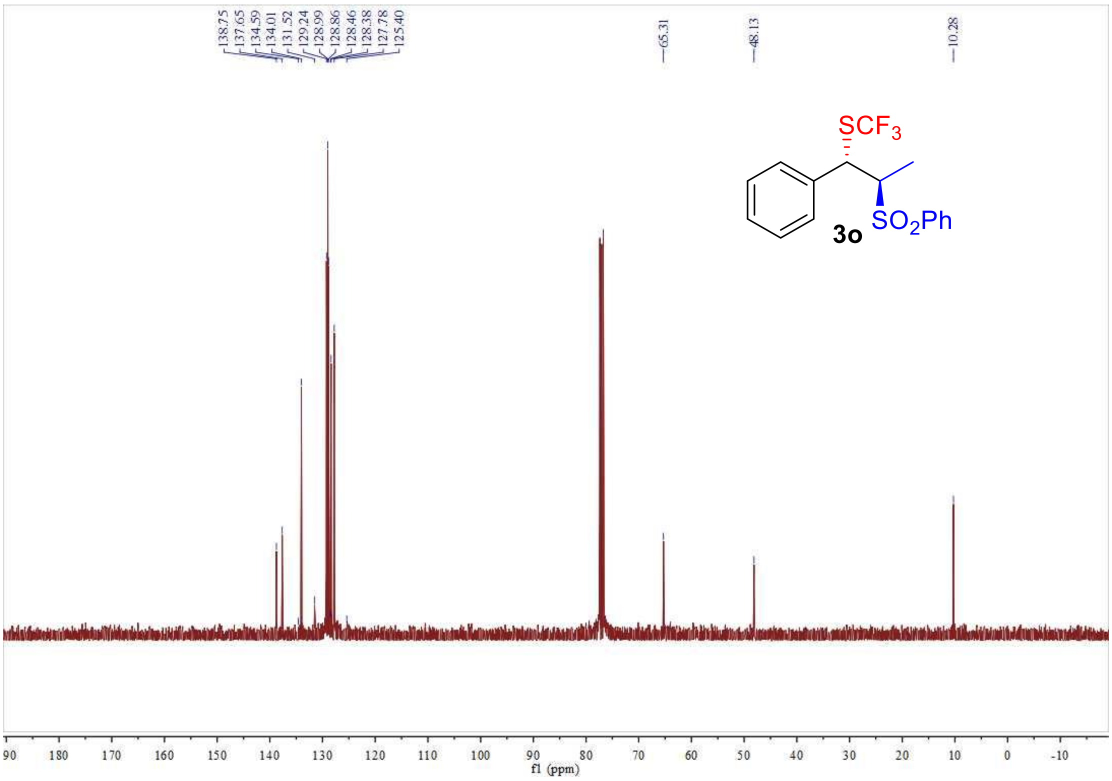
138.75
137.65
134.59
134.01
131.52
129.24
128.99
128.86
128.46
128.38
127.78
125.40
65.31
48.13
10.28
SCF3
SO2Ph
3o
f1 (ppm)

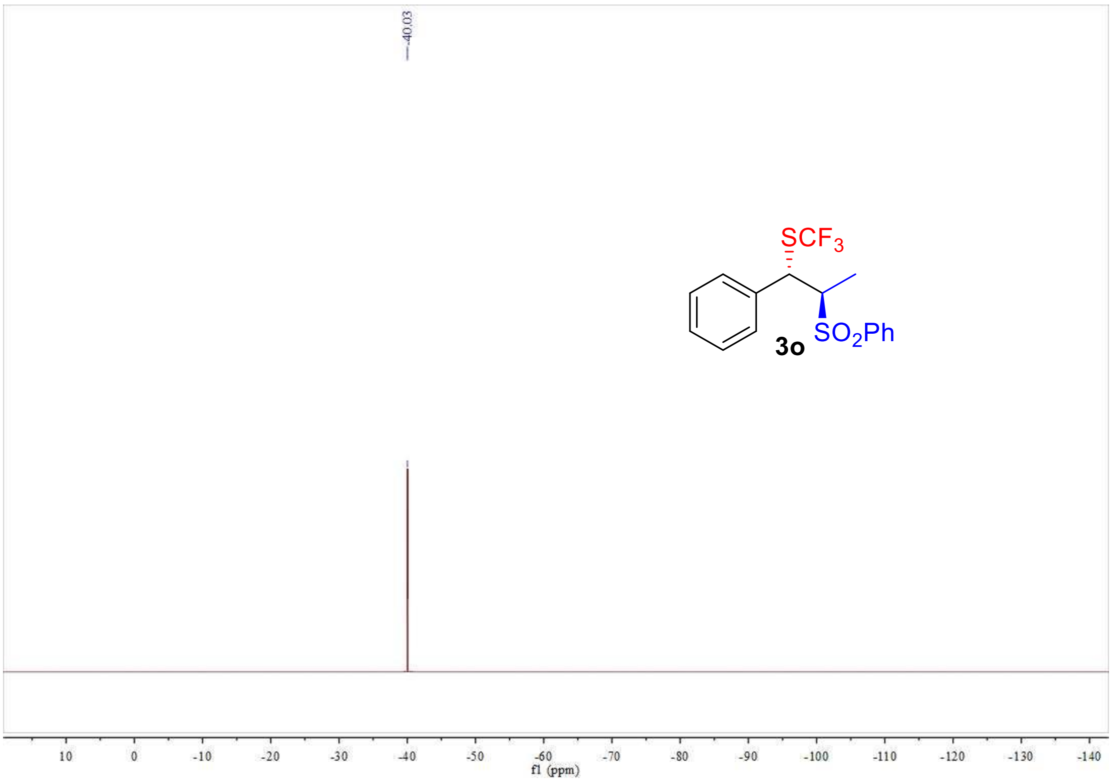
-40.03
SCF3
3o
SO2Ph
10
0
-10
-20
-30
-40
-50
-60
-70
-80
-90
-100
-110
-120
-130
-140
f1 (ppm)

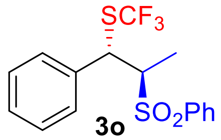

3o

Chemical Formula: $C_{16}H_{15}F_{3}O_{2}S_{2}$

Exact Mass: 360.0466

Molecular Weight: 360.4092

m/z: 360.0466 (100.0%), 361.0499 (17.3%), 362.0424 (9.0%), 361.0459 (1.6%), 363.0457 (1.6%), 362.0533 (1.4%)

Elemental Analysis: C, 53.32; H, 4.20; F, 15.81; O, 8.88; S, 17.79

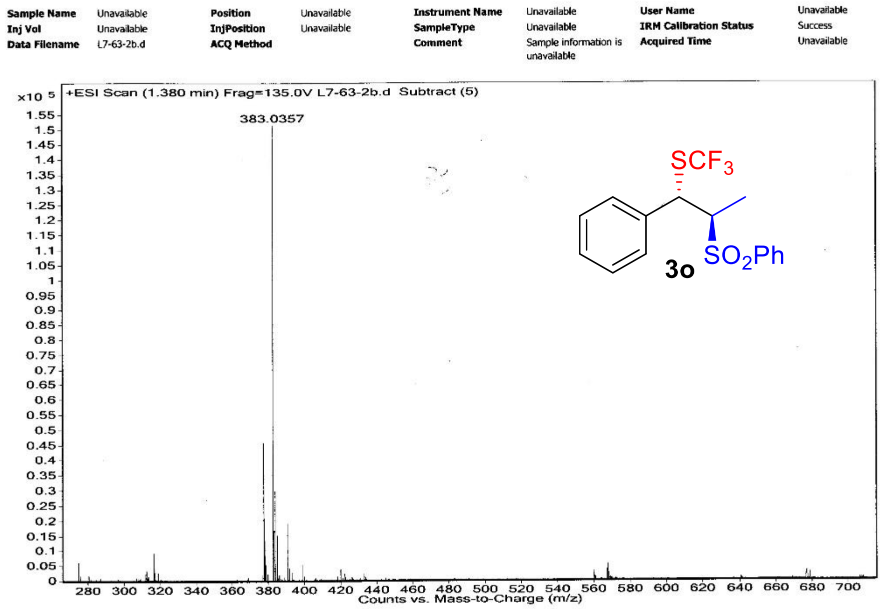

HRMS (ESI, m/z) calcd for $C_{16}H_{15}F_{3}O_{2}S_{2}$ $[M+Na]^{+}$ 383.0358, found 383.0357.

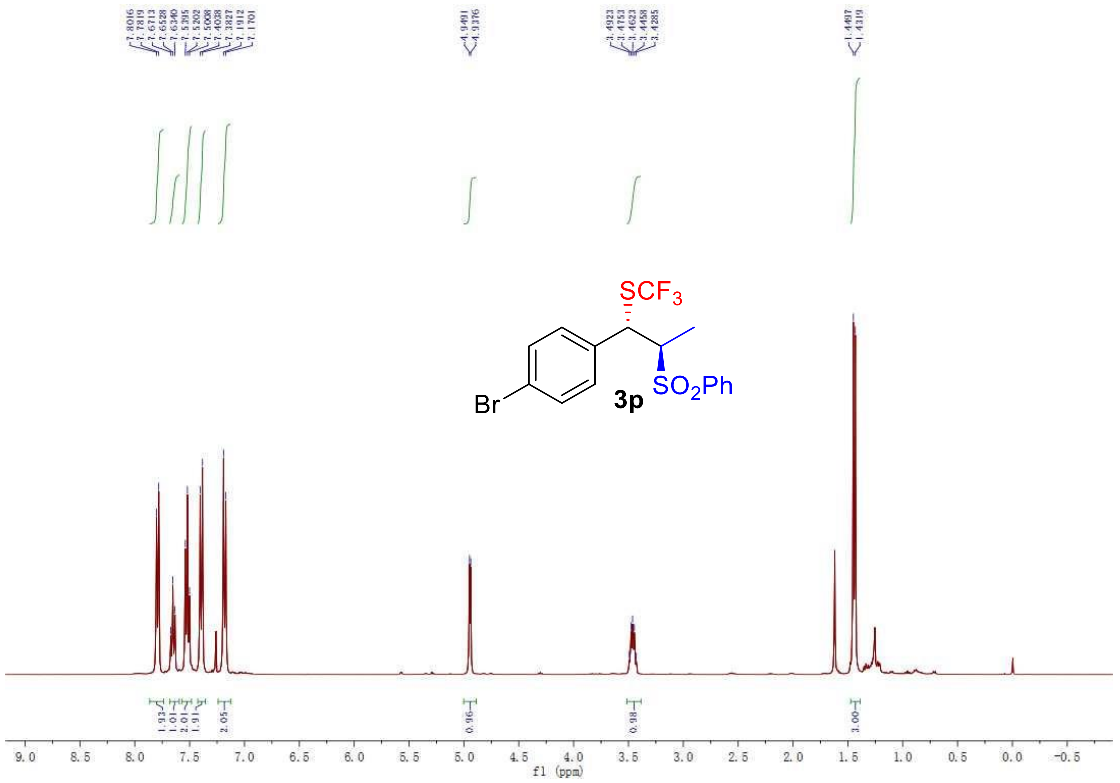
7.8016
7.7819
7.6713
7.6528
7.6340
7.5395
7.5202
7.5008
7.4028
7.3827
7.1912
7.1701
4.9491
4.9376
3.4923
3.4753
3.4623
3.4458
3.4285
1.4497
1.4319
SCF3
Br
3p
SO2Ph
1.93
1.01
2.01
1.91
2.05
0.96
0.98
3.00
9.0 8.5 8.0 7.5 7.0 6.5 6.0 5.5 5.0 4.5 4.0 3.5 3.0 2.5 2.0 1.5 1.0 0.5 0.0 -0.5
f1 (ppm)

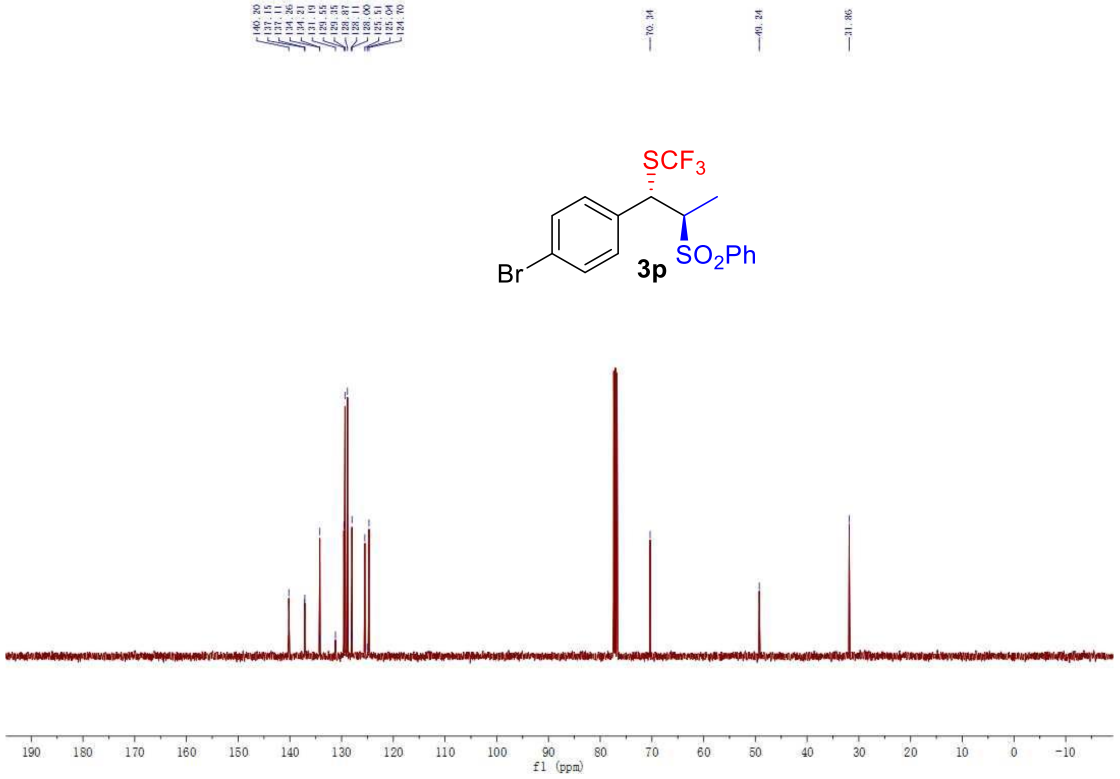
140.20
137.15
137.11
134.26
134.21
131.19
129.55
129.35
128.87
128.11
128.00
125.51
125.04
124.70
70.34
49.24
31.86
SCF3
Br
3p
SO2Ph
190 180 170 160 150 140 130 120 110 100 90 80 70 60 50 40 30 20 10 0 -10
f1 (ppm)

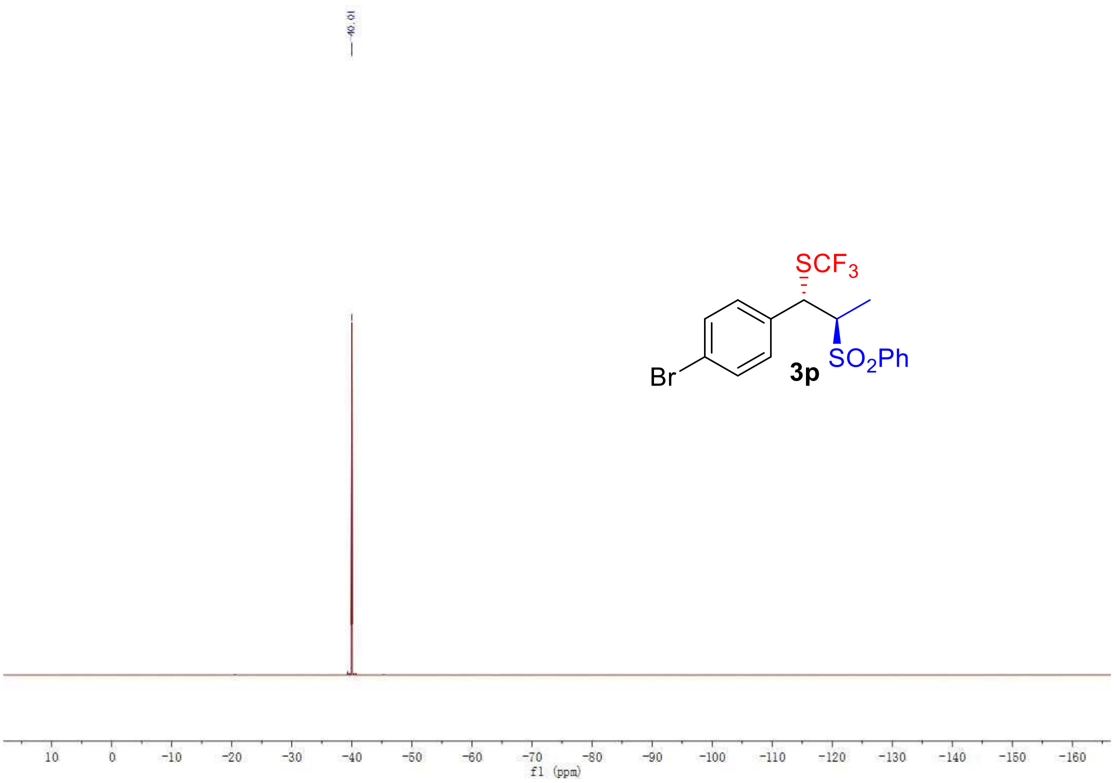
-40.01
10
0
-10
-20
-30
-40
-50
-60
-70
-80
-90
-100
-110
-120
-130
-140
-150
-160
f1 (ppm)
SCF3
Br
SO2Ph

3p

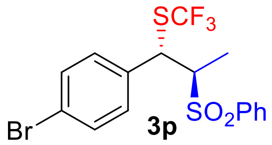

Chemical Formula: $C_{16}H_{14}BrF_3O_2S_2$
Exact Mass: 437.9571
Molecular Weight: 439.3052
m/z: 437.9571 (100.0%), 439.9550 (97.3%), 438.9604 (17.3%), 440.9584 (16.8%), 439.9529 (9.0%), 441.9508 (8.8%), 438.9565 (1.6%), 440.9544 (1.6%), 442.9542 (1.5%), 440.9562 (1.5%), 441.9617 (1.4%), 439.9638 (1.1%)
Elemental Analysis: C, 43.75; H, 3.21; Br, 18.19; F, 12.97; O, 7.28; S, 14.60

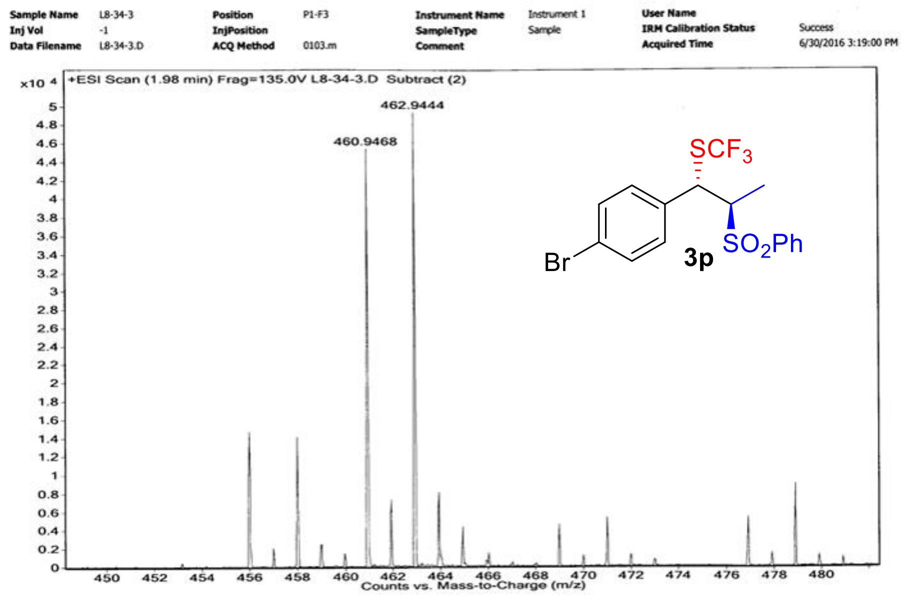

HRMS (ESI, m/z) calcd for $C_{16}H_{14}BrF_3O_2S_2$ $[M+Na]^+$ 460.9463, found 460.9468.

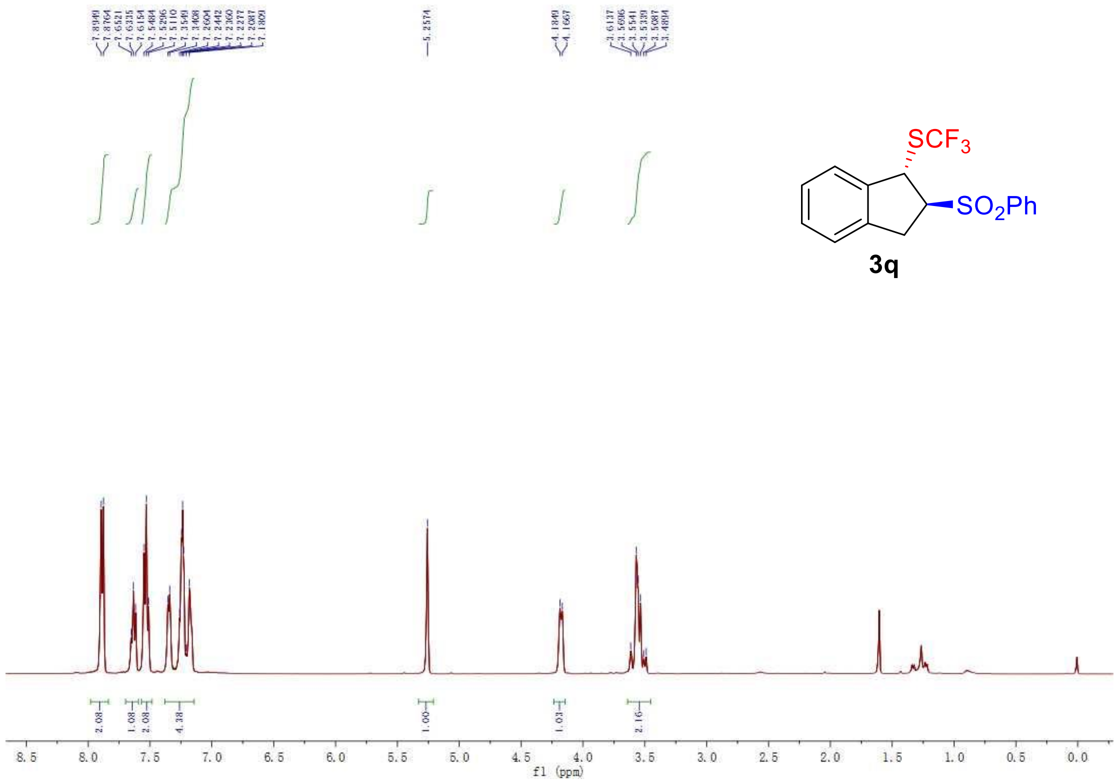

7.8940
7.8764
7.6521
7.6335
7.6154
7.5484
7.5296
7.5110
7.3549
7.3408
7.2604
7.2442
7.2362
7.2277
7.2087
7.1809
5.2574
4.1849
4.1667
3.6137
3.5696
3.5541
3.5339
3.5087
3.4894
SCF3
SO2Ph
3q
2.08
1.08
2.08
4.38
1.00
1.03
2.16
8.5 8.0 7.5 7.0 6.5 6.0 5.5 5.0 4.5 4.0 3.5 3.0 2.5 2.0 1.5 1.0 0.5 0.0
f1 (ppm)

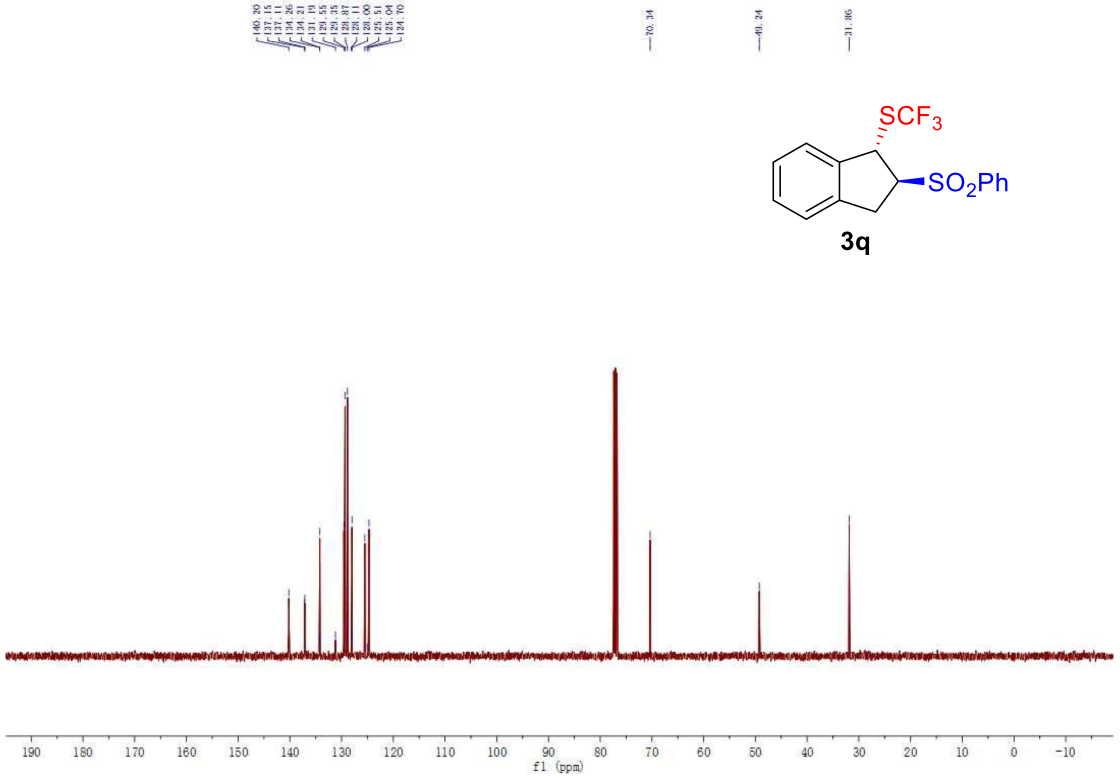

140.20
137.15
137.11
134.26
134.21
131.19
129.55
129.35
128.87
128.11
128.00
125.51
125.04
124.70
70.34
49.24
31.86
SCF3
SO2Ph
3q
190 180 170 160 150 140 130 120 110 100 90 80 70 60 50 40 30 20 10 0 -10
f1 (ppm)

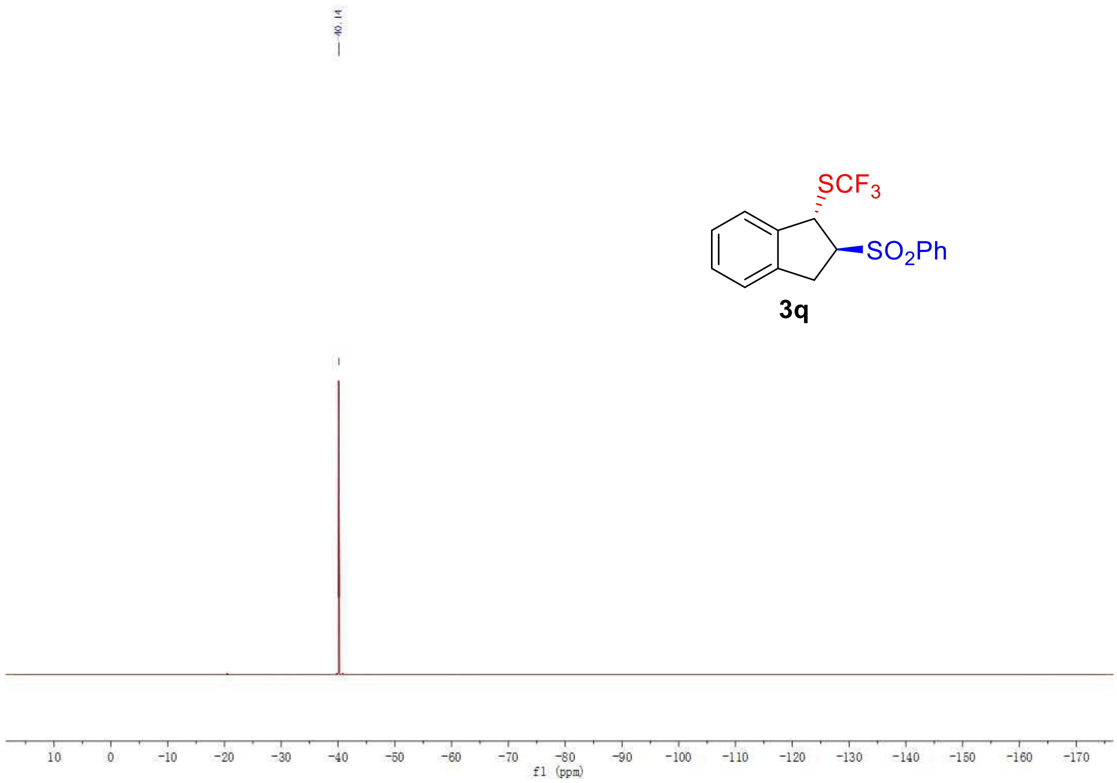

-40.14
SCF3
SO2Ph
3q
10
0
-10
-20
-30
-40
-50
-60
-70
-80
-90
-100
-110
-120
-130
-140
-150
-160
-170
f1 (ppm)

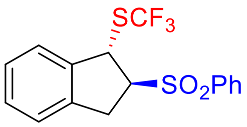

3q

Chemical Formula: $C_{16}H_{13}F_3O_2S_2$
Exact Mass: 358.0309
Molecular Weight: 358.3932
m/z: 358.0309 (100.0%), 359.0343 (17.3%), 360.0267 (9.0%), 359.0303 (1.6%), 361.0301 (1.6%), 360.0376 (1.4%)
Elemental Analysis: C, 53.62; H, 3.66; F, 15.90; O, 8.93; S, 17.89

**Sample Name**	2016-0718-L8-31-4	**Position**	P1-E9	**Instrument Name**	Instrument 1	**User Name**	
**Inj Vol**	-1	**InjPosition**		**SampleType**	Sample	**IRM Calibration Status**	Success
**Data Filename**	2016-0718-L8-31-4.d	**ACQ Method**	0103.m	**Comment**		**Acquired Time**	7/18/2016 3:44:55 PM

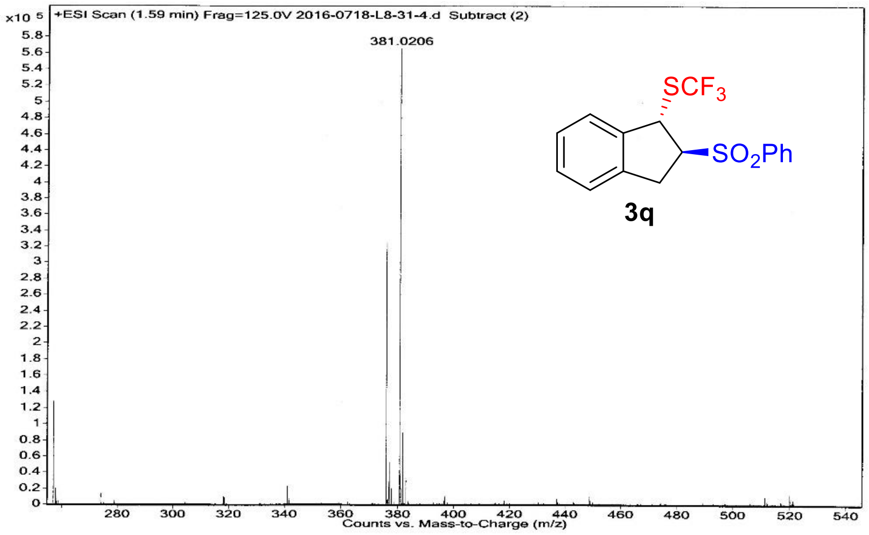

HRMS (ESI, m/z) calcd for $C_{16}H_{13}F_3O_2S_2$ $[M+Na]^+$ 381.0201, found 381.0206.

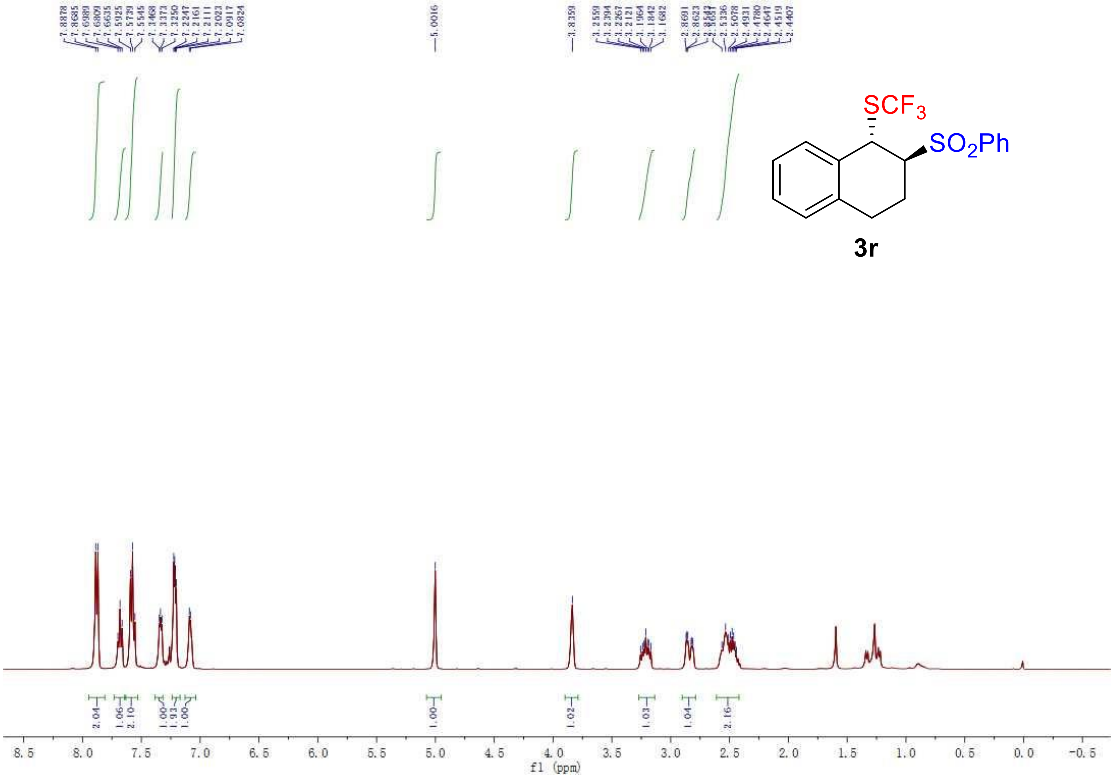
SCF3
SO2Ph
3r
f1 (ppm)

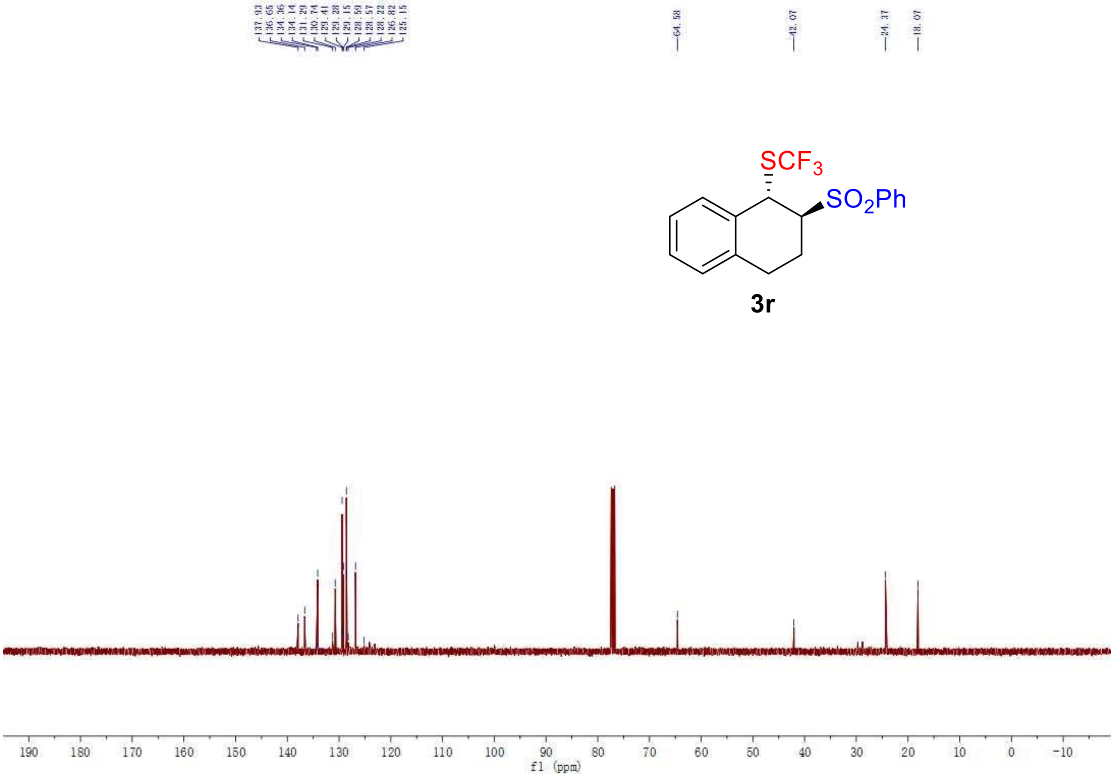
SCF3
SO2Ph
3r
137.93
136.65
134.36
134.14
131.29
130.74
129.41
129.28
129.15
128.59
128.57
128.22
126.82
125.15
64.58
42.07
24.37
18.07
f1 (ppm)

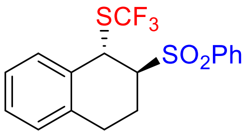
SCF3
SO2Ph

3r

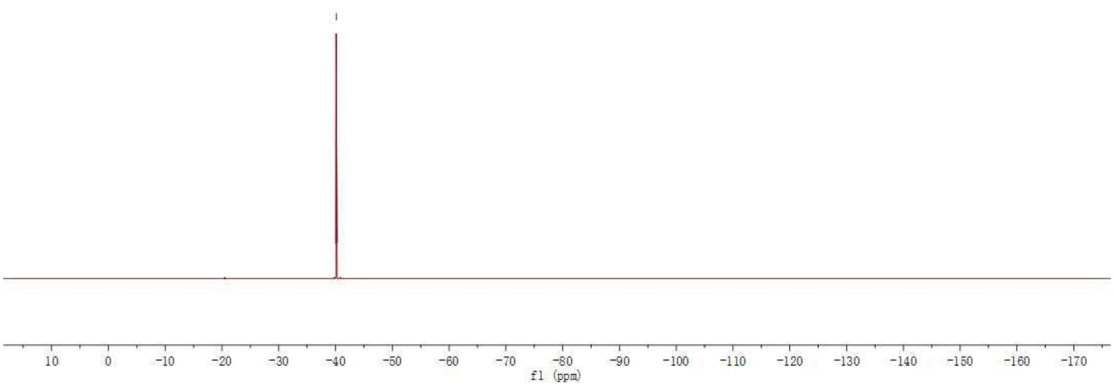
-40.14
10 0 -10 -20 -30 -40 -50 -60 -70 -80 -90 -100 -110 -120 -130 -140 -150 -160 -170
f1 (ppm)

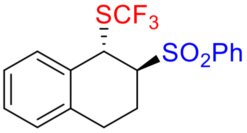

3r

Chemical Formula: $C_{17}H_{15}F_3O_2S_2$
Exact Mass: 372.0466
Molecular Weight: 372.4202
m/z: 372.0466 (100.0%), 373.0499 (18.4%), 374.0424 (9.0%), 375.0457 (1.7%), 373.0459 (1.6%), 374.0533 (1.6%)
Elemental Analysis: C, 54.83; H, 4.06; F, 15.30; O, 8.59; S, 17.22

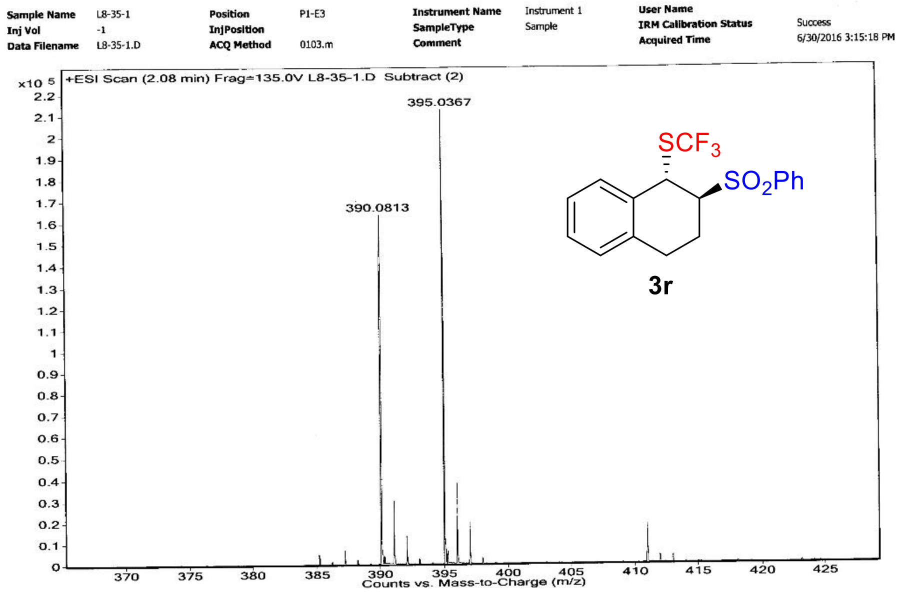

HRMS (ESI, m/z) calcd for $C_{17}H_{15}F_3O_2S_2$ $[M+Na]^+$ 395.0358, found 395.0367.

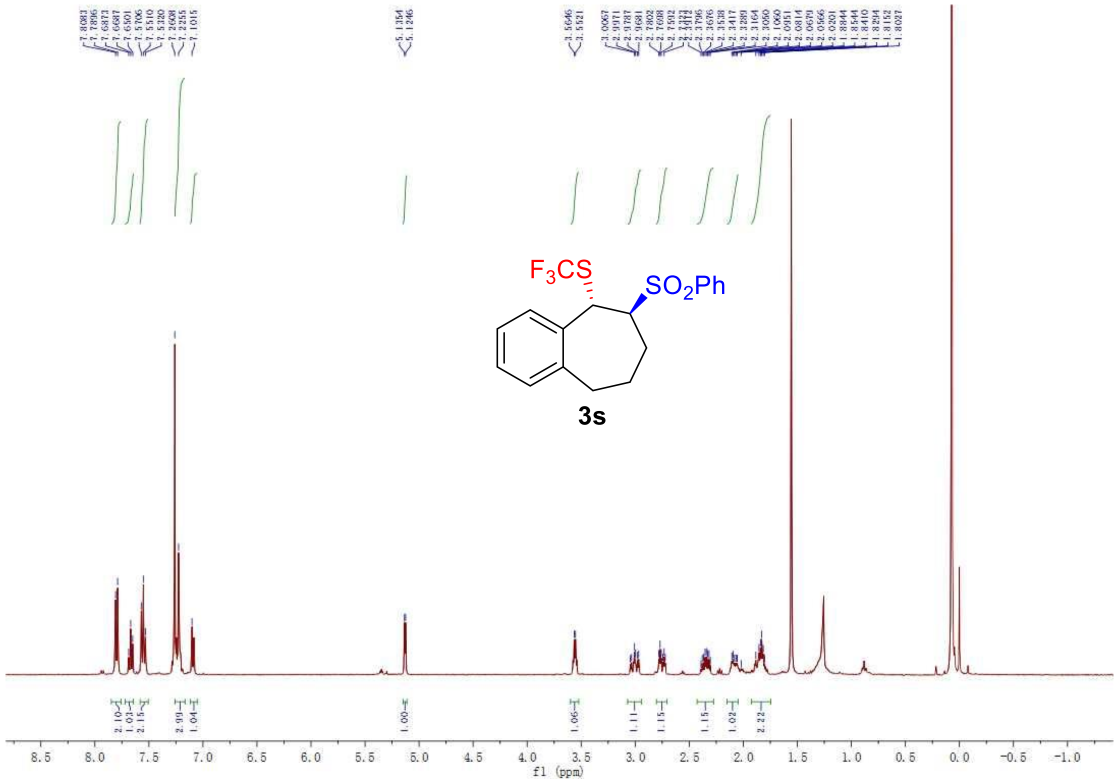
F3CS
SO2Ph
3s
f1 (ppm)

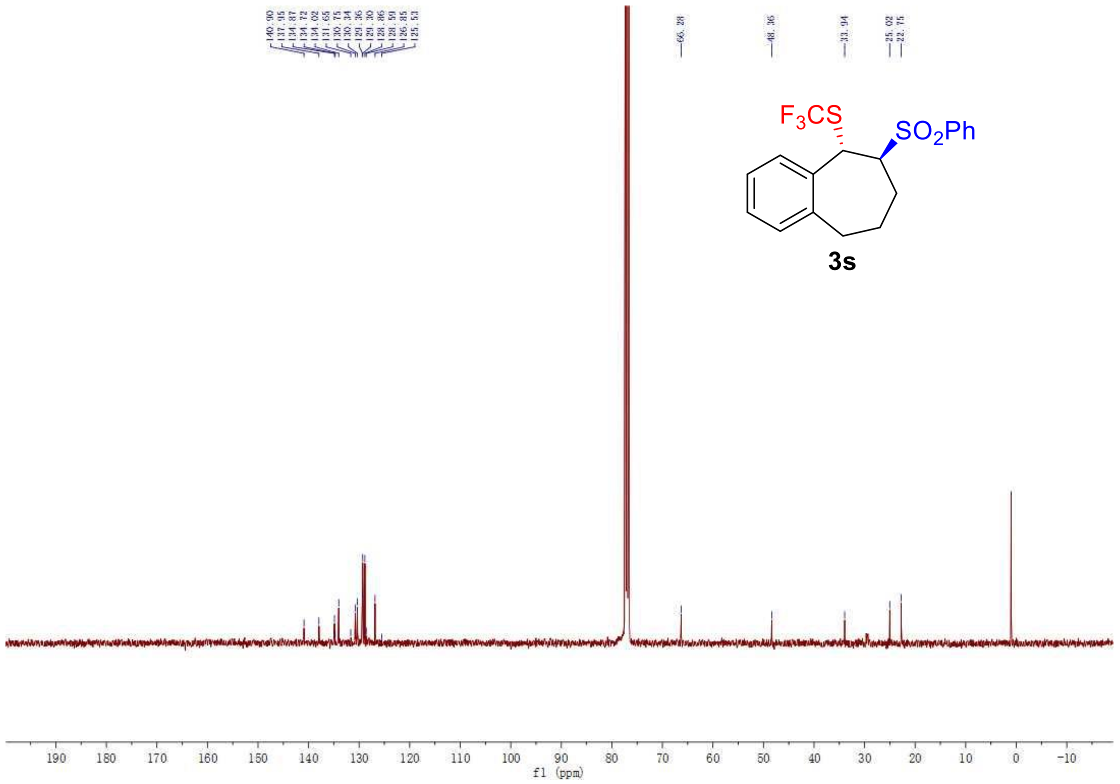
140.90
137.95
134.87
134.72
134.02
131.65
130.75
130.34
129.36
129.30
128.86
128.51
126.85
125.53
66.28
48.36
33.94
25.02
22.74
F3CS
SO2Ph
3s
f1 (ppm)

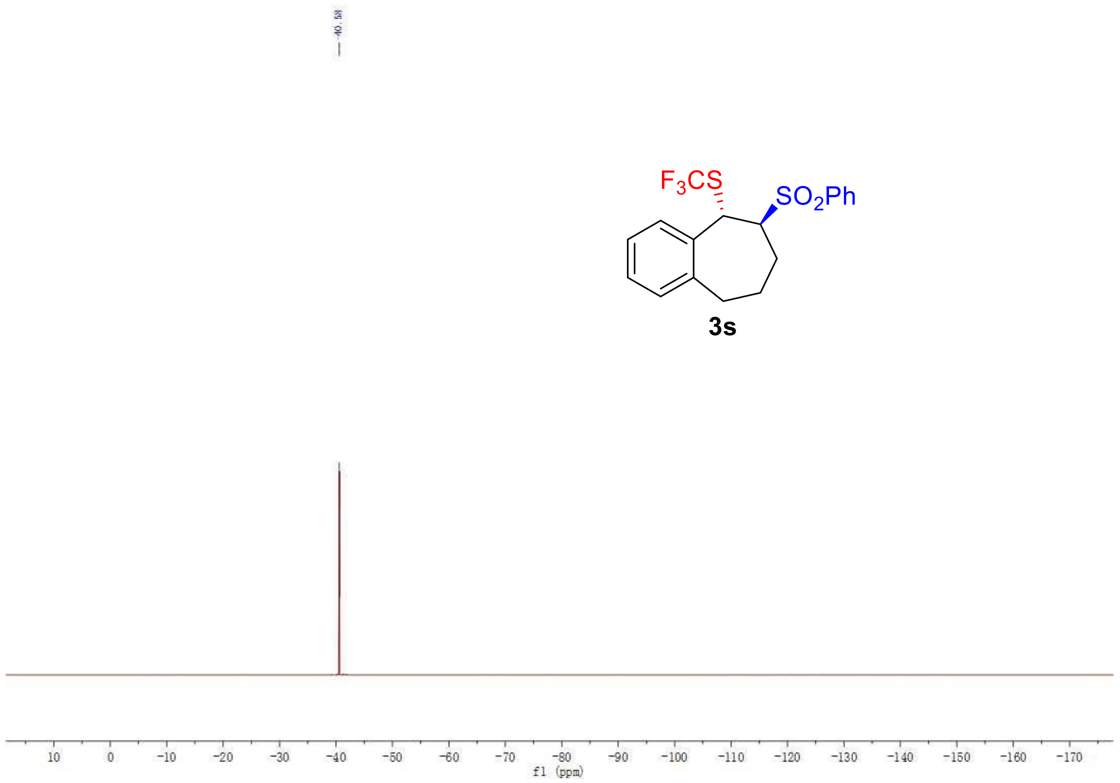

-40.58
F3CS
SO2Ph
3s
10 0 -10 -20 -30 -40 -50 -60 -70 -80 -90 -100 -110 -120 -130 -140 -150 -160 -170
f1 (ppm)

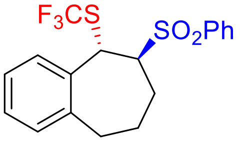

3s

Chemical Formula: $C_{18}H_{17}F_3O_2S_2$
Exact Mass: 386.0622
Molecular Weight: 386.4472
m/z: 386.0622 (100.0%), 387.0656 (19.5%), 388.0580 (9.0%), 388.0689 (1.8%), 389.0614 (1.8%), 387.0616 (1.6%)
Elemental Analysis: C, 55.95; H, 4.43; F, 14.75; O, 8.28; S, 16.59

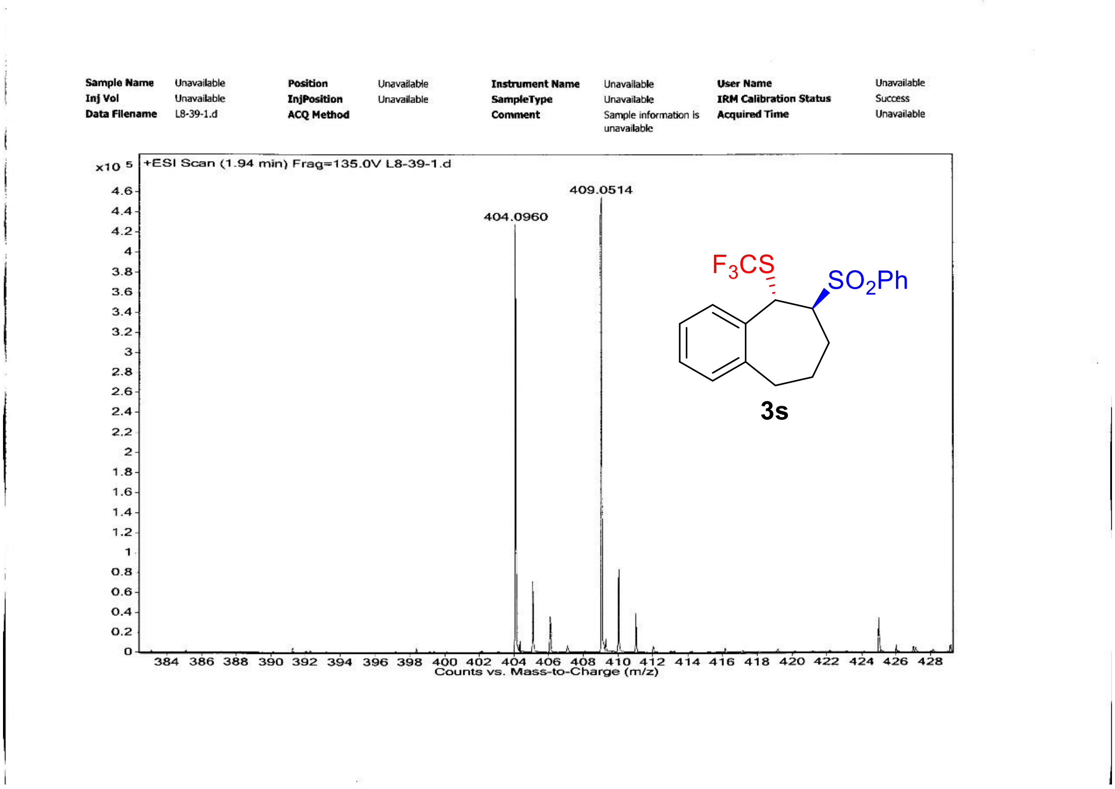

HRMS (ESI, m/z) calcd for $C_{18}H_{17}F_3O_2S_2$ $[M+Na]^+$ 409.0514, found 409.0514.

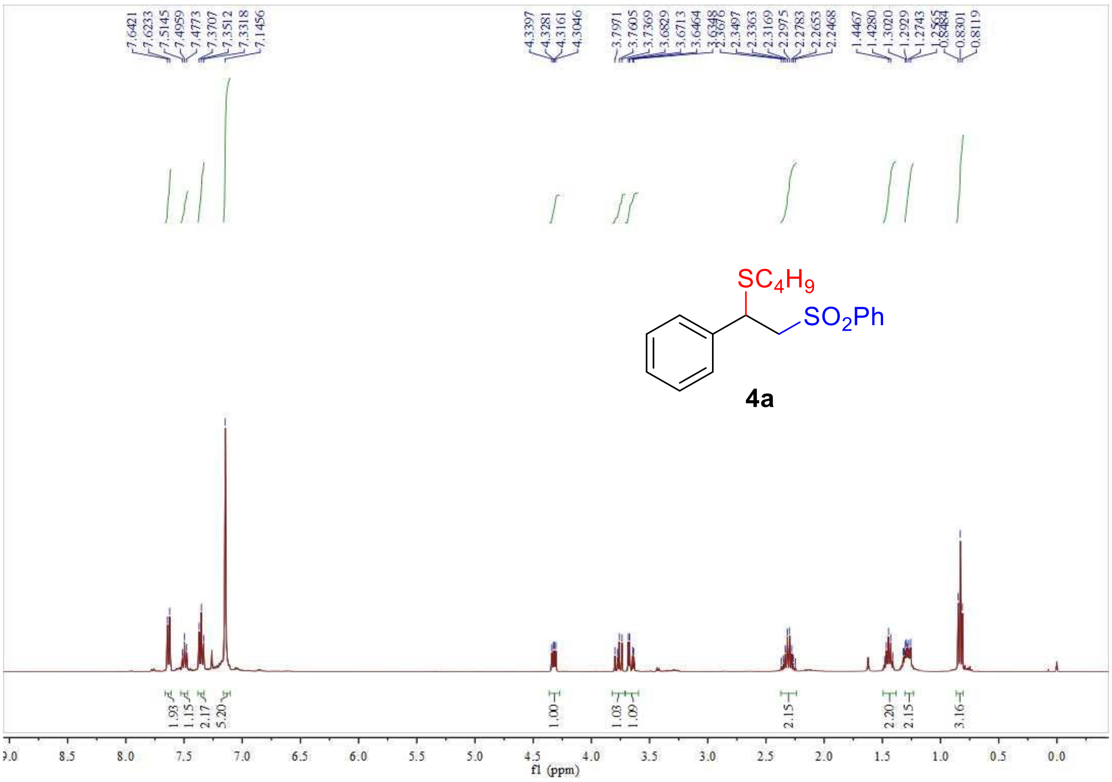
SC4H9
SO2Ph
4a
f1 (ppm)

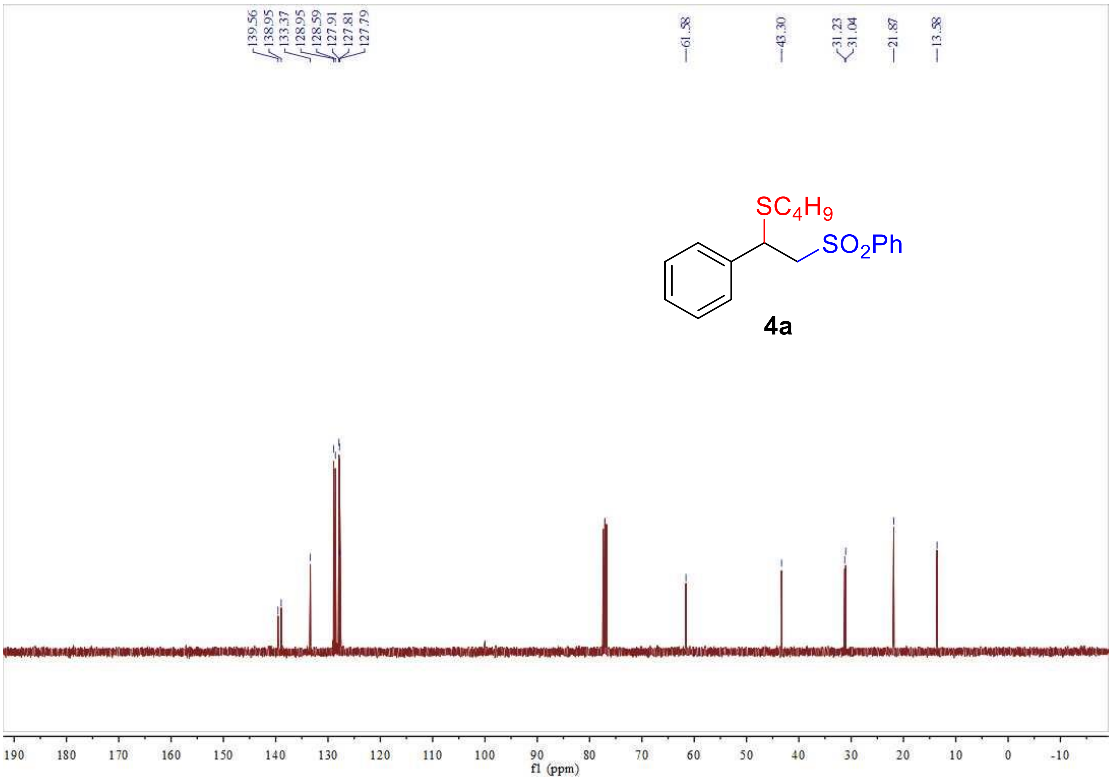
SC4H9
SO2Ph
4a
f1 (ppm)

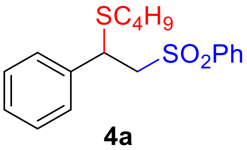

4a

Chemical Formula: $C_{18}H_{22}O_2S_2$
Exact Mass: 334.1061
Molecular Weight: 334.4920
m/z: 334.1061 (100.0%), 335.1095 (19.5%), 336.1019 (9.0%), 336.1128 (1.8%), 337.1053 (1.8%), 335.1055 (1.6%)
Elemental Analysis: C, 64.63; H, 6.63; O, 9.57; S, 19.17

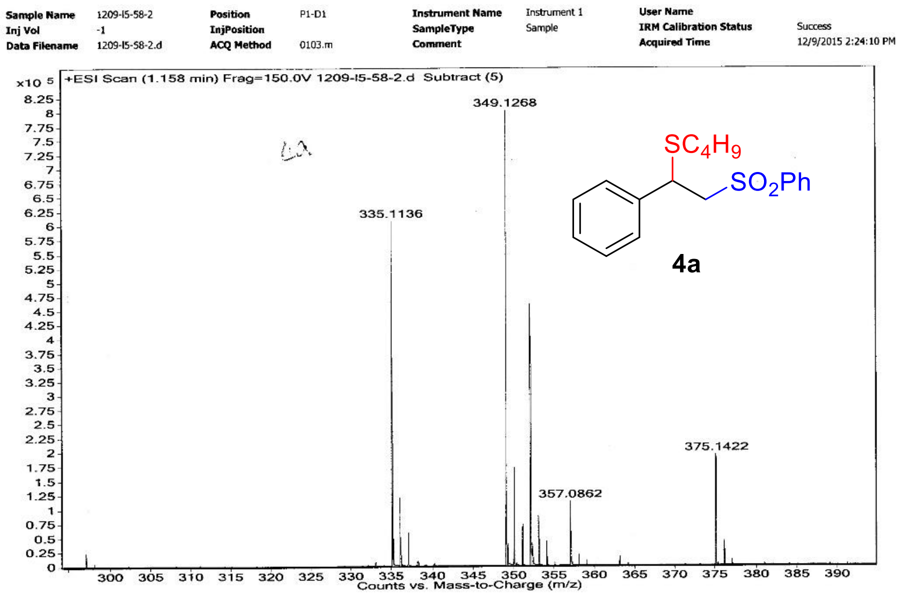

HRMS (ESI, m/z) calcd for $C_{18}H_{22}O_2S_2$ $[M+H]^+$ 335.1134, found 335.1136.

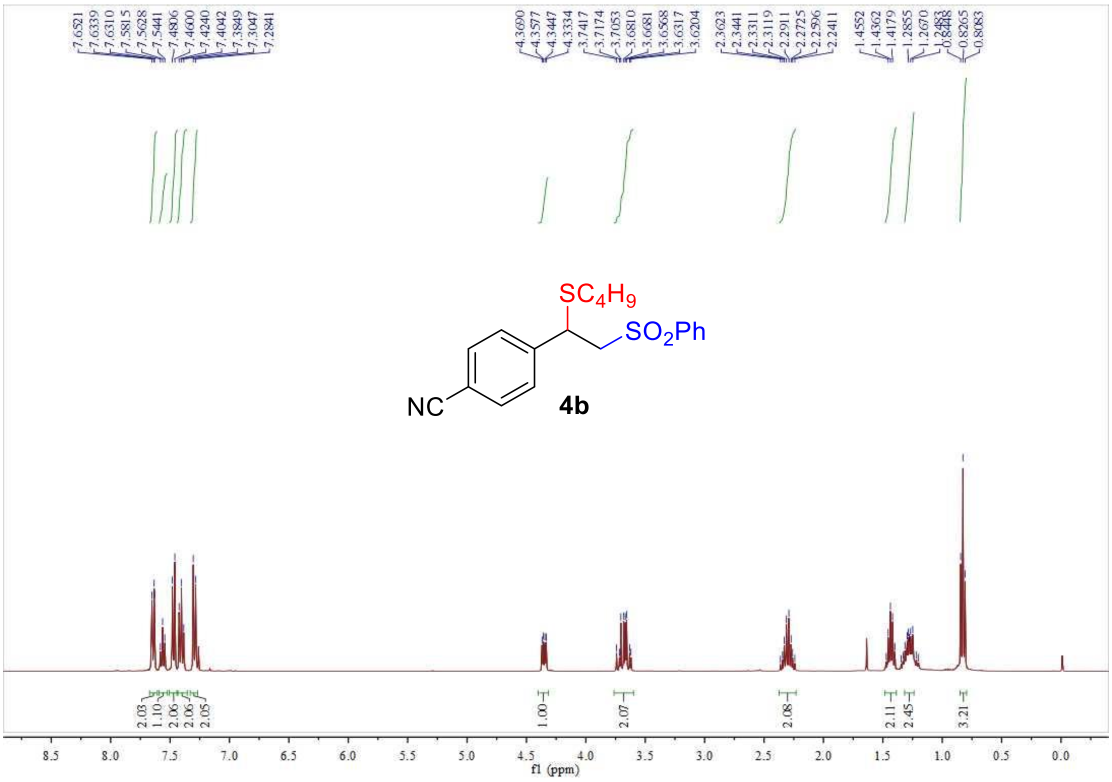
SC4H9
SO2Ph
NC
4b
f1 (ppm)

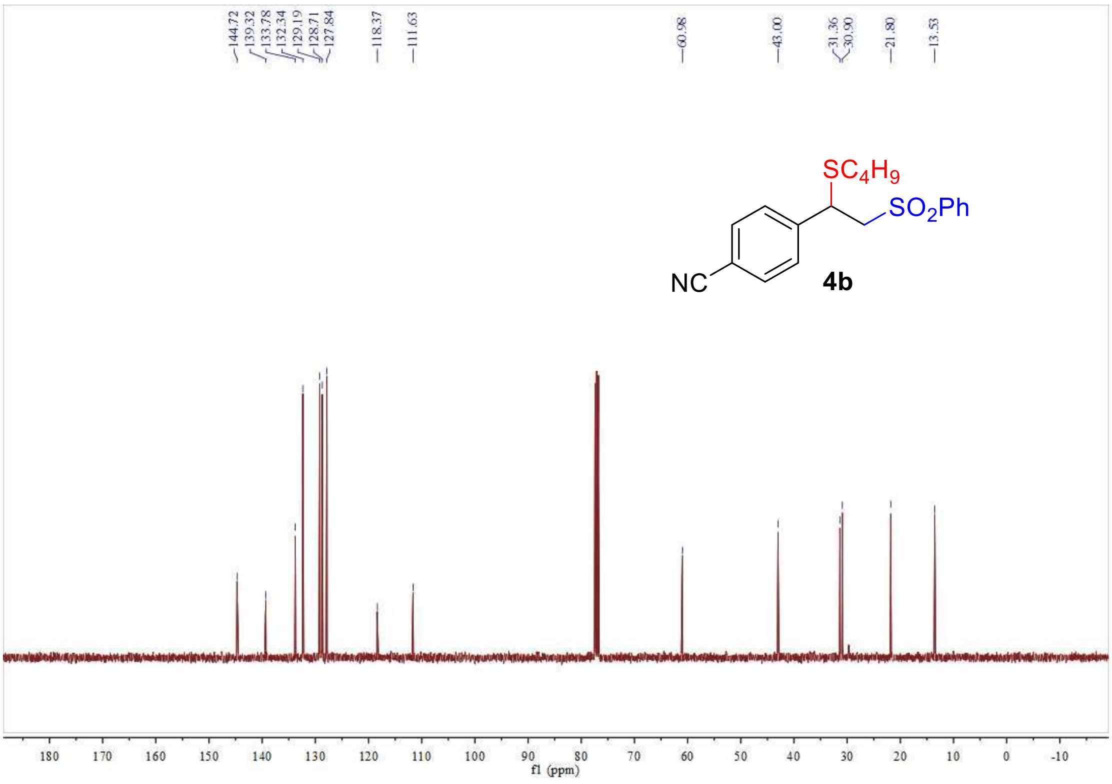
SC4H9
SO2Ph
NC
4b
f1 (ppm)

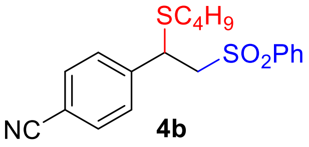

Chemical Formula: $C_{19}H_{21}NO_2S_2$
Exact Mass: 359.1014
Molecular Weight: 359.5020
m/z: 359.1014 (100.0%), 360.1047 (20.5%), 361.0972 (9.0%), 361.1081 (2.0%), 362.1005 (1.9%), 360.1008 (1.6%)
Elemental Analysis: C, 63.48; H, 5.89; N, 3.90; O, 8.90; S, 17.84

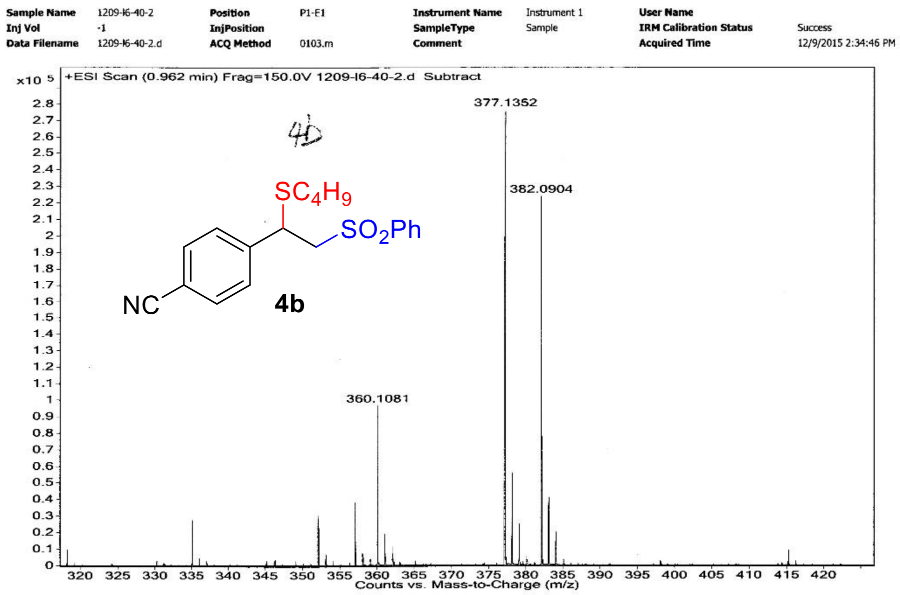

HRMS (ESI, m/z) calcd for $C_{19}H_{21}NO_2S_2$ $[M+H]^+$ 360.1086, found 360.1081.

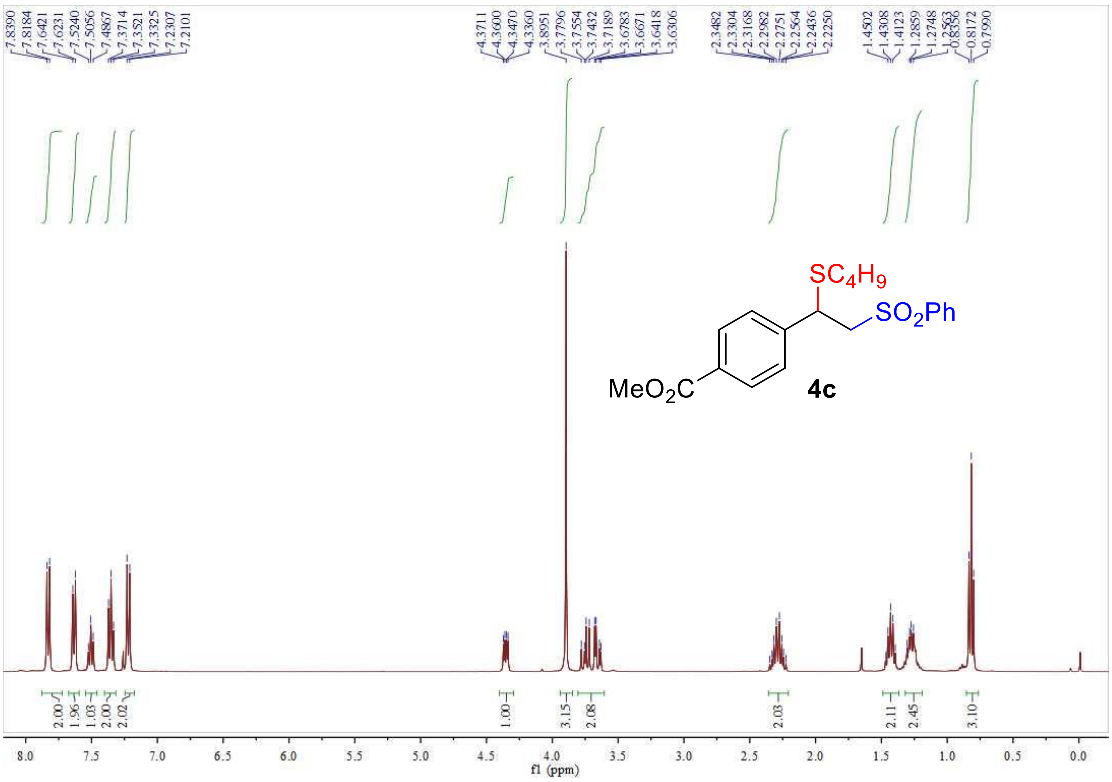

7.8390
7.8184
7.6421
7.6231
7.5240
7.5056
7.4867
7.3714
7.3521
7.3325
7.2307
7.2101
4.3711
4.3600
4.3470
4.3360
3.8951
3.7796
3.7554
3.7432
3.7189
3.6783
3.6671
3.6418
3.6306
2.3482
2.3304
2.3168
2.2982
2.2751
2.2564
2.2436
2.2250
1.4502
1.4308
1.4123
1.2859
1.2748
0.8356
0.8172
0.7990
SC_4H_9
SO_2Ph
MeO_2C
4c
2.00
1.96
1.03
2.00
2.02
1.00
3.15
2.08
2.03
2.11
2.45
3.10
f1 (ppm)

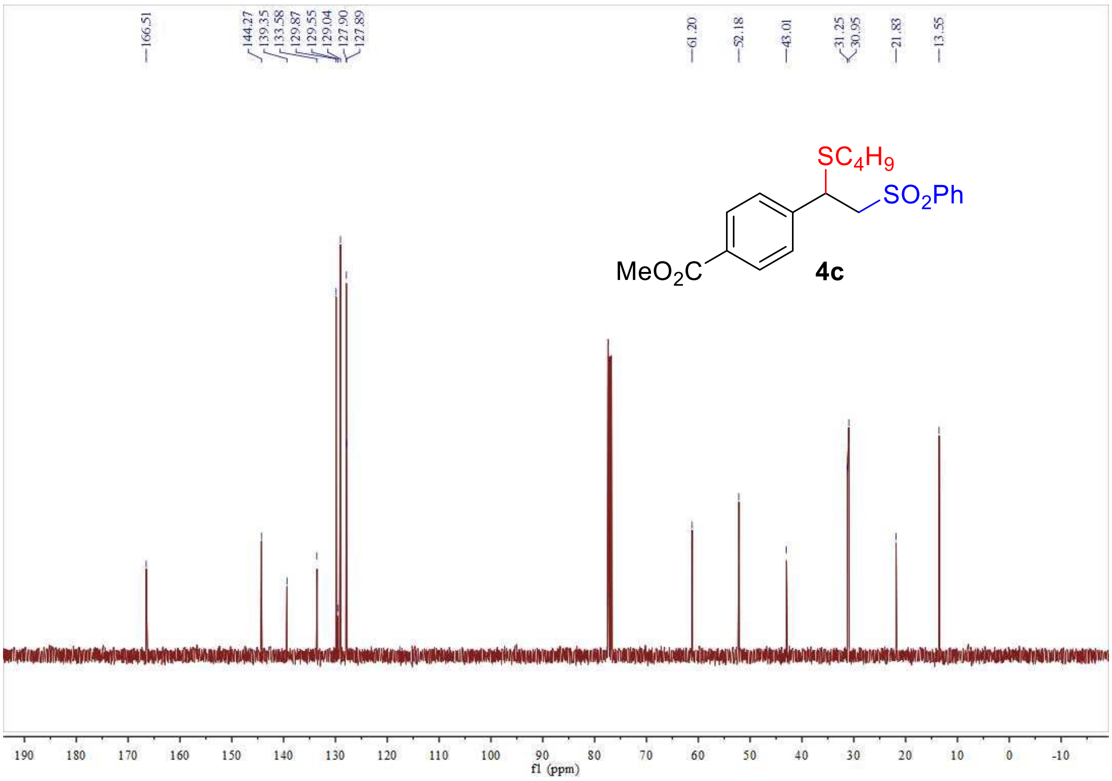

166.51
144.27
139.35
133.58
129.87
129.55
129.04
127.90
127.89
61.20
52.18
43.01
31.25
30.95
21.83
13.55
SC_4H_9
SO_2Ph
MeO_2C
4c
f1 (ppm)

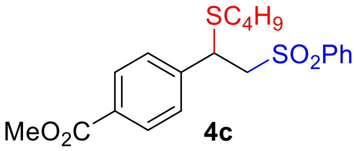

4c

Chemical Formula: $C_{20}H_{24}O_4S_2$

Exact Mass: 392.1116

Molecular Weight: 392.5280

m/z: 392.1116 (100.0%), 393.1150 (21.6%), 394.1074 (9.0%), 394.1183 (2.2%), 395.1108 (2.0%), 393.1110 (1.6%)

Elemental Analysis: C, 61.20; H, 6.16; O, 16.30; S, 16.34

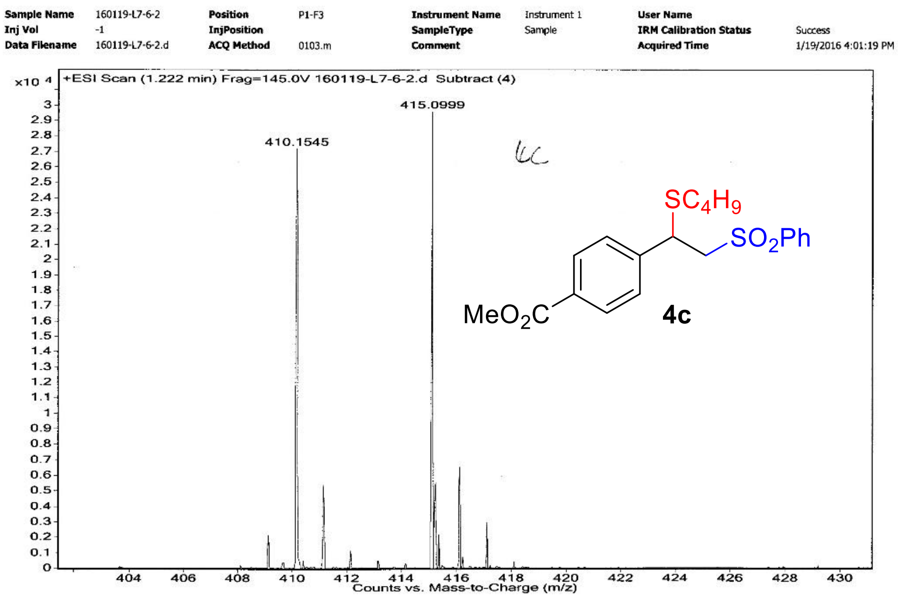

HRMS (ESI, m/z) calcd for $C_{20}H_{24}O_4S_2$ $[M+Na]^+$ 415.1008, found 415.0999.

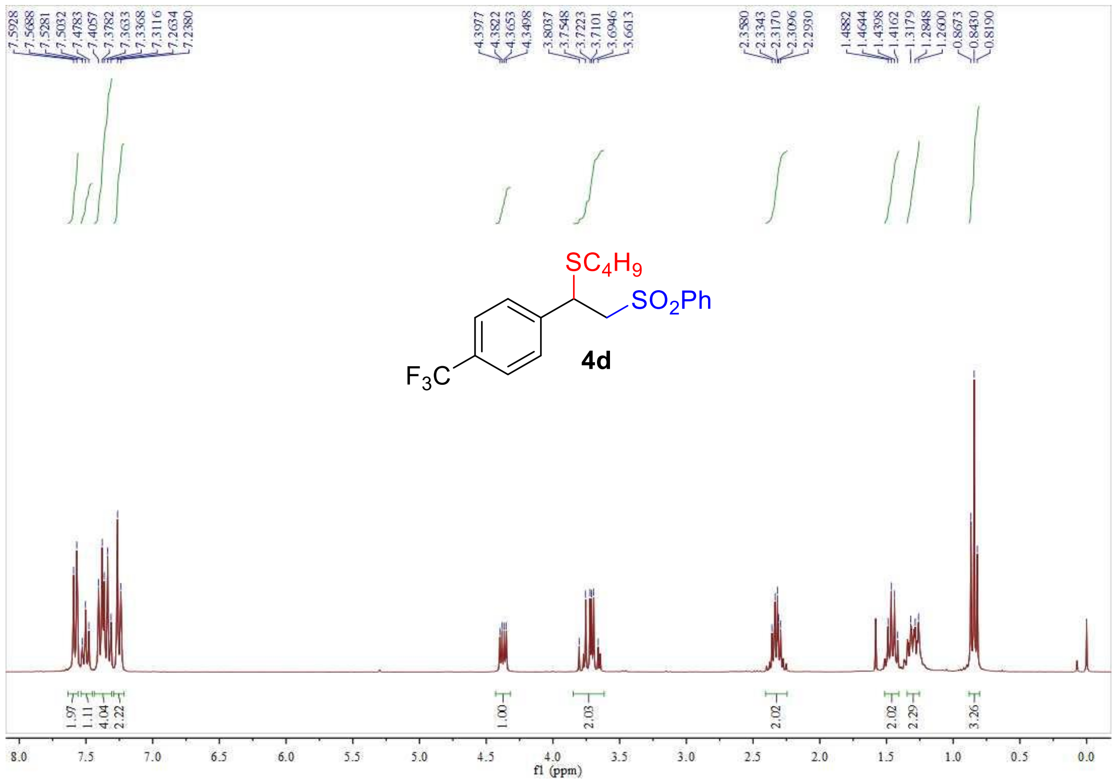
7.5928
7.5688
7.5281
7.5032
7.4783
7.4057
7.3782
7.3633
7.3368
7.3116
7.2634
7.2380
4.3977
4.3822
4.3653
4.3498
3.8037
3.7548
3.7223
3.7101
3.6946
3.6613
2.3580
2.3343
2.3170
2.3096
2.2930
1.4882
1.4644
1.4398
1.4162
1.3179
1.2848
1.2600
0.8673
0.8430
0.8190
SC4H9
SO2Ph
F3C
4d
1.97
1.11
4.04
2.22
1.00
2.03
2.02
2.02
2.29
3.26
f1 (ppm)

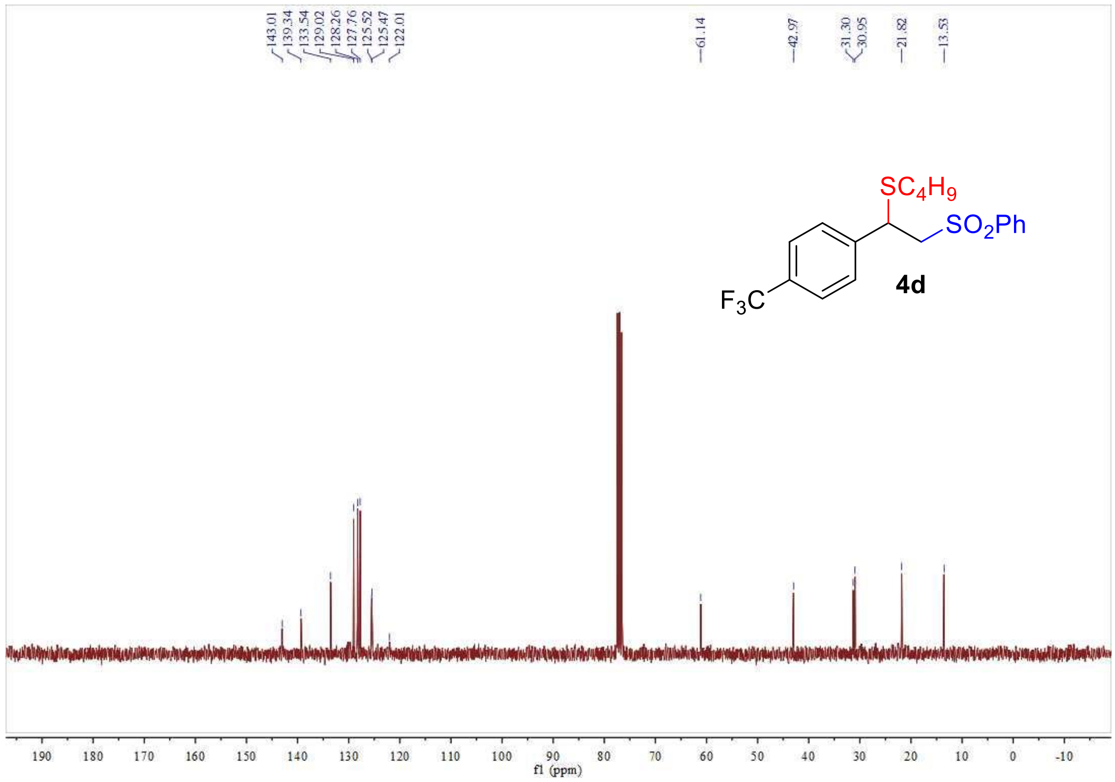
143.01
139.34
133.54
129.02
128.26
127.76
125.52
125.47
122.01
61.14
42.97
31.30
30.95
21.82
13.53
SC4H9
SO2Ph
F3C
4d
f1 (ppm)

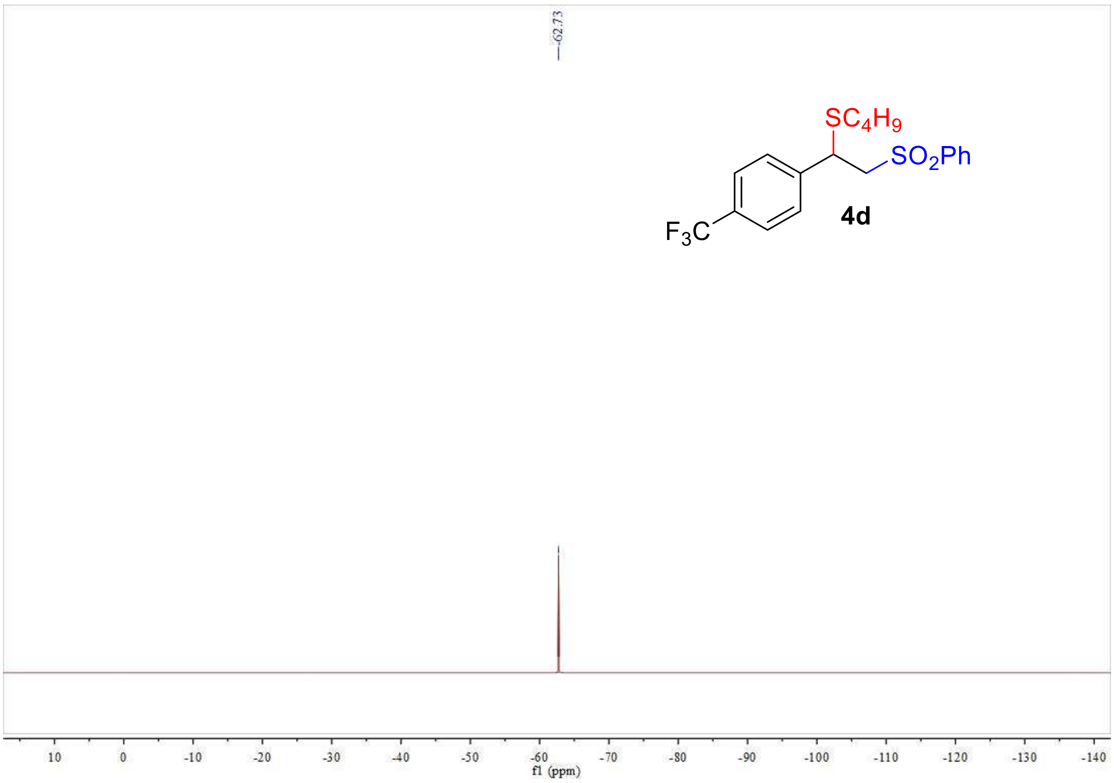

-62.73
SC4H9
SO2Ph
F3C
4d
10
0
-10
-20
-30
-40
-50
-60
-70
-80
-90
-100
-110
-120
-130
-140
f1 (ppm)

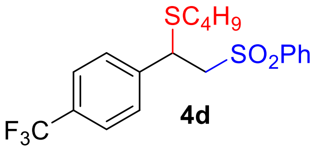

4d

Chemical Formula: $C_{19}H_{21}F_3O_2S_2$
Exact Mass: 402.0935
Molecular Weight: 402.4902
m/z: 402.0935 (100.0%), 403.0969 (20.5%), 404.0893 (9.0%), 404.1002 (2.0%), 405.0927 (1.9%), 403.0929 (1.6%)
Elemental Analysis: C, 56.70; H, 5.26; F, 14.16; O, 7.95; S, 15.93

**Sample Name**	2016-0309-L6-71-1	**Position**	P1-E9	**Instrument Name**	Instrument 1	**User Name**	
**Inj Vol**	-1	**InjPosition**		**SampleType**	Sample	**IRM Calibration Status**	Success
**Data Filename**	2016-0309-L6-71-1.d	**ACQ Method**	0103.m	**Comment**		**Acquired Time**	3/6/2016 2:45:23 PM

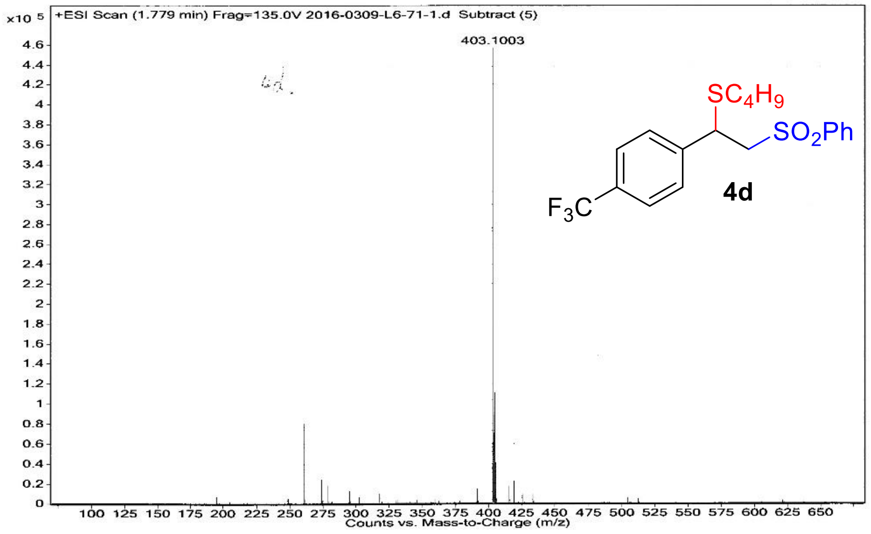

HRMS (ESI, m/z) calcd for $C_{19}H_{21}F_3O_2S_2$ $[M+H]^+$ 403.1008, found 403.1003.

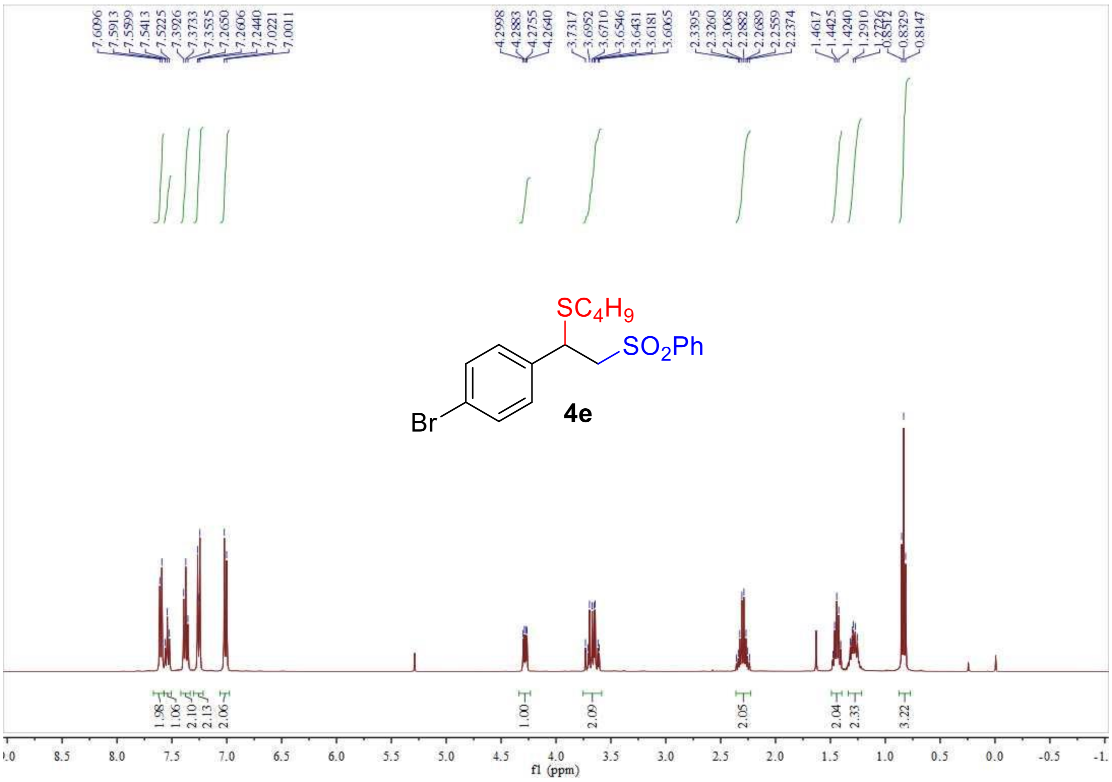
7.6096
7.5913
7.5599
7.5413
7.5225
7.3926
7.3733
7.3535
7.2650
7.2606
7.2440
7.0221
7.0011
4.2998
4.2883
4.2755
4.2640
3.7317
3.6952
3.6710
3.6546
3.6431
3.6181
3.6065
2.3395
2.3260
2.3068
2.2882
2.2689
2.2559
2.2374
1.4617
1.4425
1.4240
1.2910
0.8512
0.8329
0.8147
SC4H9
SO2Ph
Br
4e
1.98
1.06
2.10
2.13
2.06
1.00
2.09
2.05
2.04
2.33
3.22
f1 (ppm)

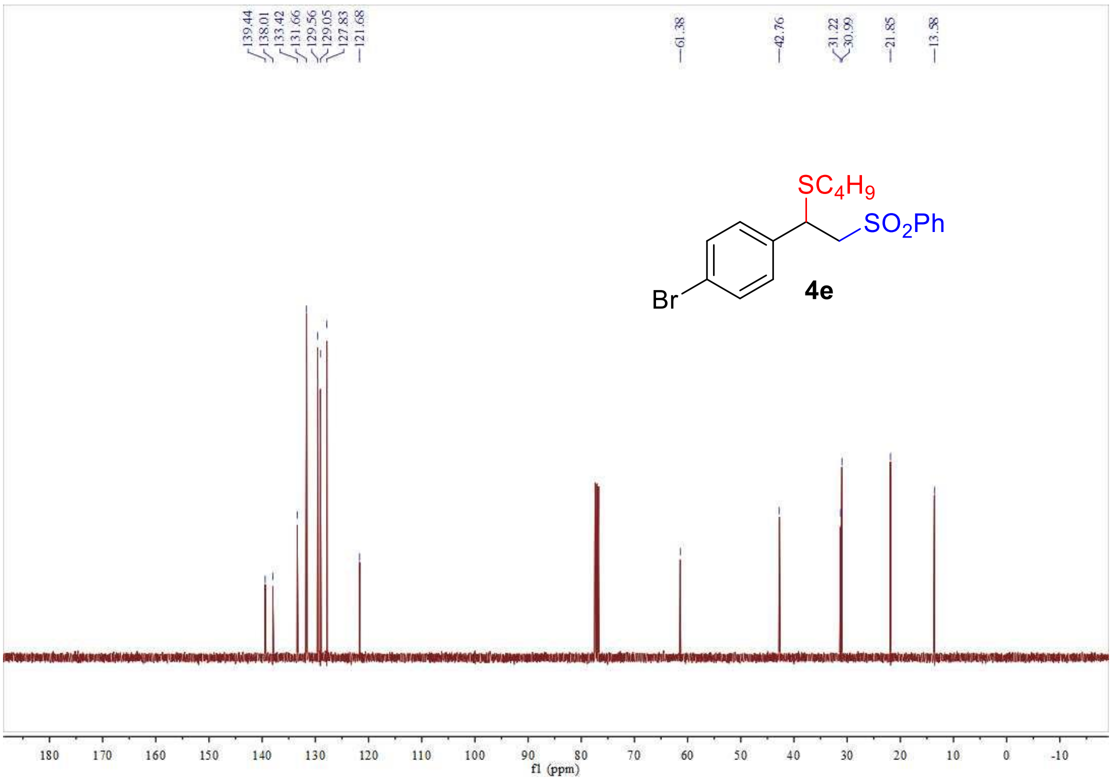
139.44
138.01
133.42
131.66
129.56
129.05
127.83
121.68
61.38
42.76
31.22
30.99
21.85
13.58
SC4H9
SO2Ph
Br
4e
f1 (ppm)

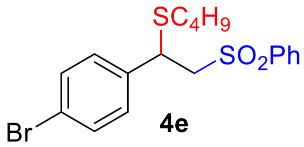

4e

Chemical Formula: $C_{18}H_{21}BrO_2S_2$
Exact Mass: 412.0166
Molecular Weight: 413.3880
m/z: 412.0166 (100.0%), 414.0146 (97.3%), 413.0200 (19.5%), 415.0179 (18.9%), 414.0124 (9.0%), 416.0104 (8.8%), 416.0213 (1.7%), 417.0137 (1.7%), 413.0160 (1.6%), 415.0140 (1.6%), 415.0158 (1.5%), 414.0233 (1.1%)
Elemental Analysis: C, 52.30; H, 5.12; Br, 19.33; O, 7.74; S, 15.51

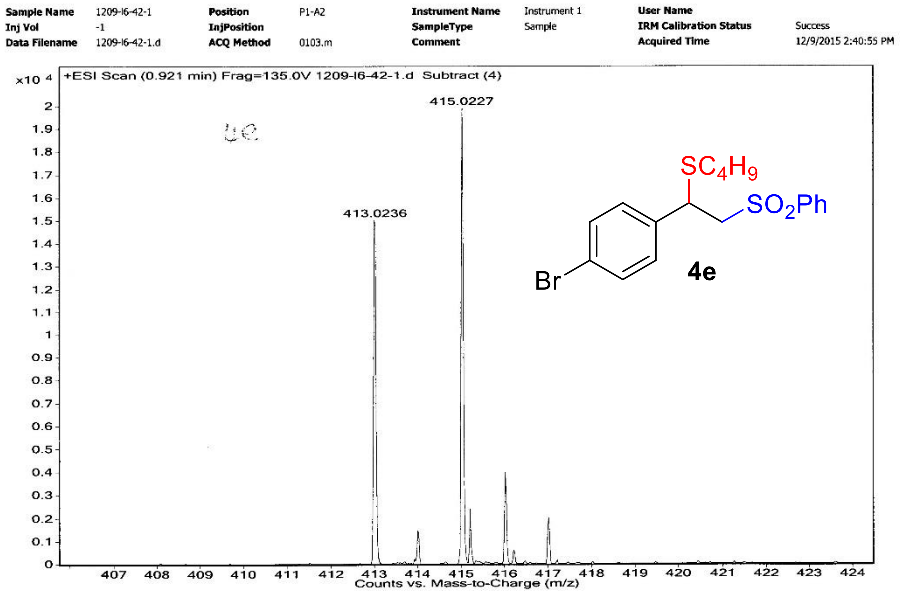

HRMS (ESI, m/z) calcd for $C_{18}H_{21}BrO_2S_2$ $[M+H]^+$ 413.0239, found 413.0236.

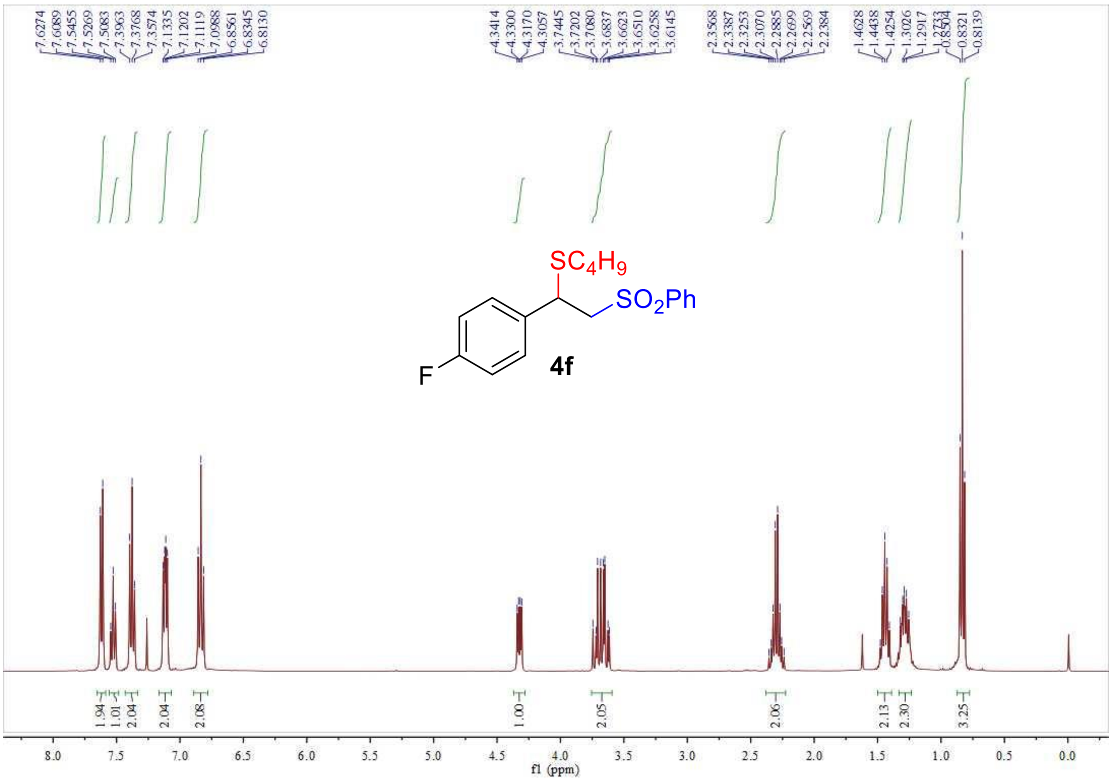
SC4H9
SO2Ph
F
4f
f1 (ppm)

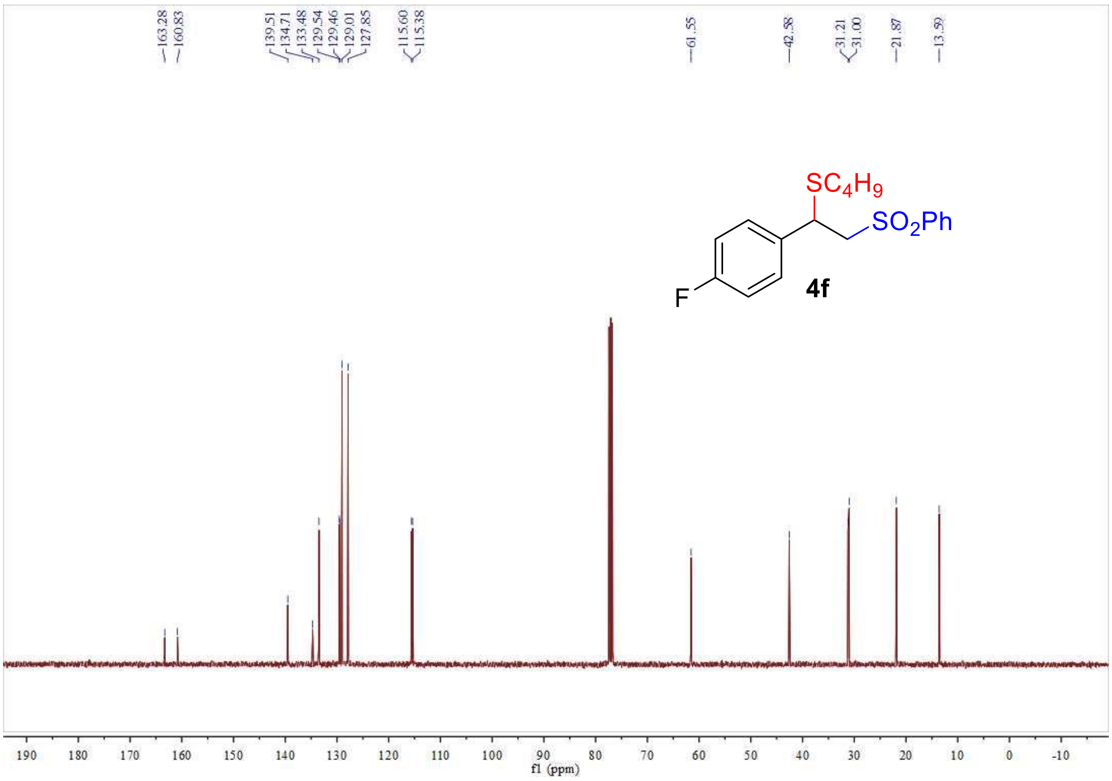
SC4H9
SO2Ph
F
4f
f1 (ppm)

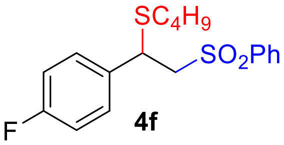
SC4H9
SO2Ph
F

4f

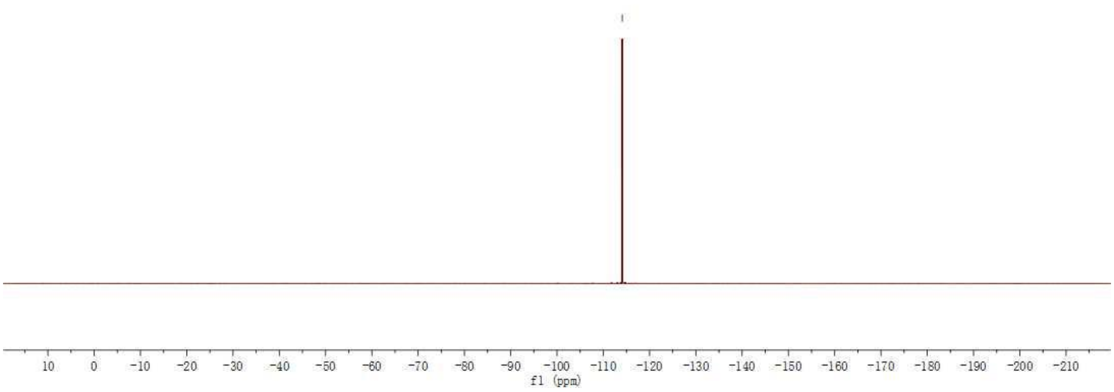
-114.10
10 0 -10 -20 -30 -40 -50 -60 -70 -80 -90 -100 -110 -120 -130 -140 -150 -160 -170 -180 -190 -200 -210
f1 (ppm)

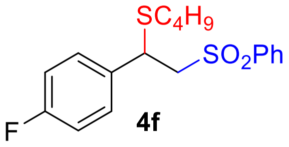

4f

Chemical Formula: $C_{18}H_{21}FO_2S_2$
Exact Mass: 352.0967
Molecular Weight: 352.4824
m/z: 352.0967 (100.0%), 353.1001 (19.5%), 354.0925 (9.0%), 354.1034 (1.8%), 355.0959 (1.8%), 353.0961 (1.6%)
Elemental Analysis: C, 61.34; H, 6.01; F, 5.39; O, 9.08; S, 18.19

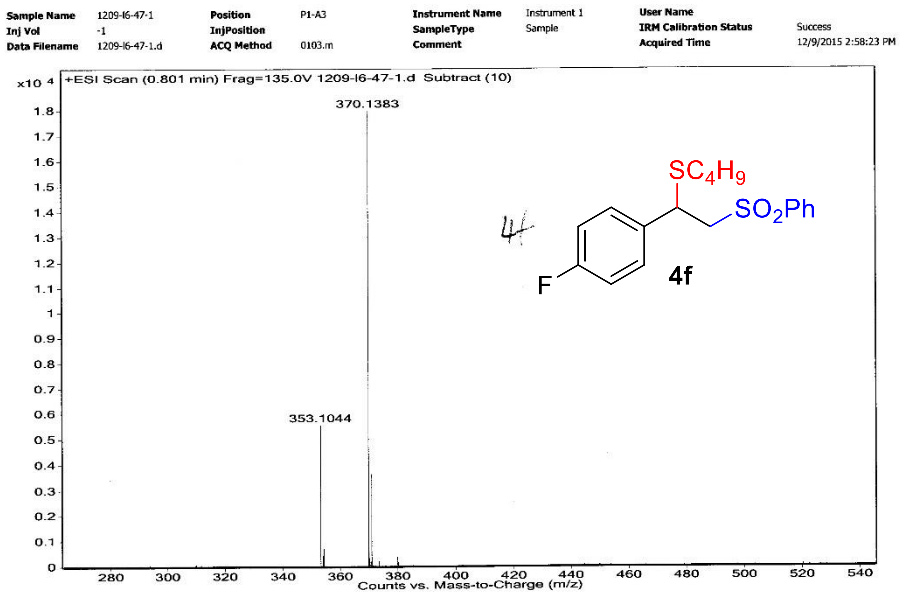

HRMS (ESI, m/z) calcd for $C_{18}H_{21}FO_2S_2$ $[M+H]^+$ 353.1040, found 353.1044.

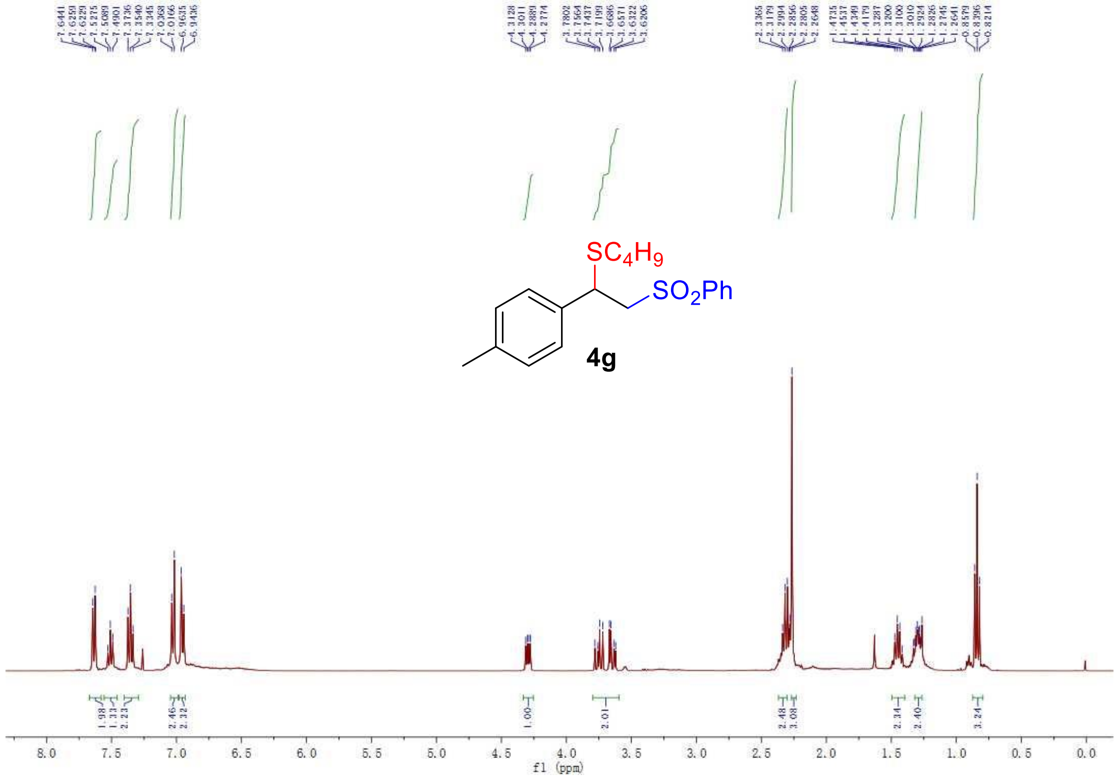
SC4H9
SO2Ph
4g
f1 (ppm)

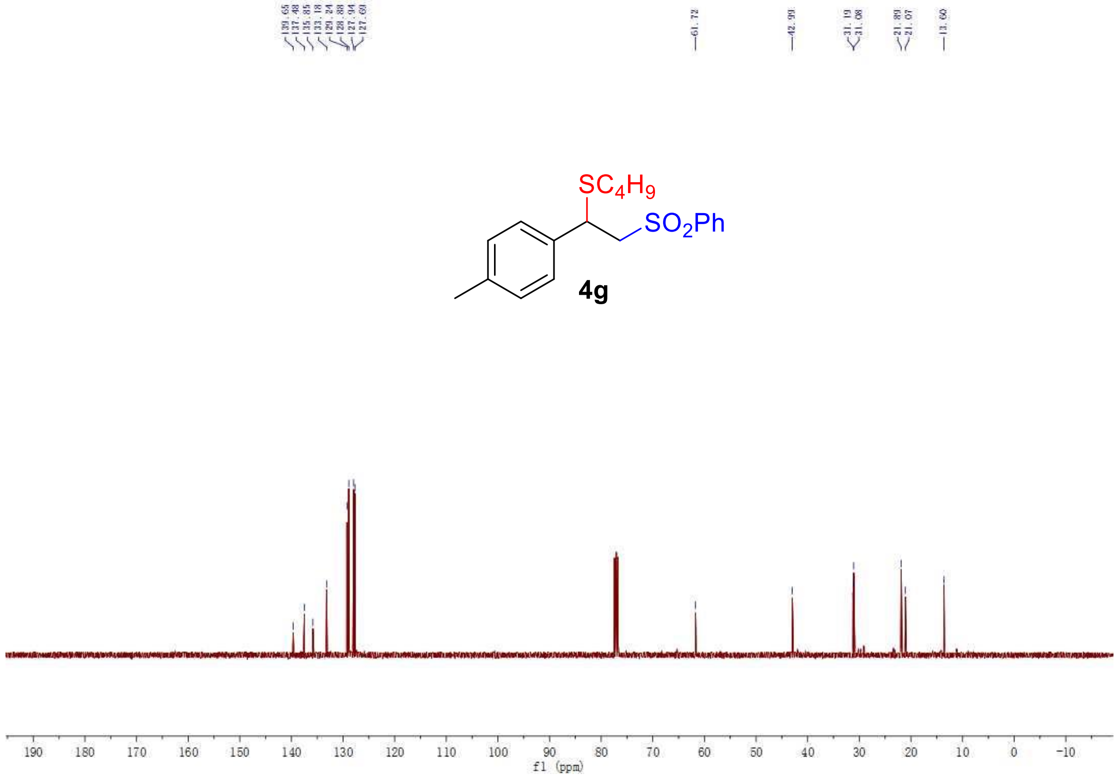
SC4H9
SO2Ph
4g
f1 (ppm)

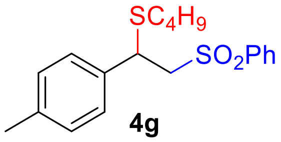

4g

Chemical Formula: $C_{19}H_{24}O_2S_2$
Exact Mass: 348.1218
Molecular Weight: 348.5190
m/z: 348.1218 (100.0%), 349.1251 (20.5%), 350.1176 (9.0%), 350.1285 (2.0%), 351.1209 (1.9%), 349.1212 (1.6%)
Elemental Analysis: C, 65.48; H, 6.94; O, 9.18; S, 18.40

**Sample Name**	2016-0718-L8-31-5	**Position**	P1-D9	**Instrument Name**	Instrument 1	**User Name**	
**Inj Vol**	-1	**InjPosition**		**SampleType**	Sample	**IRM Calibration Status**	Success
**Data Filename**	2016-0718-L8-31-5.d	**ACQ Method**	0103.m	**Comment**		**Acquired Time**	7/18/2016 3:48:20 PM

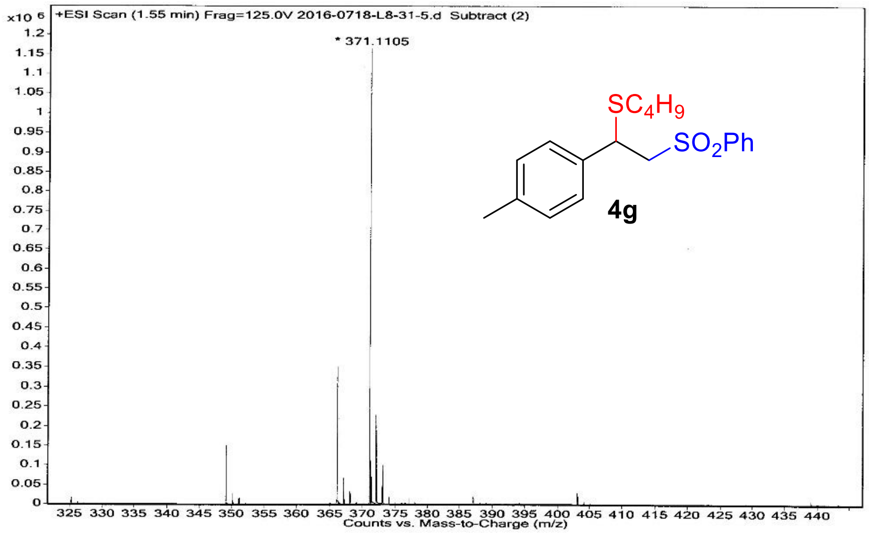

HRMS (ESI, m/z) calcd for $C_{19}H_{24}O_2S_2$ $[M+Na]^+$ 371.1110, found 371.1115.

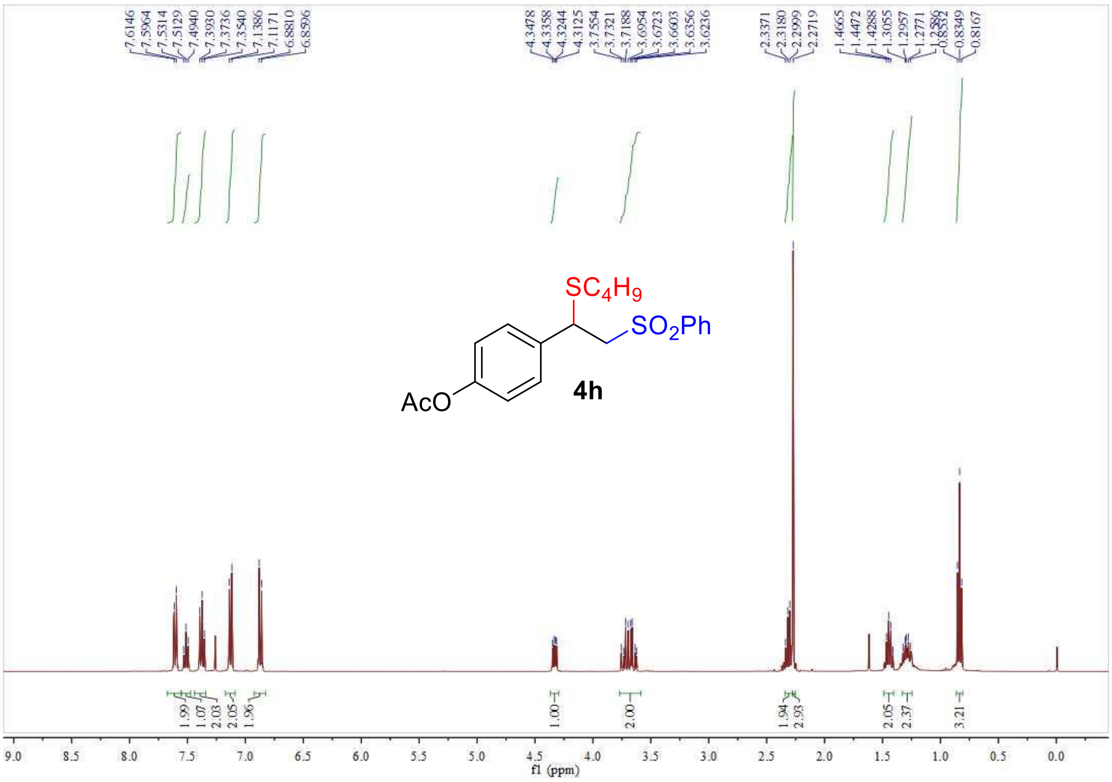
SC4H9
SO2Ph
AcO
4h
f1 (ppm)

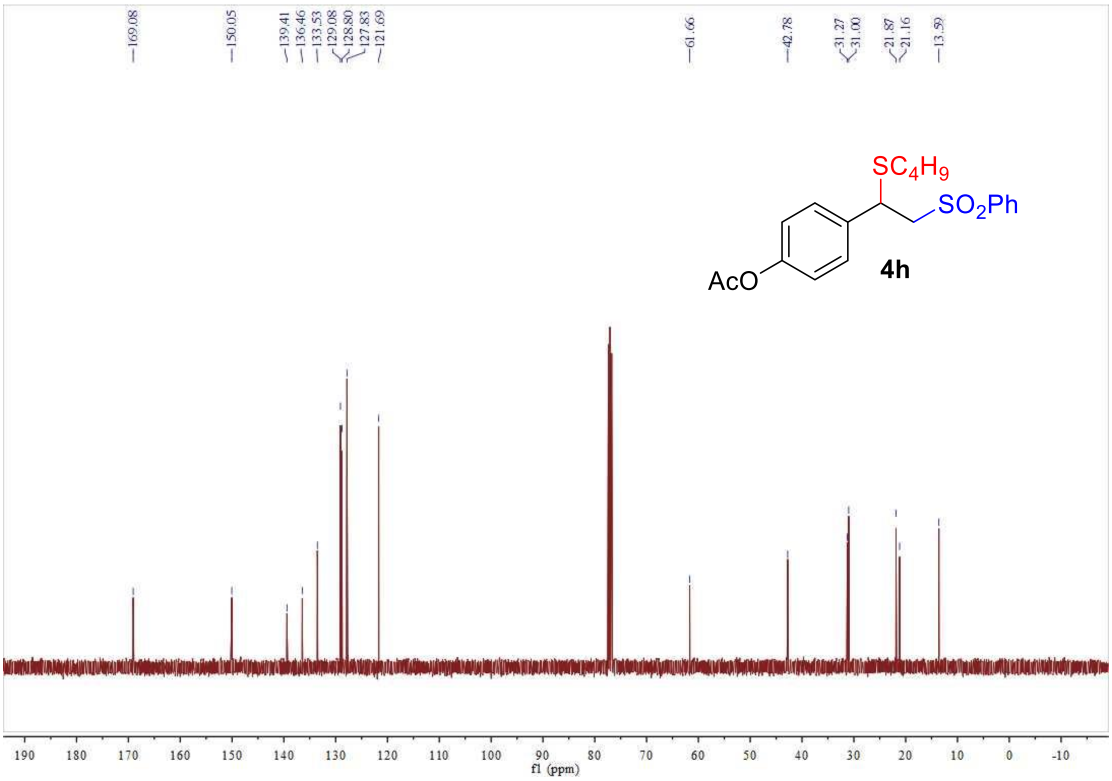
SC4H9
SO2Ph
AcO
4h
f1 (ppm)

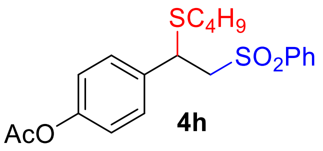

Chemical Formula: $C_{20}H_{24}O_4S_2$
Exact Mass: 392.1116
Molecular Weight: 392.5280
m/z: 392.1116 (100.0%), 393.1150 (21.6%), 394.1074 (9.0%), 394.1183 (2.2%), 395.1108 (2.0%), 393.1110 (1.6%)
Elemental Analysis: C, 61.20; H, 6.16; O, 16.30; S, 16.34

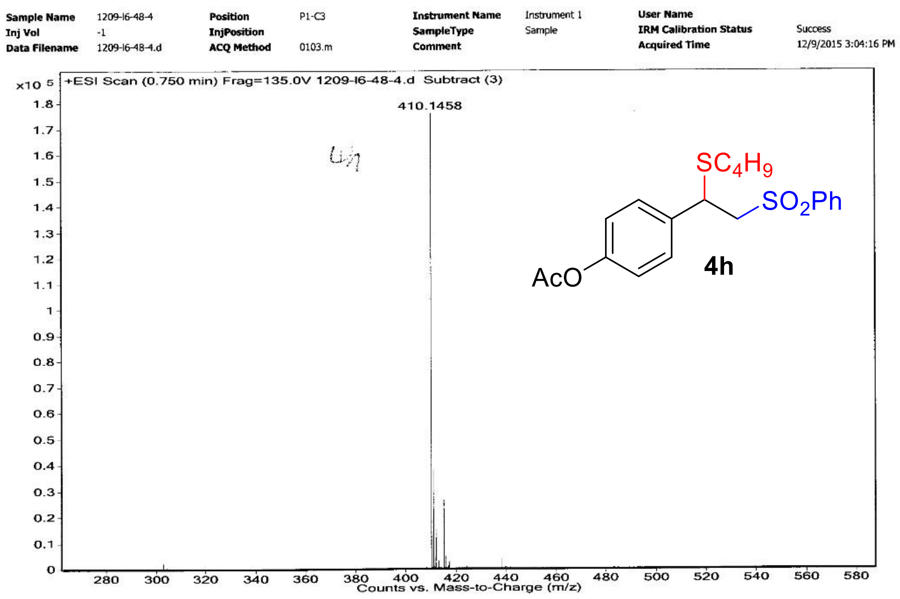

HRMS (ESI, m/z) calcd for $C_{20}H_{24}O_4S_2$ $[M+NH_4]^+$ 410.1454, found 410.1458.

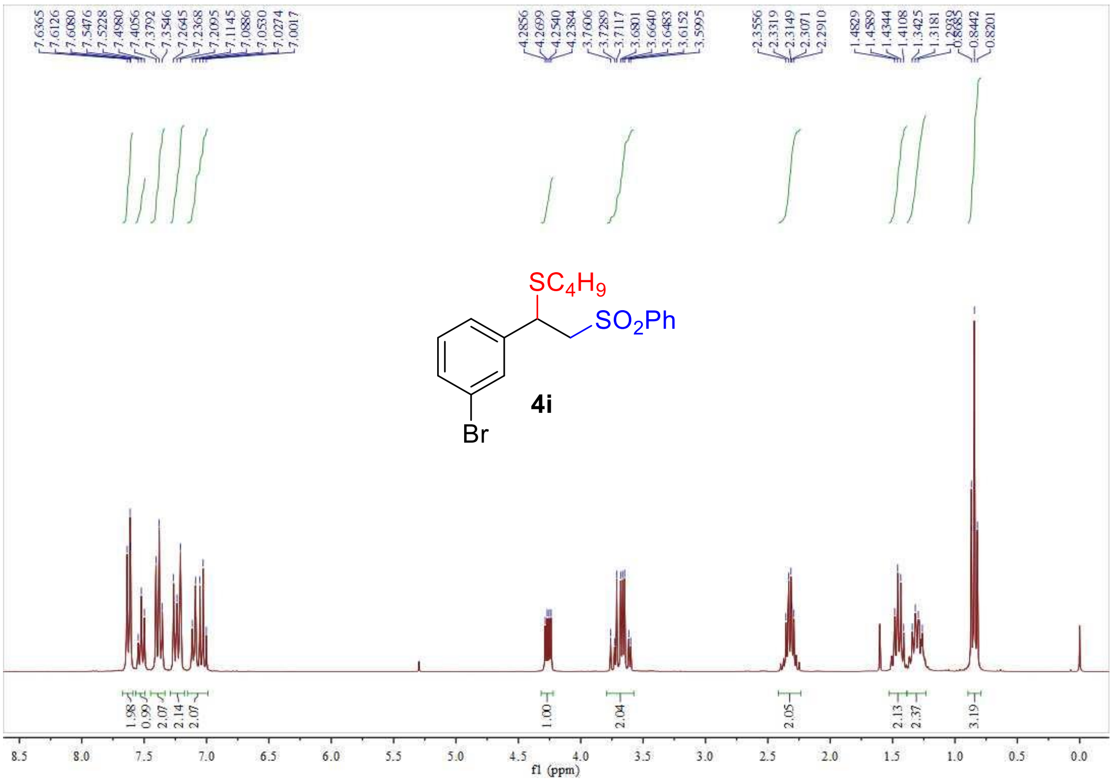

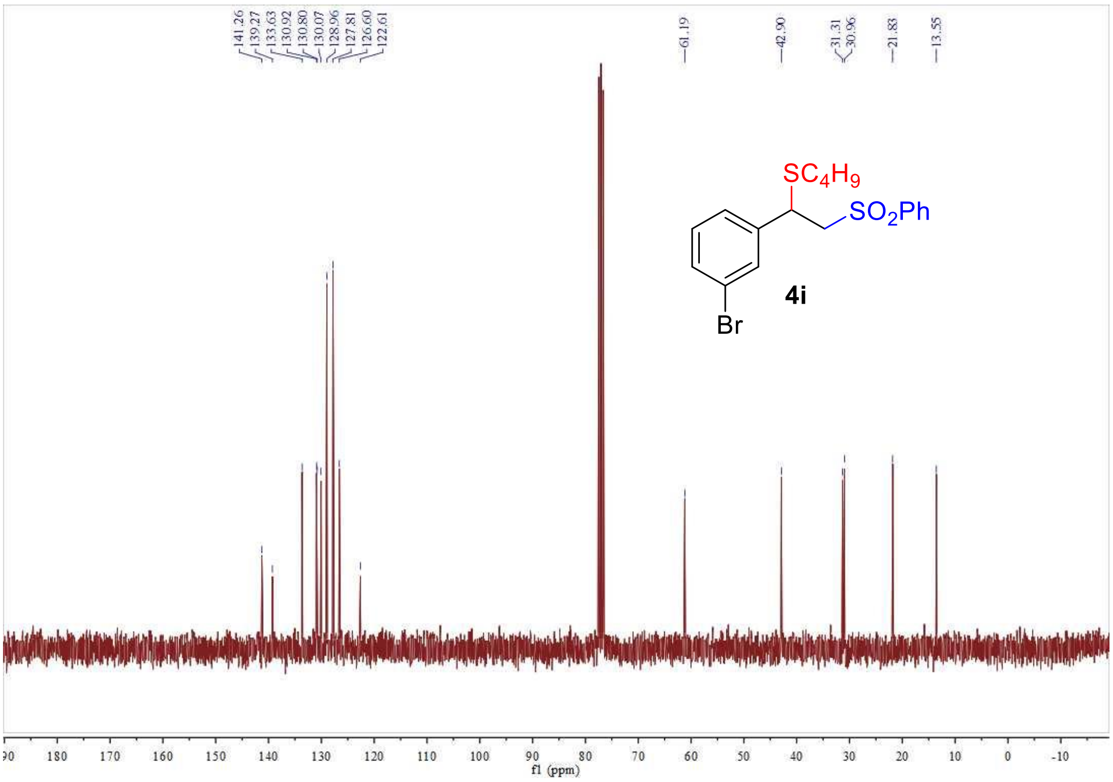

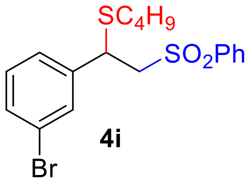

4i

Chemical Formula: $C_{18}H_{21}BrO_2S_2$
Exact Mass: 412.0166
Molecular Weight: 413.3880
m/z: 412.0166 (100.0%), 414.0146 (97.3%), 413.0200 (19.5%), 415.0179 (18.9%), 414.0124 (9.0%), 416.0104 (8.8%), 416.0213 (1.7%), 417.0137 (1.7%), 413.0160 (1.6%), 415.0140 (1.6%), 415.0158 (1.5%), 414.0233 (1.1%)
Elemental Analysis: C, 52.30; H, 5.12; Br, 19.33; O, 7.74; S, 15.51

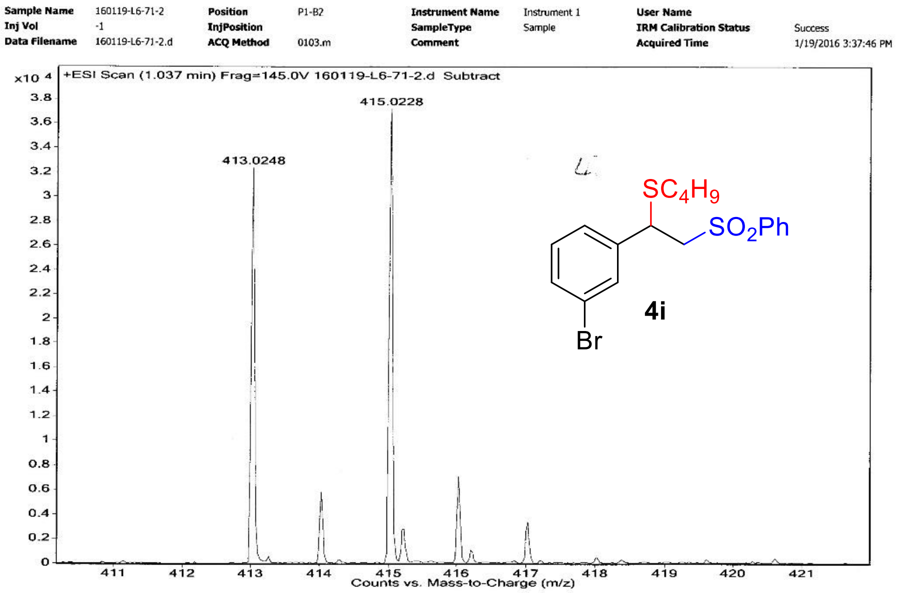

HRMS (ESI, m/z) calcd for $C_{18}H_{21}BrO_2S_2$ $[M+H]^+$ 413.0239, found 413.0248.

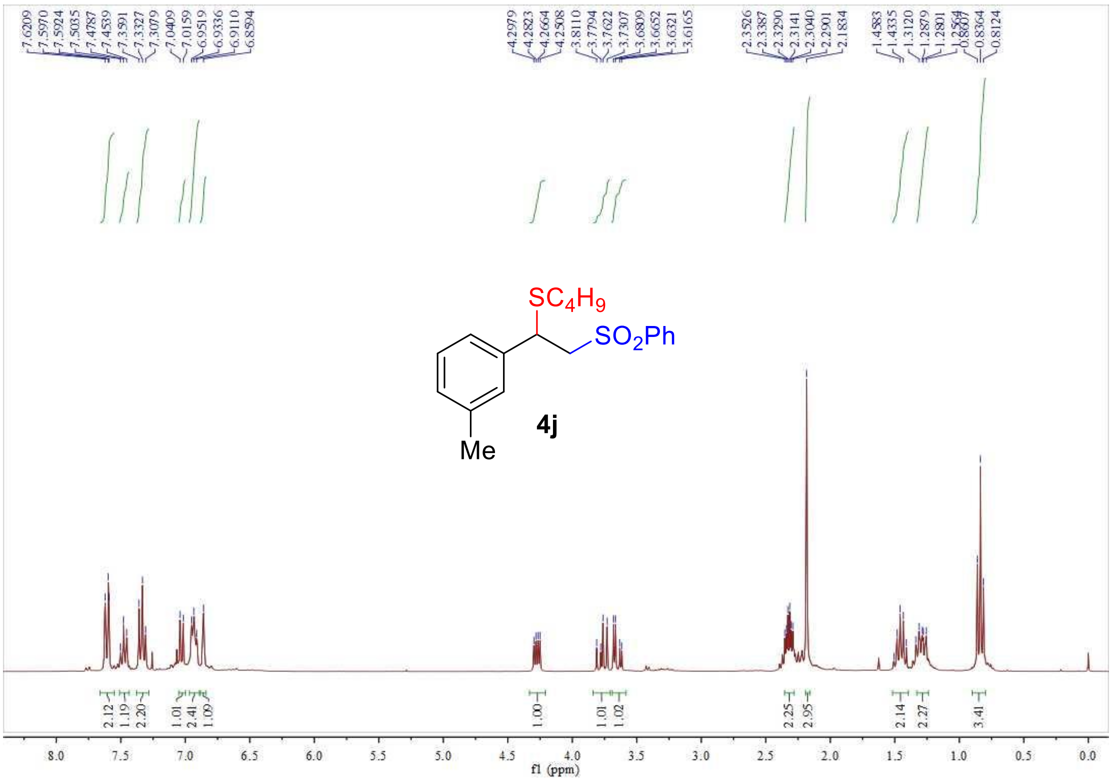
7.6209
7.5970
7.5924
7.5035
7.4787
7.4539
7.3591
7.3327
7.3079
7.0409
7.0159
6.9519
6.9336
6.9110
6.8594
4.2979
4.2823
4.2664
4.2508
3.8110
3.7794
3.7622
3.7307
3.6809
3.6652
3.6321
3.6165
2.3526
2.3387
2.3290
2.3141
2.3040
2.2901
2.1834
1.4583
1.4335
1.3120
1.2879
1.2801
0.8364
0.8124
SC4H9
SO2Ph
Me
4j
2.12
1.19
2.20
1.01
2.41
1.09
1.00
1.01
1.02
2.25
2.95
2.14
2.27
3.41
8.0
7.5
7.0
6.5
6.0
5.5
5.0
4.5
4.0
3.5
3.0
2.5
2.0
1.5
1.0
0.5
0.0
f1 (ppm)

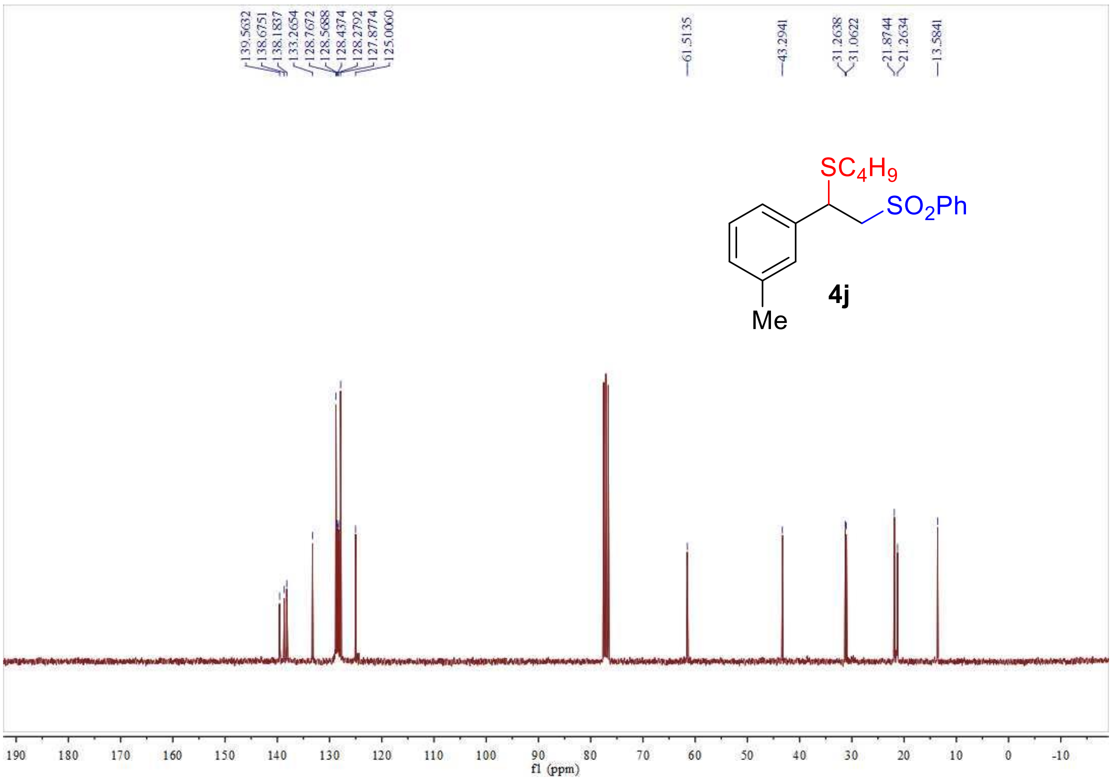
139.5632
138.6751
138.1837
133.2654
128.7672
128.5688
128.4374
128.2792
127.8774
125.0060
61.5135
43.2941
31.2638
31.0622
21.8744
21.2634
13.5841
SC4H9
SO2Ph
Me
4j
190
180
170
160
150
140
130
120
110
100
90
80
70
60
50
40
30
20
10
0
-10
f1 (ppm)

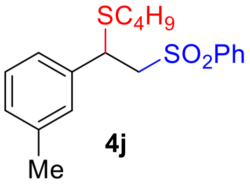

4j

Chemical Formula: $C_{19}H_{24}O_2S_2$
Exact Mass: 348.1218
Molecular Weight: 348.5190
m/z: 348.1218 (100.0%), 349.1251 (20.5%), 350.1176 (9.0%), 350.1285 (2.0%), 351.1209 (1.9%), 349.1212 (1.6%)
Elemental Analysis: C, 65.48; H, 6.94; O, 9.18; S, 18.40

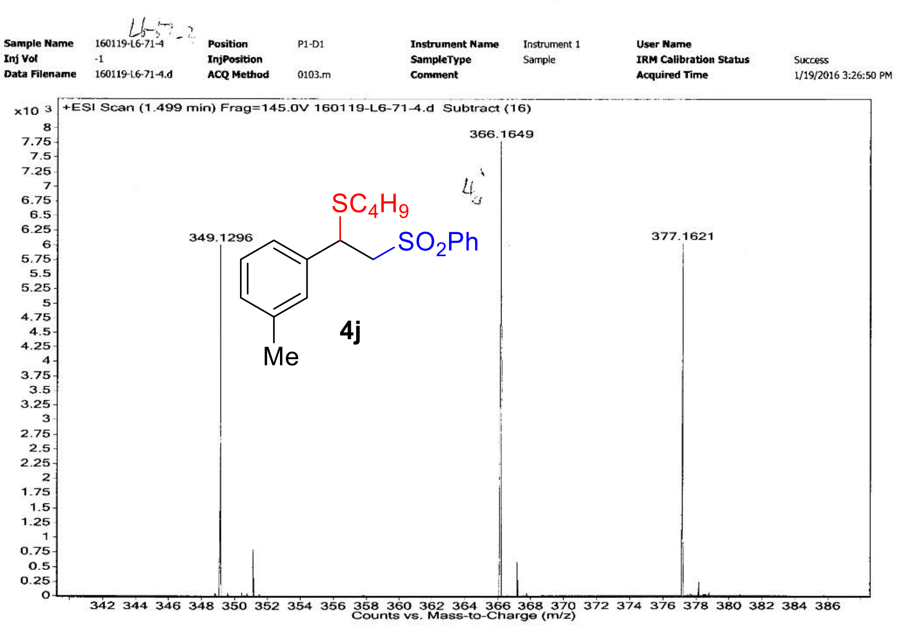

HRMS (ESI, m/z) calcd for $C_{19}H_{24}O_2S_2$ $[M+H]^+$ 349.1290, found 349.1296.

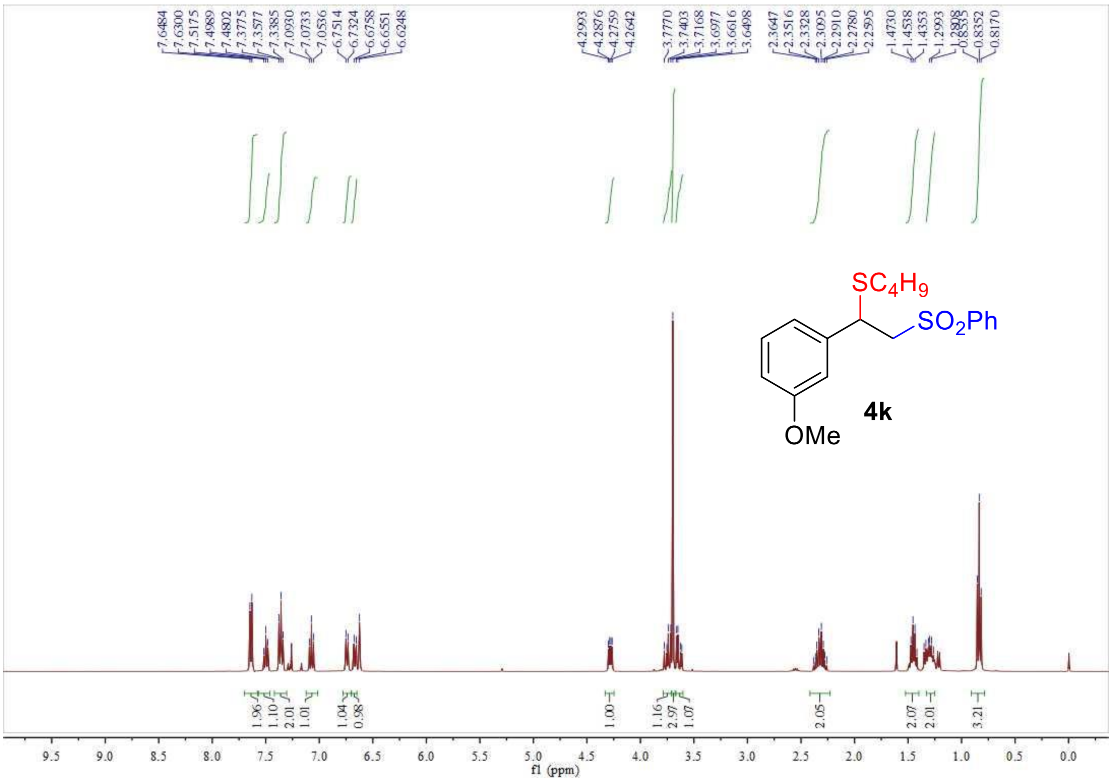
SC4H9
SO2Ph
OMe
4k
f1 (ppm)

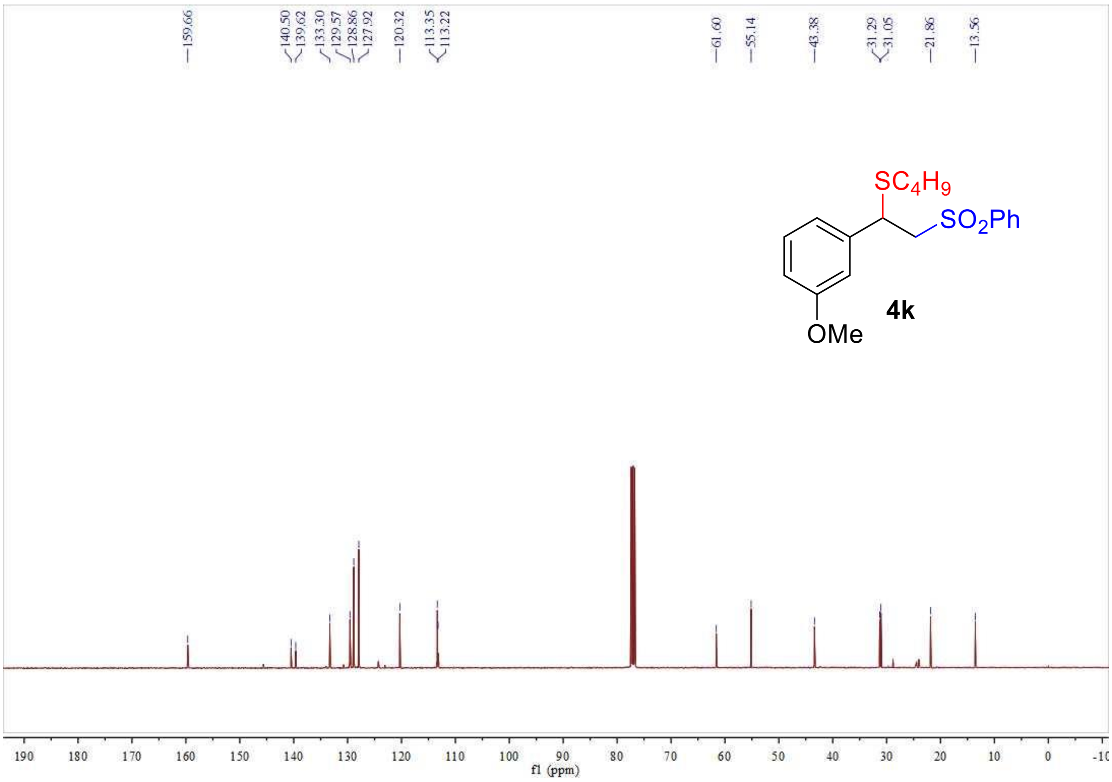
159.66
140.50
139.62
133.30
129.57
128.86
127.92
120.32
113.35
113.22
61.60
55.14
43.38
31.29
31.05
21.86
13.56
SC4H9
SO2Ph
OMe
4k
f1 (ppm)

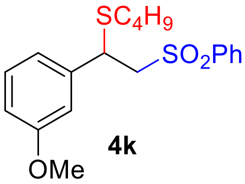

4k

Chemical Formula: $C_{19}H_{24}O_3S_2$
Exact Mass: 364.1167
Molecular Weight: 364.5180
m/z: 364.1167 (100.0%), 365.1200 (20.5%), 366.1125 (9.0%), 366.1234 (2.0%), 367.1158 (1.9%), 365.1161 (1.6%)
Elemental Analysis: C, 62.61; H, 6.64; O, 13.17; S, 17.59

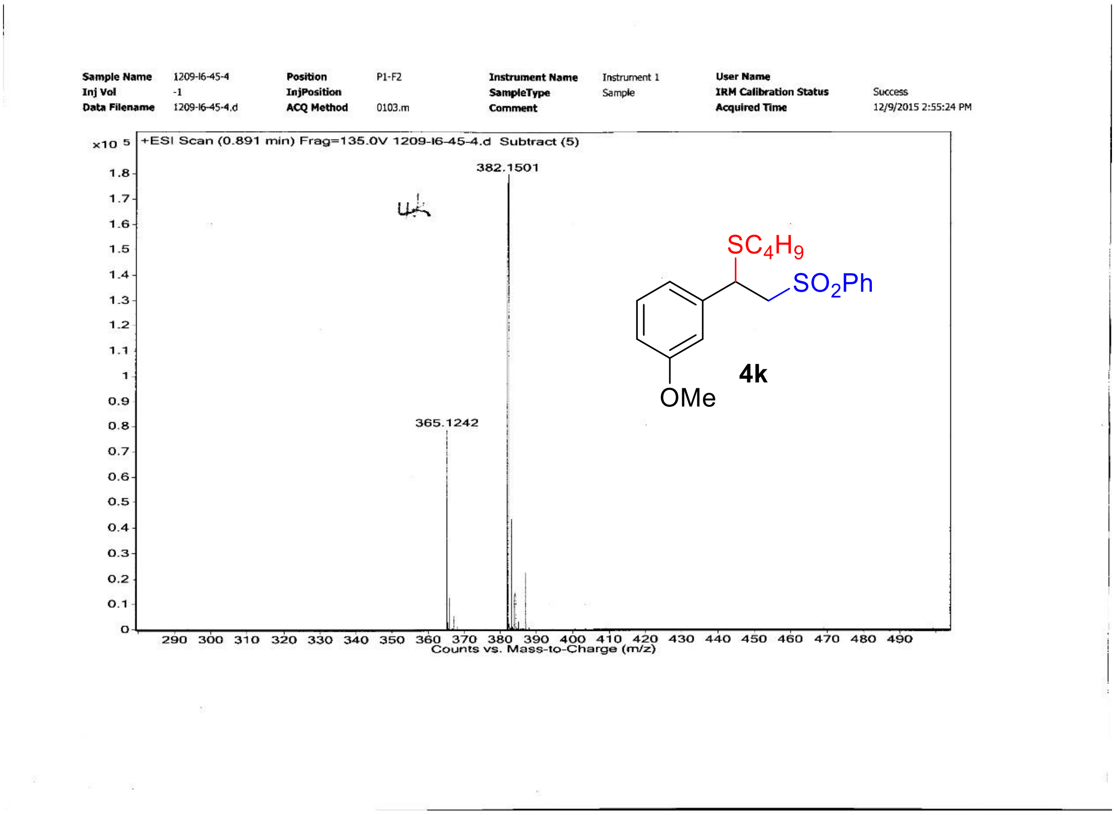

HRMS (ESI, m/z) calcd for $C_{19}H_{24}O_3S_2$ $[M+H]^+$ 365.1240, found 365.1242.

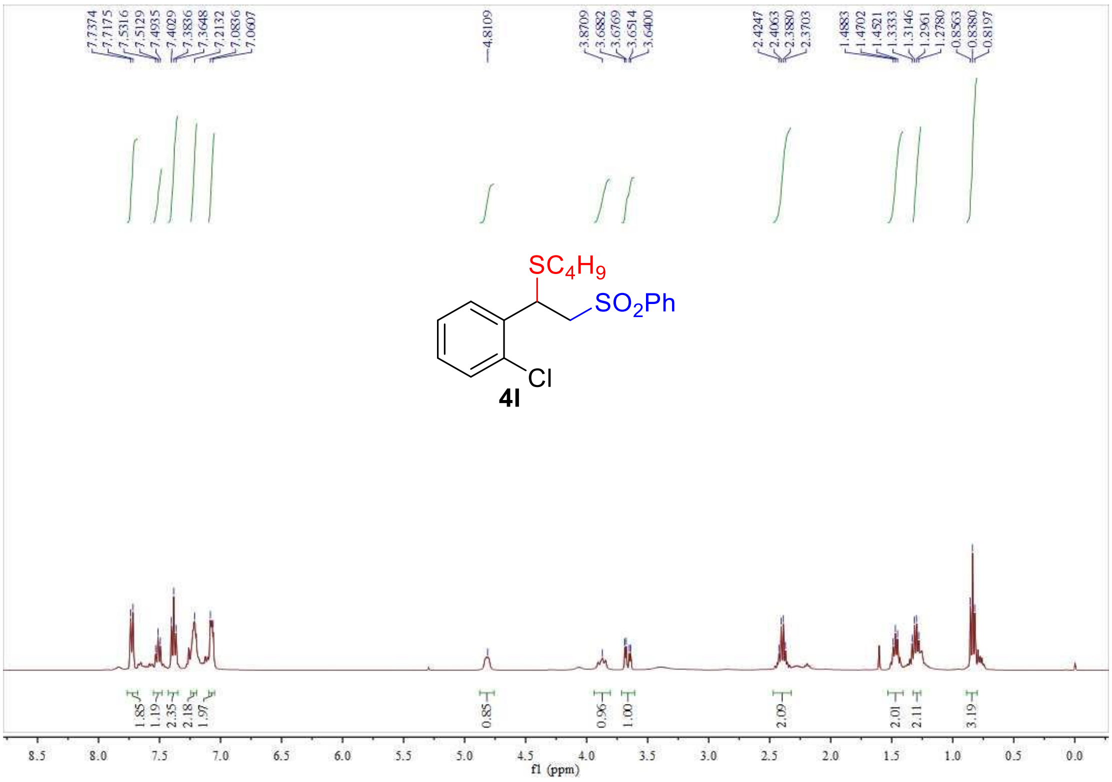
7.7374
7.7175
7.5316
7.5129
7.4935
7.4029
7.3836
7.3648
7.2132
7.0836
7.0607
4.8109
3.8709
3.6882
3.6769
3.6514
3.6400
2.4247
2.4063
2.3880
2.3703
1.4883
1.4702
1.4521
1.3333
1.3146
1.2961
1.2780
0.8563
0.8380
0.8197
SC4H9
SO2Ph
Cl
4l
1.85
1.19
2.35
2.18
1.97
0.85
0.96
1.00
2.09
2.01
2.11
3.19
f1 (ppm)

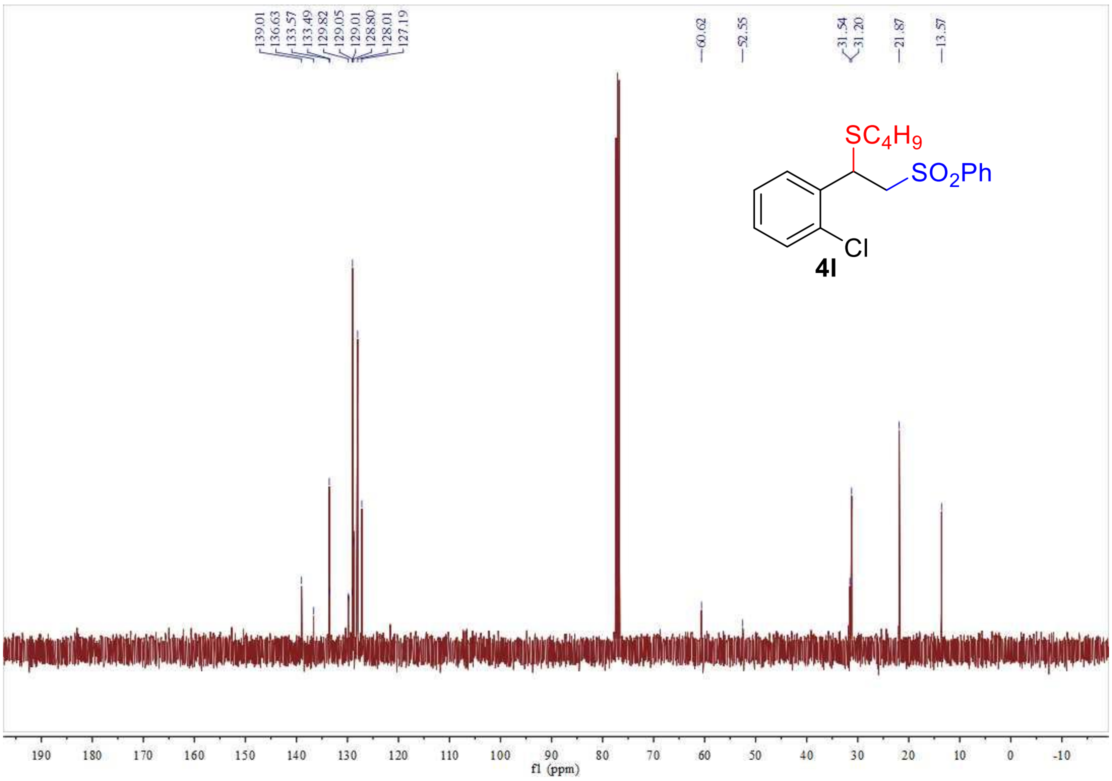
139.01
136.63
133.57
133.49
129.82
129.05
129.01
128.80
128.01
127.19
60.62
52.55
31.54
31.20
21.87
13.57
SC4H9
SO2Ph
Cl
4l
f1 (ppm)

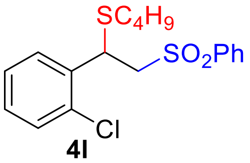

4l

Chemical Formula: $C_{18}H_{21}ClO_2S_2$
Exact Mass: 368.0671
Molecular Weight: 368.9340
m/z: 368.0671 (100.0%), 370.0642 (32.0%), 369.0705 (19.5%), 370.0629 (9.0%), 371.0676 (6.2%), 372.0600 (2.9%), 370.0739 (1.8%), 371.0663 (1.8%), 369.0665 (1.6%)
Elemental Analysis: C, 58.60; H, 5.74; Cl, 9.61; O, 8.67; S, 17.38

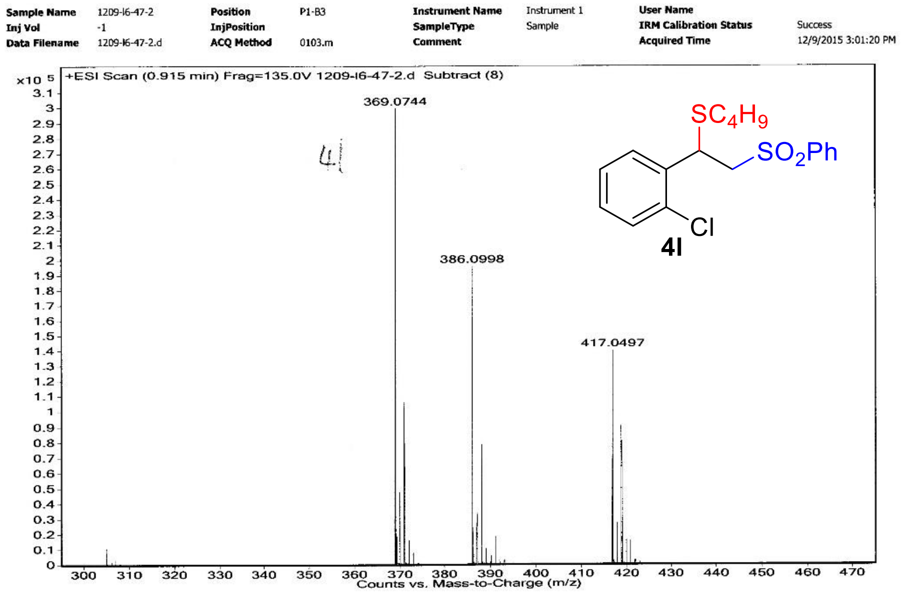

HRMS (ESI, m/z) calcd for $C_{18}H_{21}ClO_2S_2$ $[M+H]^+$ 369.0744, found 369.0744.

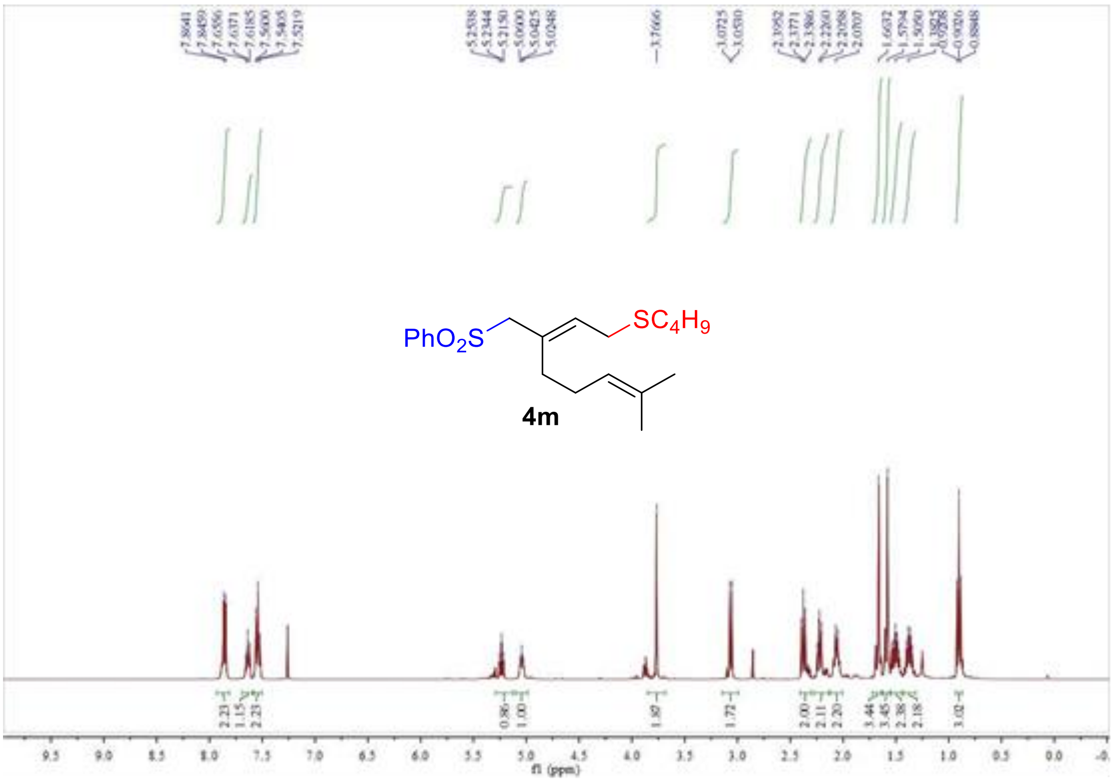

PhO2S
SC4H9
4m
f1 (ppm)

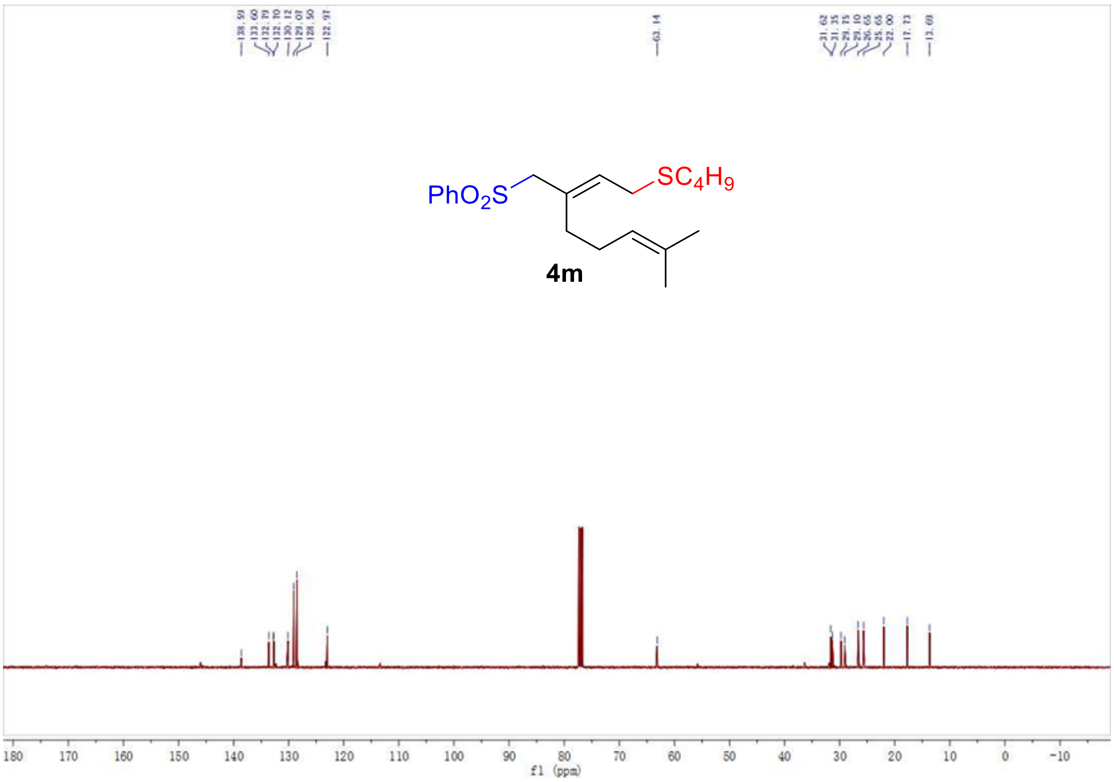

PhO2S
SC4H9
4m
f1 (ppm)

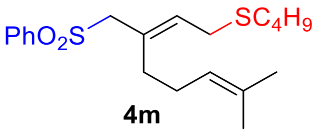

4m

Chemical Formula: $C_{20}H_{30}O_2S_2$
Exact Mass: 366.1687
Molecular Weight: 366.5780
m/z: 366.1687 (100.0%), 367.1721 (21.6%), 368.1645 (9.0%), 368.1754 (2.2%), 369.1679 (2.0%), 367.1681 (1.6%)
Elemental Analysis: C, 65.53; H, 8.25; O, 8.73; S, 17.49

Sample Name	2016-0309-L7-55-1	Position	P1-B9	Instrument Name	Instrument 1	User Name	
Inj Vol	-1	InjPosition		SampleType	Sample	IRM Calibration Status	Success
Data Filename	2016-0309-L7-55-1.d	ACQ Method	0103.m	Comment		Acquired Time	3/6/2016 2:58:27 PM

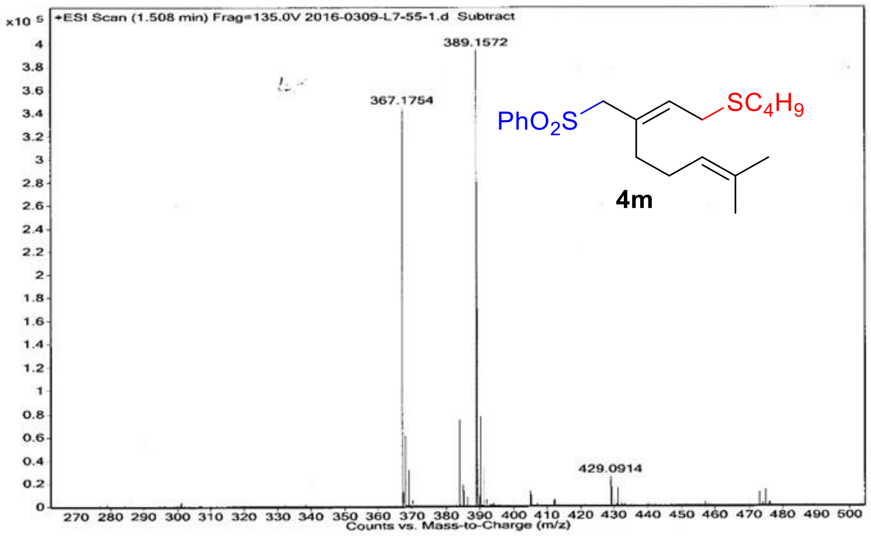

HRMS (ESI, m/z) calcd for C20H30O2S2 [M+H]$^+$ 367.1760, found 367.1754.

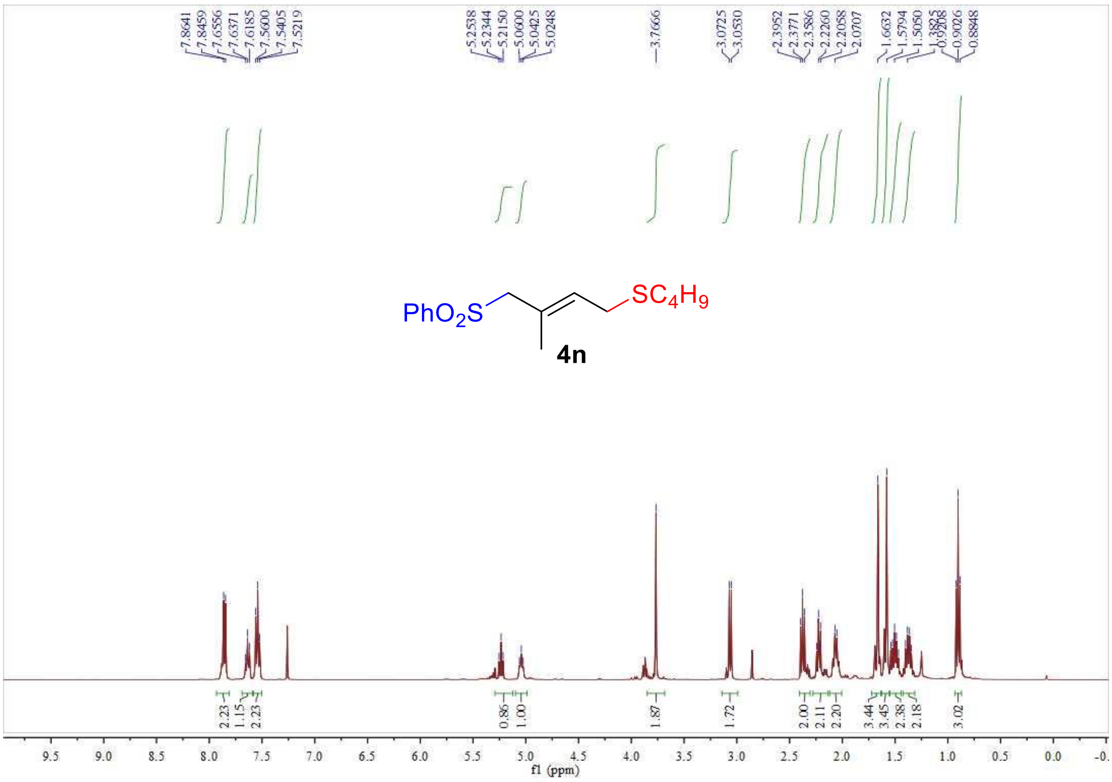
7.8641
7.8459
7.6556
7.6371
7.6185
7.5600
7.5405
7.5219
5.2538
5.2344
5.2150
5.0600
5.0425
5.0248
3.7666
3.0725
3.0530
2.3952
2.3771
2.3586
2.2260
2.2058
2.0707
1.6632
1.5794
1.5050
0.9208
0.9026
0.8848
PhO2S
SC4H9
4n
2.23
1.15
2.23
0.86
1.00
1.87
1.72
2.00
2.11
2.20
3.44
3.45
2.38
2.18
3.02
f1 (ppm)

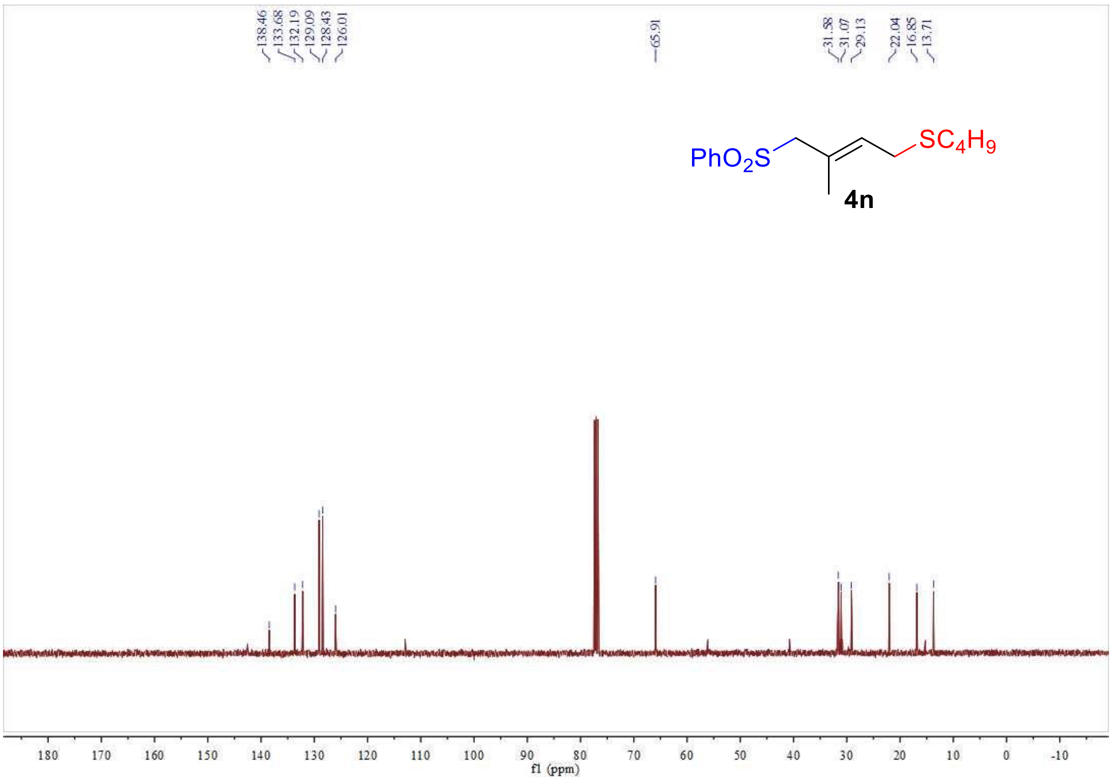
138.46
133.68
132.19
129.09
128.43
126.01
65.91
31.58
31.07
29.13
22.04
16.85
13.71
PhO2S
SC4H9
4n
f1 (ppm)

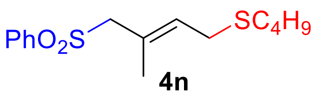

4n

Chemical Formula: $C_{15}H_{22}O_2S_2$
Exact Mass: 298.1061
Molecular Weight: 298.4590
m/z: 298.1061 (100.0%), 299.1095 (16.2%), 300.1019 (9.0%), 299.1055 (1.6%), 300.1128 (1.2%)
Elemental Analysis: C, 60.37; H, 7.43; O, 10.72; S, 21.48

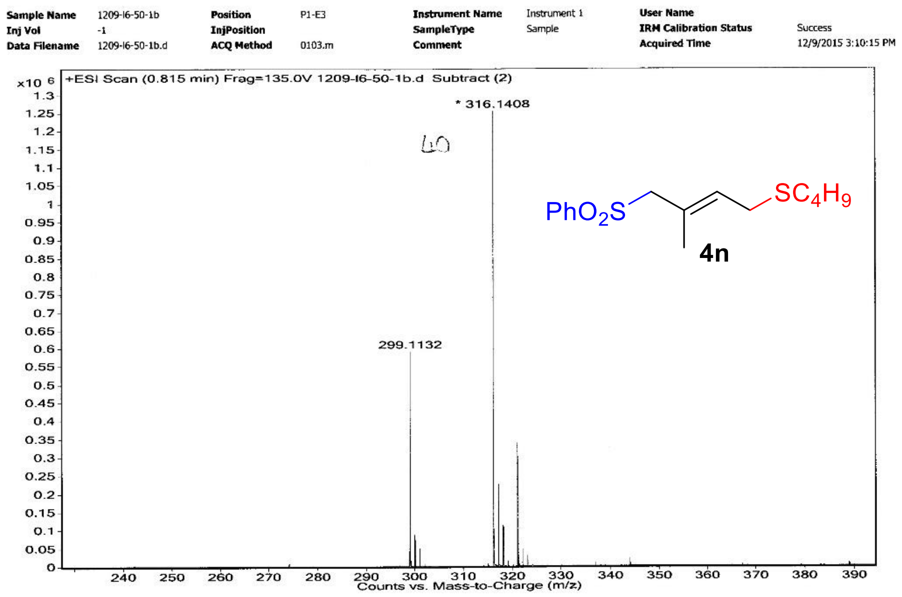

HRMS (ESI, m/z) calcd for $C_{15}H_{22}O_2S_2$ $[M+H]^+$ 299.1134, found 299.1132.

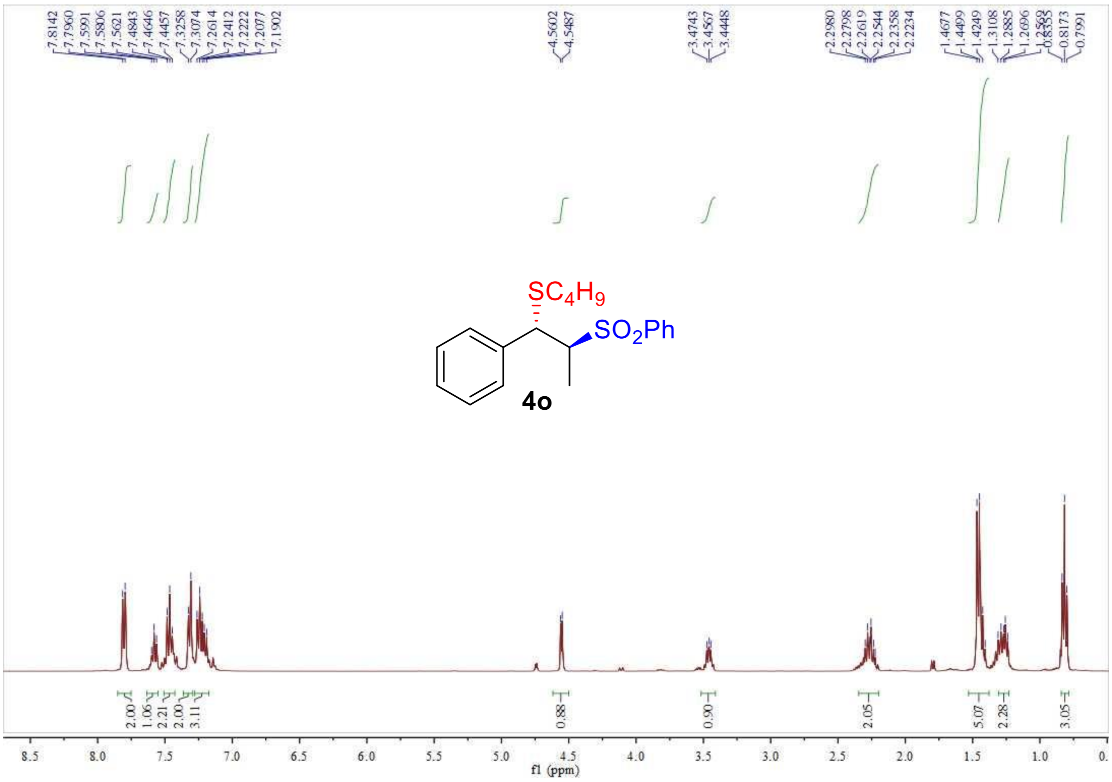
7.8142
7.7960
7.5991
7.5806
7.5621
7.4843
7.4646
7.4457
7.3258
7.3074
7.2614
7.2412
7.2222
7.2077
7.1902
4.5602
4.5487
3.4743
3.4567
3.4448
2.2980
2.2798
2.2619
2.2544
2.2358
2.2234
1.4677
1.4499
1.4249
1.3108
1.2885
1.2696
0.8173
0.7991
SC4H9
SO2Ph
4o
2.00
1.06
2.21
2.00
3.11
0.88
0.90
2.05
5.07
2.28
3.05
f1 (ppm)

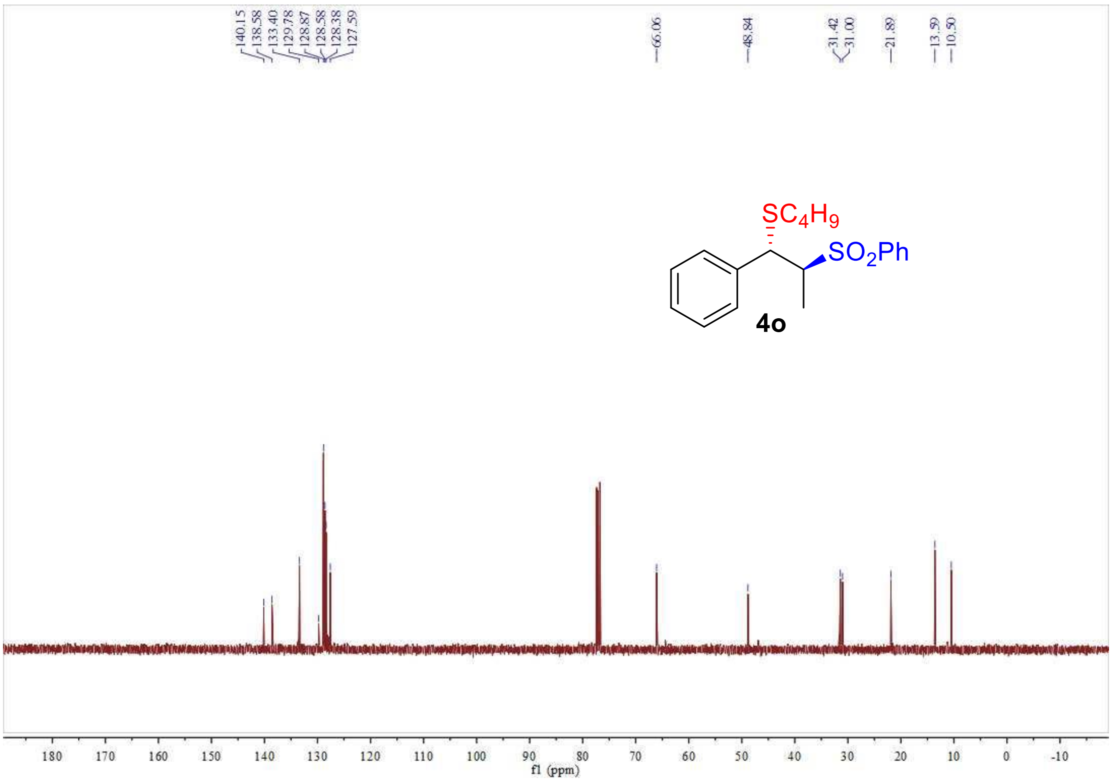
140.15
138.58
133.40
129.78
128.87
128.58
128.38
127.59
66.06
48.84
31.42
31.00
21.89
13.59
10.50
SC4H9
SO2Ph
4o
f1 (ppm)

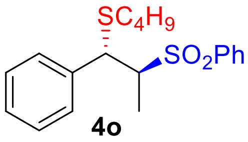

Chemical Formula: $C_{19}H_{24}O_2S_2$

Exact Mass: 348.1218

Molecular Weight: 348.5190

m/z: 348.1218 (100.0%), 349.1251 (20.5%), 350.1176 (9.0%), 350.1285 (2.0%), 351.1209 (1.9%), 349.1212 (1.6%)

Elemental Analysis: C, 65.48; H, 6.94; O, 9.18; S, 18.40

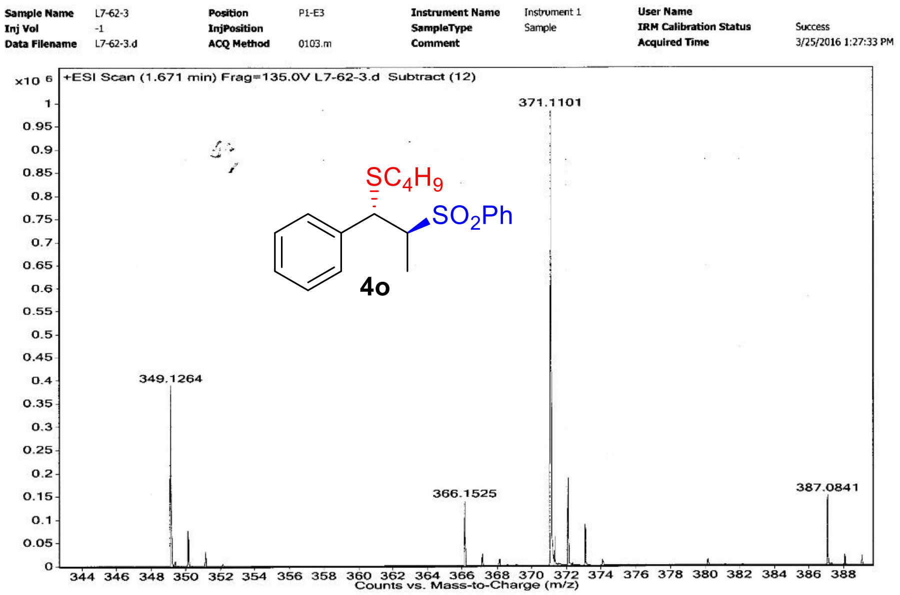

HRMS (ESI, m/z) calcd for $C_{19}H_{24}O_2S_2$ $[M+Na]^+$ 371.1110, found 371.1110.

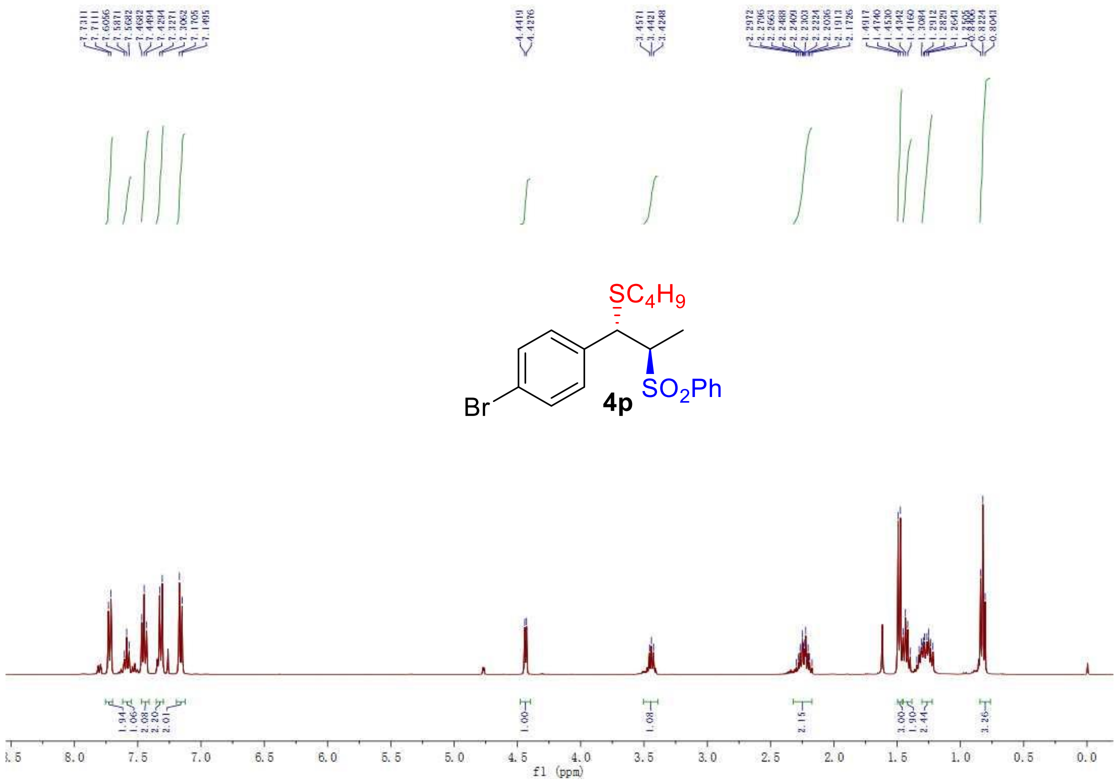

SC4H9
Br
4p
SO2Ph
f1 (ppm)

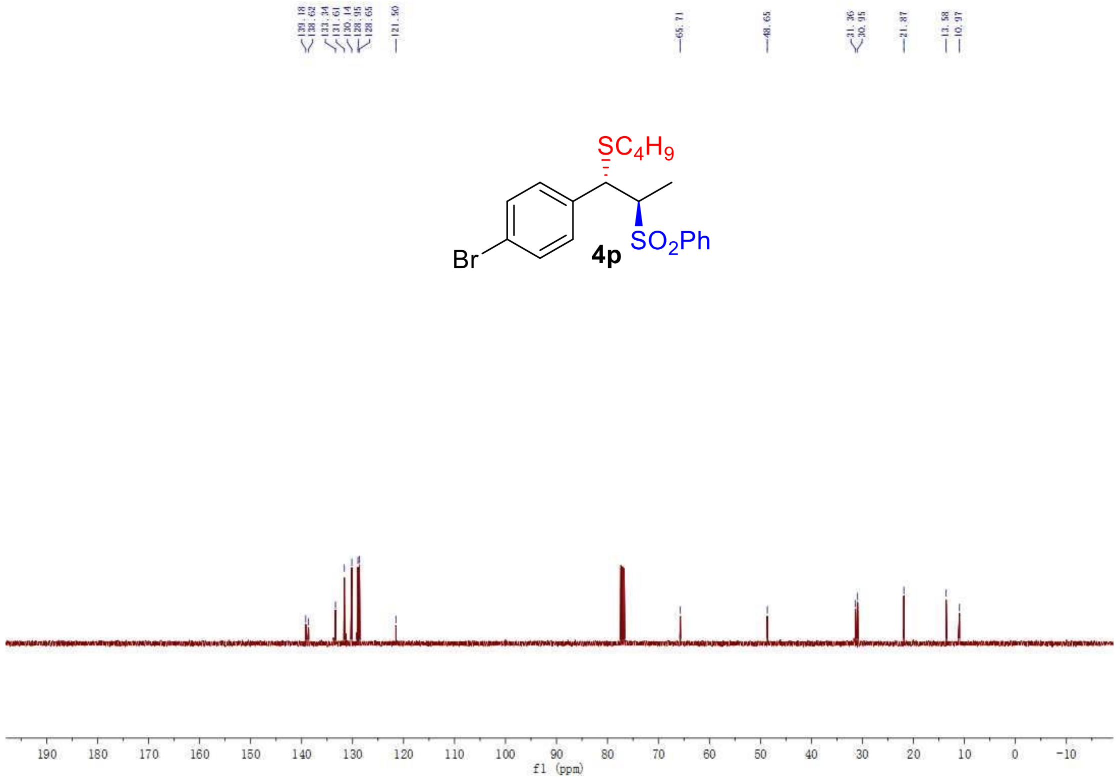

139.18
138.62
133.34
131.61
130.14
128.95
128.65
121.50
65.71
48.65
31.36
30.95
21.87
13.58
10.97
SC4H9
Br
4p
SO2Ph
f1 (ppm)

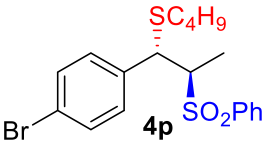

Chemical Formula: $C_{19}H_{23}BrO_2S_2$
Exact Mass: 426.0323
Molecular Weight: 427.4150
m/z: 426.0323 (100.0%), 428.0302 (97.3%), 429.0336 (20.0%), 427.0356 (16.2%), 428.0281 (9.0%), 430.0260 (8.8%), 427.0356 (4.3%), 431.0294 (1.8%), 427.0317 (1.6%), 429.0296 (1.6%), 429.0314 (1.5%), 430.0369 (1.2%), 428.0390 (1.1%)
Elemental Analysis: C, 53.39; H, 5.42; Br, 18.69; O, 7.49; S, 15.00

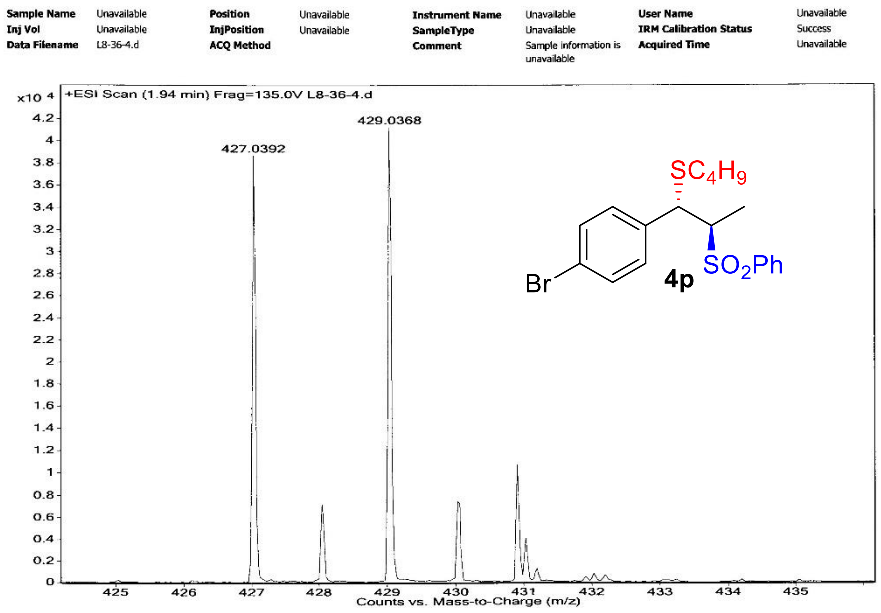

HRMS (ESI, m/z) calcd for $C_{19}H_{23}BrO_2S_2$ $[M+H]^+$ 427.0396, found 427.0392.

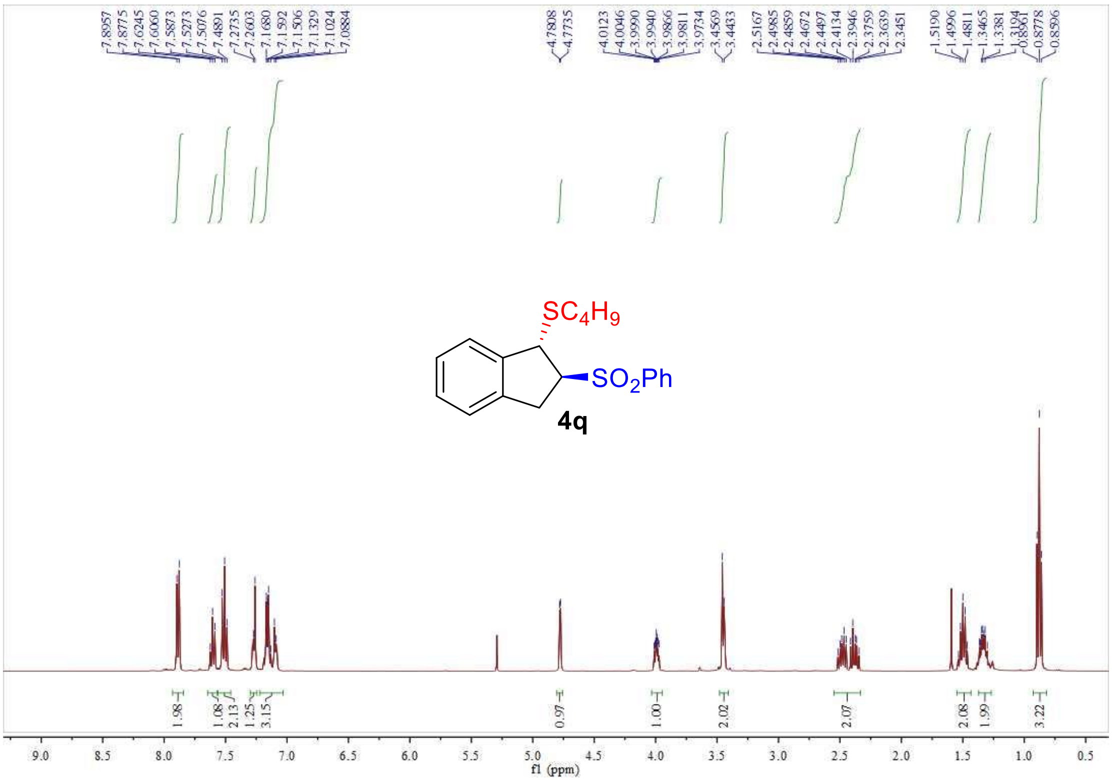
SC4H9
SO2Ph
4q
f1 (ppm)

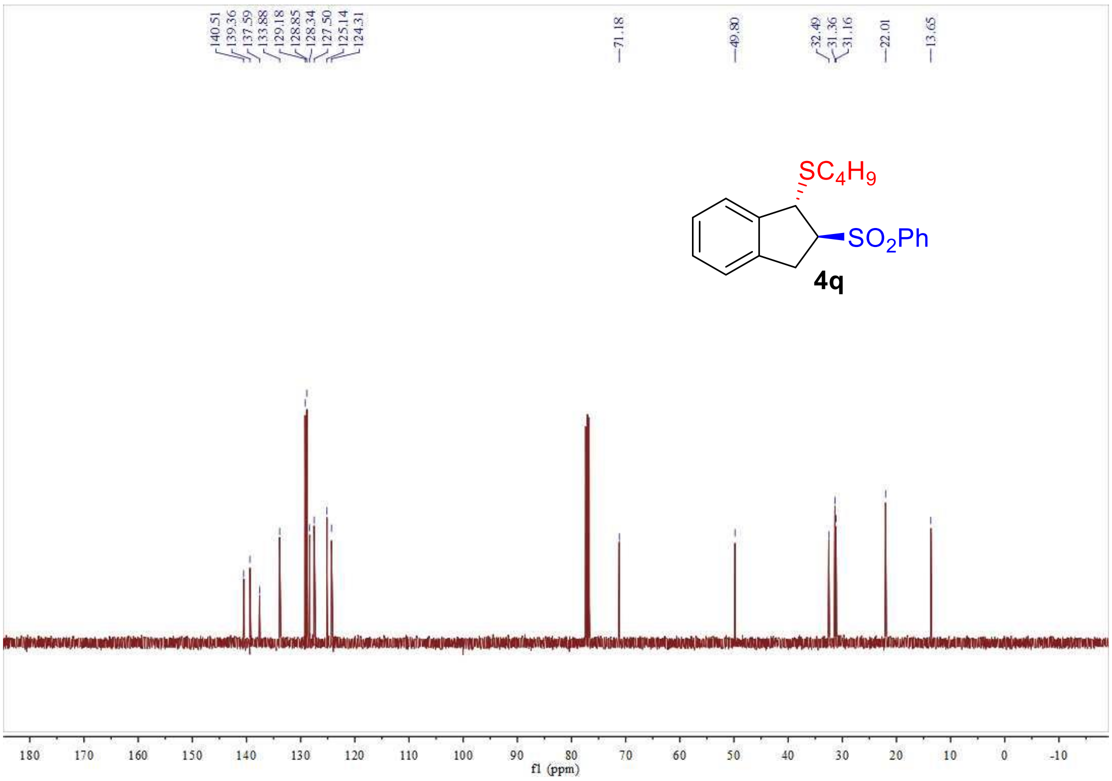
SC4H9
SO2Ph
4q
f1 (ppm)

Chemical Formula: $C_{19}H_{22}O_2S_2$
Exact Mass: 346.1061
Molecular Weight: 346.5030
m/z: 346.1061 (100.0%), 347.1095 (20.5%), 348.1019 (9.0%), 348.1128 (2.0%), 349.1053 (1.9%), 347.1055 (1.6%)
Elemental Analysis: C, 65.86; H, 6.40; O, 9.23; S, 18.50

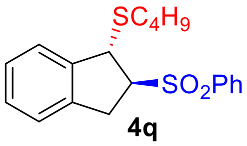

4q

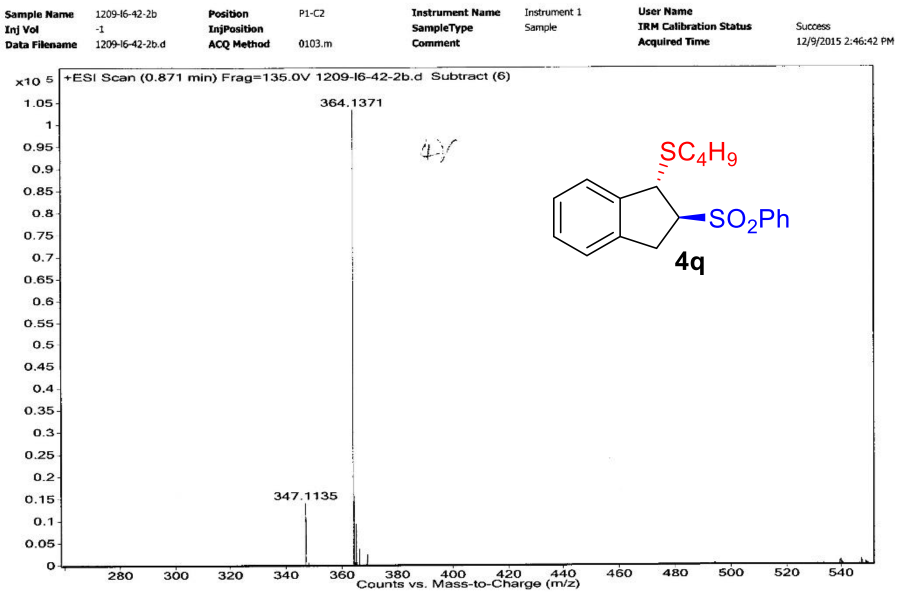

HRMS (ESI, m/z) calcd for $C_{19}H_{22}O_2S_2$ $[M+H]^+$ 347.1134, found 347.1135.

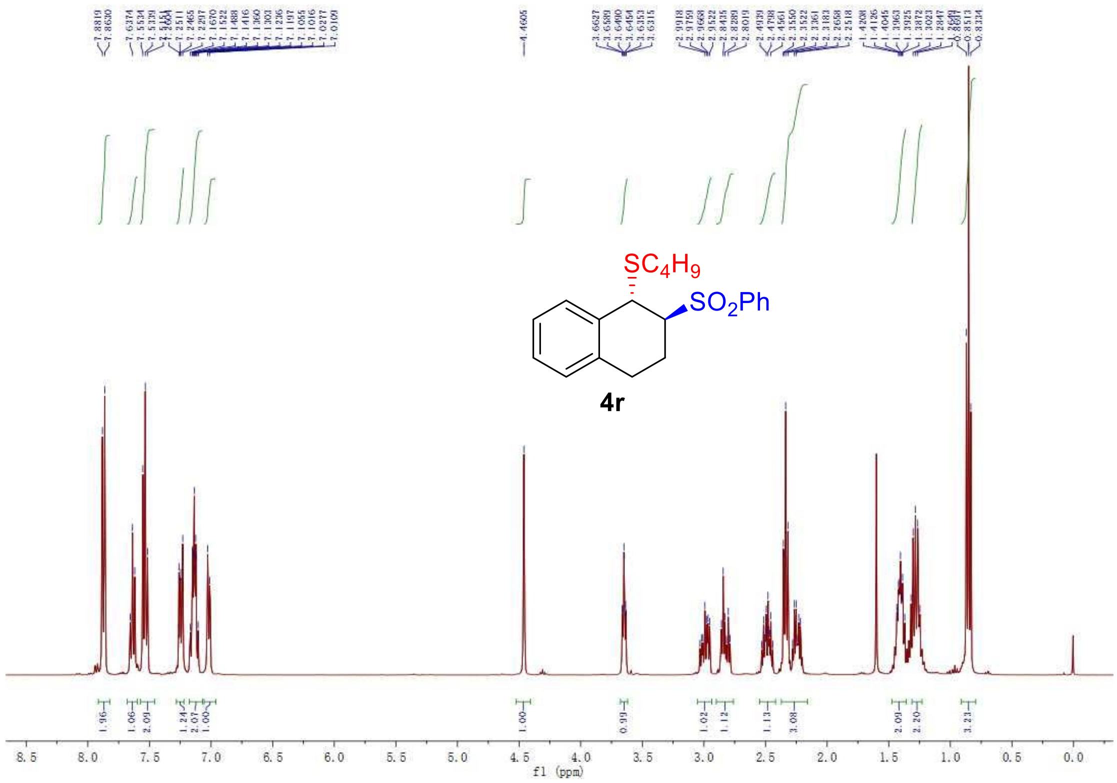
SC4H9
SO2Ph
4r
f1 (ppm)

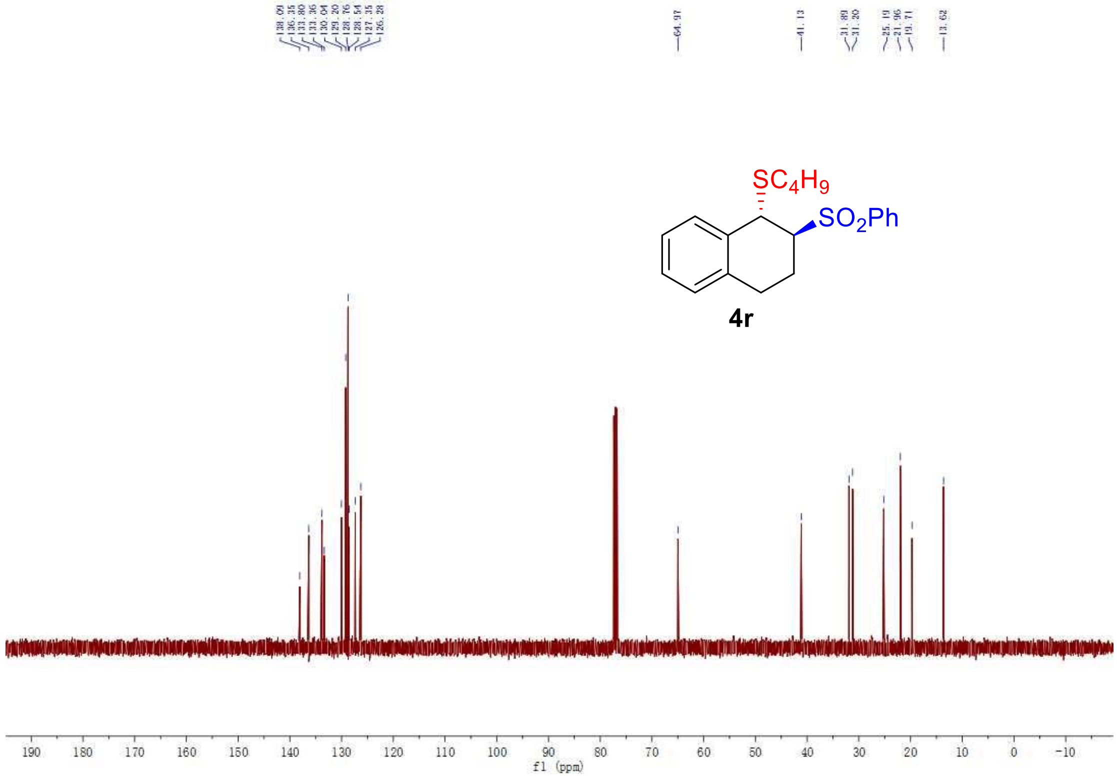
SC4H9
SO2Ph
4r
f1 (ppm)

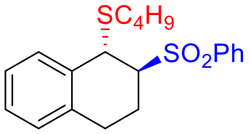

4r

Chemical Formula: $C_{20}H_{24}O_2S_2$
Exact Mass: 360.1218
Molecular Weight: 360.5300
m/z: 360.1218 (100.0%), 361.1251 (21.6%), 362.1176 (9.0%), 362.1285 (2.2%), 363.1209 (2.0%), 361.1212 (1.6%)
Elemental Analysis: C, 66.63; H, 6.71; O, 8.88; S, 17.78

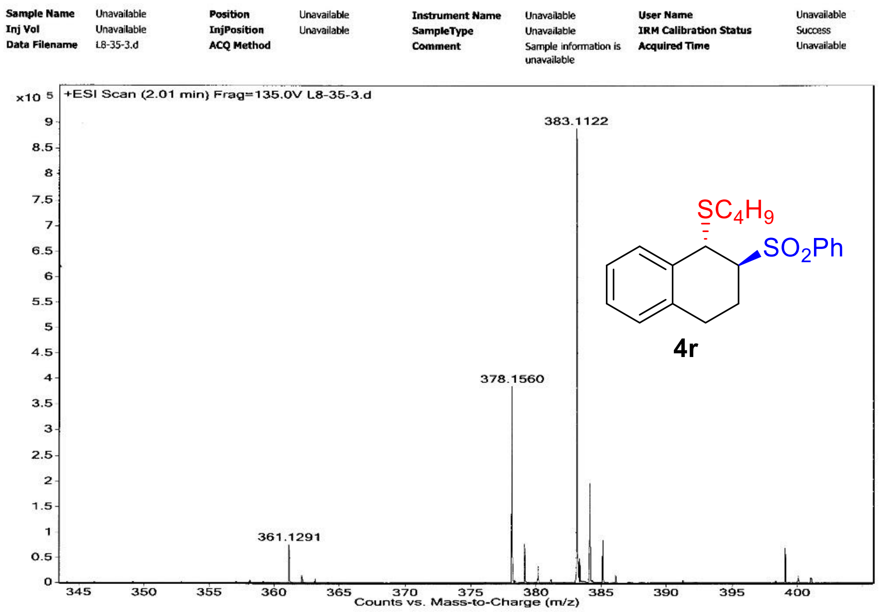

HRMS (ESI, m/z) calcd for $C_{20}H_{24}O_2S_2$ $[M+Na]^+$ 383.1110, found 383.1122.

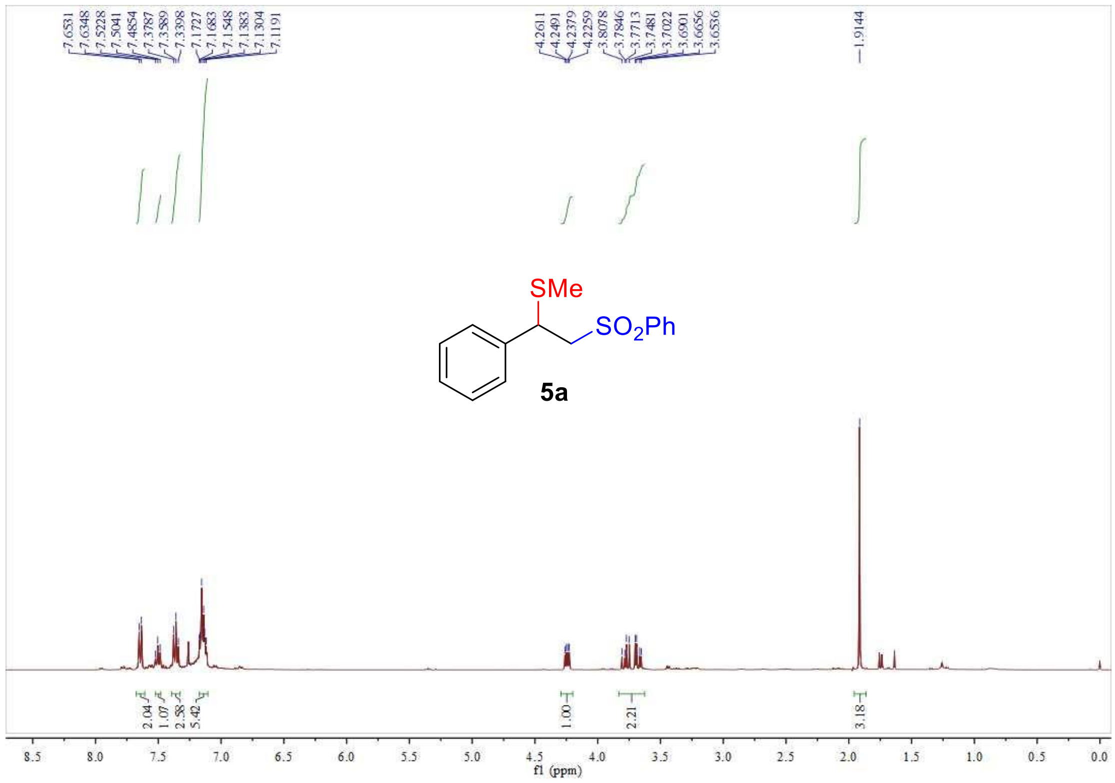

7.6531
7.6348
7.5228
7.5041
7.4854
7.3787
7.3589
7.3398
7.1727
7.1683
7.1548
7.1383
7.1304
7.1191
4.2611
4.2491
4.2379
4.2259
3.8078
3.7846
3.7713
3.7481
3.7022
3.6901
3.6656
3.6536
1.9144
SMe
SO2Ph
5a
2.04
1.07
2.58
5.42
1.00
2.21
3.18
f1 (ppm)

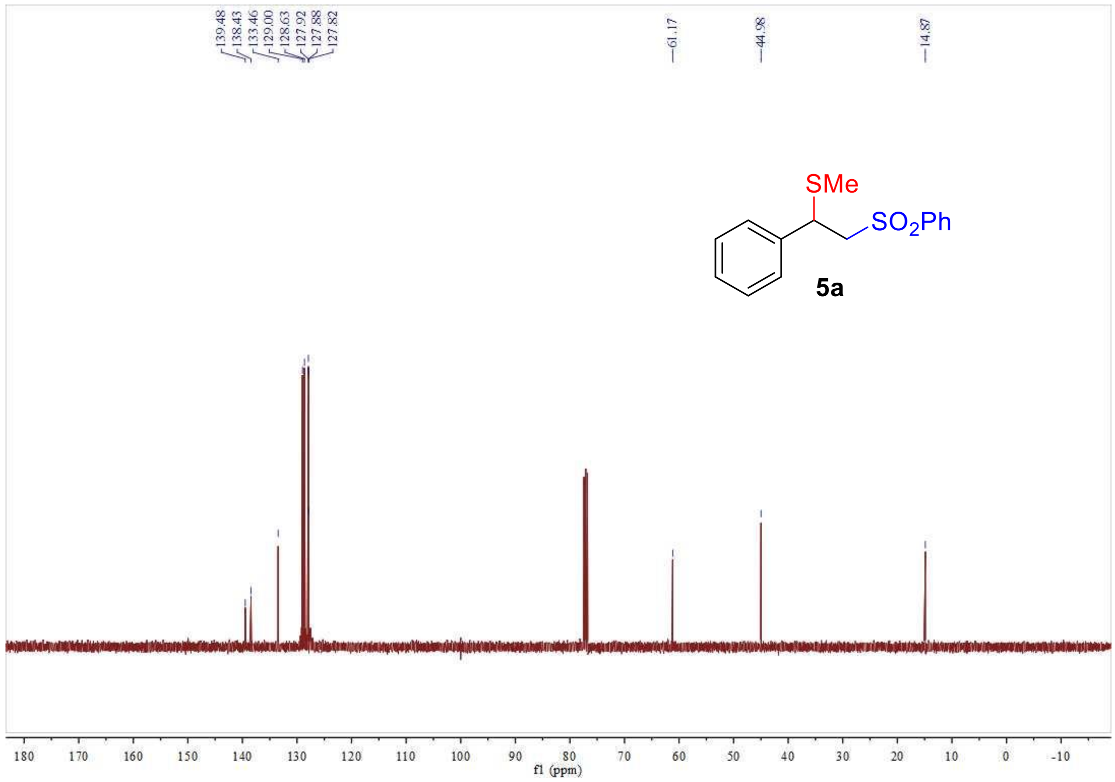

139.48
138.43
133.46
129.00
128.63
127.92
127.88
127.82
61.17
44.98
14.87
SMe
SO2Ph
5a
f1 (ppm)

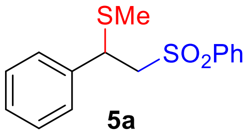

5a

Chemical Formula: $C_{15}H_{16}O_2S_2$
Exact Mass: 292.0592
Molecular Weight: 292.4110
m/z: 292.0592 (100.0%), 293.0625 (16.2%), 294.0550 (9.0%), 293.0586 (1.6%), 294.0659 (1.2%)
Elemental Analysis: C, 61.61; H, 5.52; O, 10.94; S, 21.93

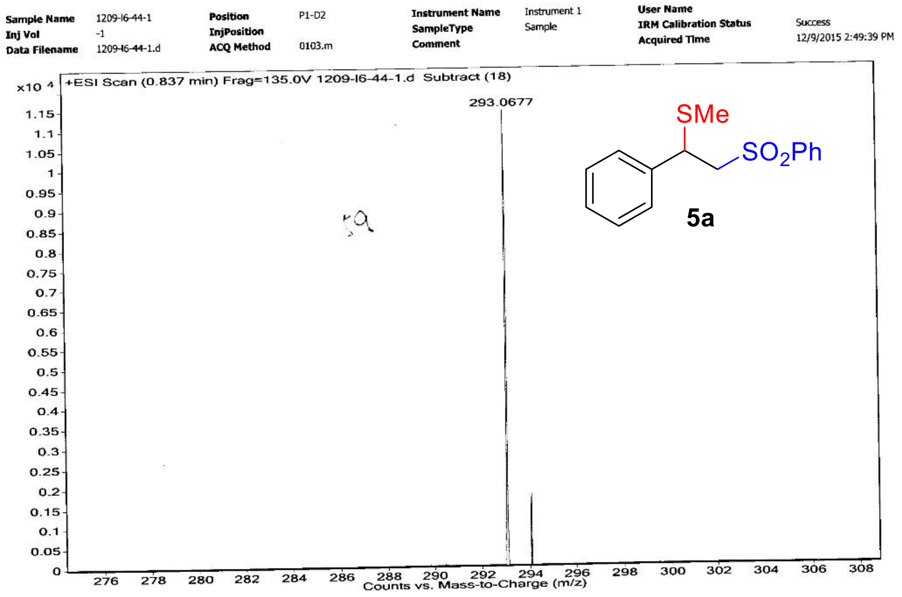

HRMS (ESI, m/z) calcd for $C_{15}H_{16}O_2S_2$ $[M+H]^+$ 293.0664, found 293.0677.

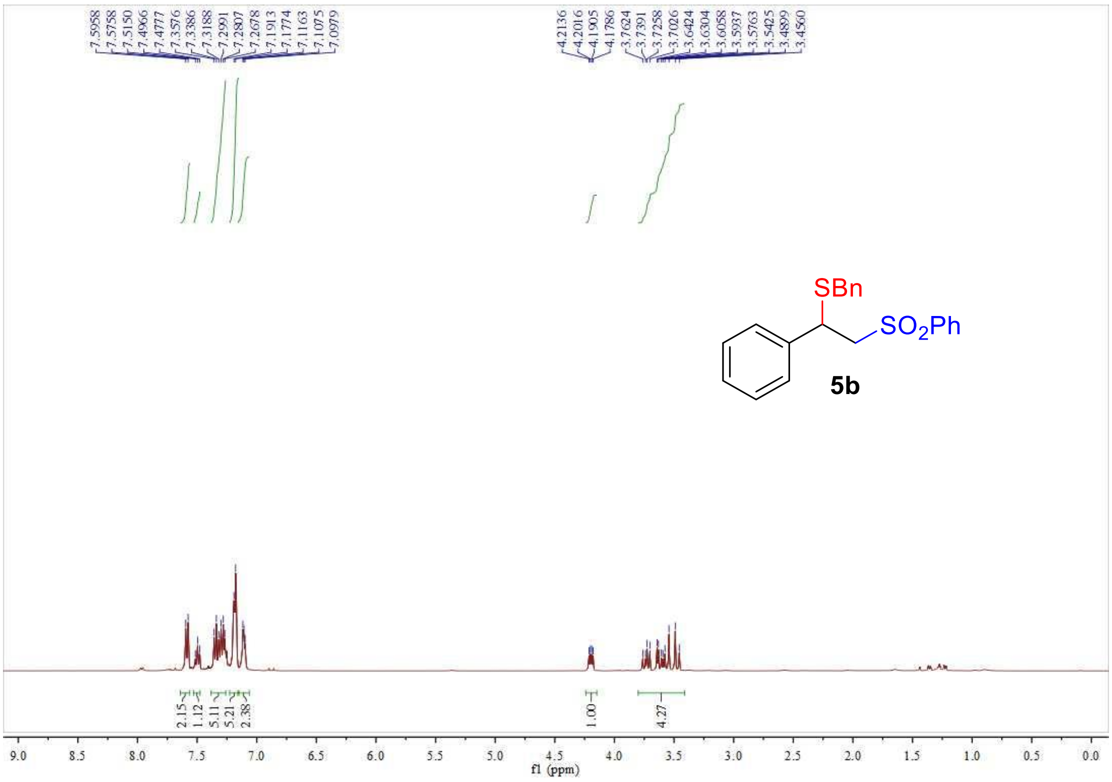
7.5958
7.5758
7.5150
7.4966
7.4777
7.3576
7.3386
7.3188
7.2991
7.2807
7.2678
7.1913
7.1774
7.1163
7.1075
7.0979
4.2136
4.2016
4.1905
4.1786
3.7624
3.7391
3.7258
3.7026
3.6424
3.6304
3.6058
3.5937
3.5763
3.5425
3.4899
3.4560
SBn
SO2Ph
5b
2.15
1.12
5.11
5.21
2.38
1.00
4.27
f1 (ppm)

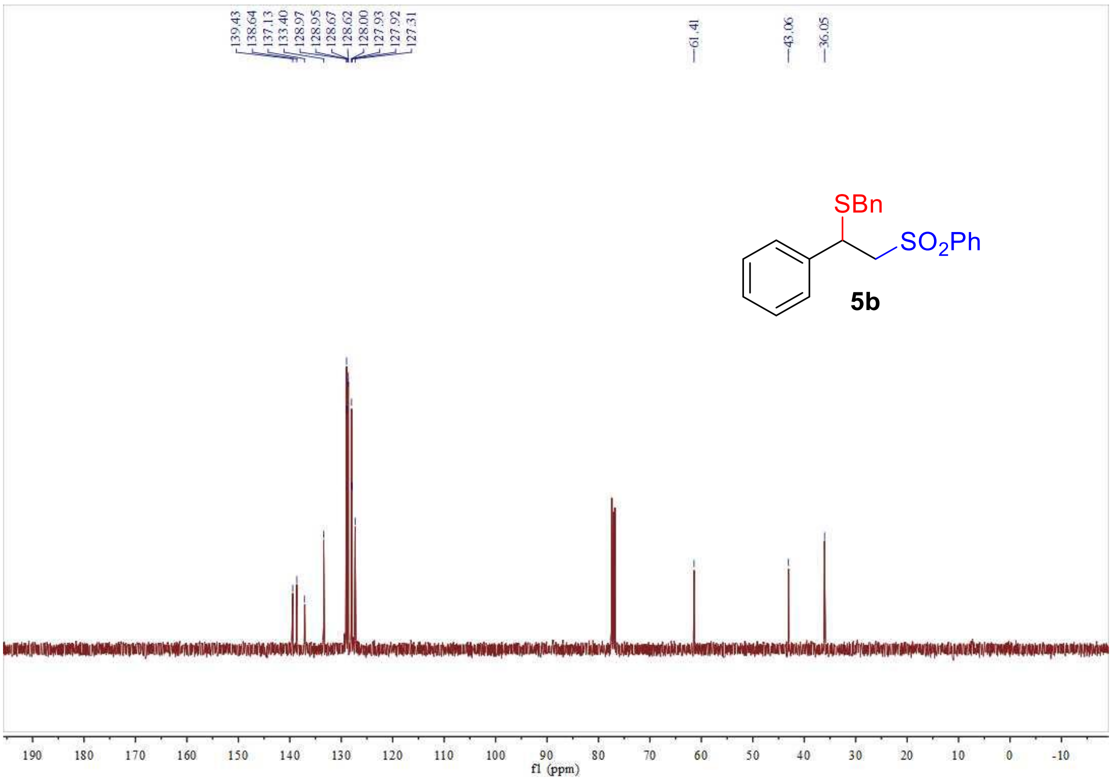
139.43
138.64
137.13
133.40
128.97
128.95
128.67
128.62
128.00
127.93
127.92
127.31
61.41
43.06
36.05
SBn
SO2Ph
5b
f1 (ppm)

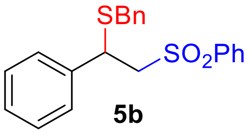

5b

Chemical Formula: $C_{21}H_{20}O_2S_2$
Exact Mass: 368.0905
Molecular Weight: 368.5090
m/z: 368.0905 (100.0%), 369.0938 (22.7%), 370.0863 (9.0%), 370.0972 (2.5%), 371.0896 (2.1%), 369.0899 (1.6%)
Elemental Analysis: C, 68.45; H, 5.47; O, 8.68; S, 17.40

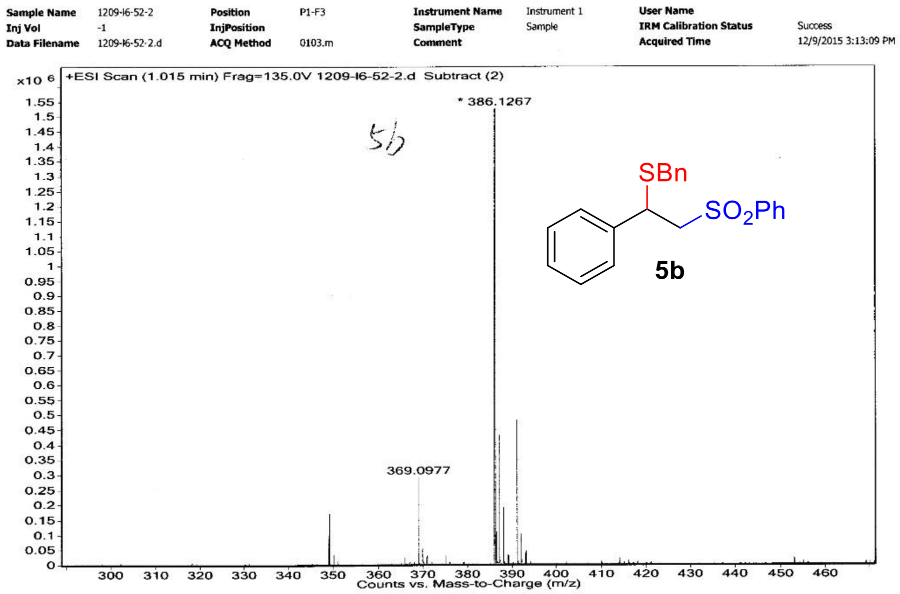

HRMS (ESI, m/z) calcd for $C_{21}H_{20}O_2S_2$ $[M+H]^+$ 369.0977, found 369.0977.

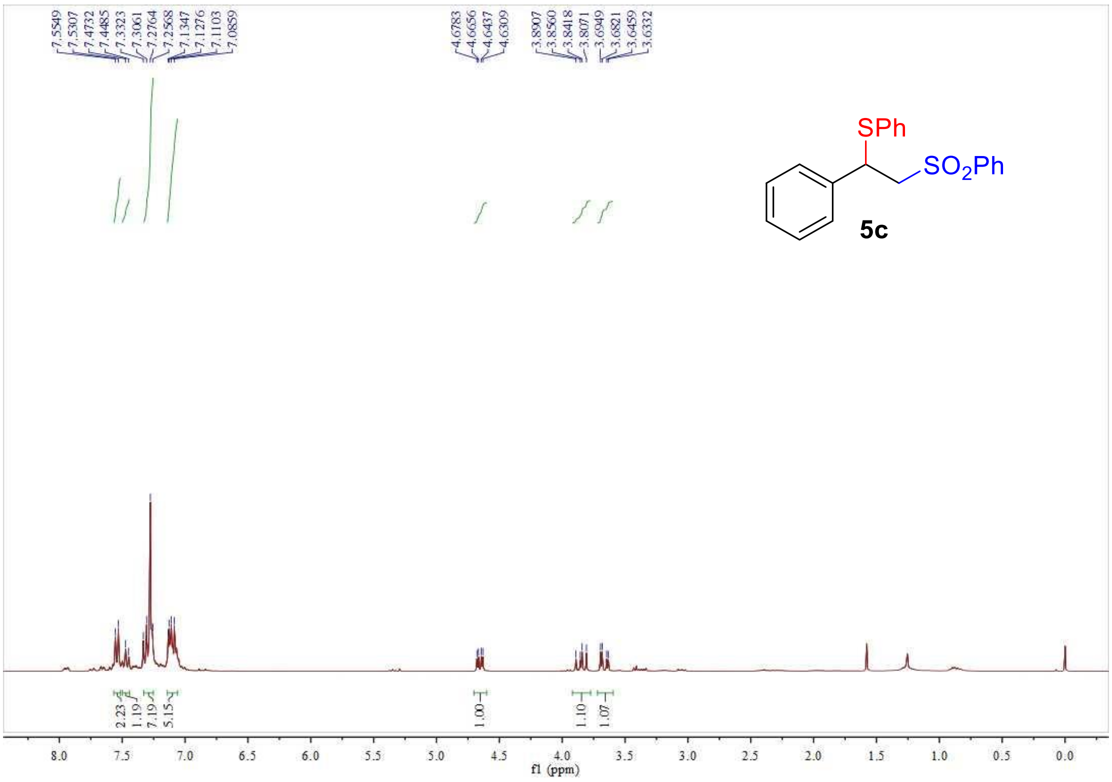
SPh
SO2Ph
5c
f1 (ppm)

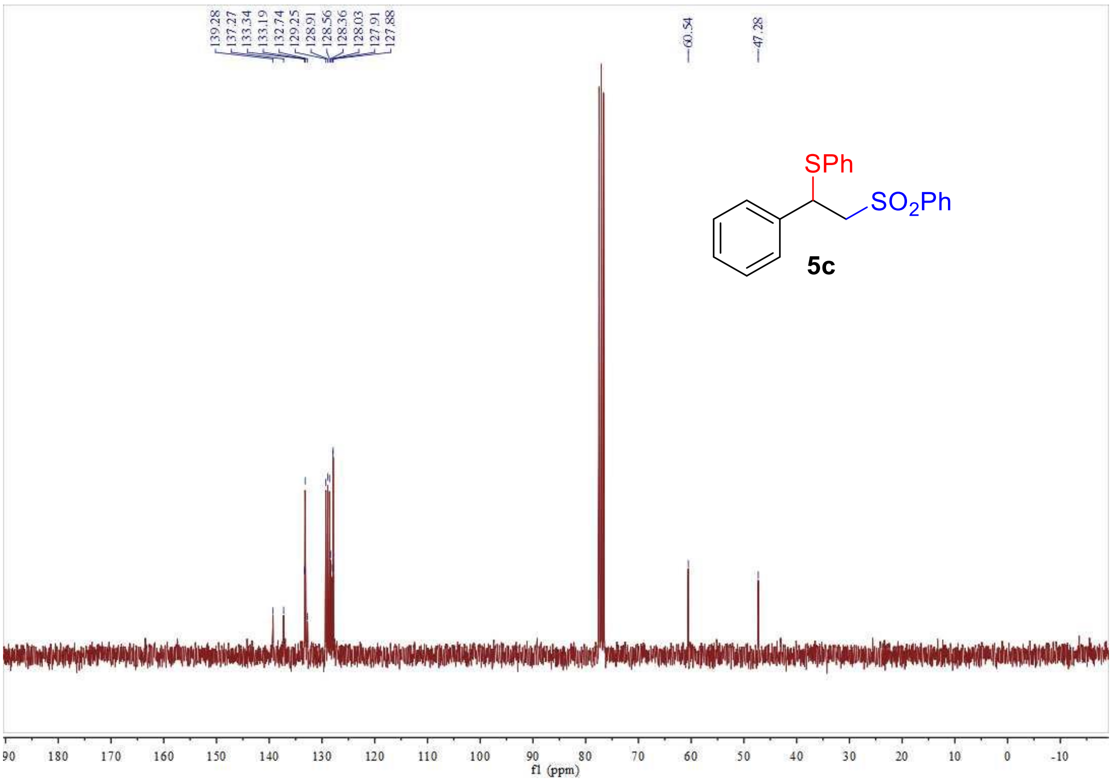
SPh
SO2Ph
5c
f1 (ppm)

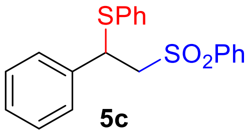

5c

Chemical Formula: $C_{20}H_{18}O_2S_2$
Exact Mass: 354.0748
Molecular Weight: 354.4820
m/z: 354.0748 (100.0%), 355.0782 (21.6%), 356.0706 (9.0%), 356.0815 (2.2%), 357.0740 (2.0%), 355.0742 (1.6%)
Elemental Analysis: C, 67.77; H, 5.12; O, 9.03; S, 18.09

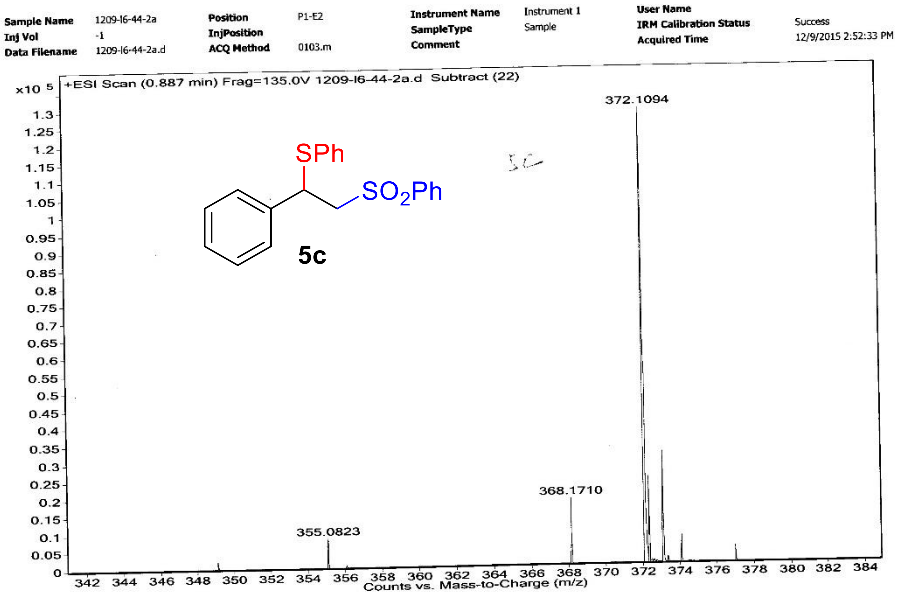

HRMS (ESI, m/z) calcd for $C_{20}H_{18}O_2S_2$ $[M+H]^+$ 355.0821, found 355.0823.

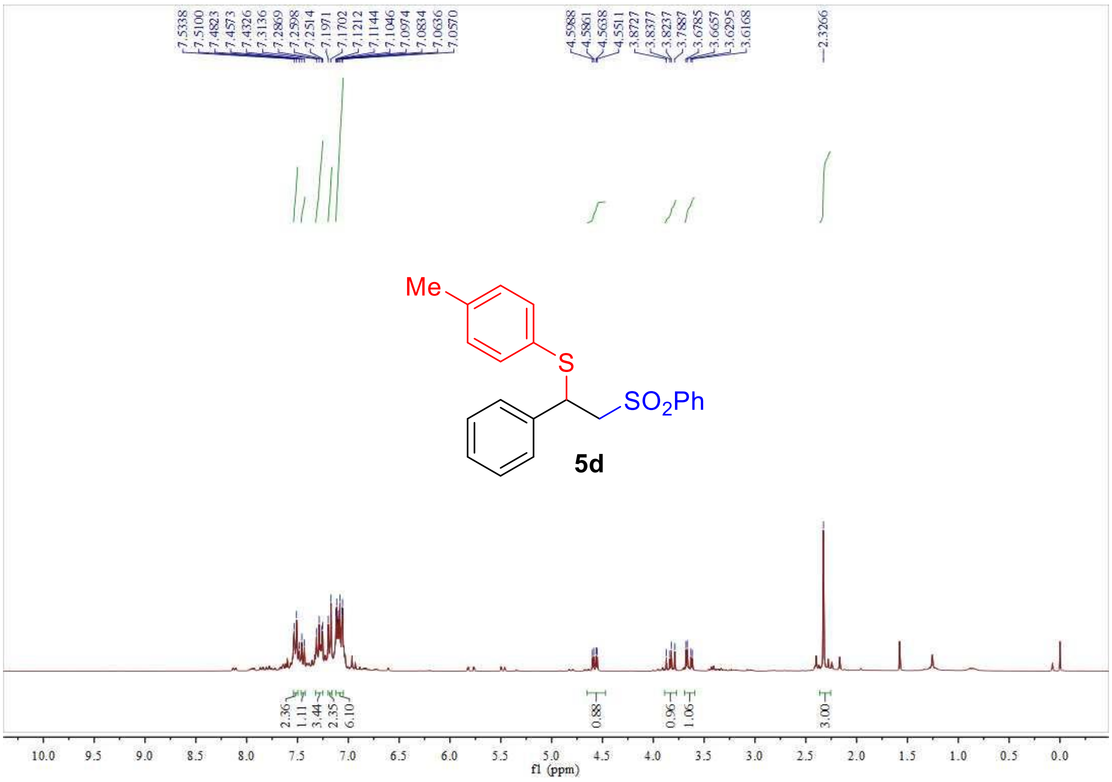
7.5338
7.5100
7.4823
7.4573
7.4326
7.3136
7.2869
7.2598
7.2514
7.1971
7.1702
7.1212
7.1144
7.1046
7.0974
7.0834
7.0636
7.0570
4.5988
4.5861
4.5638
4.5511
3.8727
3.8377
3.8237
3.7887
3.6785
3.6657
3.6295
3.6168
2.3266
Me
S
SO2Ph
5d
2.36
1.11
3.44
2.35
6.10
0.88
0.96
1.06
3.00
f1 (ppm)

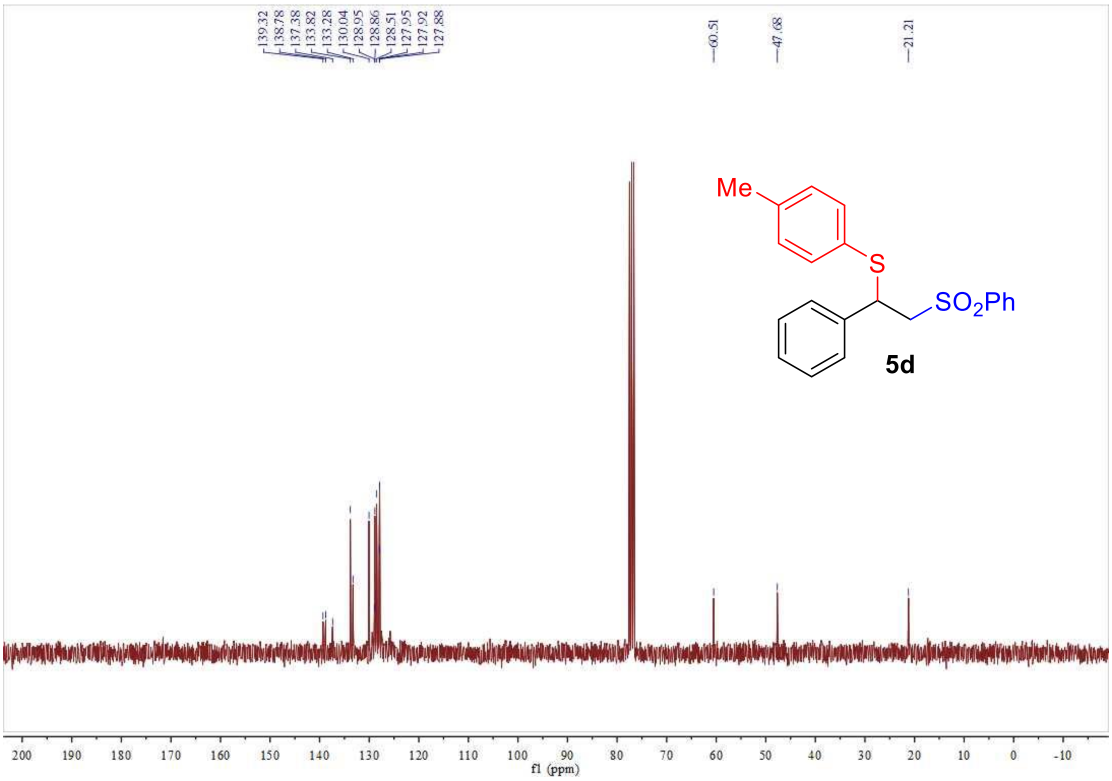
139.32
138.78
137.38
133.82
133.28
130.04
128.95
128.86
128.51
127.95
127.92
127.88
60.51
47.68
21.21
Me
S
SO2Ph
5d
f1 (ppm)

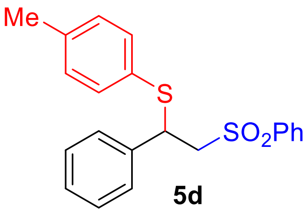

5d

Chemical Formula: $C_{21}H_{20}O_2S_2$
Exact Mass: 368.0905
Molecular Weight: 368.5090
m/z: 368.0905 (100.0%), 369.0938 (22.7%), 370.0863 (9.0%), 370.0972 (2.5%), 371.0896 (2.1%), 369.0899 (1.6%)
Elemental Analysis: C, 68.45; H, 5.47; O, 8.68; S, 17.40

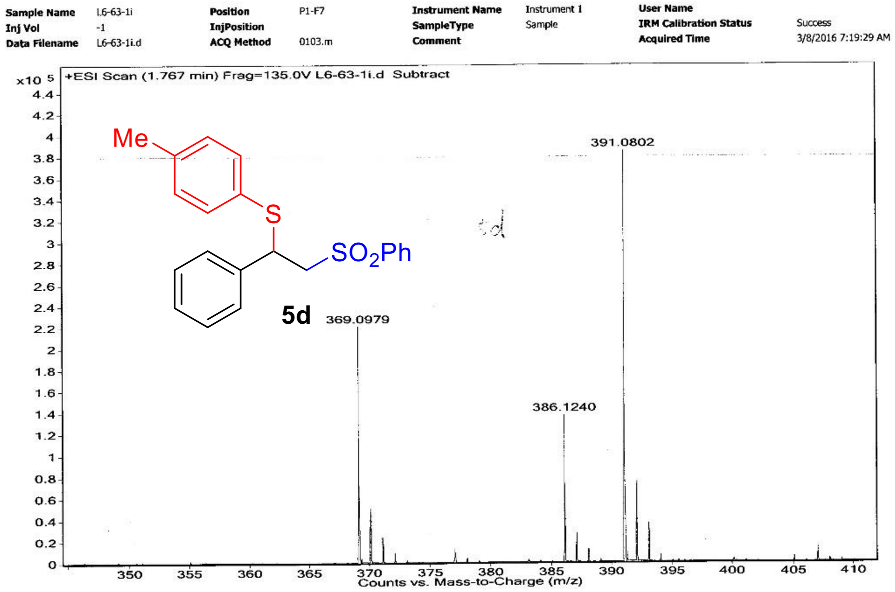

HRMS (ESI, m/z) calcd for $C_{21}H_{20}O_2S_2$ $[M+H]^+$ 369.0977, found 369.0979.

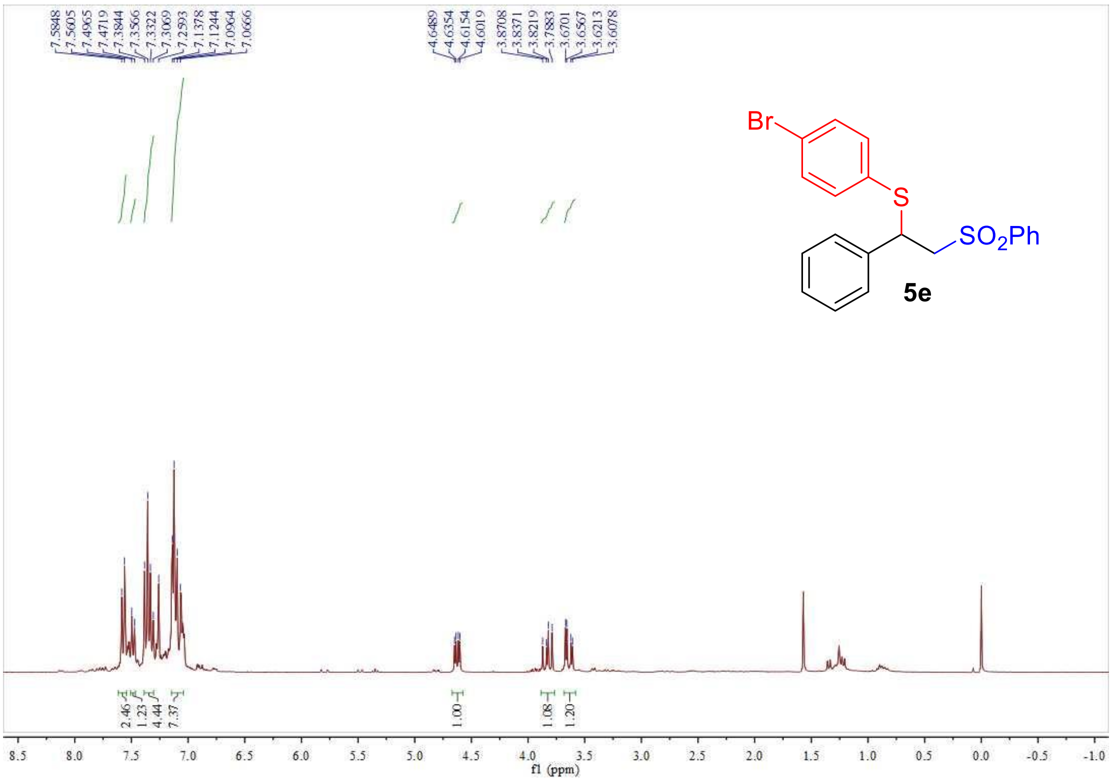
7.5848
7.5605
7.4965
7.4719
7.3844
7.3566
7.3322
7.3069
7.2593
7.1378
7.1244
7.0964
7.0666
4.6489
4.6354
4.6154
4.6019
3.8708
3.8371
3.8219
3.7883
3.6701
3.6567
3.6213
3.6078
Br
S
SO2Ph
5e
2.46
1.23
4.44
7.37
1.00
1.08
1.20
f1 (ppm)

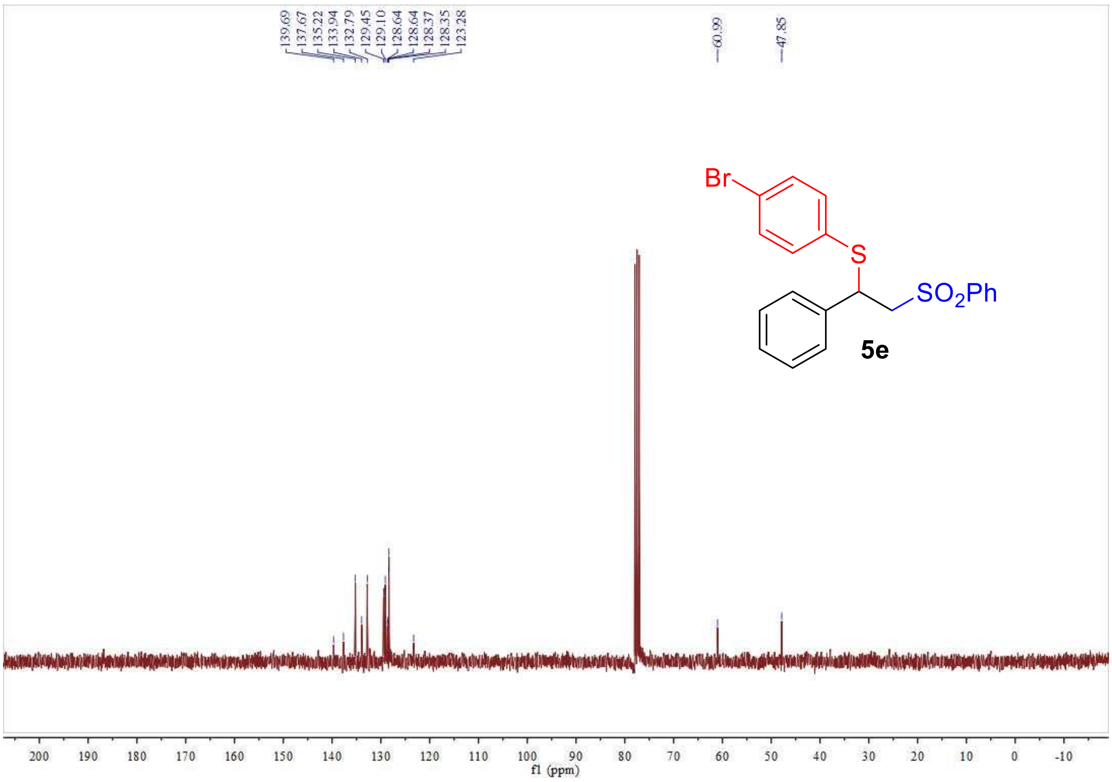
139.69
137.67
135.22
133.94
132.79
129.45
129.10
128.64
128.64
128.37
128.35
123.28
60.99
47.85
Br
S
SO2Ph
5e
f1 (ppm)

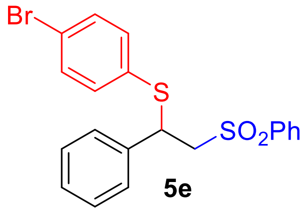

5e

Chemical Formula: $C_{20}H_{17}BrO_2S_2$

Exact Mass: 431.9853

Molecular Weight: 433.3780

m/z: 431.9853 (100.0%), 433.9833 (97.3%), 432.9887 (21.6%), 434.9866 (21.0%), 433.9811 (9.0%), 435.9791 (8.8%), 435.9900 (2.2%), 436.9824 (1.9%), 432.9847 (1.6%), 434.9827 (1.6%), 434.9845 (1.5%), 433.9920 (1.1%), 433.9920 (1.0%)

Elemental Analysis: C, 55.43; H, 3.95; Br, 18.44; O, 7.38; S, 14.80

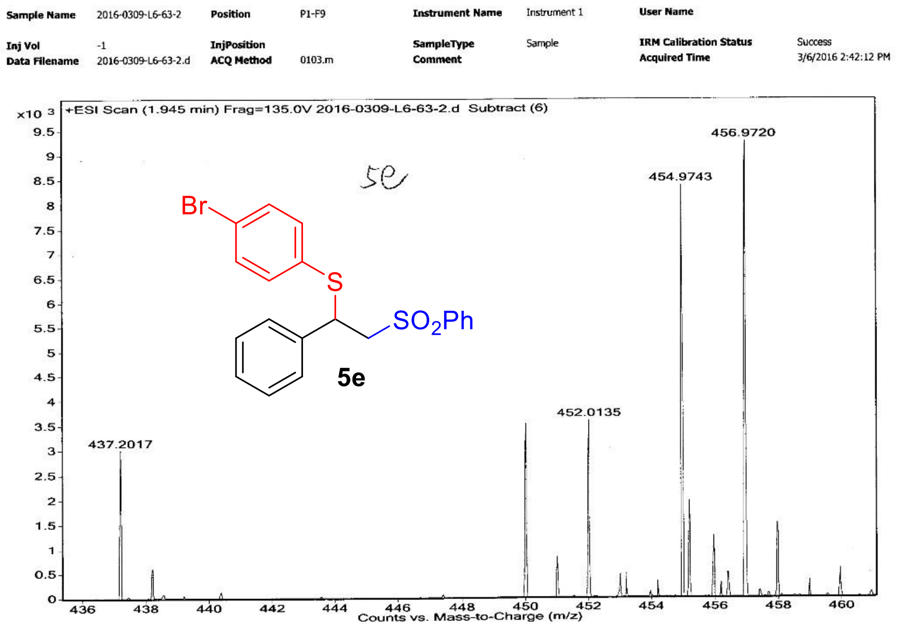

HRMS (ESI, m/z) calcd for $C_{20}H_{17}BrO_2S_2$ $[M+Na]^+$ 454.9746, found 454.9743.

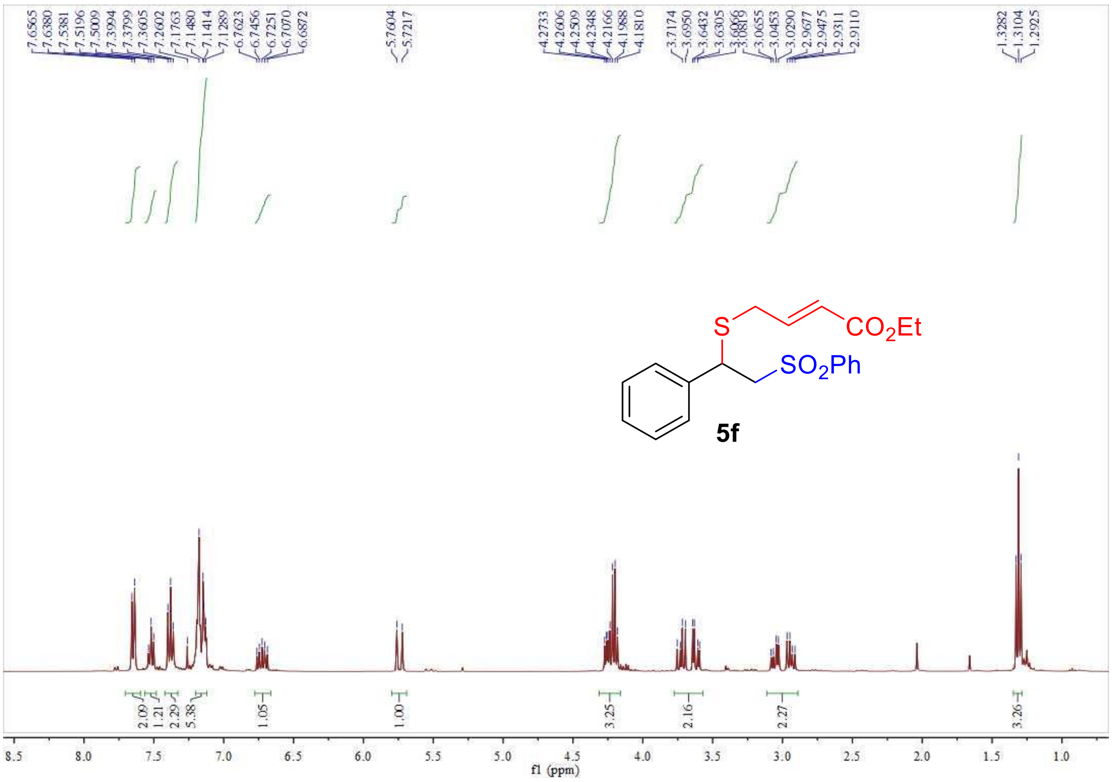

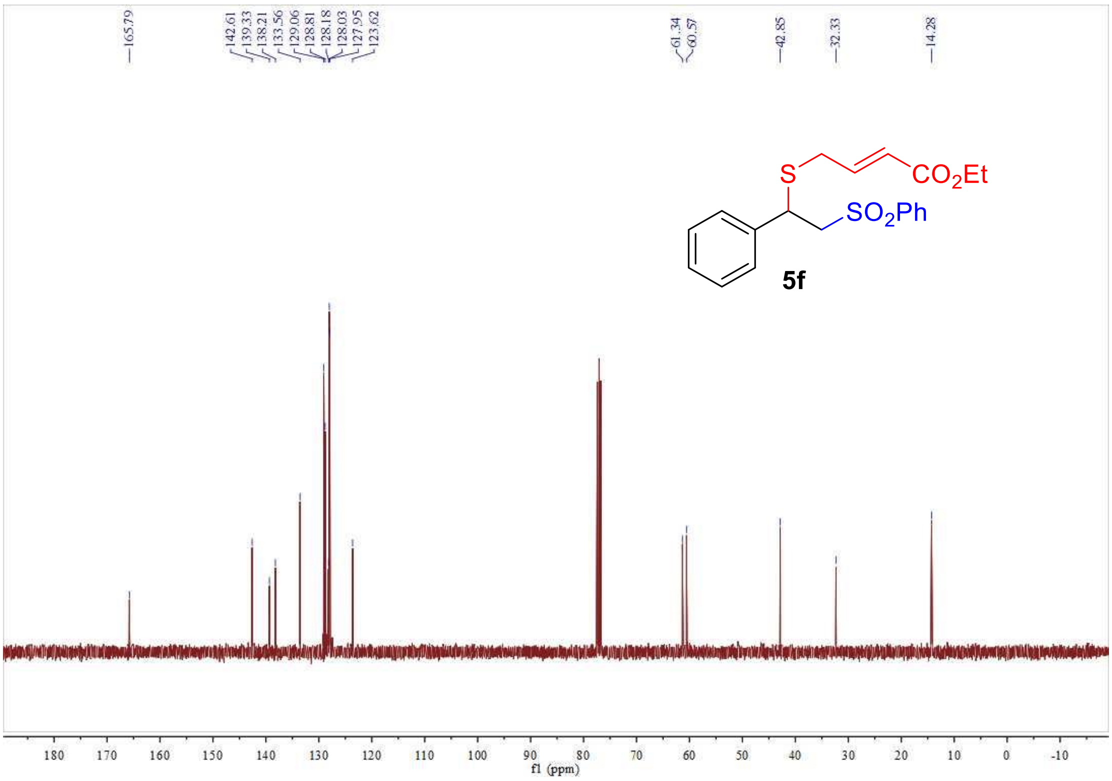

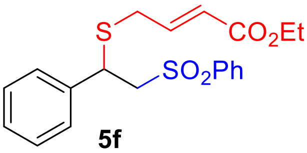

5f

Chemical Formula: $C_{20}H_{22}O_4S_2$
Exact Mass: 390.0960
Molecular Weight: 390.5120
m/z: 390.0960 (100.0%), 391.0993 (21.6%), 392.0917 (9.0%), 392.1027 (2.2%), 393.0951 (2.0%), 391.0953 (1.6%)
Elemental Analysis: C, 61.51; H, 5.68; O, 16.39; S, 16.42

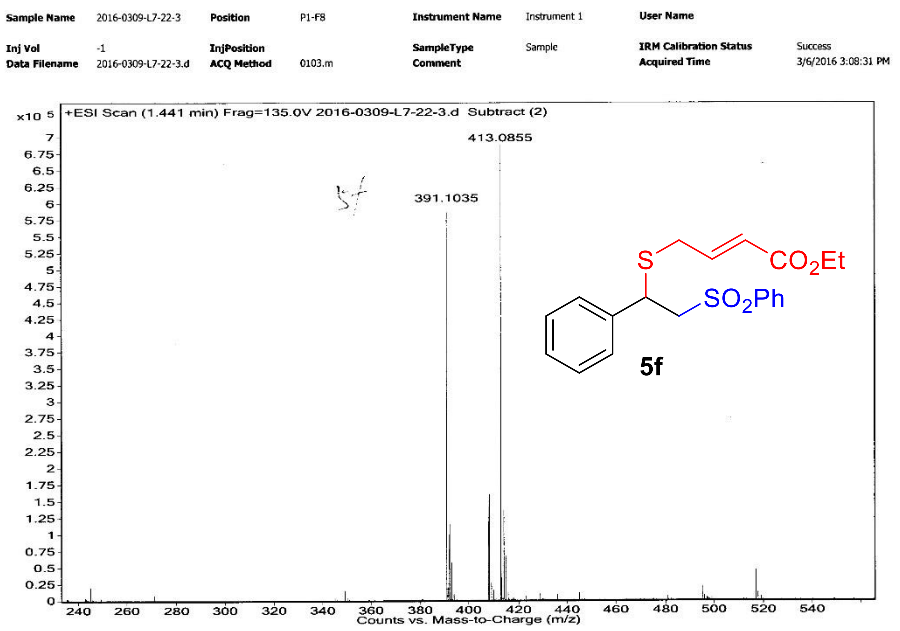

HRMS (ESI, m/z) calcd for $C_{20}H_{22}O_4S_2$ $[M+H]^+$ 391.1032, found 391.1035.

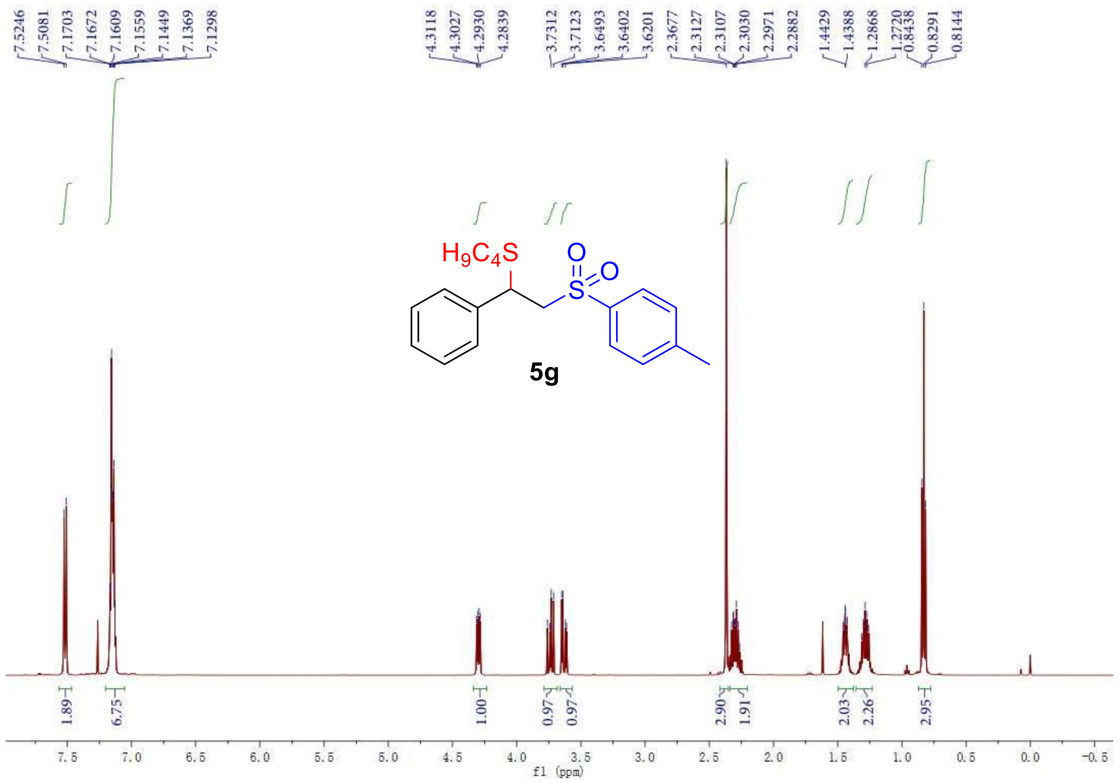
7.5246
7.5081
7.1703
7.1672
7.1609
7.1559
7.1449
7.1369
7.1298
4.3118
4.3027
4.2930
4.2839
3.7312
3.7123
3.6493
3.6402
3.6201
2.3677
2.3127
2.3107
2.3030
2.2971
2.2882
1.4429
1.4388
1.2868
1.2720
0.8438
0.8291
0.8144
H9C4S
O
S
O
5g
1.89
6.75
1.00
0.97
0.97
2.90
1.91
2.03
2.26
2.95
7.5 7.0 6.5 6.0 5.5 5.0 4.5 4.0 3.5 3.0 2.5 2.0 1.5 1.0 0.5 0.0 -0.5
f1 (ppm)

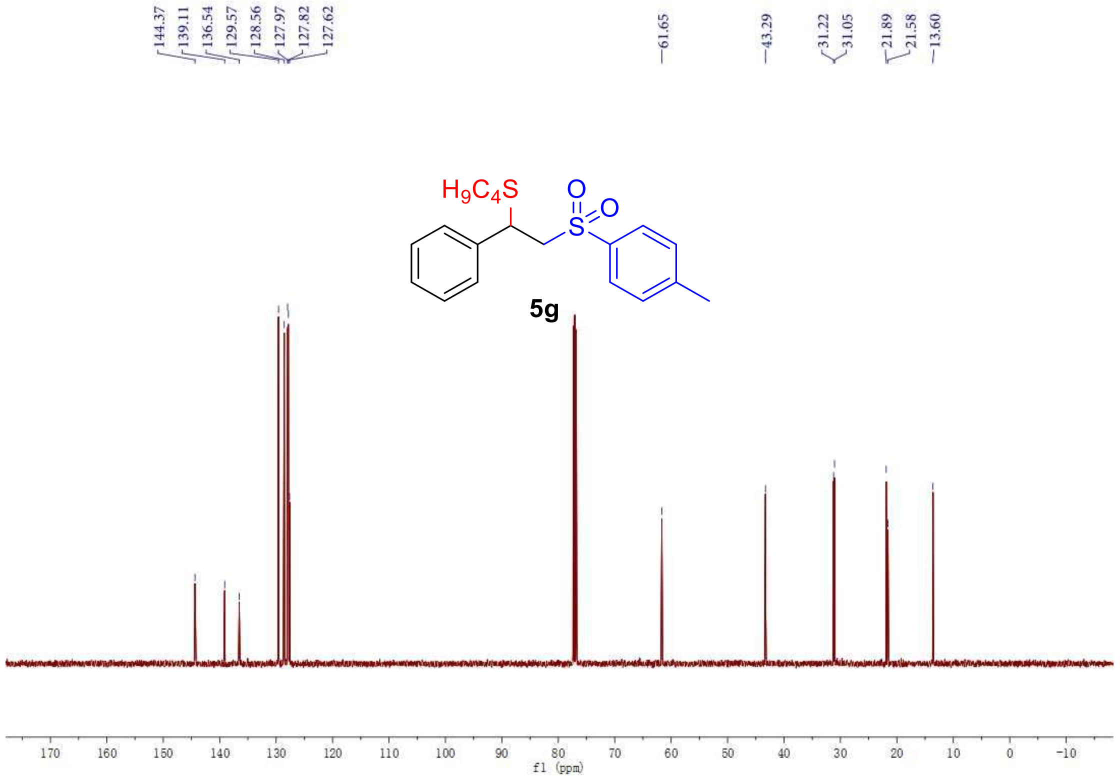
144.37
139.11
136.54
129.57
128.56
127.97
127.82
127.62
61.65
43.29
31.22
31.05
21.89
21.58
13.60
H9C4S
O
S
O
5g
170 160 150 140 130 120 110 100 90 80 70 60 50 40 30 20 10 0 -10
f1 (ppm)

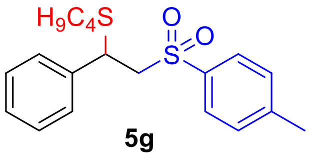

5g

Chemical Formula: $C_{19}H_{24}O_2S_2$
Exact Mass: 348.1218
Molecular Weight: 348.5190
m/z: 348.1218 (100.0%), 349.1251 (20.5%), 350.1176 (9.0%), 350.1285 (2.0%), 351.1209 (1.9%), 349.1212 (1.6%)
Elemental Analysis: C, 65.48; H, 6.94; O, 9.18; S, 18.40

**Sample Name**	20161215-L9-55-2	**Position**	P1-C8	**Instrument Name**	Instrument 1	**User Name**	
**Inj Vol**	-1	**InjPosition**		**SampleType**	Sample	**IRM Calibration Status**	Success
**Data Filename**	20161215-L9-55-2.d	**ACQ Method**	0103.m	**Comment**		**Acquired Time**	12/15/2016 11:03:41 AM

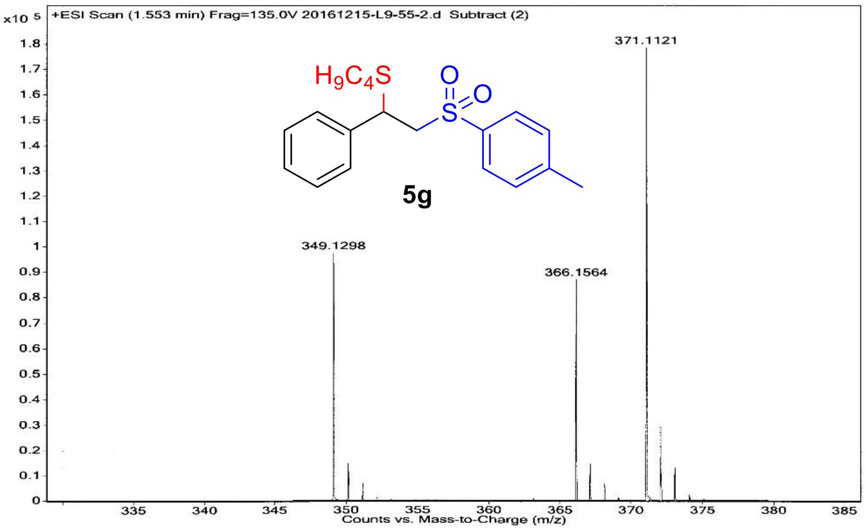

HRMS (ESI, m/z) calcd for $C_{19}H_{24}O_2S_2$ $[M+Na]^+$ 371.1110, found 371.1121.

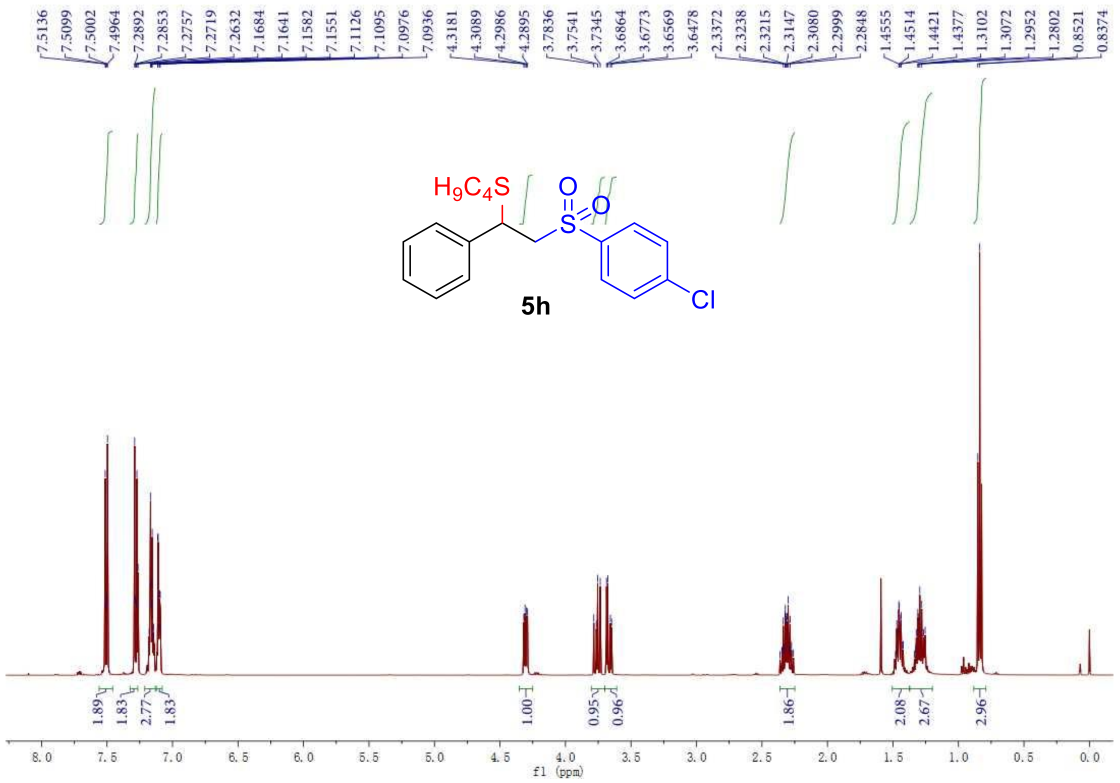
7.5136
7.5099
7.5002
7.4964
7.2892
7.2853
7.2757
7.2719
7.2632
7.1684
7.1641
7.1582
7.1551
7.1126
7.1095
7.0976
7.0936
4.3181
4.3089
4.2986
4.2895
3.7836
3.7541
3.7345
3.6864
3.6773
3.6569
3.6478
2.3372
2.3238
2.3215
2.3147
2.3080
2.2999
2.2848
1.4555
1.4514
1.4421
1.4377
1.3102
1.3072
1.2952
1.2802
0.8521
0.8374
H_9C_4S
5h
Cl
1.89
1.83
2.77
1.83
1.00
0.95
0.96
1.86
2.08
2.67
2.96
f1 (ppm)

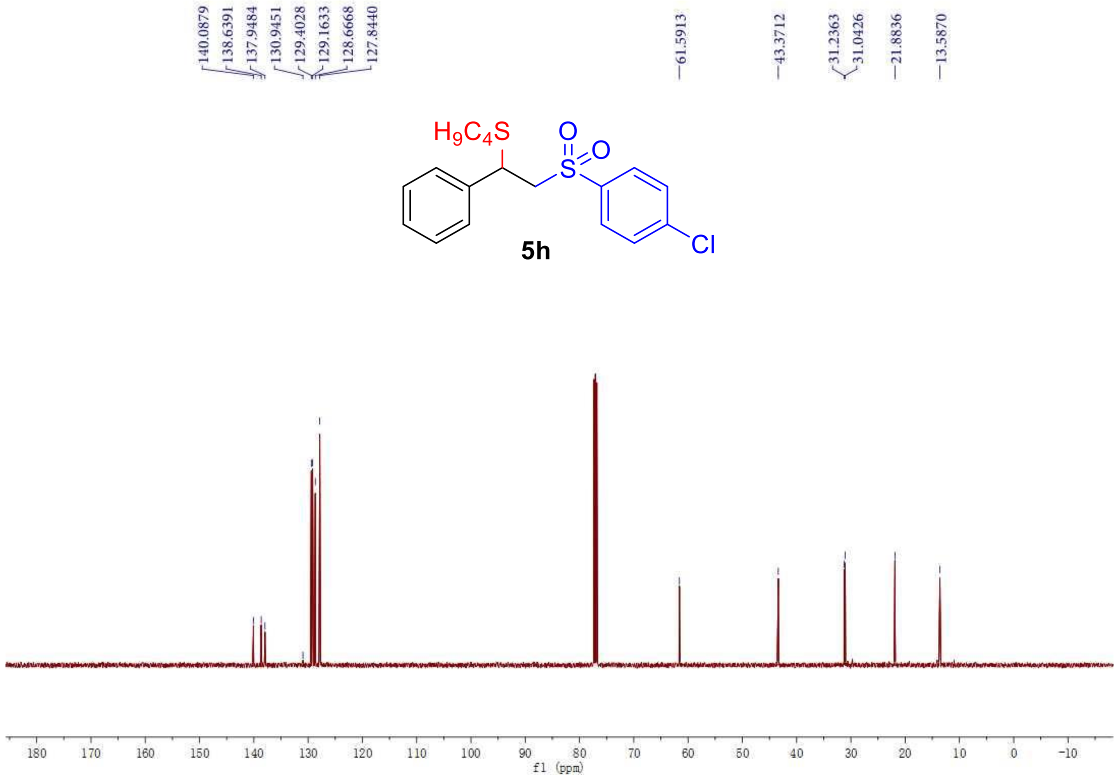
140.0879
138.6391
137.9484
130.9451
129.4028
129.1633
128.6668
127.8440
61.5913
43.3712
31.2363
31.0426
21.8836
13.5870
H_9C_4S
5h
Cl
f1 (ppm)

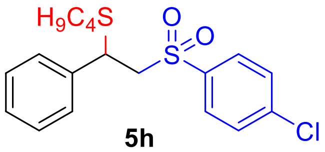

5h

Chemical Formula: $C_{18}H_{21}ClO_2S_2$
Exact Mass: 368.0671
Molecular Weight: 368.9340
m/z: 368.0671 (100.0%), 370.0642 (32.0%), 369.0705 (19.5%), 370.0629 (9.0%), 371.0676 (6.2%), 372.0600 (2.9%), 370.0739 (1.8%), 371.0663 (1.8%), 369.0665 (1.6%)
Elemental Analysis: C, 58.60; H, 5.74; Cl, 9.61; O, 8.67; S, 17.38

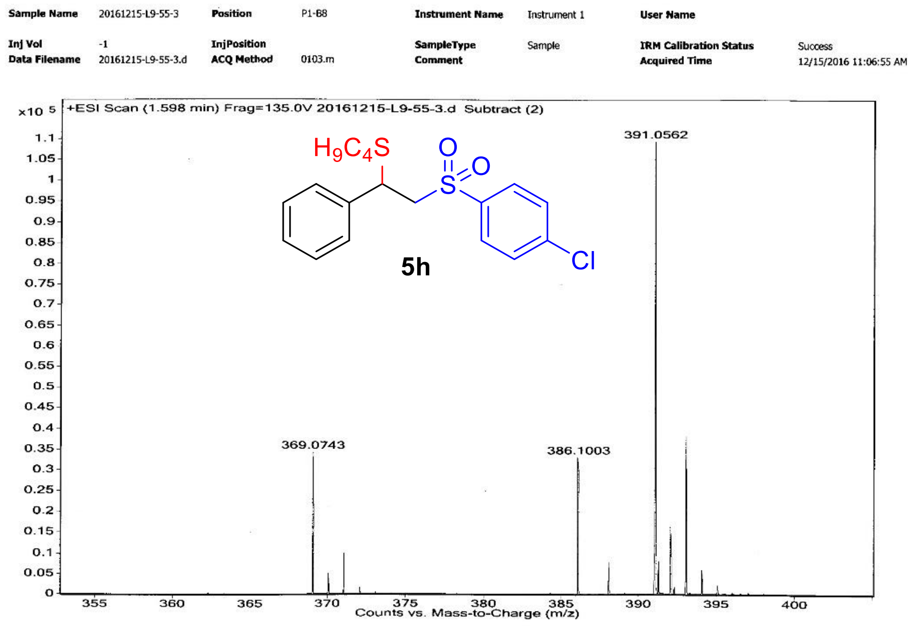

HRMS (ESI, m/z) calcd for $C_{18}H_{21}ClO_2S_2$ $[M+Na]^+$ 391.0564, found 391.0562.

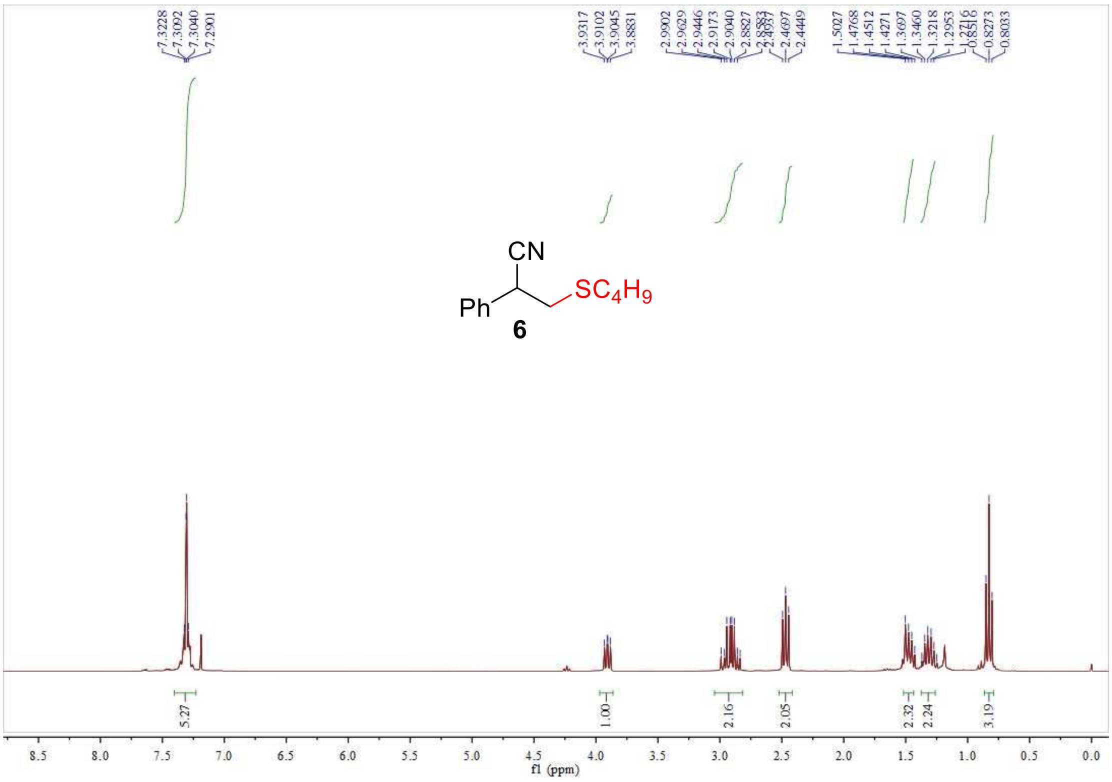
7.3228
7.3092
7.3040
7.2901
3.9317
3.9102
3.9045
3.8831
2.9902
2.9629
2.9446
2.9173
2.9040
2.8827
2.4697
2.4449
1.5027
1.4768
1.4512
1.4271
1.3697
1.3460
1.3218
1.2953
0.8273
0.8033
CN
Ph
SC_4H_9
6
5.27
1.00
2.16
2.05
2.32
2.24
3.19
8.5
8.0
7.5
7.0
6.5
6.0
5.5
5.0
4.5
4.0
3.5
3.0
2.5
2.0
1.5
1.0
0.5
0.0
f1 (ppm)

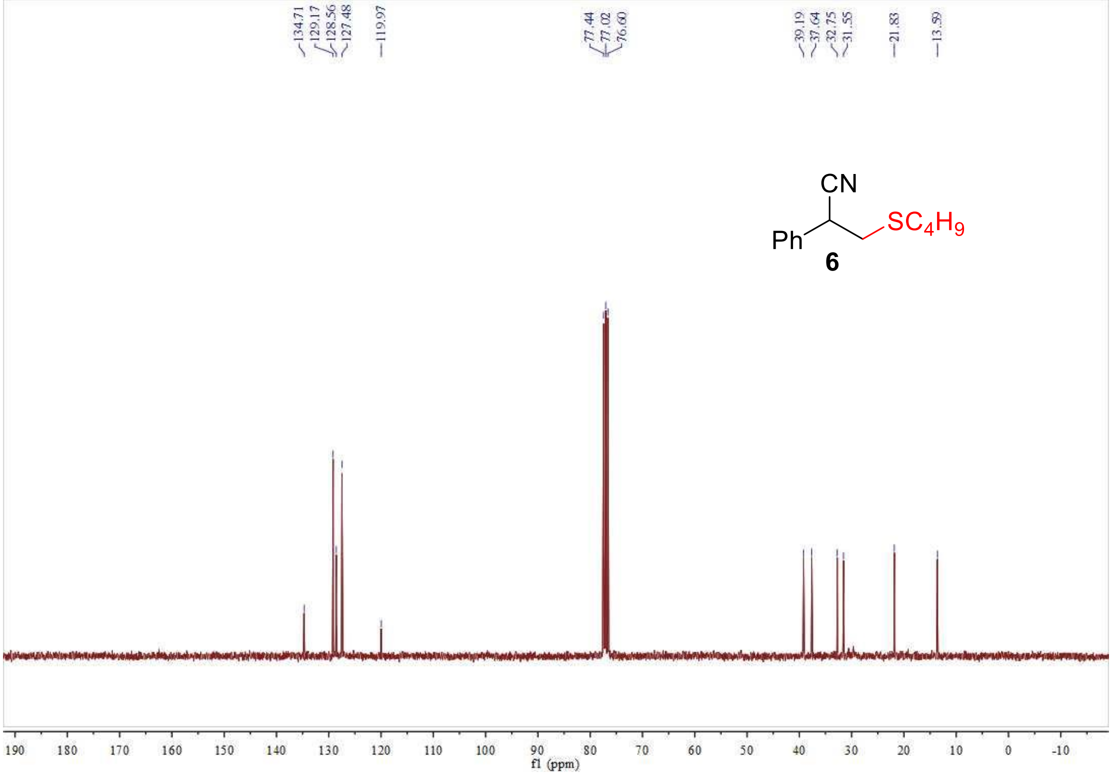
134.71
129.17
128.56
127.48
119.97
77.44
77.02
76.60
39.19
37.64
32.75
31.55
21.83
13.59
CN
Ph
SC_4H_9
6
190
180
170
160
150
140
130
120
110
100
90
80
70
60
50
40
30
20
10
0
-10
f1 (ppm)

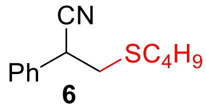

6

Chemical Formula: $C_{13}H_{17}NS$
Exact Mass: 219.1082
Molecular Weight: 219.3460
m/z: 219.1082 (100.0%), 220.1115 (14.1%), 221.1040 (4.5%)
Elemental Analysis: C, 71.19; H, 7.81; N, 6.39; S, 14.62

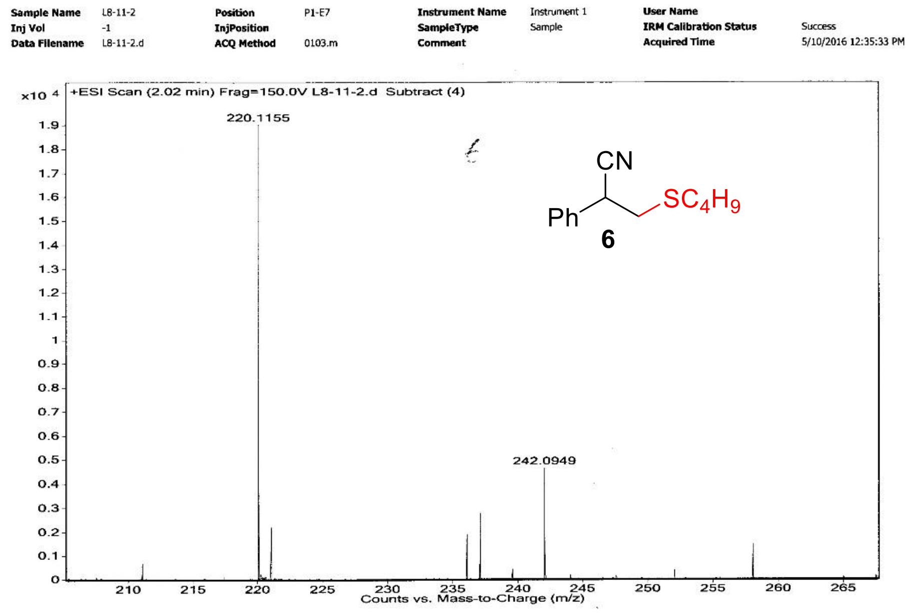

HRMS (ESI, m/z) calcd for $C_{13}H_{17}NS$ $[M+H]^+$ 220.1154, found 220.1155.

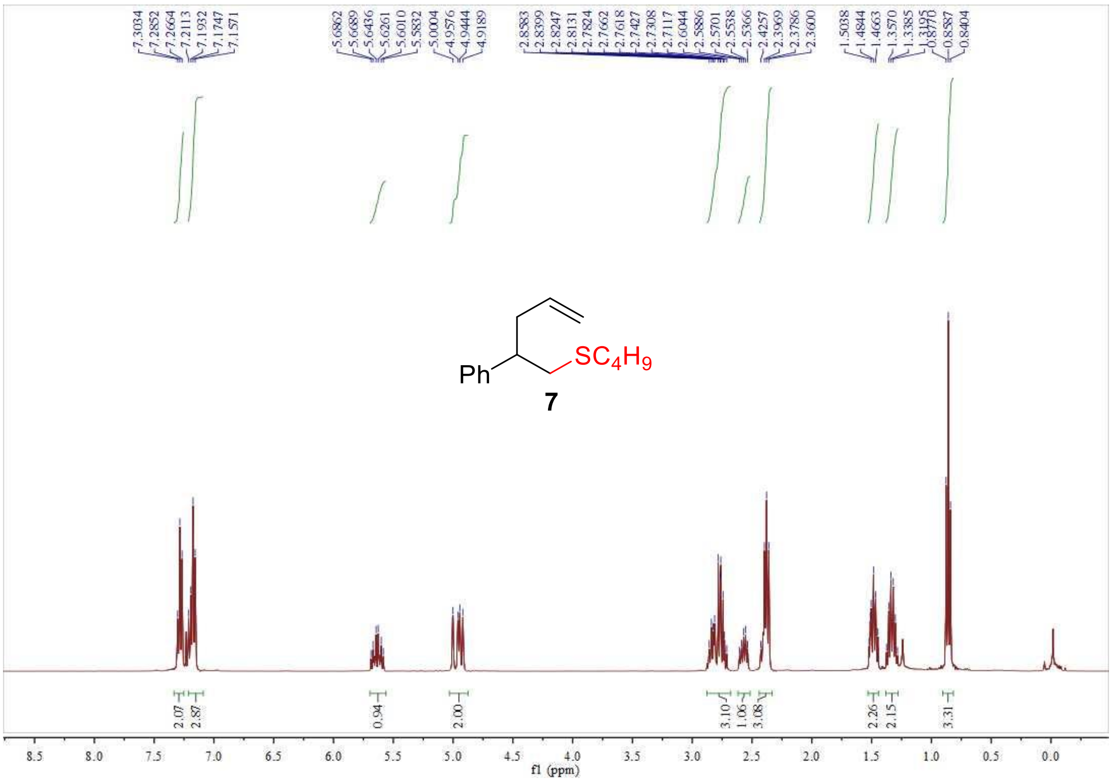
7.3034
7.2852
7.2664
7.2113
7.1932
7.1747
7.1571
5.6862
5.6689
5.6436
5.6261
5.6010
5.5832
5.0004
4.9576
4.9444
4.9189
2.8583
2.8399
2.8247
2.8131
2.7824
2.7662
2.7618
2.7427
2.7308
2.7117
2.6044
2.5886
2.5701
2.5538
2.5366
2.4257
2.3969
2.3786
2.3600
1.5038
1.4844
1.4663
1.3570
1.3385
1.3195
0.8770
0.8587
0.8404
Ph
SC_4H_9
7
2.07
2.87
0.94
2.00
3.10
1.06
3.08
2.26
2.15
3.31
f1 (ppm)

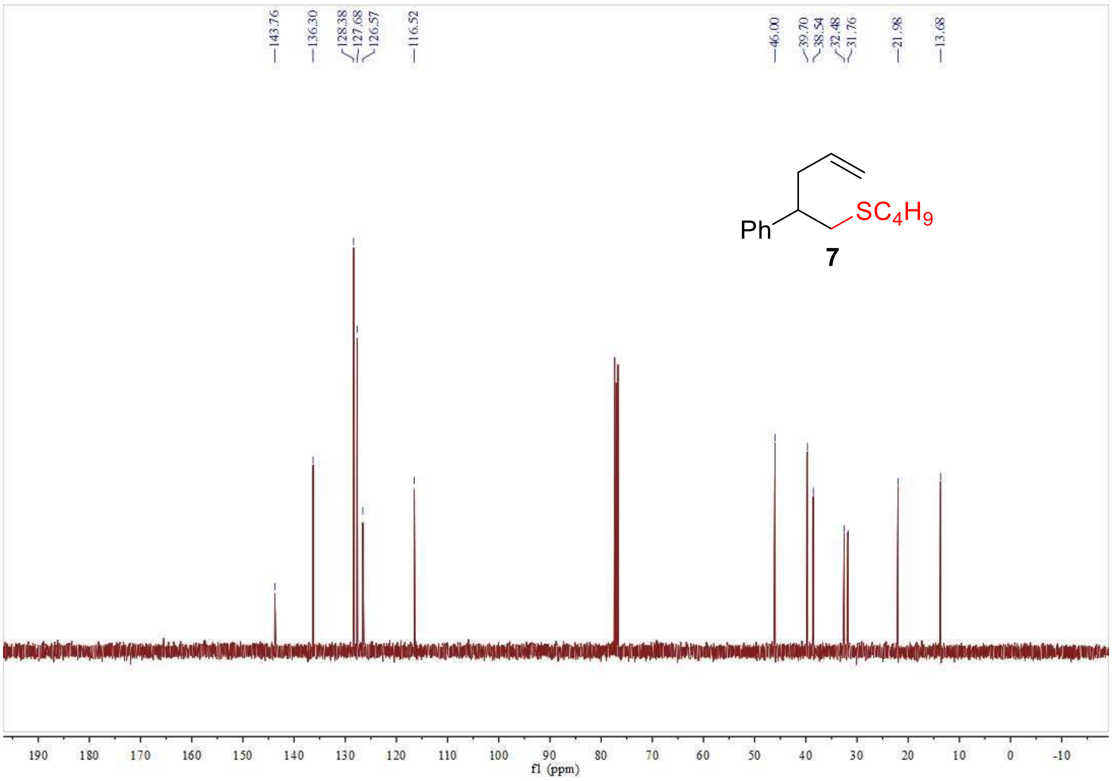
143.76
136.30
128.38
127.68
126.57
116.52
46.00
39.70
38.54
32.48
31.76
21.98
13.68
Ph
SC_4H_9
7
f1 (ppm)

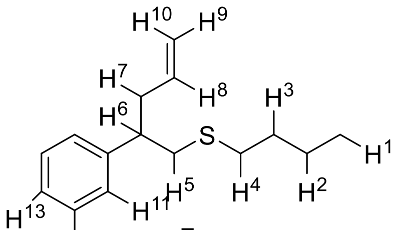
H10
H9
H7
H8
H3
H6
S
H1
H5
H4
H2
H13
H11
H12

7

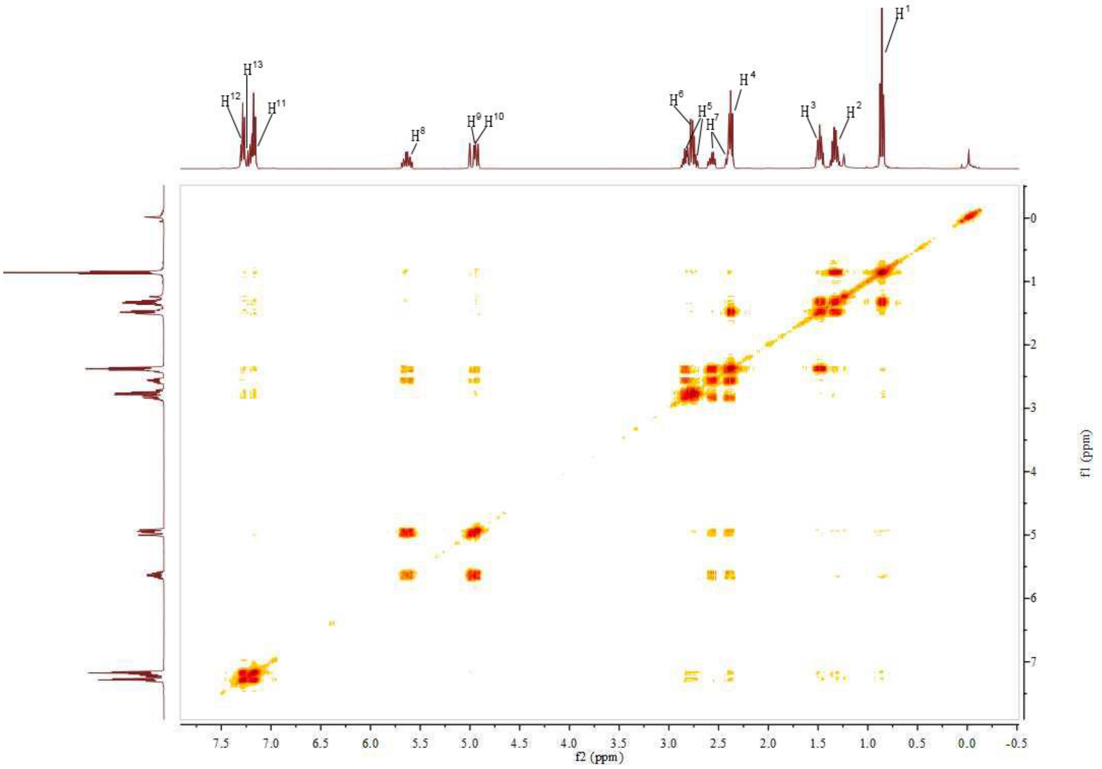
H1
H13
H4
H12
H11
H6
H5
H3
H2
H9
H10
H7
H8
f1 (ppm)
f2 (ppm)

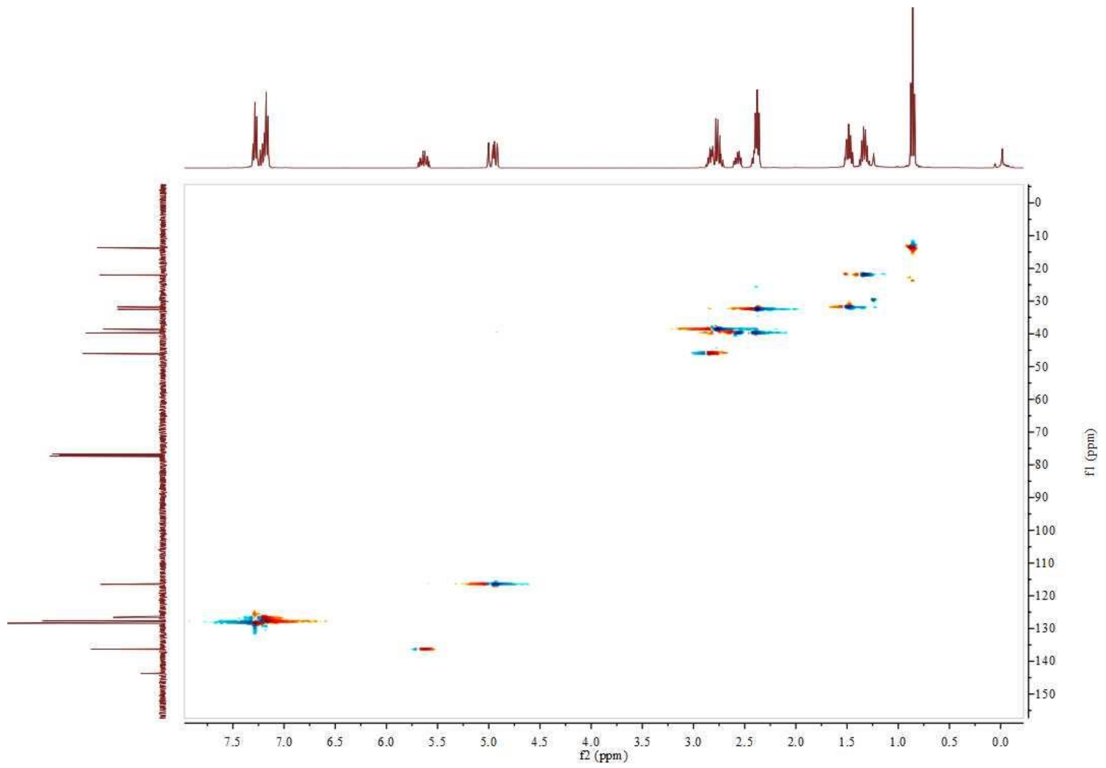
f2 (ppm)
f1 (ppm)

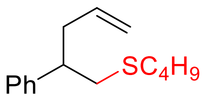

7

Chemical Formula: $C_{15}H_{22}S$
Exact Mass: 234.1442
Molecular Weight: 234.4010
m/z: 234.1442 (100.0%), 235.1476 (16.2%), 236.1400 (4.5%), 236.1509 (1.2%)
Elemental Analysis: C, 76.86; H, 9.46; S, 13.68

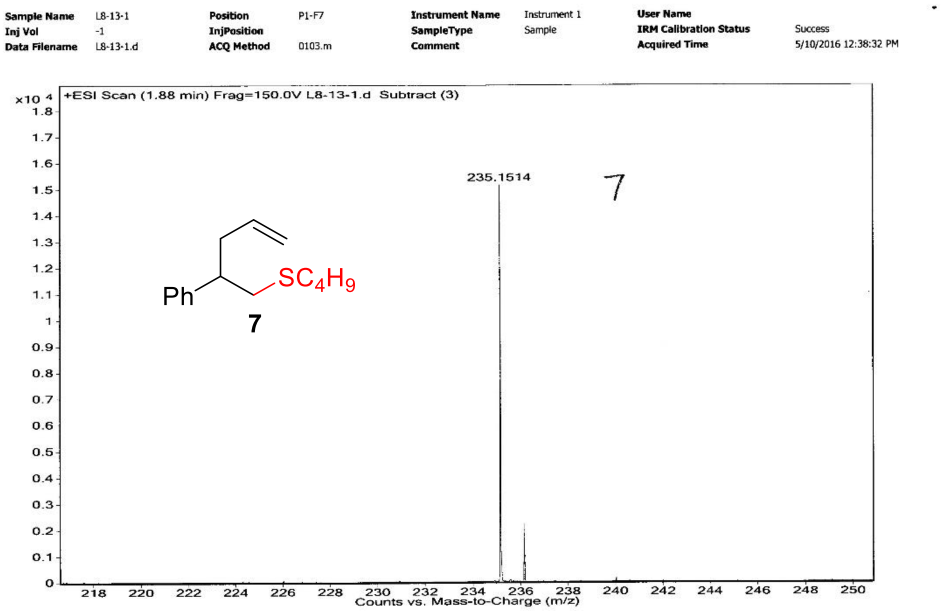

HRMS (ESI, m/z) calcd for $C_{15}H_{22}S$ $[M+H]^+$ 235.1515, found 235.1514.

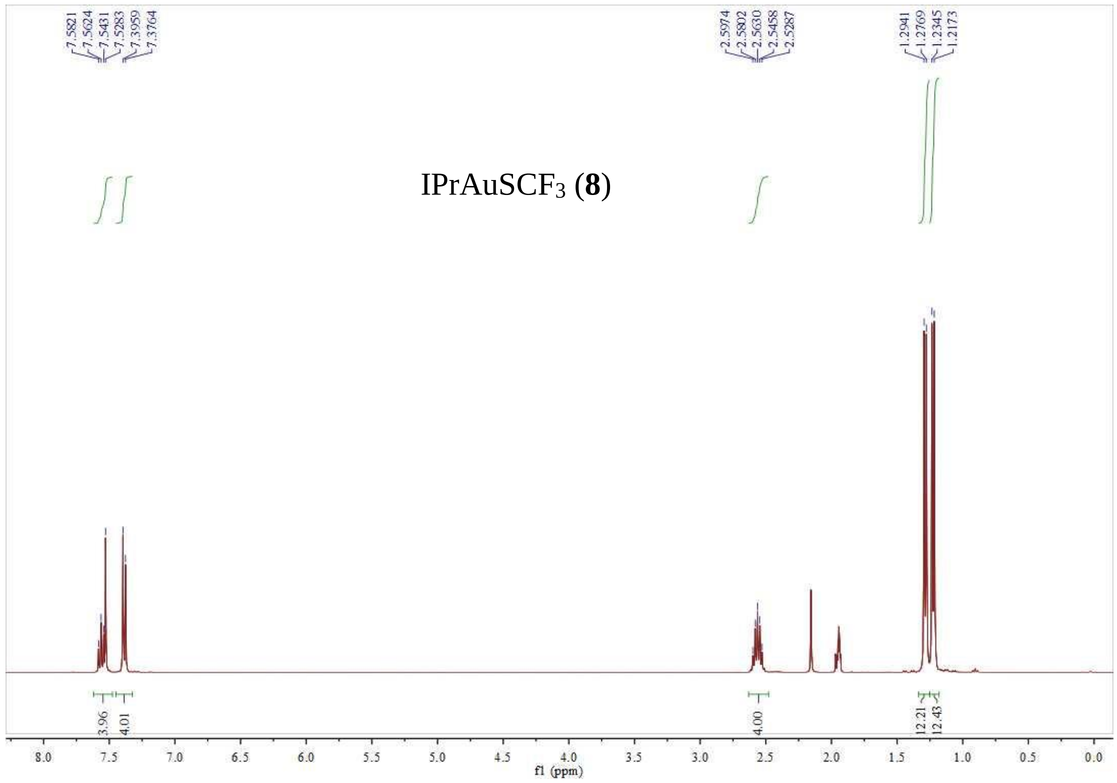

7.5821
7.5624
7.5431
7.5283
7.3959
7.3764
2.5974
2.5802
2.5630
2.5458
2.5287
1.2941
1.2769
1.2345
1.2173
IPrAuSCF3 (8)
3.96
4.01
4.00
12.21
12.43
8.0 7.5 7.0 6.5 6.0 5.5 5.0 4.5 4.0 3.5 3.0 2.5 2.0 1.5 1.0 0.5 0.0
f1 (ppm)

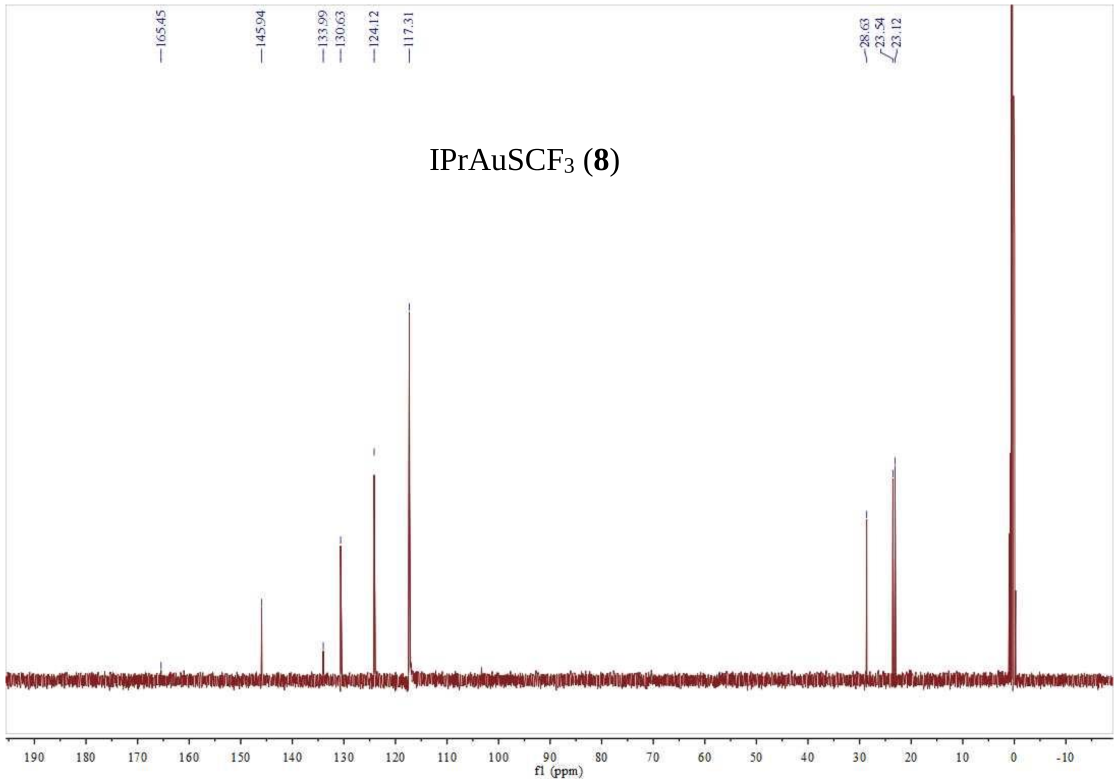

165.45
145.94
133.99
130.63
124.12
117.31
28.63
23.54
23.12
IPrAuSCF3 (8)
190 180 170 160 150 140 130 120 110 100 90 80 70 60 50 40 30 20 10 0 -10
f1 (ppm)

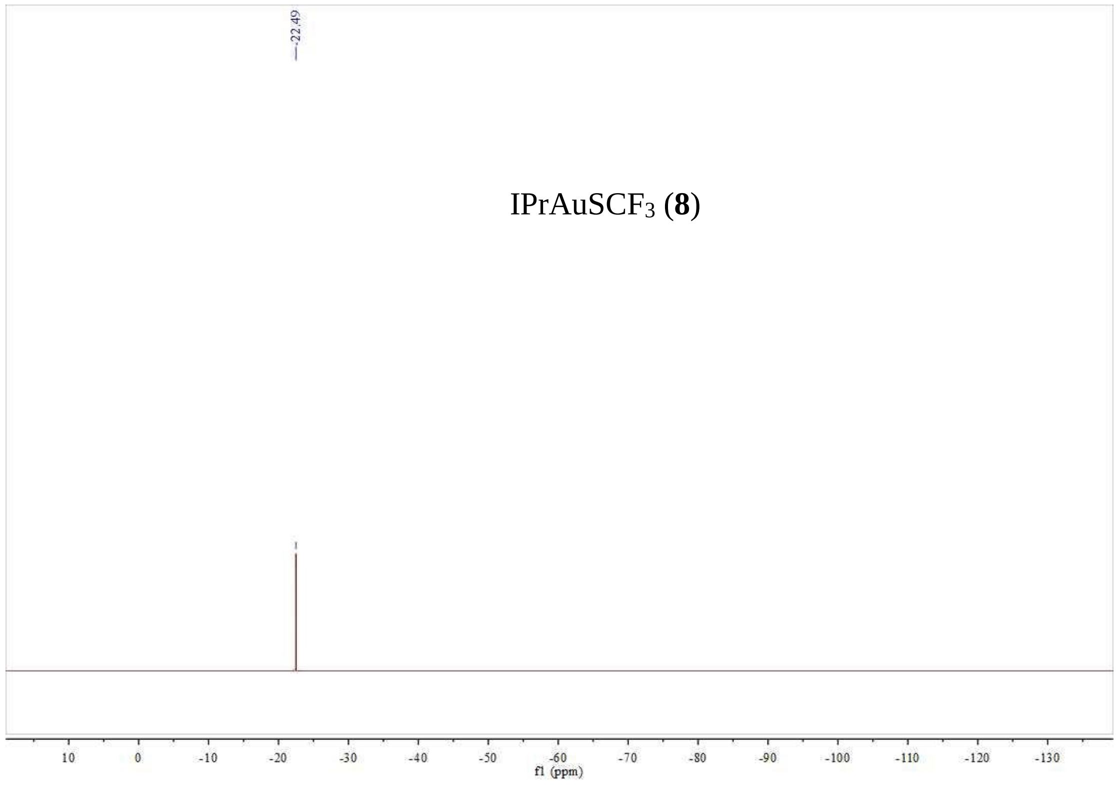

-22.49
IPrAuSCF3 (8)
10 0 -10 -20 -30 -40 -50 -60 -70 -80 -90 -100 -110 -120 -130
f1 (ppm)

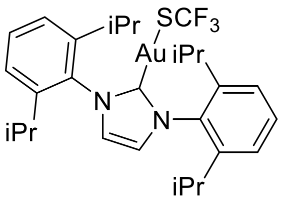

Chemical Formula: $C_{28}H_{36}AuF_3N_2S$
Exact Mass: 686.2217
Molecular Weight: 686.6318
m/z: 686.2217 (100.0%), 687.2250 (30.3%), 688.2175 (4.5%), 688.2284 (3.0%), 688.2284 (1.4%), 689.2208 (1.4%)
Elemental Analysis: C, 48.98; H, 5.28; Au, 28.69; F, 8.30; N, 4.08; S, 4.67

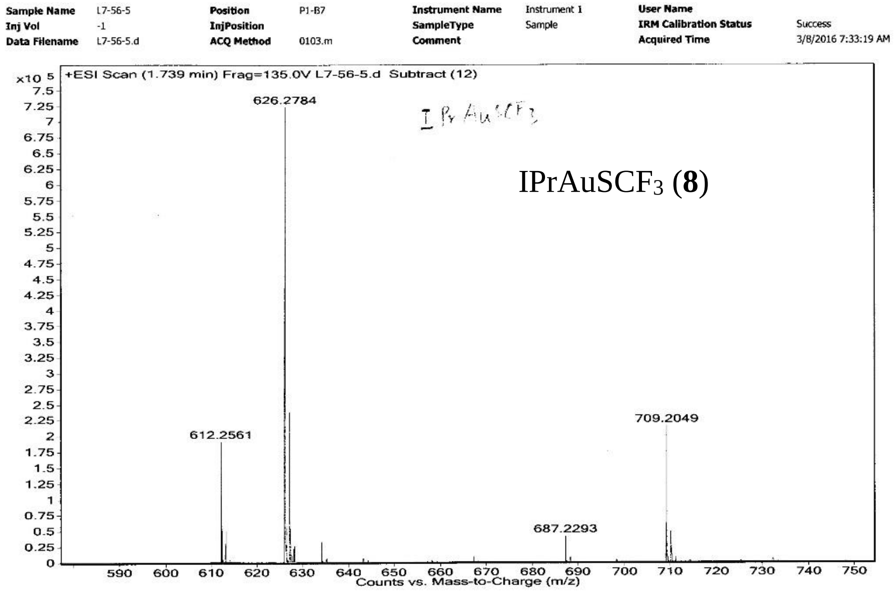

HRMS (ESI, m/z) calcd for $C_{28}H_{36}Au\ F_3N_2S$ $[M+H]^+$ 687.2290, found 687.2293.

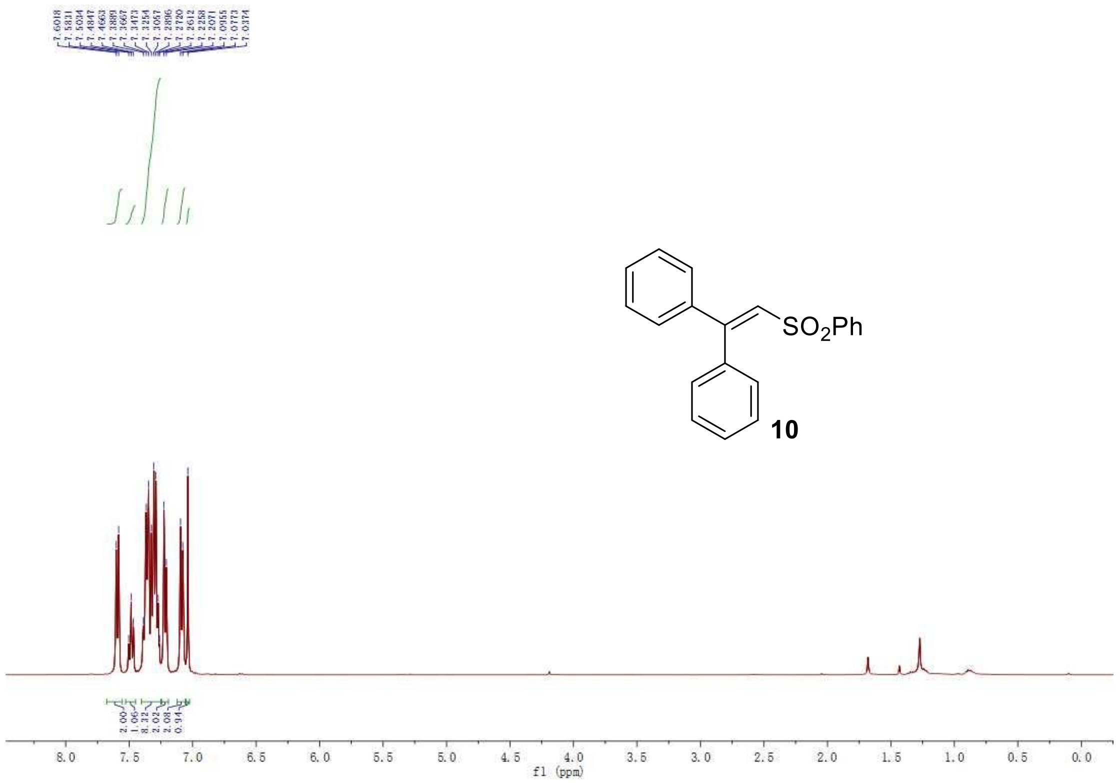
SO2Ph
10
8.0
7.5
7.0
6.5
6.0
5.5
5.0
4.5
4.0
3.5
3.0
2.5
2.0
1.5
1.0
0.5
0.0
f1 (ppm)
